# Catalytic Enantioselective Synthesis of Heterocyclic Vicinal Fluoroamines by Using Asymmetric Protonation: Method Development and Mechanistic Study†

**DOI:** 10.1002/chem.202002543

**Published:** 2020-08-18

**Authors:** Matthew W. Ashford, Chao Xu, John J. Molloy, Cameron Carpenter‐Warren, Alexandra M. Z. Slawin, Andrew G. Leach, Allan J. B. Watson

**Affiliations:** ^1^ EaStCHEM School of Chemistry University of St Andrews North Haugh St Andrews Fife KY16 9ST UK; ^2^ School of Health Sciences University of Manchester Oxford Road Manchester M13 9PL UK

**Keywords:** asymmetric synthesis, Brønsted acids, catalysis, fluorine, heterocycles

## Abstract

A catalytic enantioselective synthesis of heterocyclic vicinal fluoroamines is reported. A chiral Brønsted acid promotes aza‐Michael addition to fluoroalkenyl heterocycles to give a prochiral enamine intermediate that undergoes asymmetric protonation upon rearomatization. The reaction accommodates a range of azaheterocycles and nucleophiles, generating the C−F stereocentre in high enantioselectivity, and is also amenable to stereogenic C−CF_3_ bonds. Extensive DFT calculations provided evidence for stereocontrolled proton transfer from catalyst to substrate as the rate‐determining step, and showed the importance of steric interactions from the catalyst's alkyl groups in enforcing the high enantioselectivity. Crystal structure data show the dominance of noncovalent interactions in the core structure conformation, enabling modulation of the conformational landscape. Ramachandran‐type analysis of conformer distribution and Protein Data Bank mining indicated that benzylic fluorination by this approach has the potential to improve the potency of several marketed drugs.

## Graphical abstract

**Chiral Brønsted acid catalysis** enables the synthesis of heterocyclic vicinal fluoroamines by asymmetric protonation. The reaction accommodates a range of azaheterocycles and nucleophiles, generating the C−F stereocentre in high enantioselectivity, and allows the formation of chiral C−CF_3_ bonds. DFT calculations provided insight into the reaction mechanism, and conformational control of the products is elucidated.

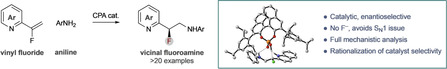

## Introduction

The incorporation of fluorine into organic compounds is prominent in the pharmaceutical, agrochemical, and materials industries.^1^ The unique characteristics of the C−F bond enable modulation of physicochemical properties while mitigating steric contributions.^2^ A key attribute is the intrinsic polarity of the C−F bond, which can induce conformational changes through electrostatic and dipole interactions with neighbouring functional groups. Installation of a chiral C−F bond with a vicinal relationship to a heteroatom or electron‐withdrawing group is particularly valuable, as exploitation of the *gauche* effect allows predictable conformational control.^3^ For example, fluorinated phenethylamines are especially valuable given the demonstrable utility of this compound class within bioactive molecules,^4^ and the topological control afforded by the *gauche* effect can enable bespoke biological target engagement.^5^ Similarly, fluorine is often considered as a minimal change to block metabolism, for example of labile benzyl positions.^2^, ^6^ Given the stereoselective nature of most metabolic processes, substitution of a specific C−H for C−F in a methylene represents a very efficient means of benefiting from this effect, thus the introduction of fluorine in a stereoselective fashion holds significant appeal.

**Scheme 1:**
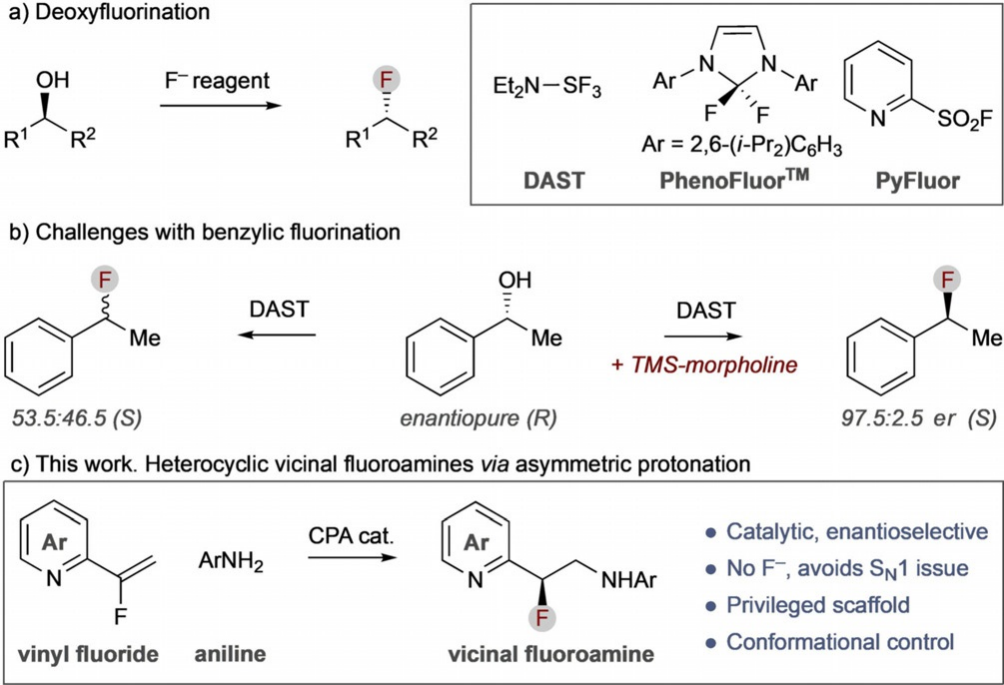
a) Deoxyfluorination and exemplar reagents. b) Enantioerosion during deoxyfluorination of benzylic alcohols. c) This work. Chiral heterocyclic vicinal fluoroamines by asymmetric protonation.

Deoxyfluorination of alcohols is the most common method to install C(sp^3^)−F bonds, and numerous reagents have been developed to facilitate this transformation (e.g., Scheme 1 a).^7^, ^8^ A noted problem with the introduction of a benzyl fluoride by deoxyfluorination is the propensity for stereochemical erosion due to variable contribution of S_N_1 pathways (e.g., Scheme 1 b).8a, 9 This can be ameliorated in some cases by altering the deoxyfluorination reagent in situ by using specific additives; however, this general problem arises specifically from the nucleophilic fluorinating reagents commonly used. An alternative strategy for C(sp^3^)−F bond formation that avoids S_N_2 is by asymmetric protonation of prochiral C(sp^2^)−F centres; however, there are limited examples of this and none for benzylic C(sp^3^)−F.^10^

Herein, we present a method for the enantioselective synthesis of benzylic C(sp^3^)−F vicinal fluoroamines by asymmetric protonation of in situ‐generated prochiral fluoroenamines (Scheme 1 c).^10^, ^11^, ^12^ This method allows the formation of a new C−N bond and a benzylic stereogenic C−F bond in a single catalytic reaction, thereby providing direct modular access to chiral heterocyclic vicinal fluoroamines from readily accessible vinyl fluoride precursors.^13^ As the process does not rely upon the use of a fluorinating reagent, the issue of stereochemical erosion is avoided. The mechanism is fully investigated, with refinement of previous proposals, and we also show how the relationship of the azaheterocycle, amine, and C−F bonds provides unique conformational control, which could offer benefits in drug design by better alignment with bound ligand conformation.

## Results and Discussion

### Method development

**Scheme 2:**
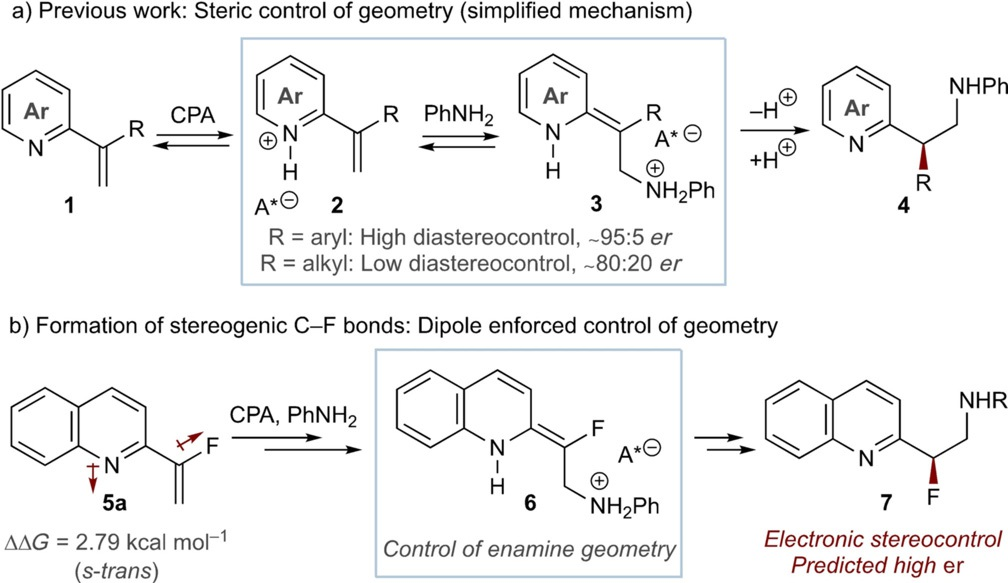
a) Our previous work and simplified mechanism indicating asymmetric induction is a function of enamine geometry control. b) Preliminary DFT conformational analysis suggesting high enamine diastereoselectivity.

We have previously shown that Brønsted acid catalysis enables conjugate addition and highly selective asymmetric protonation of prochiral enamines (Scheme 2 a).^14^ However, this was only amenable with the steric control of enamine geometry afforded by aryl substituents at the α‐carbon: alkyl substituents led to poor geometry control of the intermediate enamine **3**, resulting in lower enantioinduction in product **4**. Although fluorine has a small steric footprint, we postulated that dipole minimization might provide an alternative selectivity determinant (Scheme 2 b). Indeed, preliminary DFT studies highlighted the preferred *s‐trans* geometry for benchmark starting material **5 a**, which was anticipated to assist geometrical control of the developing fluoroenamine **6** and enhancing enantioinduction in **7**.

Based on this hypothesis, a benchmark process was established whereby **5 a** was subjected to aniline (**8**), and Brønsted acid catalyst **9 a** (Table 1; a range of catalysts were evaluated, vide infra and see the Supporting Information). Optimization of reaction parameters delivered a system that afforded the desired product **7 a** in high conversion and enantioselectivity (82 % and 96:4 *er*; entry 1). Several observations relating to optimization were noted (see the Supporting Information for full details and additional experiments). Ethereal solvents (THF, CPME) were particularly effective (entries 1 and 2), with other solvents affording good to excellent conversion but with notably poorer enantioselectivity (entries 3 and 4). Lowering the reaction temperature from −10 to −20 °C had little effect on enantioselectivity but impacted reaction efficiency (entry 5). A similar effect was observed by lowering catalyst loading, where 10 mol % was less efficient but maintained selectivity (entry 6). Control experiments supported a catalyst‐promoted reaction that lacked background reactivity (entry 7).

**Table 1:** Reaction development.

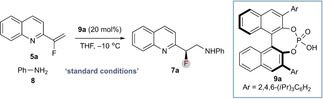		
	Deviation from ‘standard conditions’	Yield [%] (*er* (*R*:*S*))^[a]^
1	None	82 (96:4)^[b]^
2	CPME	89 (95:5)^[b]^
3	PhMe	95 (89:11)
4	CH_2_Cl_2_	70 (75:25)
5	−20 °C	40 (96:4)
6	10 mol % **9 a**, −20 °C	37 (95:5)
7	0 mol % **9 a**, −20 °C	0 (–)

[a] Determined by HPLC using an internal standard. [b] Isolated yield.

**Scheme 3:**
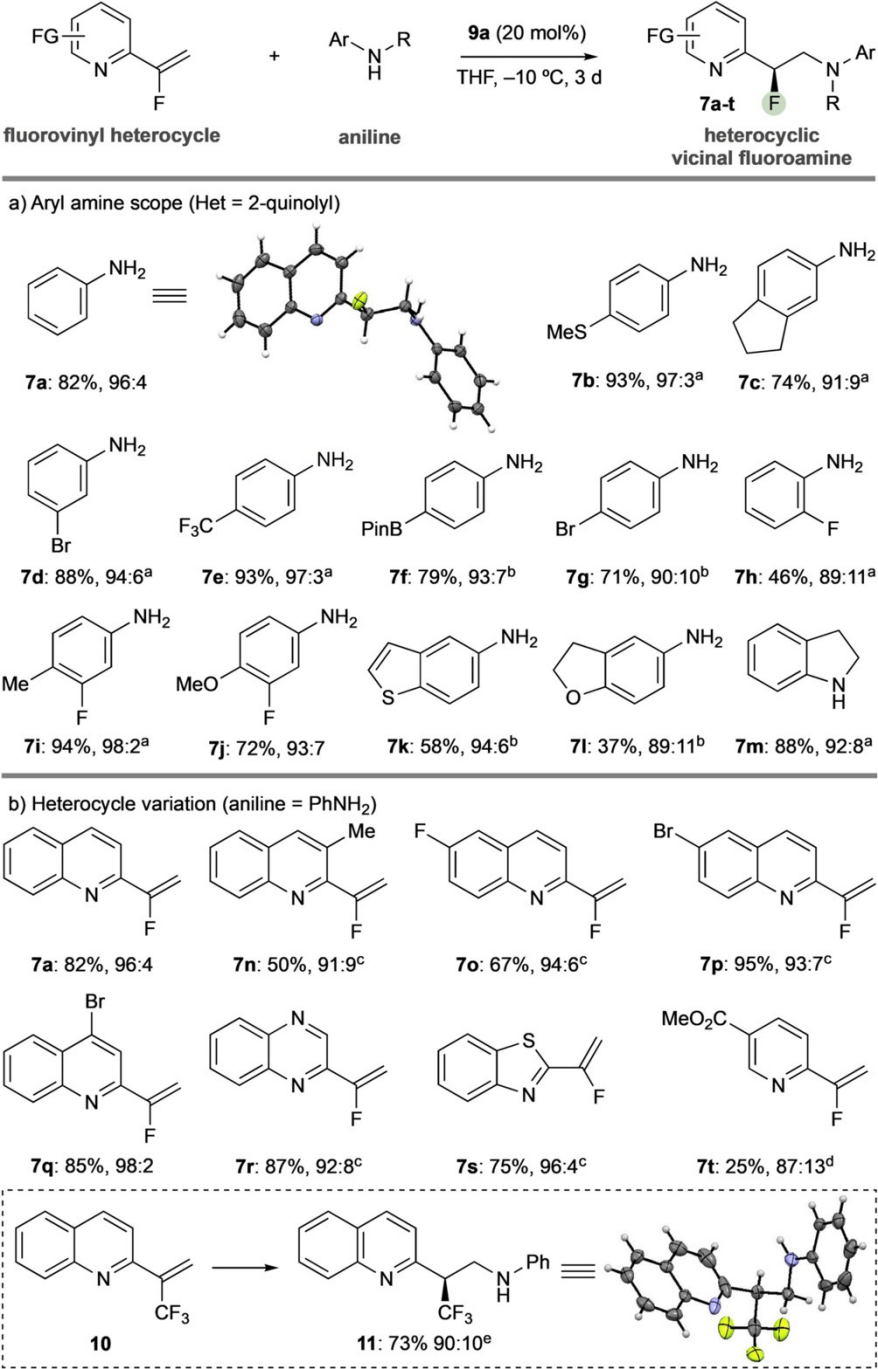
Substrate scope and isolated yields. Enantiomeric ratios determined by HPLC analysis on a chiral stationary phase. See the Supporting Information for details. [a] CPME, −20 °C, 5 d. [b] −20 °C, 5 d. [c] CPME, −10 °C. [d] CPME, RT. [e] 1 equiv of **9 a**, CPME, −50 °C.

The scope of the reaction was investigated (Scheme 3). A range of aryl amine nucleophiles was accommodated with variation in functional group (e.g., halides, alkyl groups, BPin, heterocycles) and regiochemical substitution (*ortho*, *meta*, *para*) was typically accommodated while maintaining selectivity (Scheme 3 a). Additionally, substitution on the aniline nitrogen was tolerated (**7 m**). It should be noted that the choice of solvent was important for conversion, due to solubility: product precipitation as the reaction progressed became problematic for certain substrates; however, changing ethereal solvent based on substrate (THF or CPME) resolved this issue.

A range of vinylheterocycles was also generally well accommodated (Scheme 3 b). Variation in substitution of the benchmark 2‐vinylquinoline was straightforward (**7 a**, **7 n**–**q**) and the reaction tolerated quinoxaline (**7 r**), benzothiazole (**7 s**), and pyridine (**7 t**), with the latter a significantly more challenging substrate due to its higher dearomatization barrier, hence requiring a high temperature for the reaction to proceed, which negatively affects enantioinduction. Significantly, the reaction also allows enantioselective formation of stereogenic C−CF_3_ bonds (**11**); however, catalyst loading had to be increased and reaction temperature decreased to overcome a significant nonselective background reaction observed for this substrate (see the Supporting Information).

### Mechanistic analysis

**Scheme 4:**
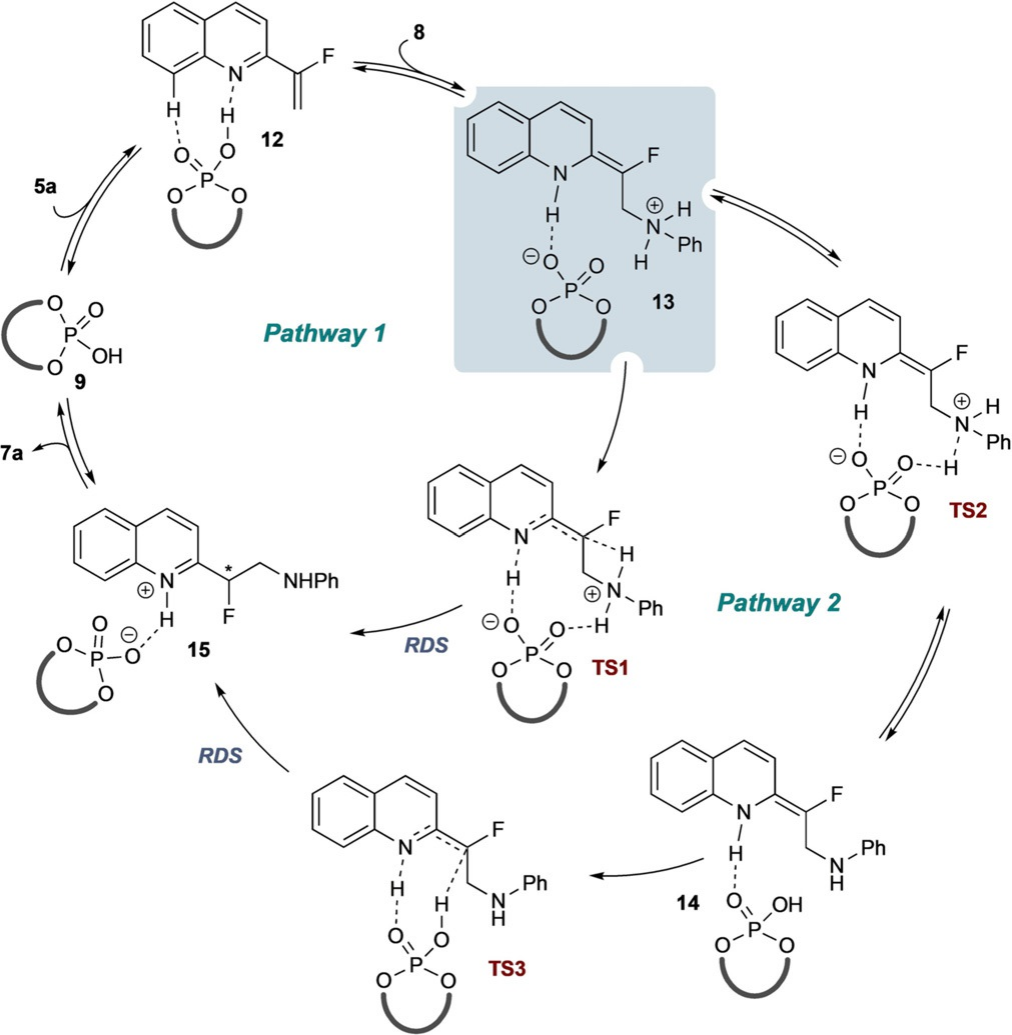
Proposed mechanistic pathways. Pathway 1: direct proton transfer from anilinium to a prochiral centre. Pathway 2: proton transfer to/from phosphate. RDS=rate determining step; TS=transition state.

Two main mechanistic pathways are possible for the key asymmetric protonation event (Scheme 4). The initial events common to both pathways involve reversible protonation of the substrate (**5 a**) by the catalyst to provide LUMO‐lowered intermediate complex **12** and enabling reversible aza‐Michael using PhNH_2_ (**8**) to deliver key intermediate **13**. Two mechanistic pathways are then possible from this intermediate: pathway 1 proceeds via direct stereocontrolled proton transfer from the anilinium via **TS1** and delivers the product‐catalyst complex **15**, which subsequently liberates the product (**7 a**). Alternatively, in pathway 2 proton transfer from the anilinium of **13** to the phosphate (via **TS2**) delivers **14**, which undergoes stereocontrolled proton transfer via **TS3** to deliver **15**. In our previous report,^14^ computational analysis supported pathway 1, with selectivity arising from good shape and electrostatic complementarity between the catalyst and **TS1** leading to the observed enantiomer. These purely quantum mechanical studies did not yield transition states that would have supported pathway 2 (or other alternative mechanisms). A series of kinetic isotope effect experiments were conducted via the use of ^15^N‐aniline and PhND_2_. However, these proved inconclusive, with independent rate experiments (see the Supporting Information) resulting in observed ^14/15^N KIE of approximately 0.8 and H/D KIE of approximately 1.8, which might be affected by the pre‐RDS equilibrium associated with this reaction.

**Figure 1:**
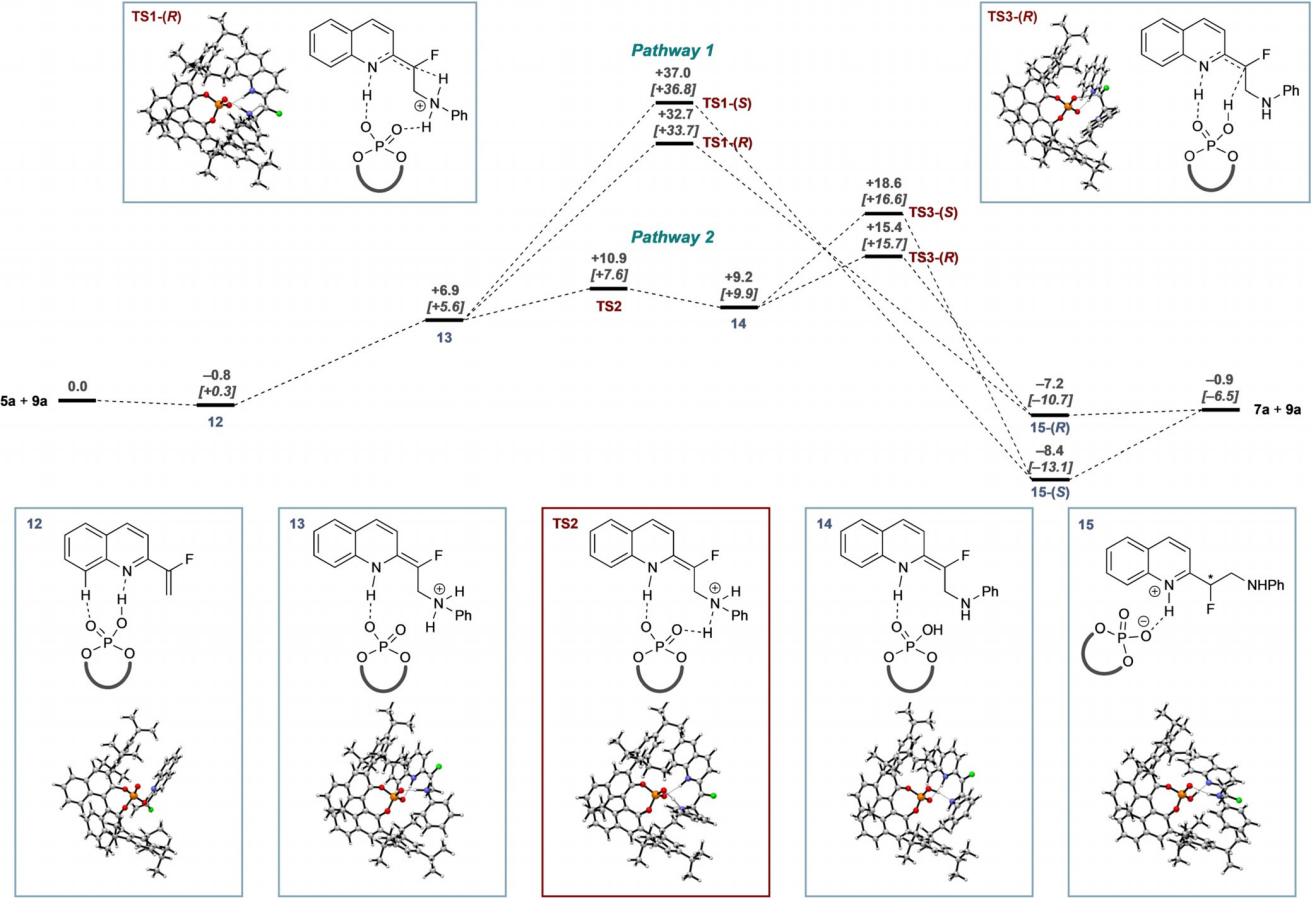
Free‐energy profile comparison [ONIOM (B3LYP/6‐31G**:UFF; single‐point energy M06‐2X/6‐31G**(+PCM for Et_2_O)]. Two pathways are compared: Pathway 1: direct proton transfer and pathway 2: proton transfer to and from the phosphate. Free energies in kcal mol^−1^ are reported relative to separated substrate and catalyst bound to THF. All calculations were performed in Gaussian09, and free energies at −20 °C and 1 m concentration were obtained by using goodvibes.^16^

Goodman and others have shown that catalysis by BINOL‐derived catalysts, such as **9 a**, can be studied effectively and efficiently by QM/MM ONIOM calculations where the quantum mechanical aspects are described by B3LYP/6‐31G** and the molecular mechanics by UFF.^15^ Accordingly, a more exhaustive theoretical exploration was undertaken using this approach (Figure 1 and Supporting Information).

Experimentally, no background reaction was observed, which was consistent with DFT calculations that indicated a prohibitively high barrier for direct reaction of **5 a** with **8** (see the Supporting Information).

Complexation of **5 a** with **9 a** to give **12** is moderately favourable, with the preferred dipole‐induced *s‐trans* conformation of **5 a** also retained in **12**. This initial complex is held together by a H‐bond (OH**⋅⋅⋅**N=1.63 Å) and a weak *peri* CH**⋅⋅⋅**O=P interaction (2.40 Å). Complex **12** then undergoes dearomatizing aza‐Michael addition to deliver **13**, where the loss of aromaticity is compensated for by the formation of a tightly bound ionic interaction between the anilinium NH and phosphate (P=O**⋅⋅⋅**HNHPH=1.38 Å and P=O**⋅⋅⋅**HN_quin_=1.71 Å). Rearrangement within this complex by proton transfer from the anilinium to the phosphate involves a low barrier and yields a complex of the enamine (**14**) that is higher in energy than reactants. All of these steps are therefore strongly reversible and no significant concentration of any of the intermediates subsequent to quinoline complexation would be expected—this was confirmed by parallel NMR experiments (see the Supporting Information).

Transition states leading to each low‐energy conformation of complex **15**‐(*R*) and **15**‐(*S*) were optimized leading to an array of conformations for each of **TS1** and **TS3**. Consistent with our previous report,^14^ pathway 1, direct proton transfer to the prochiral centre was identified (**TS1**). This process has a significant barrier (+35 kcal mol^−1^) but is predicted to be highly stereoselective (ΔΔ*G* ^≠^=4.4 kcal mol^−1^, >99:1 *er*) in favour of the experimentally observed enantiomer. Although this rationalizes the stereoselectivity of the process, it is not consistent with the experimental rate of reaction.

However, pathway 2 was more consistent with the experimentally observed rate. The key step, in which the stereochemistry is generated, involves protonation of the enamine by the POH in complex **14** via **TS3** and exhibits a clear preference for the experimentally observed enantiomer (ΔΔ*G* ^≠^ = +3.5 kcal mol^−1^), which arises from geometrical restrictions between the catalyst and enamine in the developing transition states **TS3**‐(*R*) and **TS3**‐(*S*) (vide infra). The *i*Pr substituents of catalyst **9 a** are also particularly important for imposing this geometrical restriction (vide infra: Table 2 and Figure 3, below). This mechanistic overview reveals that the catalyst provides its effect by acting as both acid and base at each stage as required and does so in a way that imposes specific shape requirements on the substrate that interplay with the polar interactions that hold the complex together.

**Table 2:** Catalyst structure vs. enantioselectivity.

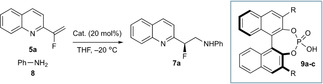			
	Catalyst	R	Yield [%] (*er* (*R*:*S*))^[a]^
1	**9 a**	2,4,6‐(*i*‐Pr)_3_C_6_H_2_	40 (96:4)
2	**9 b**	2,4,6‐(Me)_3_C_6_H_2_	25 (88:12)
3	**9 c**	Ph	11 (58:42)

[a] Determined by HPLC using an internal standard.

Based on these results, a complete reinvestigation of the computational analysis of our previous process using aryl substituents (Scheme 2 a)^14^ using the approach delineated above suggests that Pathway 2 is a more likely reaction mechanism in this process. The full profile for this reaction is provided in the Supporting Information.

**Figure 2:**
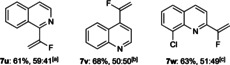
Control substrates. Reaction conditions as per Scheme 3 unless noted. [a] CPME. [b] CPME, RT. [c] CPME, 40 °C.

Control substrates **7 u**–**7 w** provided additional support of the DFT conclusions (Figure 2).

Despite moderate yields of product for each, enantioinduction was poor, which arises from features that are not well‐tolerated in the lowest energy transition state. Substrate **7 u** places the fused phenyl ring in a position that clashes with the *i*Pr groups of **9 a** in **TS3**. In contrast, **7 v** prevents the required simultaneous interactions of **5 a** and **8** with **9 a** and also lacks the dipole‐induced geometry control. Lastly, the essential enamine NH‐OP H‐bond in **TS3** is impaired by the adjacent chlorine in substrate **7 w**, weakening the association between the substrate and catalyst.

**Figure 3:**
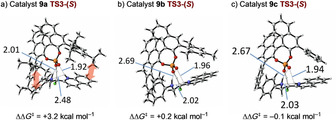
Structures equivalent to **TS3**‐(*S*) for catalysts **9 a**, **9 b**, and **9 c**.

With regards to the optimal catalyst, a catalyst survey demonstrated the superior level of asymmetric induction using **9 a** (Table 2; see the Supporting Information for full details). To determine the origin of this enhanced selectivity, we analysed **9 a** in comparison to the related 3,3′‐mesityl (**9 b**) and ‐phenyl (**9 c**) analogues (Figure 3). As the stereodirecting group on the catalyst is reduced in size from **9 a** to **9 b** and **9 c**, there is a general tendency for the barrier for the catalysed reaction to increase (from 16.2 to 16.6 and 18.5 kcal mol^−1^, respectively), resulting in the observed diminished conversion. This is accompanied by a sharp erosion in ΔΔ*G* ^≠^ between **TS3**‐(*R*) and **TS3**‐(*S*)—the energy associated with **TS3**‐(*R*) remains similar for all three, and this erosion is principally driven by a change in energy of **TS3**‐(*S*). This is highlighted in the preferred conformation of each of the three structures equivalent to **TS3**‐(*S*) (Figure 3). The red arrows (Figure 3 a) indicate where the bulk of the *i*Pr groups of **9 a** press against both ends of the bound substrate. This causes the break‐up of an intramolecular H‐bond between the aniline nitrogen and the NH of the nitrogen arising from the quinoline (2.48 Å for **9 a**, but 2.02 and 2.03 Å for **9 b** and **9 c**, respectively); this interchanges with an interaction between the aniline NH and a phosphate oxygen (2.01 Å for **9 a**, but 2.69 and 2.67 Å for **9 b** and **9 c**, respectively). The combination of the steric clashing and this change in hydrogen bonding pattern clearly disfavours **TS3**‐(*S*) compared to **TS3**‐(*R*) for **9 a**; this difference is significantly reduced for **9 b** and **9 c**.

In line with experimental observations, the computational model also confirms a lower rate of catalysed reaction for **5 t**, associated with the larger dearomatization barrier (see the Supporting Information). The observed significant background reaction for **10** was also investigated computationally, confirming the accelerating role of the LUMO‐lowering CF_3_ unit as previously observed for other Michael acceptors.^17^

### Implications for conformational control

**Figure 4:**
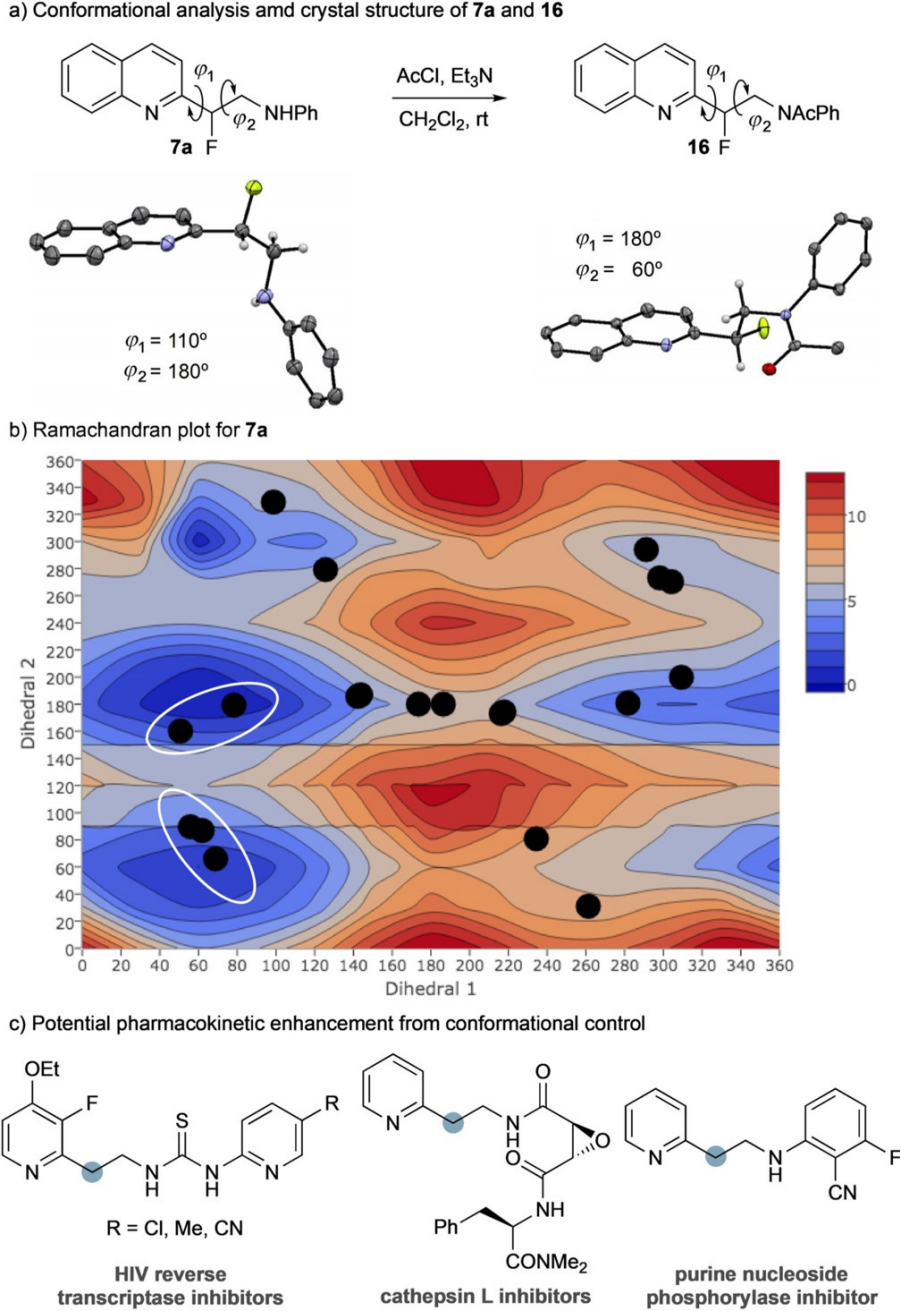
a) Conformational analysis and crystal structures of **7 a** and **16**. b) Ramachandran plot for **7 a**. c) Drug‐like molecules in the PDB that might benefit from conformational control induced by benzylic fluorination.

The value of the substructures accessible using the developed protocol was explored by investigating their conformational properties. The crystal structure of **7 a** shows an *anti* relationship between C−F and the aniline nitrogen (dihedral angle=179°), which is likely preferred in comparison to the *gauche* due to a favourable N_pz_ **⋅⋅⋅**σ*_C‐F_ interaction (Figure 4). The C−F bond is almost perpendicular to the carbon framework of the quinoline (dihedral angle=107°), which we believe arises due to a favourable σ*_C‐F_ **⋅⋅⋅**π_Ar_ interaction competing with C−F/N_quin_ dipole minimization.^18^ Hydrogen bonding of the quinoline nitrogen with a hydrogen bond donor would reduce this dipole and is a key feature of this system: the crystal structure has an intermolecular hydrogen bond between the quinoline nitrogen and the HNPh in an adjacent molecule.

To reduce the impact of hydrogen bonding and any N_pz_ interactions, **7 a** was acetylated to give **16** (Figure 4 a). The preference towards the *anti* configuration is diminished, with the *gauche* conformation noted in the crystal structure (FCCN_Aniline_ dihedral angle=58°). The dihedral angle between the C−F bond and quinoline nitrogen is 174°, explicitly affected by the C−F/N_Ar_ dipole minimization and no longer modulated by the other effects described.

Ramachandran plots for dihedral angles 1 and 2 of **7 a**, **16**, and the parent 2‐pyridylethylamine (not shown) were computed and reveal that the introduction of the benzylic fluoride has a profound effect on the overall conformational landscape (Figure 4 and Supporting Information). Compounds **7 a** and **16** display specific low‐energy conformations biased by the presence of the fluorine and that are likely to be populated in solution. This presents opportunities for application in drug discovery by improving binding affinity and selectivity by decreasing the population of alternative, less favourable conformations. The protein databank was searched for ligands that contain the 2‐pyridylethylamine substructure and the conformations that are populated in these crystal structures are mapped onto the Ramachandran plot for **7 a** (Figure 4 b, black dots). Five compounds in particular adopt a conformation that would be enhanced by the introduction of fluorine at the benzylic position (Figure 4 b, circled), including inhibitors of HIV reverse transcriptase,^19^ cathepsin L,^20^ and purine nucleoside phosphorylases (Figure 4 c).^21^ This highlights the value of this structural change for enhancing potency and selectivity of potential drug molecules.

## Conclusions

In summary, a Brønsted acid‐catalysed aza‐Michael/asymmetric protonation method for the synthesis of heterocyclic vicinal fluoroamines has been developed. The method allows access to stereogenic C−F bonds in high selectivity and on a selection of different heterocyclic templates. The method also translates to establishing stereogenic CF_3_ analogues. The origin of the reactivity and stereoinduction has been investigated by extended DFT calculations that have established a phosphate proton transfer as being more consistent with experimental observations than a direct proton‐transfer process. This has led to a revision of our interpretation of the mechanism associated with our previous report. Conformational control of the vicinal phenethylamine system has been interrogated by DFT and crystallography, identifying the likely preferred topologies in the solid and solution state. This might have strategic applications in drug discovery by introducing conformational bias to access conformations more like the bound state, illustrated with examples extracted from the PDB.^22^

## Conflict of interest

The authors declare no conflict of interest.

## Supporting information

As a service to our authors and readers, this journal provides supporting information supplied by the authors. Such materials are peer reviewed and may be re‐organized for online delivery, but are not copy‐edited or typeset. Technical support issues arising from supporting information (other than missing files) should be addressed to the authors.

Supplementary
