# Supplementary material for: Catalytic Enantioselective Synthesis of Heterocyclic Vicinal Fluoroamines by Using Asymmetric Protonation: Method Development and Mechanistic Study
Source: Chemistry. 2020 Aug 18;26(53):12249–55. doi: 10.1002/chem.202002543 (PMC7540707; doi:10.1002/chem.202002543)
Supplement: Supplementary file 1 — Supplementary [file CHEM-26-12249-s001.pdf]

Chemistry–A European Journal

Supporting Information

Catalytic Enantioselective Synthesis of Heterocyclic Vicinal Fluoroamines by Using Asymmetric Protonation: Method Development and Mechanistic Study**

Matthew W. Ashford,[a] Chao Xu,[a] John J. Molloy,[a] Cameron Carpenter-Warren,[a] Alexandra M. Z. Slawin,[a] Andrew G. Leach,*[b] and Allan J. B. Watson*[a]

Table of Contents

1. General **S3**
2. General Experimental Procedures **S4**
3. Optimization of Reaction Conditions **S6**
4. Catalyst ee *vs.* Product ee **S7**
5. Mechanistic Study **S8**
5.1 DFT Calculations S8
5.2 HRMS Study S12
5.2 Kinetic Isotope Effect Study S13
6. Ramachandran Plots **S15**
7. Characterization Data for Compounds **S17**
8. References **S65**
9. NMR Spectra **S66**
10. X-Ray Data **S122**

1. General

All reagents and solvents were obtained from commercial suppliers and were used without further purification unless otherwise stated. Purification was carried out according to standard laboratory methods.[1]

1.1 Purification of Solvents

Dry THF for reactions were obtained from a PureSolv SPS-400-5 solvent purification system. Et_2O, EtOAc, and petroleum ether 40-60 °C for purification purposes were used as obtained from suppliers without further purification.

1.2 Experimental Details

Reactions were carried out using conventional glassware (preparation of intermediates), 2 mL HPLC vials, or in capped 5, 10, and 20 mL microwave vials. The glassware was oven-dried (150 °C) and purged with N_2 before use. Purging refers to a vacuum/nitrogen-refilling procedure. Prior to use, Mg turnings were oven-dried at 150 °C overnight. TRIP refers to 3,3′-bis(2,4,6-triisopropylphenyl)-1,1′-binaphthyl-2,2′-diyl hydrogenphosphate. Room temperature (rt) was generally *ca.* 18 °C. Reactions were carried out at elevated temperatures using a temperature-regulated hotplate/stirrer with a sand bath. Reactions were carried out at −10, −20, and −50 °C using an isopropanol bath cooled by a Thermo Haake EK90 cryocooler. Reactions were carried out at 0 °C using an ice/water bath.

1.3 Purification of Products

Thin layer chromatography was carried out using Merck silica plates coated with fluorescent indicator UV254. These were analyzed under 254 nm UV light, developed using potassium permanganate or vanillin solutions. Normal phase flash chromatography was carried out using ZEOprep 60 HYD 40-63 μm silica gel.

1.4 Analysis of Products

Fourier Transformed Infra-Red (FTIR) spectra were obtained on a Shimadzu IRAffinity-1 machine. ^{19}F NMR spectra were obtained on either a Bruker AV 400 spectrometer at 376 or 377 MHz, or a Bruker AV 500 spectrometer at 470 MHz, respectively. ^{11}B NMR spectra were obtained on a Bruker AV 400 spectrometer at 128 MHz. ^{1}H and ^{13}C NMR spectra were obtained on either a Bruker AV 400 at 400 MHz and 101 MHz, respectively, or Bruker DRX 500 at 500 MHz and 126 MHz, respectively. Chemical shifts are reported in ppm and coupling constants are reported in Hz with $CDCl_3$ referenced at 7.26 (^{1}H) and 77.0 ppm (^{13}C) and DMSO-d_6 referenced at 2.50 (^{1}H) and 39.5 (^{13}C). ^{11}B NMR spectra are referenced to $BF_3{\cdot}Et_2O$. Unless otherwise stated, J refers to $^{3}J_{HH}$ and J_{CF} in ^{1}H and ^{13}C NMR, respectively. High-resolution mass spectra were obtained either through analysis at the EPSRC UK National Mass Spectrometry Facility at Swansea University, or through analysis at the University of St Andrews. High performance liquid chromatography (HPLC) was performed on an Agilent 1200 series HPLC using a chiral stationary phase column (column, Daicel Co. CHIRALCEL OJ-H, or CHIRALPAK IA; eluent: *n*-hexane/*i*-PrOH). All solvents used were HPLC-grade solvents purchased from Fisher. The column employed and the respective solvent mixture are indicated for each experiment. Optical rotations were obtained on a Perkin Elmer Model 341 polarimeter.

2. General Experimental Procedures

General Procedure A: Aza-Michael reaction catalyzed using a chiral acid (THF as solvent). For example, preparation of 7a.

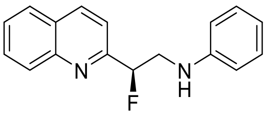

A 2 mL HPLC vial was charged with 2-(1-fluorovinyl)quinoline (17.3 mg, 0.10 mmol, 1.0 equiv), and (*S*)-TRIP catalyst (15.1 mg, 0.02 mmol, 20 mol%). The vial was then capped and purged with N_2 before adding THF (200 µL, 0.5 M). The reaction mixture was cooled to −10 °C for 15 min before the addition of aniline (27 µL, 0.30 mmol, 3.0 equiv). The resulting mixture was then allowed to stir at −10 °C for 3 d before being quenched by sat. aq. K_2CO_3 solution (2 mL). The phases were separated and the aqueous phase extracted with EtOAc (5 mL × 3). The organic phases were combined, dried over Na_2SO_4, filtered, and concentrated *in vacuo*. Purification of the crude *via* flash chromatography (silica gel, EtOAc:petroleum ether, 5:95 to 10:90) gave the desired product as a pale yellow oil (21.8 mg, 82%, 96:4 e.r.).

General Procedure B: Aza-Michael reaction catalyzed using a chiral acid (CPME as solvent). For example, preparation of 7b.

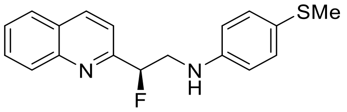

A 2 mL HPLC vial was charged with 2-(1-fluorovinyl)quinoline (17.3 mg, 0.10 mmol, 1.0 equiv), and (*S*)-TRIP catalyst (15.1 mg, 0.02 mmol, 20 mol%). The vial was then capped and purged with N_2 before adding CPME (200 µL, 0.5 M). The reaction mixture was cooled to −20 °C for 15 min before the addition of 4-(methylthio)aniline (41.7 mg, 0.3 mmol, 3.0 equiv). The resulting mixture was then allowed to stir at −20 °C for 5 d before being quenched by sat. aq. K_2CO_3 solution (2 mL). The phases were separated and the aqueous phase extracted with EtOAc (5 mL × 3). The organic phases were combined, dried over Na_2SO_4, filtered, and concentrated *in vacuo*. Purification of the crude *via* flash chromatography (silica gel, EtOAc:petroleum ether, 5:95 to 10:90) gave the desired product as a pale yellow oil (29.0 mg, 93%, 97:3 e.r.).

General Procedure C: Hiyama coupling. For example, preparation of 5a.

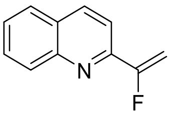

Prepared according to adapted literature procedure.[2]

An oven dried, 20 mL microwave vial was charged with 2-bromoquinoline (207 mg, 1.00 mmol, 1.0 equiv), (1-fluorovinyl)(methyl)diphenylsilane (356 mg, 1.5 mmol, 1.5 equiv), $Pd(PPh_3)_4$ (57.8 mg, 0.05 mmol, 5 mol%), CuI (9.7 mg, 0.05 mmol, 5 mol%) and CsF (357 mg, 2.35 mmol, 2.35 equiv). The microwave vial was then capped and purged with N_2 before addition of DMF (10 mL, 0.1 M). The reaction mixture was then allowed to stir at rt for 3.5 h. The reaction was quenched with water (20 mL)

and diluted with Et_2O (20 mL). The organic phase was separated, washed with water (2 × 10 mL) and brine (10 mL), dried over Na_2SO_4, filtered, and concentrated *in vacuo*. Purification of the crude *via* flash chromatography (silica gel, Et_2O:petroleum ether, 1:99) gave the desired product as a pale yellow oil (161 mg, 93%).

General Procedure D: Catalyst e.r. vs product e.r.

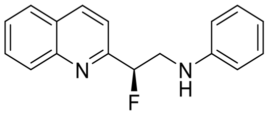

A 2 mL HPLC vial was charged with 2-(1-fluorovinyl)quinoline (17.3 mg, 0.10 mmol, 1.0 equiv), and different ratios of (*S*)- and (*R*)-TRIP catalyst (15.1 mg, 0.02 mmol, 20 mol%) (cat. e.r.: 60:40, 70:30, 80:20, 90:10 and 100:0). The vial was then capped and purged with N_2 before adding THF (200 µL, 0.5 M). The reaction mixture was cooled to −10 °C for 15 min before the addition of aniline (27 µL, 0.30 mmol, 3.0 equiv). The resulting mixture was then allowed to stir at −10 °C for 3 d before being quenched by sat. aq. K_2CO_3 solution (1 mL). The phases were separated and the aqueous phase was extracted with EtOAc (10 mL × 3). The organic phases were combined, dried over Na_2SO_4, filtered, and concentrated *in vacuo*. The e.r. of the product was determined by HPLC with an internal standard.

General Procedure E: Kinetic isotope effect

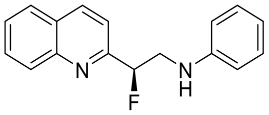

A 2mL HPLC vial was charged with 2-(1-fluorovinyl)quinoline (17.3 mg, 0.10 mmol, 1.0 equiv), (S)-TRIP catalyst (15.1 mg, 0.02 mmol, 20 mol%), and aniline (27 µL, 0.30 mmol, 3.0 equiv). The vial was then capped and purged with N_2 before adding d_8-THF (400 µL, 0.25 M). The resulting mixture was then transferred into a 0.5mm NMR tubes (7 inch) and ^{19}F NMR was taken every 6 min for a period of 5 h to allow the determination of the conversion.

3. Optimization of Reaction Conditions

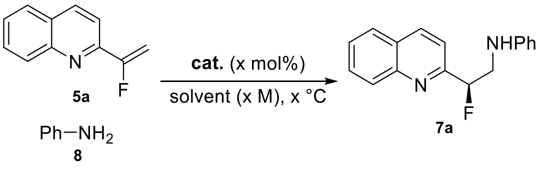

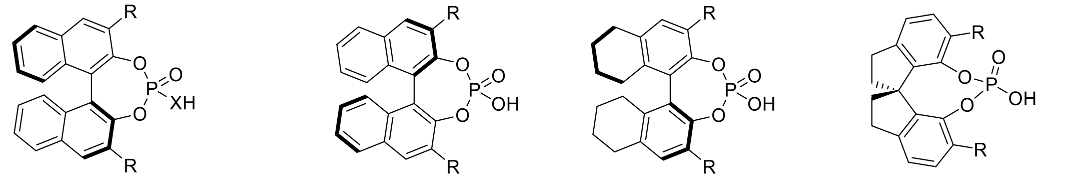

R = 2,4,6-triisopropylphenyl, X = O (**9a**)
R = 2,4,6-trimethylphenyl, X = O (**9b**)
R = Phenyl, X = O (**9c**)
R = 3,5-di-trifluoromethylphenyl, X = O (**9d**)
R = Iodine, X = O (**9e**)
R = 2,4,6-triisopropylphenyl, X = NHTf (**9f**)

R = triphenylsilyl (**9g**)
R = 9-anthryl (**9h**)

R = 2,4,6-triisopropylphenyl (**9i**)

R = 2,4,6-triisopropylphenyl (**9j**)

Entry	Solvent	Concentration (M)	Time (h)	Catalyst	Cat. loading (mol%)	Temp. (°C)	Aniline (equiv)	% conv.[a] (e.r.)
1	THF	0.5	48	9a	---	−20	3	0 (---)
2	THF	0.5	48	9a	20	−20	3	40 (96:4)
3	THF	0.5	96	9a	20	−20	3	68 (97:3)
4	THF	0.25	48	9a	20	−20	3	28 (97:3)
5	THF	0.1	48	9a	20	−20	3	15 (98:2)
6	THF	0.5	48	9a	20	−20	5	48 (95:5)
7	THF	0.5	48	9a	20	−20	10	58 (95:5)
8	THF	0.5	48	9a	10	−20	3	37 (95:5)
9	THF	0.5	48	9b	20	−20	3	25 (88:12)
10	THF	0.5	48	9c	20	−20	3	11 (58:42)
11	THF	0.5	48	9d	20	−20	3	n.d. (---)
12	THF	0.5	48	9e	20	−20	3	6 (55:45)
13	THF	0.5	48	9f	20	−20	3	n.d. (---)
14	THF	0.5	48	9g	20	−20	3	10 (62:38)
15	THF	0.5	48	9h	20	−20	3	25 (25:75)
16	THF	0.5	48	9i	20	−20	3	28 (89:11)
17	THF	0.5	48	9j	20	−20	3	n.d. (---)
18	THF	0.5	48	9a	20	−10	3	68 (96:4)
**19**	**THF**	**0.5**	**72**	9a	**20**	**−10**	**3**	**82[b] (96:4)**
**20**	**CPME**	**0.5**	**72**	9a	**20**	**−10**	**3**	**89[b] (95:5)**
21	hexane	0.5	72	9a	20	−10	3	98 (71:29)
22	toluene	0.5	72	9a	20	−10	3	95 (89:11)
23	Et_2O	0.5	72	9a	20	−10	3	94 (94:6)
24	CH_2Cl_2	0.5	72	9a	20	−10	3	70 (75:25)
25	CPME	0.5	120	9a	20	−20	3	81 (95:5)
26	CPME	0.5	120	9a	10	−20	3	68 (93:7)

[a] Determined by HPLC analysis with internal standard; [b] isolated yield.

4. Catalyst ee *vs.* Product ee

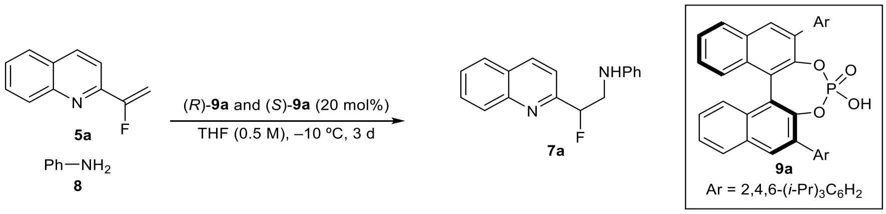

A series of mixtures of (*R*)-TRIP and (*S*)-TRIP were made in varying ratios. Reactions were carried out following General Procedure D, the enantioenrichment of each product was determined by HPLC analysis (Chiralpak IA, hexane/*i*-PrOH = 90/10, flow rate 0.5 mL/min, λ= 250 nm), t_r (major) = 23.4 min, t_r (minor) = 25.6 min. R^2 suggests no non-linear effects in these initial experiments.

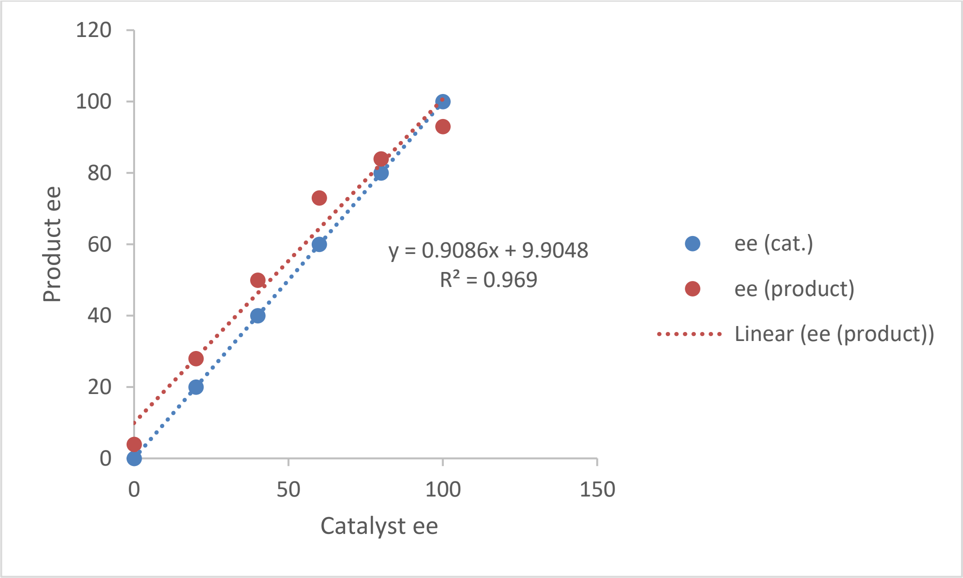

5. Mechanistic Study
5.1 DFT Calculations

Conformation Generation

The starting geometry for the catalyst was adapted from the crystal structure of pyridinium complex in the crystal structure with Cambridge database code CAZKEU.[3] It was assumed that the NH-O=P interaction present in this and all comparable crystal structures was retained and conformations of key structures were generated manually and where they yielded an optimized structure they are given below.

To study the conformational space of the bound complexes, we considered complexes with the catalyst bearing a proton on either of the two protruding phosphate oxygens (distal and proximal) and assumed a hydrogen bond with the quinoline nitrogen to be a critical interaction. Given this constraint, a full conformational exploration of both enantiomers of the product complex was undertaken. Two orientations of the quinoline ring were considered: one in which a secondary interaction between the the distal phosphate oxygen and the CH *peri* to the quinoline nitrogen was in place and one in which a 180° rotation about the $POH\cdots N_{quinoline}$ hydrogen bond takes place. All rotamers of the $CHFCH_2NHPh$ sidechain that could be sterically accommodated by the catalyst were attempted and *i*-Pr groups at ortho positions in the catalyst were retained in their preferred conformation while the *i*-Pr at the para position was adjusted to minimise clashing with the bound species (the geom=nocrowd option in Gaussian 09[4] was used in order to permit the relaxation of species featuring a small amount of steric clashing).

Calculations involving complexes containing chiral phosphoric acids

All frequency and geometry calculations were carried out using hybrid QM/MM calculations using the ONIOM method with B3LYP functional with the 6-31G** basis set for all atoms arising from aniline or quinoline and the PO_4H from the catalyst, and UFF is applied to the remainder of the catalyst. Additional single-point energy calculations were carried out on the previously optimised geometries using the M06-2X functional with the 6-31G** basis set employing a PCM solvation in diethyl ether and using an ultrafine grid. All calculations using the B3LYP, UFF or M06-2X functional have been performed using Gaussian 09.[4]

Calculations involving complexes not containing chiral phosphoric acids

All frequency and geometry calculations were carried out using the B3LYP functional with the 6-31G** basis set. Additional single-point energy calculations were carried out on the previously optimised geometries using the M06-2X functional with the 6-31G** basis set employing a PCM solvation in diethyl ether and using an ultrafine grid. All calculations using the B3LYP or M06-2X functional have been performed using Gaussian 09.[4]

Free energy calculations

Free energies are calculated from the generated electronic energies using the GoodVibes program (version 2.0.3)[5] with a standard state of 1M concentration and temperature of 253K.

Transition State Generation

All of the optimized complexes that were within 3 kcal mol^{-1} of the complex with the lowest energy were then subjected to a scan in which the proton at the stereocentre was moved towards either, the aniline nitrogen, the nearest (proximal) O=P or the furthest (distal) O=P. Each of these scans were then

used to initiate transition state optimizations. Subsequently, the lowest energy transition state structure was the starting point for a limited exploration of each of the steps that occurs prior to the stereoselectivity-determining step.

5.1.1 Reaction Profile – 5a

Free energy profile comparison (ONIOM (B3LYP/6-31G**:UFF), [single-point energy M06-2X/6-31G**(+PCM for Et_2O)]).

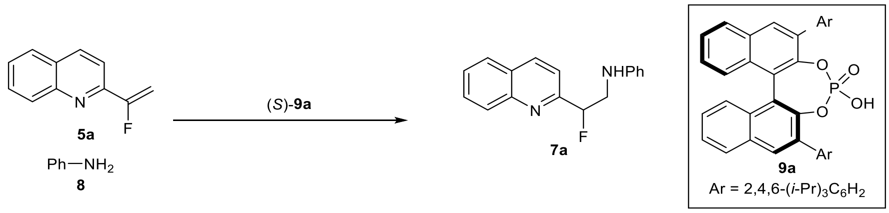

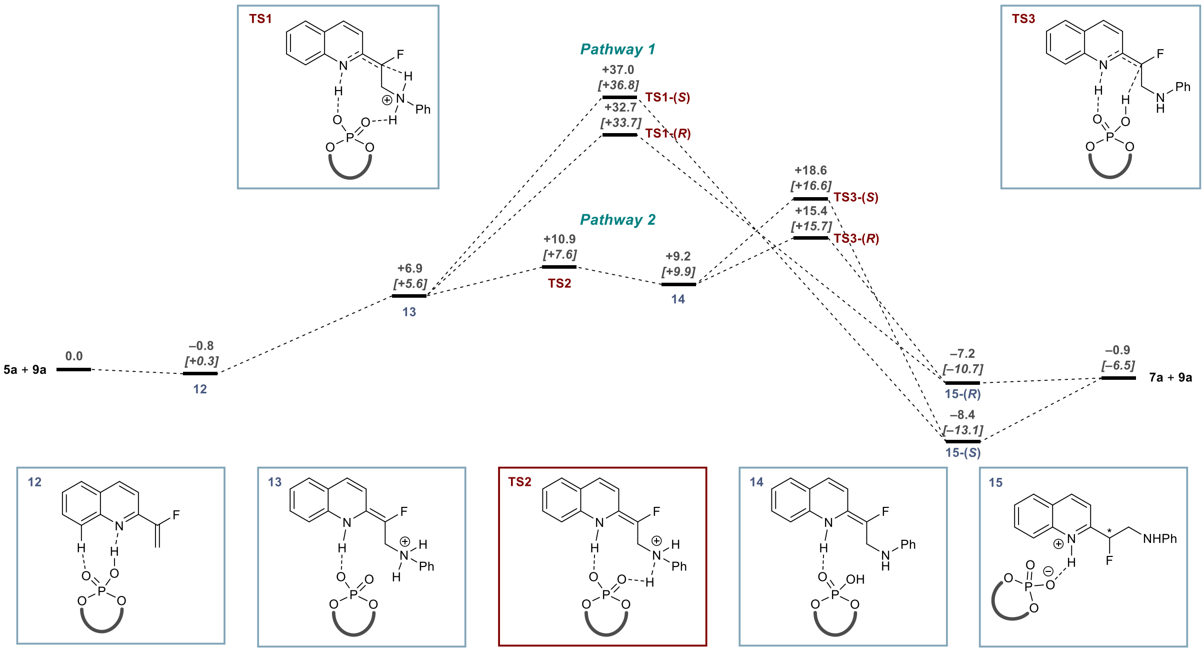

5.1.2 Reaction Profile – S1 (Phenyl System)

The reaction of compound **S1** studied previously was revisited with the insights provided by the calculations described in the main text.

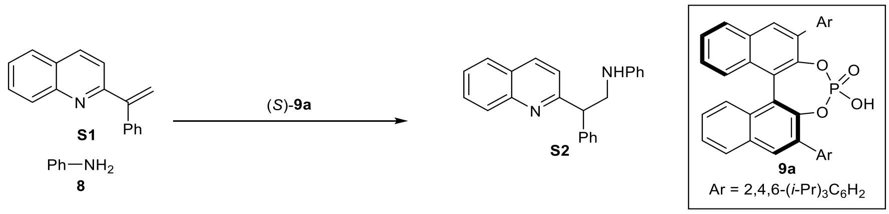

Free energy profile comparison (ONIOM (B3LYP/6-31G**:UFF), [single-point energy M06-2X/6-31G**(+PCM for Et_2O)]).

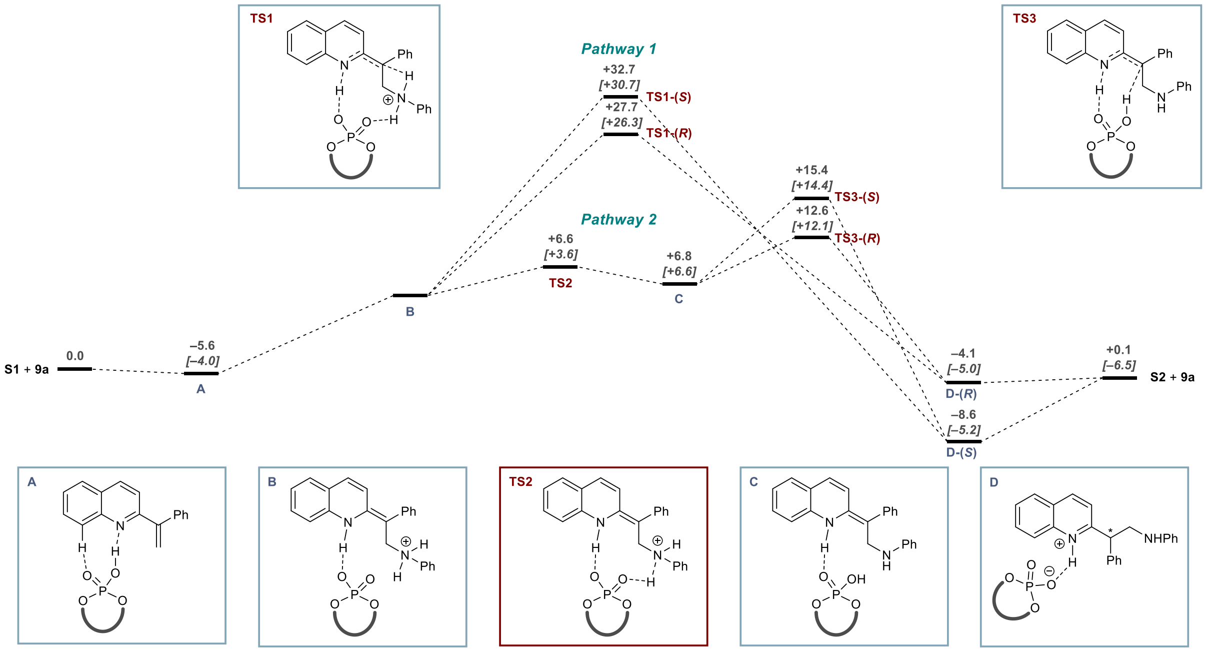

5.1.2 Reaction Profile – RDS Energy profile

A series of calculations, demonstrating the difference in energy for the RDS of different substrates. Free energy profile comparison (ONIOM (B3LYP/6-31G**:UFF), [single-point energy M06-2X/6-31G**(+PCM for Et_2O)]).

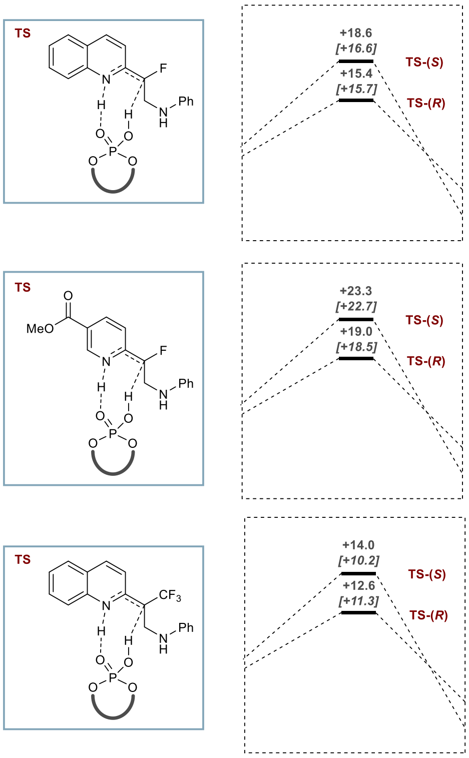

5.1.5 Background Reaction Profile – 5a & 10

A series of calculations, demonstrating the high energy barrier RDS for the uncatalyzed reaction, and decrease in the vinyl CF_3 system. Free energy profile comparison (ONIOM (B3LYP/6-31G**:UFF), [single-point energy M06-2X/6-31G**(+PCM for Et_2O)]).

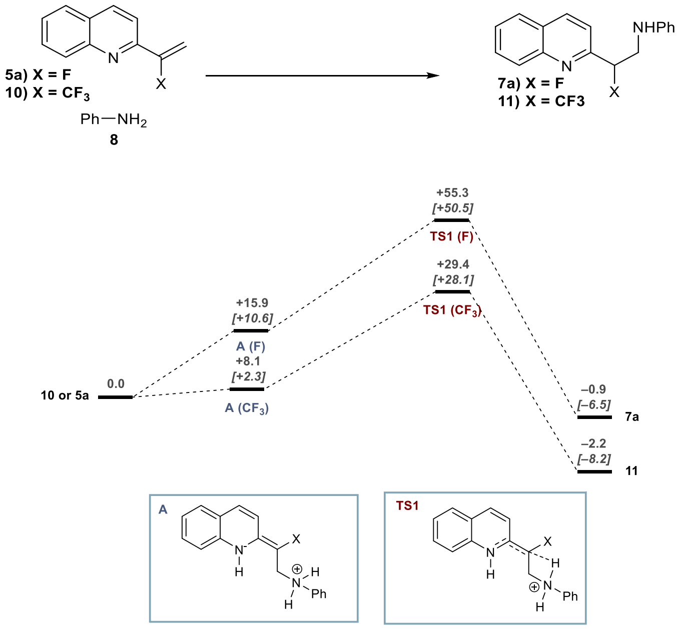

5.2 HRMS Study

A series of reactions were analyzed by ESI analysis. Results suggest the product **7a** has the largest affinity towards CPA **9a** as compared to aniline **8** and quinoline **5a**.

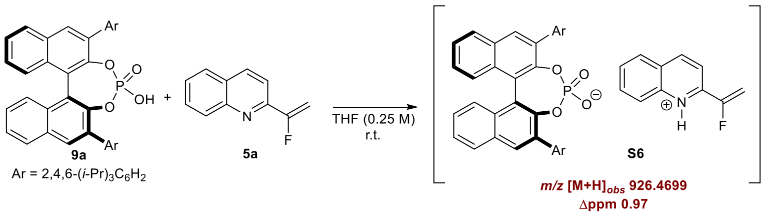

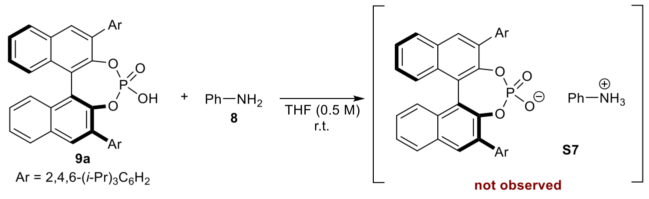

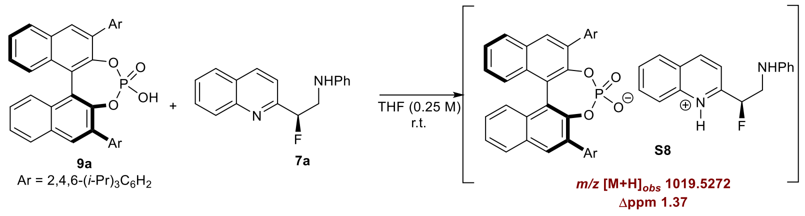

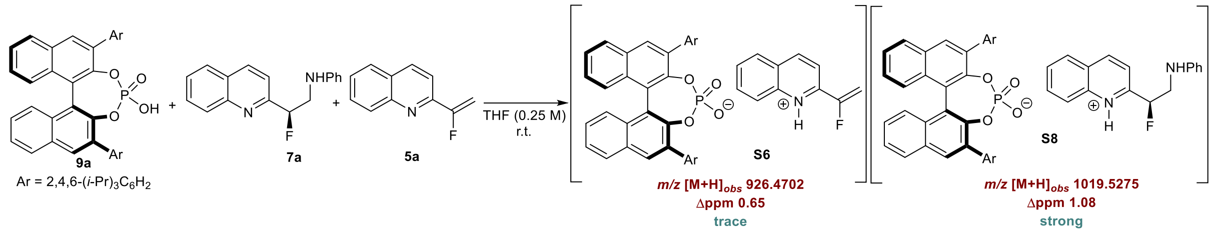

5.3 Kinetic Isotope Effect Study

5.3.1 Kinetic Isotope Effect of Aniline

Data was obtained according to General Procedure E using aniline.

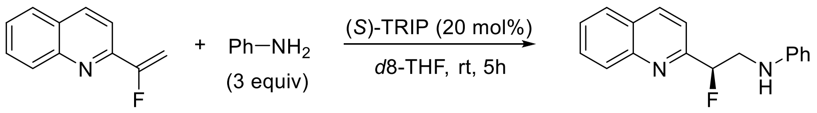

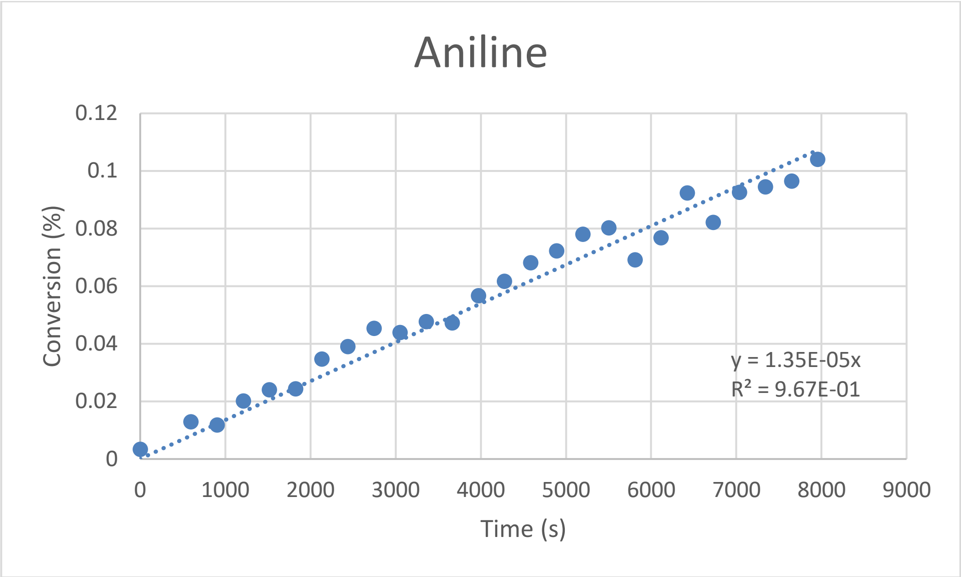

5.3.2 Kinetic Isotope Effect of *D*₂-Aniline

Data was obtained according to General Procedure E using D_2-aniline for 10 h.

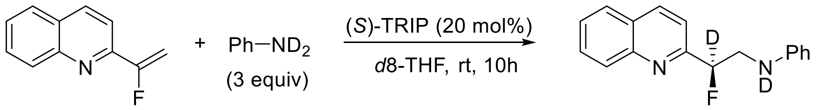

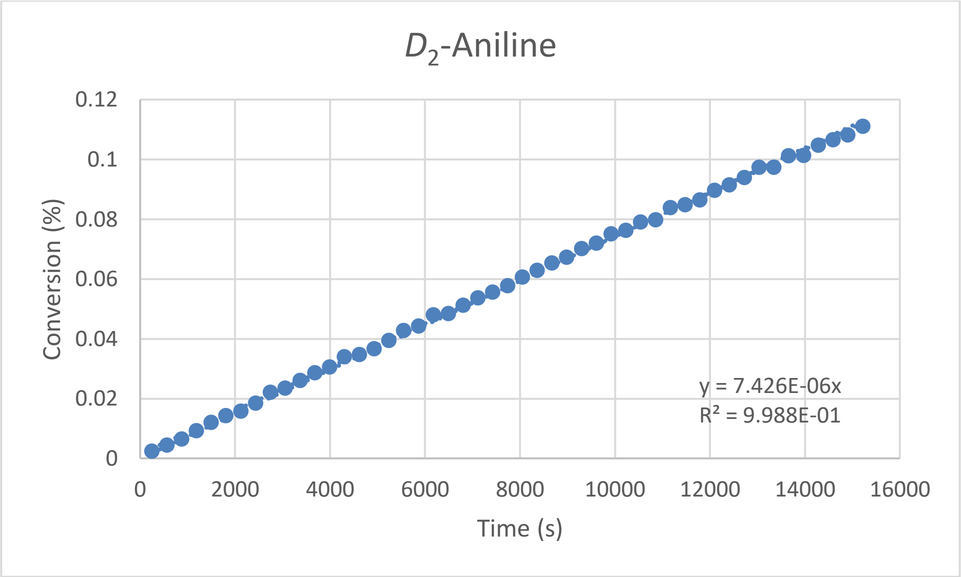

5.3.3 Kinetic Isotope Effect of ^{15}N-Aniline

Data was obtained according to General Procedure E using ^{15}N-aniline.

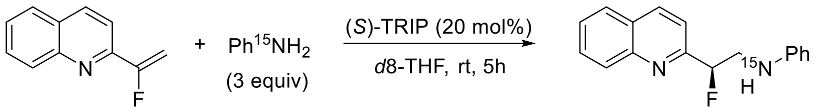

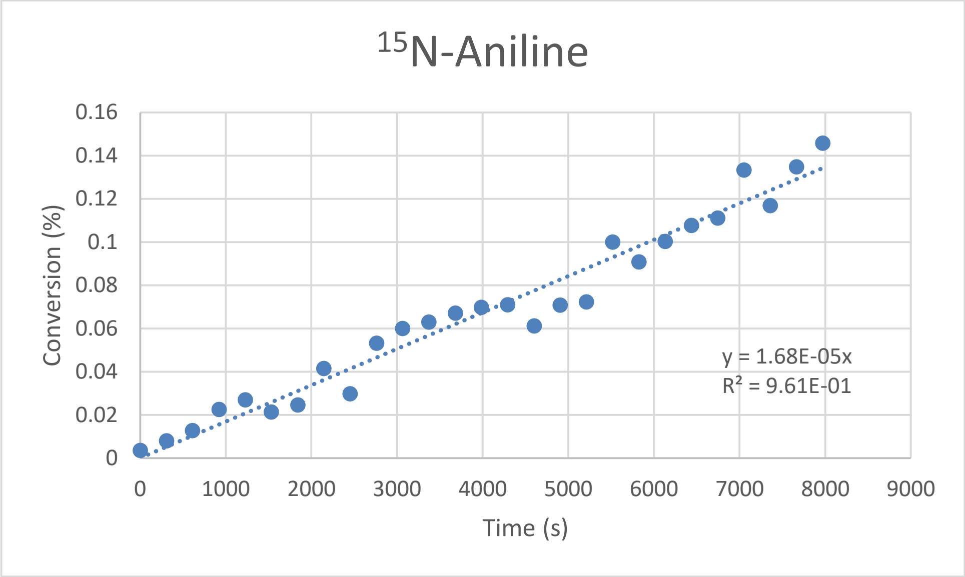

6. Ramachandran Plots

Ramachandran plots were computed at the M06-2X/6-31+G** level of theory. Calculations were performed in Gaussian09 using the modified redundant coordinate facility with dihedral angles being moved in 15° increments. Energy in kcal mol^{-1}.

6.1 Ramachandran Plot for Compound 9a

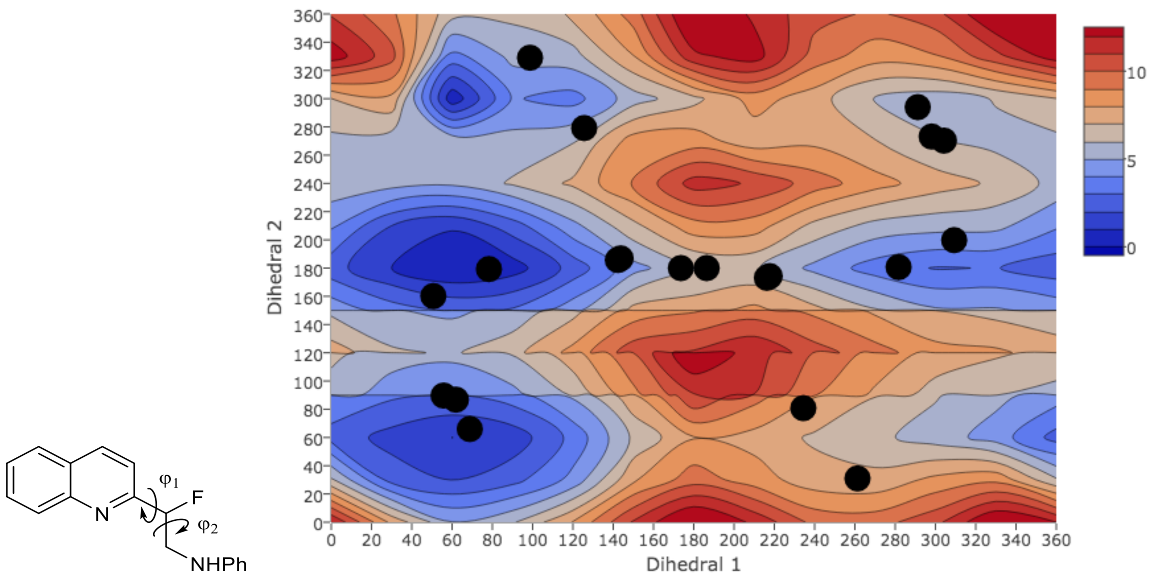

6.2 Ramachandran Plot for Compound 16

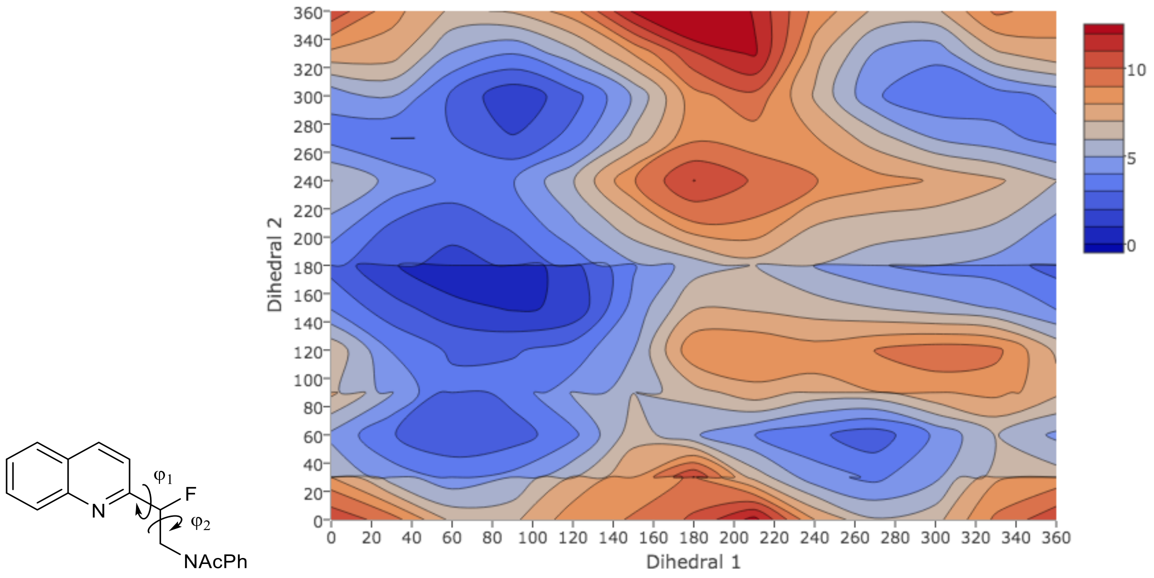

6.3 Ramachandran Plot for *N*-(2-(Quinolin-2-yl)ethyl)aniline

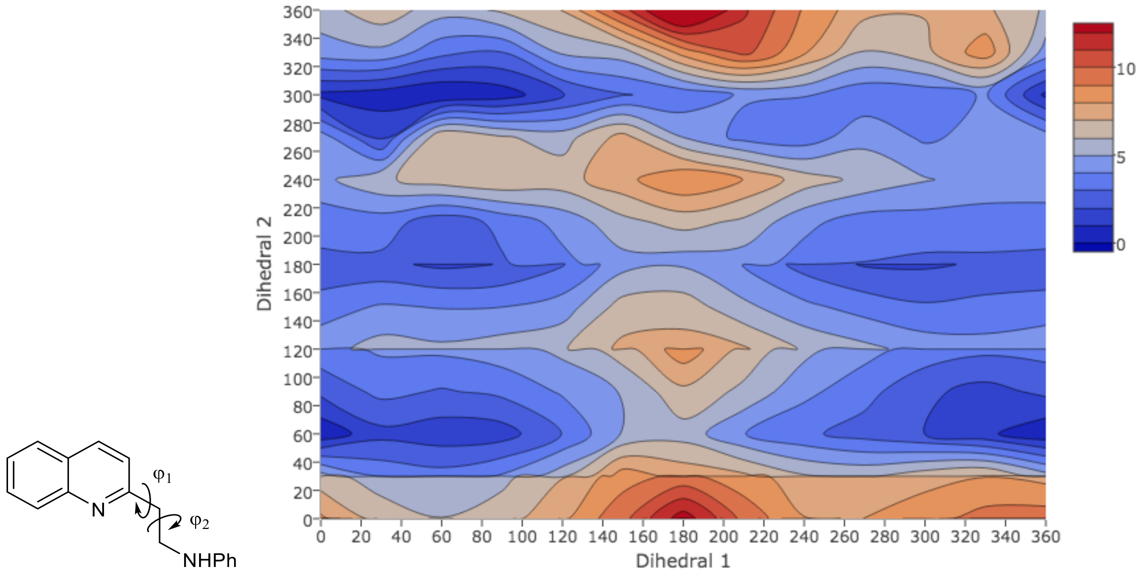

7. Characterization Data for Compounds

2-Bromo-3-methylquinoline **S3**

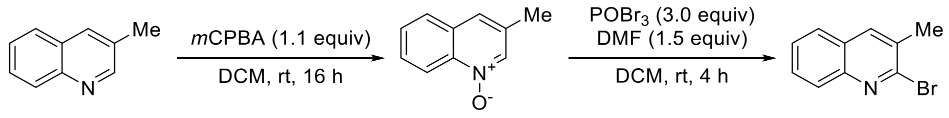

Prepared according to literature procedures.[6,7]

An oven-dried, 250 mL round bottomed flask was charged with 3-methylquinoline (1.34 mL, 10.0 mmol, 1.0 equiv). The vessel was sealed and purged with N_2. CH_2Cl_2 (50 mL, 0.2 M) was added to the vessel and the mixture was stirred at 0 °C for 15 min before the addition of *m*CPBA (1.91 g, 11.0 mmol, 1.1 equiv). The reaction was left to stir at rt for 16 h. The mixture was then diluted with CH_2Cl_2 (50 mL), and washed with aq. KOH 6 M solution (3 × 50 mL). The organic layer was collected, dried over Na_2SO_4, filtered, and concentrated *in vacuo*.

NB. Calculations based upon quantitative conversion to 2-bromo-3-methylquinoline 1-oxide in the previous step. An oven-dried, 250 mL round bottomed flask was charged with the crude 3-methylquinoline *N*-oxide (10.0 mmol, 1.0 equiv) and $POBr_3$ (8.60 g, 30.0 mmol, 3.0 equiv). The vessel was sealed and purged with N_2. Dry CH_2Cl_2 (50 mL, 0.2 M) and dry DMF (1.16 mL, 1.50 mmol, 1.5 equiv) were added. The reaction was left to stir at rt for 4 h. Sat. aq, Na_2SO_4 solution was added slowly until the pH was 7-8. The layers in the resulting mixture were separated and the aqueous was extracted with CH_2Cl_2 (3 × 50 mL). The organic phases were combined, dried over Na_2SO_4, filtered, and concentrated *in vacuo*. Purification of the crude product *via* flash chromatography (silica gel, EtOAc:petroleum ether, 1:99) gave the desired product as a white solid (506 mg, 23%).

^{1}H NMR (500 MHz, CDCl$_3$): δ 8.00 (d, *J* = 8.5 Hz, 1H), 7.90 (s, 1H), 7.71 (d, *J* = 8.1 Hz, 1H), 7.68 – 7.62 (m, 1H), 7.58 – 7.47 (m, 1H), 2.52 (d, *J* = 1.1 Hz, 3H).

^{13}C NMR (126 MHz, CDCl$_3$): δ 147.1, 145.6, 137.1, 132.4, 129.6, 128.4, 127.8, 127.2, 127.0, 22.5.

Spectroscopic data in agreement with literature values.[6]

2-Bromo-6-fluoroquinoline **S4**

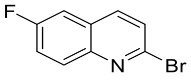

Prepared according to a literature procedure.[6]

An oven-dried, 10 mL microwave vial was charged with 6-fluoroquinolin-2(1*H*)-one (226 mg, 1.00 mmol, 1.0 equiv) and $POBr_3$ (56.2 mg, 1.10 mmol, 1.1 equiv). The vessel was capped and purged with N_2. The mixture was heated to 140 °C for 3 h, then cooled to rt and poured over ice. The resulting precipitate was collected, washed with petroleum ether and dried *in vacuo* to give the desired product as a pale brown solid (114 mg, 50%).

^{1}H NMR (400 MHz, CDCl$_3$): δ 8.04 (dd, *J* = 9.2, 5.2 Hz, 1H), 7.96 (d, *J* = 8.6 Hz, 1H), 7.54 (d, *J* = 8.6 Hz, 1H), 7.50 (td, *J* = 8.8, 2.8 Hz, 1H), 7.43 (dd, *J* = 8.6, 2.8 Hz, 1H).

^{13}C NMR (126 MHz, CDCl$_3$): δ 160.7 (d, ^1J = 249.5 Hz), 145.7, 141.1, 137.8 (d, ^4J = 5.2 Hz), 131.3 (d, ^3J = 9.3 Hz), 127.8 (d, ^3J = 10.3 Hz), 126.7, 120.9 (d, ^2J = 25.5 Hz), 111.2 (d, ^2J = 22.2 Hz).

^{19}F NMR (376 MHz, $CDCl_3$): δ −111.94.

Spectroscopic data in agreement with literature values.[7]

2,6-Dibromoquinoline **S5**

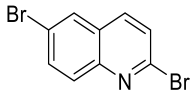

Prepared according to a literature procedure.[6]

An oven-dried, 10 mL microwave vial was charged with 6-bromoquinolin-2(1*H*)-one (251 mg, 1.13 mmol, 1.13 equiv) and $POBr_3$ (287 mg, 1.00 mmol, 1.0 equiv). The vessel was capped and purged with N_2. The mixture was heated to 140 °C for 3 h, then cooled to rt and poured over ice. The resulting precipitate was collected, washed with petroleum ether and dried *in vacuo* to give the desired product as a brown solid (178 mg, 62%).

^{1}H NMR (500 MHz, $CDCl_3$): δ 7.98 (d, J = 2.2 Hz, 1H), 7.91 (dd, J = 8.7, 2.0 Hz, 2H), 7.80 (dd, J = 8.9, 2.2 Hz, 1H), 7.55 (d, J = 8.6 Hz, 1H).

^{13}C NMR (126 MHz, $CDCl_3$): δ 147.3, 142.4, 137.4, 134.2, 130.5, 129.9, 128.2, 126.9, 121.2.

Spectroscopic data in agreement with literature values.[6]

2-(1-Fluorovinyl)quinoline **5a**

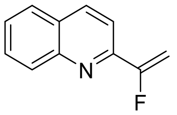

Prepared according to General Procedure C using 2-bromoquinoline (207 mg, 1.00 mmol, 1.0 equiv), (1-fluorovinyl)(methyl)diphenylsilane (356 mg, 1.50 mmol, 1.5 equiv), $Pd(PPh_3)_4$ (59.0 mg, 0.05 mmol, 5 mol%), CuI (9.7 mg, 0.05 mmol, 5 mol%), CsF (364 mg, 2.35 mmol, 2.35 equiv), and DMF (10 mL, 0.1 M). The reaction mixture was subjected to the purification outlined in the General Procedure C (silica gel, Et_2O:petroleum ether, 1:99) to afford the desired product as a pale yellow oil (161 mg, 93%).

υ_{max} (film): 3056, 2926, 1654, 1284, 1108, 834, 758 cm^{-1}.

^{1}H NMR (400 MHz, $CDCl_3$): δ 8.13 (d, J = 8.6 Hz, 1H), 8.10 (d, J = 8.5 Hz, 1H), 7.81 (dd, J = 8.1, 0.9 Hz, 1H), 7.73 (ddd, J = 8.4, 6.9, 1.4 Hz, 1H), 7.69 (dd, J = 8.6, 1.2 Hz, 1H), 7.54 (ddd, J = 8.1, 6.9, 1.1 Hz, 1H), 5.87 (dd, $^3J_{HF}$ = 48.6 Hz, $^2J_{HH}$ = 2.9 Hz, 1H), 5.14 (dd, $^3J_{HF}$ = 16.4 Hz, $^2J_{HH}$ = 2.9 Hz, 1H).

^{13}C NMR (126 MHz, $CDCl_3$): δ 162.3 (d, ^1J = 251.2 Hz), 150.1 (d, ^2J = 35.4 Hz), 147.9 (d, ^4J = 3.8 Hz), 136.9, 130.0, 129.8, 128.0, 127.6, 127.1, 116.8 (d, ^3J = 4.7 Hz), 93.8 (d, ^2J = 18.4 Hz).

^{19}F NMR (376 MHz, $CDCl_3$): δ −114.28.

HRMS (ESI): exact mass calculated for $[M+H]^+$ ($C_{11}H_9NF$) requires *m/z* 174.0714, found *m/z* 174.0712.

2-(1-Fluorovinyl)-3-methylquinoline **5b**

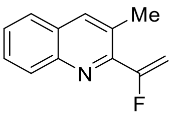

Prepared according to General Procedure C using 2-bromo-3-methylquinoline (99.5 mg, 0.45 mmol, 1.0 equiv), (1-fluorovinyl)(methyl)diphenylsilane (164 mg, 0.675 mmol, 1.5 equiv), $Pd(PPh_3)_4$ (520 mg, 0.45 mmol, 1.0 equiv), CuI (85.7 mg, 0.45 mmol, 1.0 equiv), CsF (161 mg, 1.06 mmol, 2.35 equiv), and DMF (4.5 mL, 0.1 M). The reaction mixture was subjected to the purification outlined in General Procedure C (silica gel, Et_2O:petroleum ether, 1:99) to afford the desired product as a pale yellow oil (75.4 mg, 90%).

υ_{max} (film): 2361, 2342, 1655, 1493, 1447, 1327, 1273, 1184, 1138, 1065, 930, 903, 860, 791, 756, 729 cm^{-1}.

^{1}H NMR (500 MHz, $CDCl_3$): δ 8.08 (dd, J = 8.4, 1.3 Hz, 1H), 7.97 (s, 1H), 7.75 (dd, J = 8.2, 1.5 Hz, 1H), 7.67 (ddd, J = 8.4, 6.8, 1.5 Hz, 1H), 7.53 (ddd, J = 8.1, 6.8, 1.2 Hz, 1H), 5.37 (dd, $^3J_{HF}$ = 48.2 Hz, $^2J_{HH}$ = 3.0 Hz, 1H), 5.19 (dd, $^3J_{HF}$ = 16.8 Hz, $^2J_{HH}$ = 3.0 Hz, 1H), 2.60 (d, J = 4.9 Hz, 3H).

^{13}C NMR (126 MHz, $CDCl_3$): δ 163.9 (d, ^1J = 256.3 Hz), 151.2 (d, ^2J = 32.3 Hz), 146.2, 146.2, 137.6, 129.6, 129.2, 128.4, 127.5, 126.8, 96.3 (d, ^2J = 19.2 Hz), 20.0 (d, ^4J = 7.6 Hz).

^{19}F NMR (470 MHz, $CDCl_3$): δ −99.90.

HRMS (ESI): exact mass calculated for $[M+H]^+$ ($C_{12}H_{11}FN$) requires *m/z* 188.0870, found *m/z* 188.0866.

6-Fluoro-2-(1-fluorovinyl)quinoline **5c**

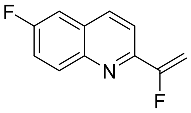

Prepared according to General Procedure C using 6-fluoro-2-bromoquinoline (150 mg, 0.664 mmol, 1.0 equiv), (1-fluorovinyl)(methyl)diphenylsilane (241 mg, 0.995 mmol, 1.5 equiv), $Pd(PPh_3)_4$ (38.3 mg, 0.033 mmol, 5 mol%), CuI (6.3 mg, 0.033 mmol, 5 mol%), CsF (237 mg, 1.56 mmol, 2.35 equiv), and DMF (6.6 mL, 0.1 M). The reaction mixture was subjected to the purification outlined in General Procedure C (silica gel, Et_2O:petroleum ether, 1:99) to afford the desired product as a pale yellow oil (84.0 mg, 66%).

υ_{max} (film): 1657, 1626, 1603, 1558, 1501, 1481, 1379, 1304, 1285, 1244, 1221, 1144, 1101, 916, 866, 829 cm^{-1}.

^{1}H NMR (400 MHz, $CDCl_3$): δ 8.12 (d, J = 8.7 Hz, 1H), 8.08 (dd, J = 9.3, 5.3 Hz, 1H), 7.68 (d, J = 8.7 Hz, 1H), 7.49 (td, J = 8.9, 2.8 Hz, 1H), 7.40 (dd, J = 8.7, 2.9 Hz, 1H), 5.84 (dd, $^3J_{HF}$ = 48.6 Hz, $^2J_{HH}$ = 3.0 Hz, 1H), 5.13, (dd, $^3J_{HF}$ = 16.4 Hz, $^2J_{HH}$ = 3.1 Hz, 1H).

^{13}C NMR (126 MHz, CDCl$_3$): δ 162.0 (d, ^1J = 251.0 Hz), 160.8 (d, ^1J = 249.4 Hz), 149.6, (dd, ^2J = 35.8, 3.1 Hz), 145.0 (d, ^4J = 4.0 Hz), 136.4 (d, ^3J = 6.6 Hz), 132.3 (d, ^3J = 9.1 Hz), 128.6 (d, ^3J = 10.1 Hz), 120.5 (d, ^2J = 26.0 Hz), 117.6 (d, ^4J = 4.7 Hz), 110.6 (d, ^2J = 21.8 Hz), 93.8 (d, ^2J = 18.5 Hz).

^{19}F NMR (376 MHz, CDCl$_3$): δ −112.23, −114.42.

HRMS (ESI): exact mass calculated for $[M+H]^+$ ($C_{11}H_8F_2N$) requires *m/z* 192.0619, found *m/z* 192.0618.

6-Bromo-2-(1-fluorovinyl)quinoline **5d**

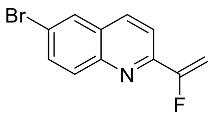

Prepared according to General Procedure C using 2,6-dibromoquinoline (259 mg, 0.90 mmol, 1.0 equiv), (1-fluorovinyl)(methyl)diphenylsilane (328 mg, 1.38 mmol, 1.5 equiv), Pd(PPh$_3$)$_4$ (52.0 mg, 0.045 mmol, 5 mol%), CuI (8.6 mg, 0.045 mmol, 5 mol%), CsF (322 mg, 2.12 mmol, 2.35 equiv), and DMF (9 mL, 0.1 M). The reaction mixture was subjected to the purification outlined in General Procedure C (silica gel, Et$_2$O:petroleum ether, 1:99) to afford the desired product as a pale yellow solid (183 mg, 81%).

υ_{max} (film): 1659, 1595, 1489, 1283, 1190, 1107, 920, 874, 827 cm^{-1}.

^{1}H NMR (400 MHz, CDCl$_3$): δ 8.11 (d, J = 8.6 Hz, 1H), 7.97 (d, J = 2.2 Hz, 1H), 7.95 (d, J = 9.0 Hz, 1H), 7.78 (dd, J = 9.0, 2.2 Hz, 1H), 7.70 (dd, J = 8.7, 1.3 Hz, 1H), 5.87 (dd, $^3J_{HF}$ = 48.5 Hz, $^2J_{HH}$ = 3.1 Hz, 1H), 5.16 (dd, $^3J_{HF}$ = 16.3 Hz, $^2J_{HH}$ = 3.1 Hz, 1H).

^{13}C NMR (126 MHz, CDCl$_3$): δ 161.9 (d, ^1J = 251.1 Hz), 150.5 (d, ^2J = 35.6 Hz), 146.5 (d, ^4J = 4.0 Hz), 136.1, 133.7, 131.5, 129.7, 129.1, 121.2, 117.8 (d, ^3J = 4.6 Hz), 94.4 (d, ^2J = 18.3 Hz).

^{19}F NMR (376 MHz, CDCl$_3$): δ −114.60.

HRMS (ESI): exact mass calculated for $[M+H]^+$ ($C_{11}H_8BrFN$) requires *m/z* 251.9819, found *m/z* 251.9816.

4-Bromo-2-(1-fluorovinyl)quinoline **5e**

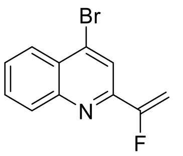

Prepared according to General Procedure C using 2,4-dibromoquinoline (259 mg, 0.90 mmol, 1.0 equiv), (1-fluorovinyl)(methyl)diphenylsilane (328 mg, 1.35 mmol, 1.5 equiv), Pd(PPh$_3$)$_4$ (52.0 mg, 0.045 mmol, 5 mol%), CuI (8.6 mg, 0.045 mmol, 5 mol%), CsF (322mg, 2.12 mmol, 2.35 equiv), and DMF (9 mL, 0.1 M). The reaction mixture was subjected to the purification outlined in General Procedure C (silica gel, Et$_2$O:petroleum ether, 1:99) to afford the desired product as a pale yellow solid (105 mg, 42%).

υ_{max} (film): 2357, 1578, 1547, 1491, 1404, 1339, 1265, 1251, 1148, 1105, 928 cm^{-1}.

^{1}H NMR (500 MHz, CDCl$_3$): δ 8.12 (dd, J = 8.5, 1.4 Hz, 1H), 8.05 (dd, J = 8.5, 1.2 Hz, 1H), 7.94 (d, J = 1.2 Hz, 1H), 7.74 (ddd, J = 8.4, 6.8, 1.4 Hz, 1H), 7.60 (ddd, J = 8.2, 6.8, 1.2 Hz, 1H), 5.89 (dd, $^3J_{HF}$ = 48.4 Hz, $^2J_{HH}$ = 3.1 Hz, 1H), 5.16 (dd, $^3J_{HF}$ = 16.2 Hz, $^2J_{HH}$ = 3.1 Hz, 1H).

^{13}C NMR (126 MHz, CDCl$_3$): δ 161.0 (d, ^1J = 251.5 Hz), 149.8 (d, ^2J = 36.1 Hz), 148.3 (d, ^4J = 4.0 Hz), 135.0 (d, ^4J = 1.7 Hz), 130.9, 130.2, 128.3, 127.5, 126.7, 120.9 (d, ^3J = 5.1 Hz), 94.7 (d, ^2J = 17.9 Hz).

^{19}F NMR (470 MHz, CDCl$_3$): δ −114.71.

HRMS (ESI): exact mass calculated for $[M+H]^+$ ($C_{11}H_8BrFN$) requires *m/z* 251.9819, found *m/z* 251.9817.

2-(1-Fluorovinyl)quinoxaline **5f**

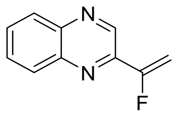

Prepared according to General Procedure C using 2-bromoquinoxaline (174 mg, 1.00 mmol, 1.0 equiv), (1-fluorovinyl)(methyl)diphenylsilane (363 mg, 1.50 mmol, 1.5 equiv), Pd(PPh$_3$)$_4$ (57.8 mg, 0.05 mmol, 5 mol%), CuI (6.6 mg, 0.05 mmol, 5 mol%), CsF (448 mg, 2.35 mmol, 2.35 equiv), and DMF (10 mL, 0.1 M). The reaction mixture was subjected to the purification outlined in General Procedure C (silica gel, Et$_2$O:petroleum ether, 1:99) to afford the desired product as a pale yellow solid (158 mg, 91%).

υ_{max} (film): 1655, 1506, 1489, 1364, 1341, 1105, 968, 924, 874, 764, 743 cm^{-1}.

^{1}H NMR (400 MHz, CDCl$_3$): δ 9.13 (s, 1H), 8.16 – 8.07 (m, 2H), 7.87 – 7.74 (m, 2H), 5.92 (dd, $^3J_{HF}$ = 48.2 Hz, $^2J_{HH}$ = 3.4 Hz, 1H), 5.27 (dd, $^3J_{HF}$ = 16.5 Hz, $^2J_{HH}$ = 3.4 Hz, 1H).

^{13}C NMR (126 MHz, CDCl$_3$): δ 160.7 (d, ^1J = 250.8 Hz), 145.0 (d, ^2J = 34.0 Hz), 142.6, 141.9, 141.3 (d, ^3J = 5.3 Hz), 130.9, 130.7, 129.8, 129.4, 95.9 (d, ^2J = 17.3 Hz).

^{19}F NMR (376 MHz, CDCl$_3$): δ −116.24.

HRMS (ESI): exact mass calculated for $[M+H]^+$ ($C_{10}H_8FN_2$) requires *m/z* 175.0666, found *m/z* 175.0665.

2-(1-Fluorovinyl)benzo[*d*]thiazole **5g**

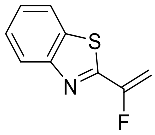

Prepared according to General Procedure C using 2-Bromobenzothiazole (214 mg, 1.00 mmol, 1.0 equiv), (1-fluorovinyl)(methyl)diphenylsilane (356 mg, 1.50 mmol, 1.5 equiv), Pd(PPh$_3$)$_4$ (1.16 g, 1.00 mmol, 1.0 equiv), CuI (132 mg, 1.00 mmol, 1.0 equiv), CsF (364 mg, 2.40 mmol, 2.4 equiv), and DMF (10 mL, 0.1 M). The reaction mixture was subjected to the purification outlined in General Procedure C (silica gel, Et$_2$O:petroleum ether, 1:99) to afford the desired product as a pale yellow solid (73.8 mg, 42%).

υ_{max} (film): 2955, 2926, 2853, 1647, 1558, 1435, 1314, 1290, 1261, 1092, 760, 729, 700 cm^{-1}.

^{1}H NMR (500 MHz, $CDCl_3$): δ 8.08 (d, J = 8.2 Hz, 1H), 7.92 (d, J = 8.0 Hz, 1H), 7.64 – 7.36 (m, 3H), 5.74 (dd, $^{3}J_{HF}$ = 47.1 Hz, $^{2}J_{HH}$ = 3.7 Hz, 1H), 5.21 (dd, $^{3}J_{HF}$ = 15.5 Hz, $^{2}J_{HH}$ = 3.8 Hz, 1H).

^{13}C NMR (126 MHz, $CDCl_3$): δ 159.9 (d, ^{2}J = 39.3 Hz), 157.3 (d, ^{1}J = 247.7 Hz), 153.5 (d, ^{4}J = 2.0 Hz), 135.0, 126.8, 126.1, 123.8, 121.9, 95.5 (d, ^{2}J = 17.2 Hz).

^{19}F NMR (470 MHz, $CDCl_3$): δ −106.57.

HRMS (ESI): exact mass calculated for $[M+H]^+$ (C_9H_7FNS) requires *m/z* 180.0278, found *m/z* 180.0277.

Methyl 6-(1-fluorovinyl)nicotinate **5h**

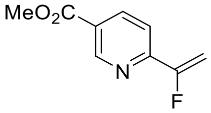

Prepared according to General Procedure C using 6-bromonicotinate (216 mg, 1.00 mmol, 1.0 equiv), (1-fluorovinyl)(methyl)diphenylsilane (356 mg, 1.50 mmol, 1.5 equiv), $Pd(PPh_3)_4$ (59.0 mg, 0.05 mmol, 5 mol%), CuI (9.7 mg, 0.05 mmol, 5 mol%), CsF (364 mg, 2.40 mmol, 2.4 equiv), and DMF (10 mL, 0.1 M). The reaction mixture was subjected to the purification outlined in General Procedure C (silica gel, Et_2O:petroleum ether, 1:99) to afford the desired product as a pale yellow solid (164 mg, 91%).

υ_{max} (film): 1722, 1655, 1593, 1560, 1437, 1366, 1287, 1273, 1196, 1130, 1101, 1022, 920, 897, 858, 789, 733 cm^{-1}.

^{1}H NMR (400 MHz, $CDCl_3$): δ 9.14 – 9.13 (m, 1H), 8.31 (dd, J = 8.2, 2.1 Hz, 1H), 7.59 (d, J = 7.9 Hz, 1H), 5.84 (dd, $^{3}J_{HF}$ = 48.5 Hz, $^{2}J_{HH}$ = 2.9 Hz, 1H), 5.11 (dd, $^{3}J_{HF}$ = 16.3 Hz, $^{2}J_{HH}$ = 2.9 Hz, 1H), 3.94 (s, 3H).

^{13}C NMR (126 MHz, $CDCl_3$): δ 165.4, 161.3 (d, ^{1}J = 249.3 Hz), 153.3 (d, ^{2}J = 36.8 Hz), 150.8 (d, ^{4}J = 4.5 Hz), 138.2 (d, ^{4}J = 1.8 Hz), 125.8, 118.2 (d, ^{3}J = 4.5 Hz), 95.2 (d, ^{2}J = 17.6 Hz), 52.6.

^{19}F NMR (376 MHz, $CDCl_3$): δ −115.94.

HRMS (ESI): exact mass calculated for $[M+H]^+$ ($C_9H_9FNO_2$) requires *m/z* 182.0612, found *m/z* 182.0610.

1-(1-Fluorovinyl)isoquinoline **5i**

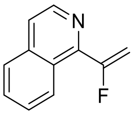

Prepared according to General Procedure C using 1-bromoisoquinoline (207 mg, 1.00 mmol, 1.0 equiv), (1-fluorovinyl)(methyl)diphenylsilane (356 mg, 1.50 mmol, 1.5 equiv), $Pd(PPh_3)_4$ (59.0 mg, 0.05 mmol, 5 mol%), CuI (9.7 mg, 0.05 mmol, 5 mol%), CsF (364 mg, 2.40 mmol, 2.4 equiv), and DMF (10 mL, 0.1 M). The reaction mixture was subjected to the purification outlined in General Procedure C (silica gel, Et_2O:petroleum ether, 1:99) to afford the desired product as a pale yellow oil (171 mg, 99%).

υ_{max} (film): 1657, 1622, 1584, 1557, 1393, 1298, 1250, 1215, 1175, 1153, 1140, 926, 858, 827, 748, 685, 579 cm^{-1}.

^{1}H NMR (400 MHz, $CDCl_3$): δ 8.53 (d, J = 5.6 Hz, 1H), 8.37 (d, J = 8.6 Hz, 1H), 7.83 (d, J = 8.2 Hz, 1H), 7.76 – 7.65 (m, 2H), 7.61 (ddd, J = 8.2, 6.9, 1.2 Hz, 1H), 5.50 – 5.19 (m, 2H).

^{13}C NMR (126 MHz, $CDCl_3$): δ 163.0 (d, ^1J = 256.4 Hz), 151.0 (d, ^2J = 31.1 Hz), 141.9, 136.9, 130.5, 128.0 (d, ^4J = 1.9 Hz), 127.2, 126.3 (d, ^3J = 9.7 Hz), 126.0 (d, ^4J = 2.3 Hz), 122.2, 97.3 (d, ^2J = 19.2 Hz).

^{19}F NMR (376 MHz, $CDCl_3$): δ −97.19.

HRMS (ESI): exact mass calculated for $[M+H]^+$ ($C_{11}H_9FN$) requires *m/z* 174.0714, found *m/z* 174.0710.

4-(1-Fluorovinyl)quinoline **5j**

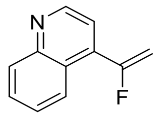

Prepared according to General Procedure C using 4-bromoquinoline (208 mg, 1.00 mmol, 1.0 equiv), (1-fluorovinyl)(methyl)diphenylsilane (356 mg, 1.50 mmol, 1.5 equiv), $Pd(PPh_3)_4$ (57.8 mg, 0.05 mmol, 5 mol%), CuI (9.7 mg, 0.05 mmol, 5 mol%), CsF (364 mg, 2.40 mmol, 2.4 equiv), and DMF (10 mL, 0.1 M). The reaction mixture was subjected to the purification outlined in General Procedure C (silica gel, Et_2O:petroleum ether, 1:99) to afford the desired product as a pale yellow oil (82.1 mg, 47%).

NB. Due to the reactive nature of this compound, an analytically pure sample was not obtained, data reported as observed. The compound was used directly in the next step.

υ_{max} (film): 2959, 2928, 2870, 1659, 1578, 1558, 1508, 1493, 1462, 1362, 1315, 1282, 1258, 1180, 1153, 926, 849, 764 cm^{-1}.

^{1}H NMR (400 MHz, $CDCl_3$): δ 8.93 (d, J = 4.5 Hz, 1H), 8.23 – 8.15 (m, 2H), 7.79 – 7.73 (m, 1H), 7.67 – 7.58 (m, 1H), 7.49 (dd, J = 4.4, 1.1 Hz, 1H), 5.30 (dd, $^3J_{HF}$ = 16.7 Hz, $^2J_{HH}$ = 3.4 Hz, 1H), 5.09 (dd, $^3J_{HF}$ = 47.9 Hz, $^2J_{HH}$ = 3.4 Hz, 1H).

^{13}C NMR (126 MHz, $CDCl_3$): δ 162.5, 160.5, 149.9, 149.9, 148.9, 148.7, 138.5, 138.3, 134.5, 134.1, 130.6, 130.1, 129.9, 128.1, 127.6, 127.0, 125.4, 125.4, 125.3, 125.1, 120.4, 97.7, 97.5, 85.5, 85.1, 82.9. *NB*. J_{CF} not indicated due to overlap of impurities.

^{19}F NMR (376 MHz, $CDCl_3$): δ −92.69.

HRMS (ESI): exact mass calculated for $[M+H]^+$ ($C_{11}H_9FN$) requires *m/z* 174.0714, found *m/z* 174.0709.

8-Chloro-2-(1-fluorovinyl)quinoline **5k**

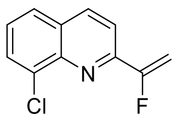

Prepared according to General Procedure C using 2-bromo-8-chloroquinoline (121 mg, 0.50 mmol, 1.0 equiv), (1-fluorovinyl)(methyl)diphenylsilane (182 mg, 0.75 mmol, 1.5 equiv), $Pd(PPh_3)_4$ (57.8 mg, 0.50 mmol, 1.0 equiv), CuI (6.6 mg, 0.50 mmol, 1.0 equiv), CsF (224 mg, 1.18 mmol, 2.35 equiv), and DMF (10 mL, 0.1 M). The reaction mixture was subjected to the purification outlined in General Procedure C (silica gel, Et_2O:petroleum ether, 1:99) to afford the desired product as a white solid (42.0 mg, 40%).

υ_{max} (film): 1655, 1595, 1499, 1425, 1312, 1287, 1206, 1123, 989, 841, 758, 669 cm^{-1}.

^{1}H NMR (400 MHz, $CDCl_3$): δ 8.20 (d, J = 8.6 Hz, 1H), 7.82 (dd, J = 7.5, 1.3 Hz, 1H), 7.72 (ddd, J = 8.1, 6.8, 1.4 Hz, 2H), 7.43 (dd, J = 8.2, 7.4 Hz, 1H), 6.06 (dd, $^3J_{HF}$ = 48.5 Hz, $^2J_{HH}$ = 2.9 Hz, 1H), 5.17 (dd, $^3J_{HF}$ = 16.0 Hz, $^2J_{HH}$ = 2.9 Hz, 1H).

^{13}C NMR (126 MHz, $CDCl_3$): δ 161.9 (d, ^1J = 250.7 Hz), 150.60 (d, ^2J = 37.5 Hz), 144.1 (d, ^4J = 4.5 Hz), 137.6, 133.9, 130.2, 129.2, 126.9, 126.7, 117.5 (d, ^3J = 4.4 Hz), 94.7 (d, ^2J = 17.6 Hz).

^{19}F NMR (376 MHz, $CDCl_3$): δ −115.61.

HRMS (ESI): exact mass calculated for $[M+H]^+$ ($C_{11}H_8ClFN$) requires *m/z* 208.0324, found *m/z* 208.0320.

(*R*)-*N*-(2-Fluoro-2-(quinolin-2-yl)ethyl)aniline **7a**

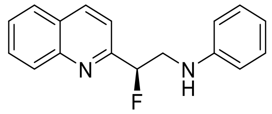

Prepared according to General Procedure A using 2-(1-fluorovinyl)quinoline (17.3 mg, 0.10 mmol, 1.0 equiv), aniline (27 μL, 0.30 mmol, 3.0 equiv), (*S*)-TRIP catalyst (15.1 mg, 0.02 mmol, 20 mol%), and THF (200 μL, 0.5 M). The reaction mixture was subjected to the purification outlined in General Procedure A (silica gel, EtOAc:petroleum ether, 5:95 to 10:90) to afford the desired product as a pale yellow solid (21.8 mg, 82%).

υ_{max} (film): 3406 (br), 3051, 3019, 2926, 2846, 1600, 1504, 1061, 751 cm^{-1}.

^{1}H NMR (500 MHz, $CDCl_3$): δ 8.24 (d, J = 8.5 Hz, 1H), 8.11 (d, J = 8.5 Hz, 1H), 7.85 (d, J = 8.2 Hz, 1H), 7.79 – 7.71 (m, 1H), 7.64 (dd, J = 8.5, 1.2 Hz, 1H), 7.60 – 7.54 (m, 1H), 7.23 – 7.15 (m, 2H), 6.79 – 6.70 (m, 3H), 5.91 (ddd, $^2J_{HF}$ = 48.7 Hz, J = 7.3, 3.7 Hz, 1H), 3.95 (ddd, $^3J_{HF}$ = 26.3 Hz, $^2J_{HH}$ = 14.0 Hz, J = 3.8 Hz, 1H), 3.73 (ddd, $^3J_{HF}$ = 19.7 Hz, $^2J_{HH}$ = 14.0 Hz, J = 7.3 Hz, 1H).

^{13}C NMR (126 MHz, $CDCl_3$): δ 158.1 (d, ^2J = 24.5 Hz), 147.6, 147.5, 137.3, 130.1, 129.5, 129.3, 127.9, 127.9, 127.0, 118.2, 117.9 (d, ^3J = 6.7 Hz), 113.5, 93.4 (d, ^1J = 174.8 Hz), 48.6 (d, ^2J = 22.5 Hz).

^{19}F NMR (376 MHz, $CDCl_3$): δ −191.33.

HRMS (NSI): exact mass calculated for $[M+H]^+$ ($C_{17}H_{16}N_2F$) requires *m/z* 267.1292, found *m/z* 267.1293.

The enantiomeric purity of the product was determined by HPLC analysis: 96:4 e.r. (Chiralpak IA, hexane/*i*-PrOH = 90/10, flow rate 0.5 mL/min, λ = 250 nm), t_r (major) = 23.1 min, t_r (minor) = 25.2 min;

$[\alpha]_D^{23}$ = +21.7 (*c* 1.01, $CHCl_3$).

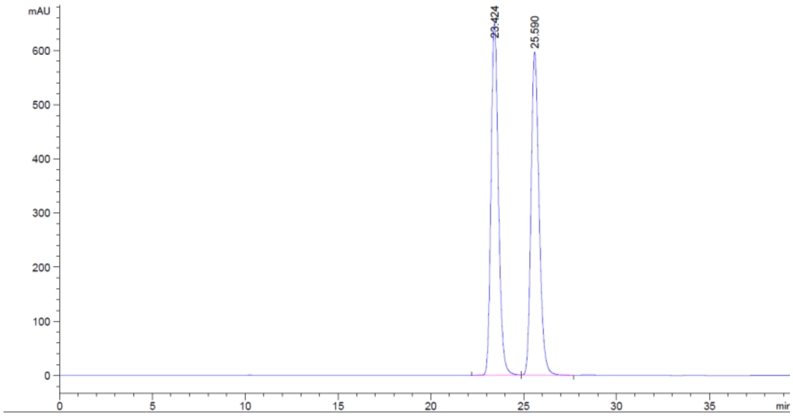

Area Percent Report

Sorted By : Signal
Multiplier : 1.0000
Dilution : 1.0000
Use Multiplier & Dilution Factor with ISTDs

Signal 1: VWD1 A, Wavelength=250 nm

Peak #	RetTime [min]	Type	Width [min]	Area [mAU*s]	Height [mAU]	Area %
1	23.424	BB	0.4287	1.80602e4	650.49353	50.0453
2	25.590	BB	0.4661	1.80275e4	596.60730	49.9547

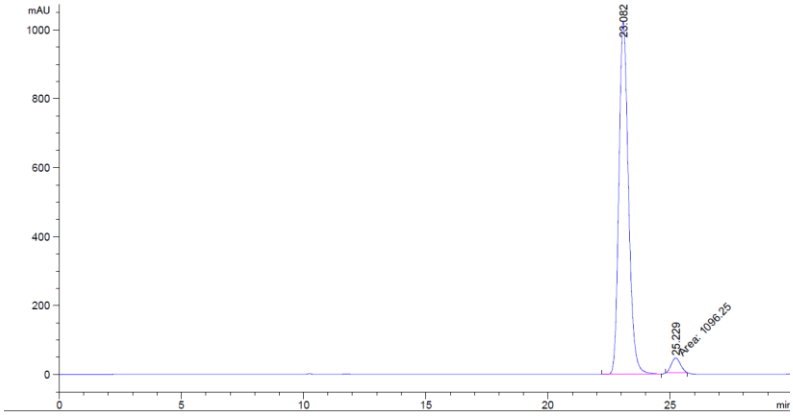

Area Percent Report

Sorted By : Signal
Multiplier : 1.0000
Dilution : 1.0000
Use Multiplier & Dilution Factor with ISTDs

Signal 1: VWD1 A, Wavelength=250 nm

Peak #	RetTime [min]	Type	Width [min]	Area [mAU*s]	Height [mAU]	Area %
1	23.082	BV	0.4204	2.79043e4	1022.16467	96.2199
2	25.229	MM	0.4293	1096.24890	42.55674	3.7801

(*R*)-*N*-(2-Fluoro-2-(quinolin-2-yl)ethyl)-4-(methylthio)aniline **7b**

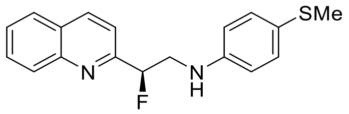

Prepared according to General Procedure B using 2-(1-fluorovinyl)quinoline (17.3 mg, 0.10 mmol, 1.0 equiv), 4-(methylthio)aniline (41.7 mg, 0.30 mmol, 3.0 equiv), (*S*)-TRIP catalyst (15.1 mg, 0.02 mmol, 20 mol%), and CPME (200 µL, 0.5 M). The reaction mixture was subjected to the purification outlined in General Procedure B (silica gel, EtOAc:petroleum ether, 5:95 to 10:90) to afford the desired product as a pale brown oil (29.0 mg, 93%).

υ_{max} (film): 3318 (br), 2970, 2916, 2839, 1597, 1504, 1319, 1034, 826 cm^{-1}.

^{1}H NMR (500 MHz, $CDCl_3$): δ 8.22 (d, *J* = 8.5 Hz, 1H), 8.10 (d, *J* = 8.5 Hz, 1H), 7.84 (d, *J* = 8.1 Hz, 1H), 7.79 – 7.72 (m, 1H), 7.63 (dd, *J* = 8.5, 1.2 Hz, 1H), 7.57 (t, *J* = 7.5 Hz, 1H), 7.21 (d, *J* = 8.6 Hz, 2H), 6.66 (d, *J* = 8.6 Hz, 2H), 5.89 (ddd, $^2J_{HF}$ = 48.6 Hz, *J* = 7.3, 3.7 Hz, 1H), 4.29 (s, 1H), 3.92 (ddd, $^3J_{HF}$ = 26.1 Hz, $^2J_{HH}$ = 14.1 Hz, *J* = 3.5 Hz, 1H), 3.71 (ddd, $^3J_{HF}$ = 21.0 Hz, $^2J_{HH}$ = 14.1 Hz, *J* = 7.3 Hz, 1H), 2.40 (s, 3H).

^{13}C NMR (126 MHz, $CDCl_3$): δ 158.1, 157.9, 146.5, 137.3, 131.4, 130.1, 129.2, 127.9, 127.0, 125.8, 125.2, 117.9 (d, ^3J = 6.4 Hz), 114.1, 93.3 (d, ^1J = 174.9 Hz), 48.6 (d, ^2J = 22.4 Hz), 19.1.

^{19}F NMR (470 MHz, $CDCl_3$): δ −191.43.

HRMS (NSI): exact mass calculated for $[M+H]^+$ ($C_{18}H_{18}N_2FS$) requires *m/z* 313.1169, found *m/z* 313.1165.

The enantiomeric purity of the product was determined by HPLC analysis: 97:3 e.r. (Chiralpak IA, hexane/*i*-PrOH = 90/10, flow rate 0.5 mL/min, λ = 250 nm), t_r (major) = 25.2 min, t_r (minor) = 27.1 min;

$[\alpha]_D^{20}$ = +4.4 (*c* 1.45, $CHCl_3$).

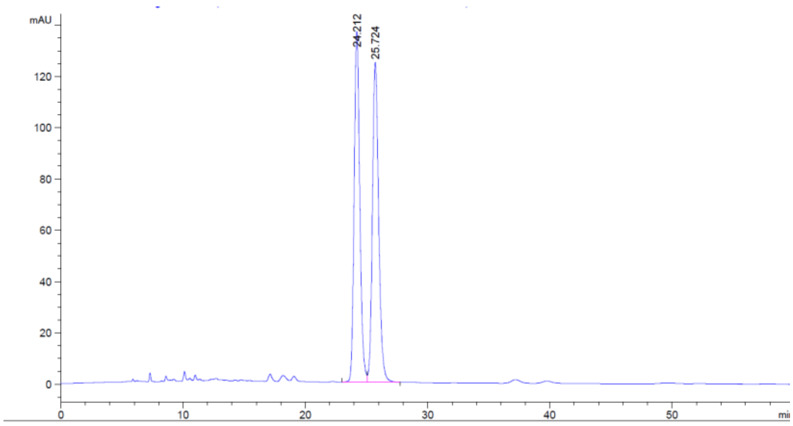


```
==================================================================
                       Area Percent Report
==================================================================

3orted By              :      Signal
4ultiplier             :      1.0000
)ilution               :      1.0000
Jse Multiplier & Dilution Factor with ISTDs


3ignal 1: VWD1 A, Wavelength=250 nm

Peak RetTime Type  Width      Area       Height     Area
  #    [min]        [min]    [mAU*s]     [mAU]       %
----|-------|----|-------|----------|----------|--------|
   1  24.212 BV     0.4947 4403.19873  136.88387  49.7183
   2  25.724 VB     0.5457 4453.10205  124.86521  50.2817
```

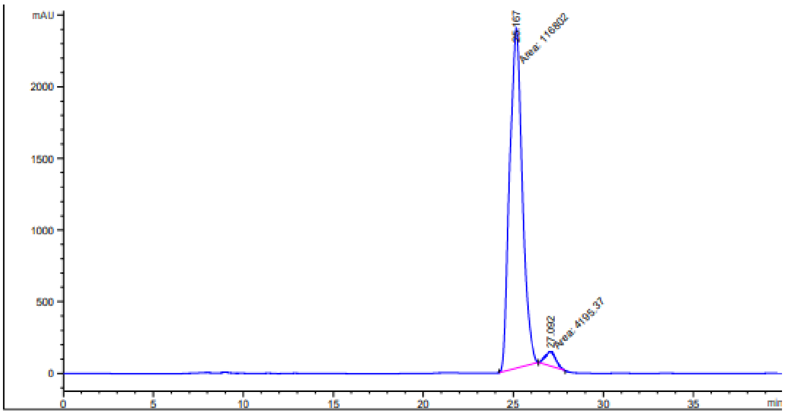


```
==================================================================
                       Area Percent Report
==================================================================

Sorted By              :      Signal
Multiplier             :      1.0000
Dilution               :      1.0000
Use Multiplier & Dilution Factor with ISTDs


Signal 1: VWD1 A, Wavelength=250 nm

Peak RetTime Type  Width      Area       Height     Area
  #    [min]        [min]    [mAU*s]     [mAU]       %
----|-------|----|-------|----------|----------|--------|
   1  25.167 MM     0.8190 1.16802e5 2376.94727  96.5327
   2  27.092 MM     0.6820 4195.36768  102.52654   3.4673
```

(*R*)-*N*-(2-Fluoro-2-(quinolin-2-yl)ethyl)-2,3-dihydro-1*H*-inden-5-amine **7c**

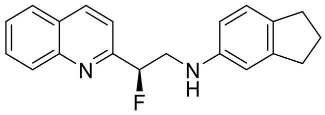

Prepared according to General Procedure B using 2-(1-fluorovinyl)quinoline (17.3 mg, 0.10 mmol, 1.0 equiv), 2,3-dihydro-1*H*-inden-5-amine (39.9 mg, 0.30 mmol, 3.0 equiv), (*S*)-TRIP catalyst (15.1 mg,

0.02 mmol, 20 mol%), and CPME (200 µL, 0.5 M). The reaction mixture was subjected to the purification outlined in General Procedure B (silica gel, EtOAc:petroleum ether, 5:95 to 10:90) to afford the desired product as a pale yellow oil (27.2 mg, 89%).

υ_{max} (film): 3395 (br), 3041, 3006, 2930, 2839, 1599, 1502, 1058, 833 cm^{-1}.

^{1}H NMR (500 MHz, CDCl$_3$): δ 8.22 (d, J = 8.5 Hz, 1H), 8.10 (d, J = 8.7 Hz, 1H), 7.84 (d, J = 8.1 Hz, 1H), 7.75 (ddd, J = 8.4, 6.9, 1.5 Hz, 1H), 7.63 (dd, J = 8.5, 1.5 Hz, 1H), 7.57 (ddd, J = 8.1, 6.9, 1.2 Hz, 1H), 7.04 (d, J = 8.0 Hz, 1H), 6.67 – 6.61 (m, 1H), 6.54 (dd, J = 8.0, 2.3 Hz, 1H), 5.90 (ddd, $^2J_{HF}$ = 48.8 Hz, J = 7.4, 3.7 Hz, 1H), 4.13 (s, 1H), 3.92 (ddd, $^3J_{HF}$ = 26.4 Hz, $^2J_{HH}$ =14.0 Hz, J = 3.7 Hz, 1H), 3.70 (ddd, $^3J_{HF}$ = 19.6 Hz, $^2J_{HH}$ = 14.0 Hz, J = 7.4 Hz, 1H), 2.86 – 2.75 (m, 4H), 2.12 – 1.90 (m, 2H).

^{13}C NMR (126 MHz, CDCl$_3$): δ 158.3 (d, ^2J = 24.5 Hz), 147.5, 146.4, 145.6, 137.2, 134.0, 130.0, 129.4, 127.8, 126.9, 124.9, 118.0, 117.9, 112.0, 109.8, 93.4 (d, ^1J = 174.4 Hz), 49.2 (d, ^2J = 22.4 Hz), 33.2, 32.1, 25.8.

^{19}F NMR (376 MHz, CDCl$_3$): δ −191.30.

HRMS (NSI): exact mass calculated for $[M+H]^+$ ($C_{20}H_{20}N_2F$) requires *m/z* 307.1605, found *m/z* 307.1607.

The enantiomeric purity of the product was determined by HPLC analysis: 91:9 e.r. (Chiralcel OJ-H, hexane/*i*-PrOH = 50/50, flow rate 1.0 mL/min, λ = 210 nm), t_r (major) = 25.7 min, t_r (minor) = 38.8 min;

$[\alpha]_D^{20}$ = +7.0 (*c* 1.36, CHCl$_3$).

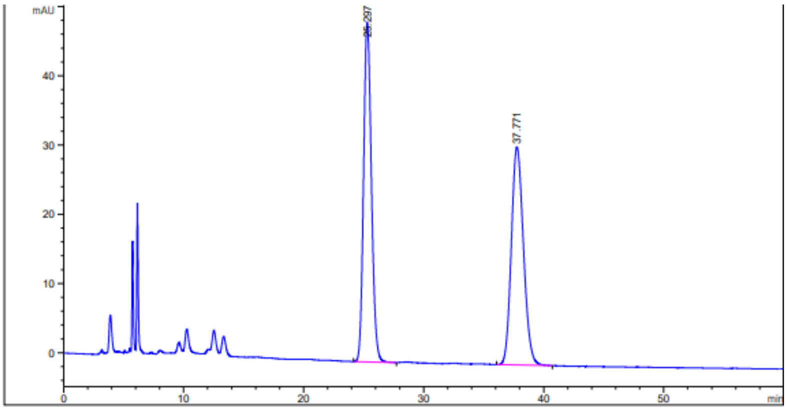

Area Percent Report

Sorted By : Signal
Multiplier : 1.0000
Dilution : 1.0000
Use Multiplier & Dilution Factor with ISTDs

Signal 1: VWD1 A, Wavelength=250 nm

Peak #	RetTime [min]	Type	Width [min]	Area [mAU*s]	Height [mAU]	Area %
1	25.297	BB	0.7026	2218.92896	49.04015	50.0879
2	37.771	BB	1.0750	2211.13745	31.50676	49.9121

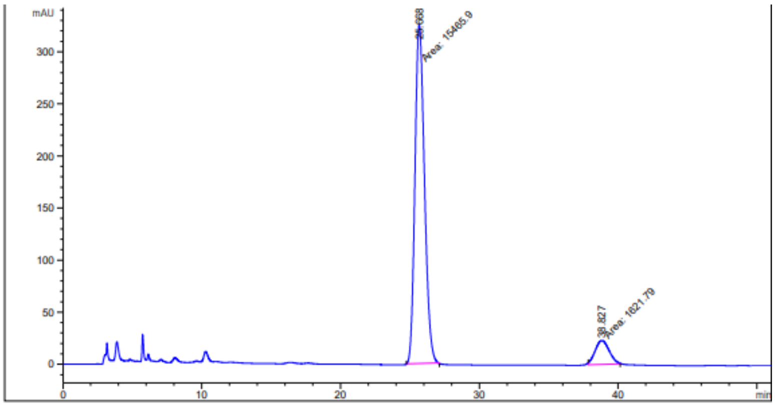

Area Percent Report

Sorted By : Signal
Multiplier : 1.0000
Dilution : 1.0000
Use Multiplier & Dilution Factor with ISTDs

Signal 1: VWD1 A, Wavelength=210 nm

Peak #	RetTime [min]	Type	Width [min]	Area [mAU*s]	Height [mAU]	Area %
1	25.668	MM	0.7928	1.54659e4	325.14124	90.5090
2	38.827	MM	1.1705	1621.78674	23.09332	9.4910

(*R*)-3-Bromo-*N*-(2-fluoro-2-(quinolin-2-yl)ethyl)aniline **7d**

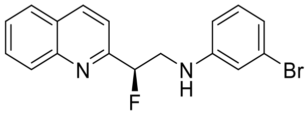

Prepared according to General Procedure B using 2-(1-fluorovinyl)quinoline (17.3 mg, 0.10 mmol, 1.0 equiv), 3-bromoaniline (53.1 mg, 0.30 mmol, 3.0 equiv), (*S*)-TRIP catalyst (15.1 mg, 0.02 mmol, 20 mol%), and CPME (200 µL, 0.5 M). The reaction mixture was subjected to the purification outlined in General Procedure B (silica gel, EtOAc:petroleum ether, 5:95 to 10:90) to afford the desired product as a pale yellow oil (30.3 mg, 88%).

υ_{max} (film): 3418 (br), 3063, 2924, 2847, 1589, 1504, 1065, 833, 764 cm^{-1}.

^{1}H NMR (400 MHz, CDCl$_3$): δ 8.23 (d, J = 8.5 Hz, 1H), 8.11 (d, J = 8.5 Hz, 1H), 7.85 (d, J = 8.2 Hz, 1H), 7.76 (ddd, J = 8.4, 6.9, 1.4 Hz, 1H), 7.63 (dd, J = 8.5, 1.3 Hz, 1H), 7.58 (ddd, J = 8.0, 7.0, 1.0 Hz, 1H), 7.01 (t, J = 8.0 Hz, 1H), 6.87 – 6.79 (m, 2H), 6.65 – 6.57 (m, 1H), 5.89 (ddd, $^2J_{HF}$ = 48.5 Hz, J = 7.0, 3.8 Hz, 1H), 4.33 (s, 1H), 3.91 (ddd, $^3J_{HF}$ = 25.0 Hz, $^2J_{HH}$ = 14.1 Hz, J = 3.8 Hz, 1H), 3.73 (ddd, $^3J_{HF}$ = 20.8 Hz, $^2J_{HH}$ = 14.1 Hz, J = 7.0 Hz, 1H).

^{13}C NMR (126 MHz, CDCl$_3$): δ 157.7 (d, ^2J = 24.6 Hz), 149.0, 147.4 (d, ^4J = 2.3 Hz), 137.4, 130.6, 130.2, 129.2, 127.9, 127.8, 127.0, 123.4, 120.8, 117.8 (d, ^3J = 6.5 Hz), 115.9, 112.0, 93.2 (d, ^1J = 175.2 Hz), 48.2 (d, ^2J = 22.4 Hz).

^{19}F NMR (377 MHz, CDCl$_3$): δ −191.34.

HRMS (ESI): exact mass calculated for $[M+H]^+$ ($C_{17}H_{15}N_2BrF$) requires *m/z* 345.0397, found *m/z* 345.0395.

The enantiomeric purity of the product was determined by HPLC analysis: 94:6 e.r. (Chiralpak IA, hexane/*i*-PrOH = 90/10, flow rate 0.5 mL/min, λ = 250 nm), t_r (major) = 16.7 min, t_r (minor) = 17.5 min;

$[\alpha]_D^{20}$ = +31.4 (*c* 2.12, $CHCl_3$).

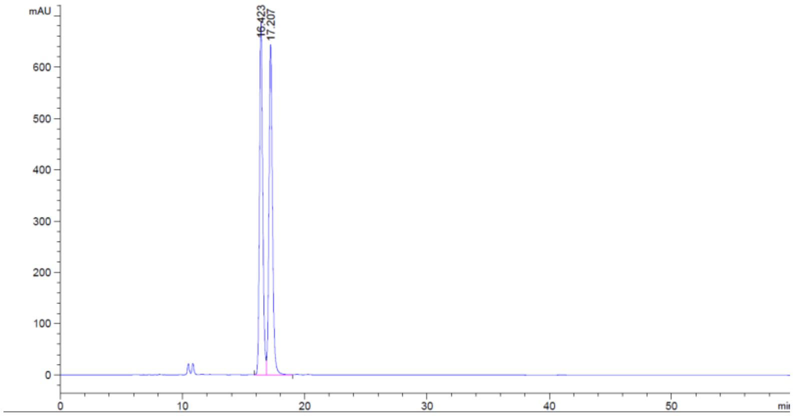

Area Percent Report

Sorted By : Signal
Multiplier : 1.0000
Dilution : 1.0000
Use Multiplier & Dilution Factor with ISTDs

Signal 1: VWD1 A, Wavelength=250 nm

Peak #	RetTime [min]	Type	Width [min]	Area [mAU*s]	Height [mAU]	Area %
1	16.423	BV	0.2796	1.25106e4	686.43469	49.3029
2	17.207	VB	0.3046	1.28644e4	643.45532	50.6971

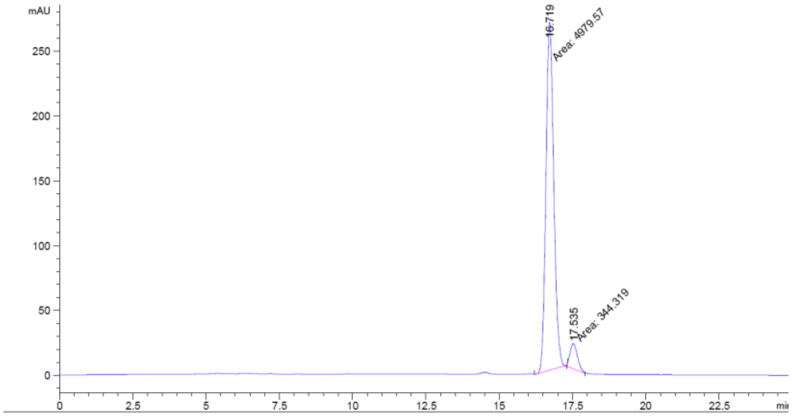

Area Percent Report

Sorted By : Signal
Multiplier : 1.0000
Dilution : 1.0000
Use Multiplier & Dilution Factor with ISTDs

Signal 1: VWD1 A, Wavelength=250 nm

Peak #	RetTime [min]	Type	Width [min]	Area [mAU*s]	Height [mAU]	Area %
1	16.719	MM	0.3096	4979.56592	268.05878	93.5326
2	17.535	MM	0.2954	344.31888	19.42668	6.4674

(*R*)-*N*-(2-Fluoro-2-(quinolin-2-yl)ethyl)-4-(trifluoromethyl)aniline **7e**

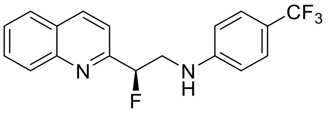

Prepared according to General Procedure B using 2-(1-fluorovinyl)quinoline (17.3 mg, 0.10 mmol, 1.0 equiv), 4-(trifluoromethyl)aniline (48.3 mg, 0.30 mmol, 3.0 equiv), (*S*)-TRIP catalyst (15.1 mg, 0.02 mmol, 20 mol%), and CPME (200 µL, 0.5 M). The reaction mixture was subjected to the purification outlined in General Procedure B (silica gel, EtOAc:petroleum ether, 5:95 to 10:90) to afford the desired product as a pale yellow oil (31.2 mg, 93%).

υ_{max} (film): 3426 (br), 3055, 2963, 2839, 1612, 1535, 1319, 1103, 1065, 826 cm^{-1}.

^{1}H NMR (500 MHz, $CDCl_3$): δ 8.24 (d, J = 8.5 Hz, 1H), 8.11 (d, J = 8.5 Hz, 1H), 7.85 (dd, J = 8.1, 1.4 Hz, 1H), 7.77 (ddd, J = 8.4, 6.8, 1.4 Hz, 1H), 7.64 (dd, J = 8.5, 1.6 Hz, 1H), 7.59 (ddd, J = 8.2, 6.9, 1.2 Hz, 1H), 7.40 (d, J = 8.5 Hz, 2H), 6.71 (d, J = 8.5 Hz, 2H), 5.90 (ddd, $^2J_{HF}$ = 48.5 Hz, J = 7.0, 3.8 Hz, 1H), 4.61 (s, 1H), 3.99 (ddd, $^3J_{HF}$ = 25.1 Hz, $^2J_{HH}$ = 14.2 Hz, J = 3.8 Hz, 1H), 3.80 (ddd, $^3J_{HF}$ = 20.9 Hz, $^2J_{HH}$ = 14.2 Hz, J = 7.0 Hz, 1H).

^{13}C NMR (126 MHz, $CDCl_3$): δ 157.6 (d, ^2J = 24.8 Hz), 150.2, 147.4, 137.5, 130.3, 129.2, 127.9, 127.9, 127.1, 126.8 (q, ^3J = 3.8 Hz), 125.0 (q, ^1J = 270.4 Hz), 119.6 (q, ^2J = 32.6 Hz), 117.8 (d, ^3J = 6.7 Hz), 112.5, 93.1 (d, ^1J = 175.6 Hz), 47.9 (d, ^2J = 22.4 Hz).

^{19}F NMR (470 MHz, $CDCl_3$): δ −61.07, −191.49.

HRMS (NSI): exact mass calculated for $[M+H]^+$ ($C_{18}H_{15}N_2F_4$) requires *m/z* 335.1166, found *m/z* 336.1158.

The enantiomeric purity of the product was determined by HPLC analysis: 97:3 e.r. (Chiralpak IA, hexane/*i*-PrOH = 90/10, flow rate 0.5 mL/min, λ = 250 nm), tr (major) = 17.2 min, tr (minor) = 18.2 min;

$[\alpha]_D^{20}$ = +4.7 (*c* 1.07, $CHCl_3$).

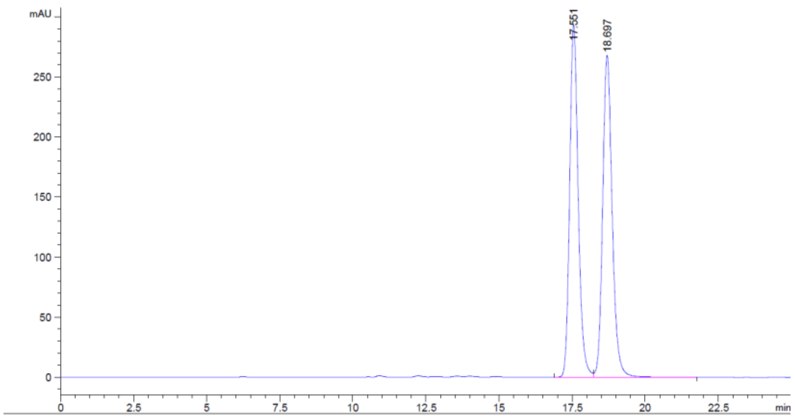

Area Percent Report

Sorted By : Signal
Multiplier : 1.0000
Dilution : 1.0000
Use Multiplier & Dilution Factor with ISTDs

Signal 1: VWD1 A, Wavelength=250 nm

Peak #	RetTime [min]	Type	Width [min]	Area [mAU*s]	Height [mAU]	Area %
1	17.551	BV	0.3124	5980.82959	292.94598	49.6500
2	18.697	VB	0.3454	6065.15576	268.12650	50.3500

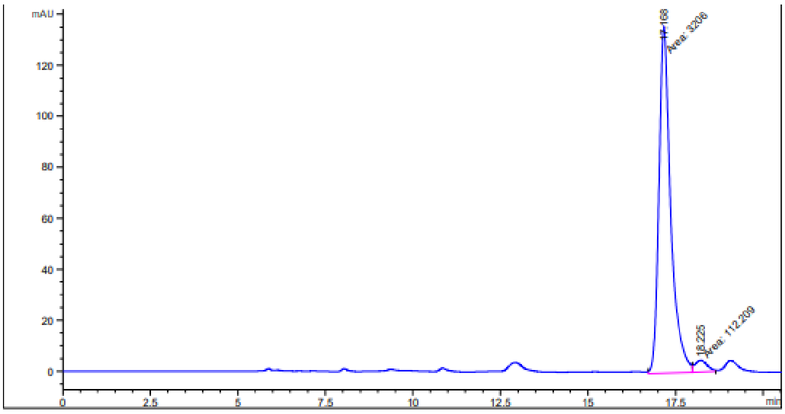

Area Percent Report

Sorted By : Signal
Multiplier : 1.0000
Dilution : 1.0000
Use Multiplier & Dilution Factor with ISTDs

Signal 1: VWD1 A, Wavelength=250 nm

Peak #	RetTime [min]	Type	Width [min]	Area [mAU*s]	Height [mAU]	Area %
1	17.168	MF	0.3925	3205.99536	136.12822	96.6184
2	18.225	FM	0.4026	112.20947	4.64504	3.3816

(R)-*N*-(2-Fluoro-2-(quinolin-2-yl)ethyl)-4-(4,4,5,5-tetramethyl-1,3,2-dioxaborolan-2-yl)aniline **7f**

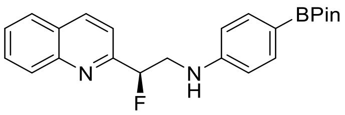

Prepared according to General Procedure A using 2-(1-fluorovinyl)quinoline (17.3 mg, 0.10 mmol, 1.0 equiv), 4-(4,4,5,5-tetramethyl-1,3,2-dioxaborolan-2-yl)aniline (65.7 mg, 0.30 mmol, 3.0 equiv), (*S*)-TRIP catalyst (15.1 mg, 0.02 mmol, 20 mol%), and THF (200 µL, 0.5 M) at −20 °C for 5 d. The reaction mixture was subjected to the purification outlined in General Procedure A (silica gel, EtOAc:petroleum ether, 5:95 to 10:90) to afford the desired product as a pale yellow solid (31.0 mg, 79%).

υ_{max} (film): 3401 (br), 3041, 2973, 2928, 1606, 1359, 1145 cm^{-1}.

^{1}H NMR (400 MHz, CD_2Cl_2): δ 8.27 (d, J = 8.5 Hz, 1H), 8.09 (d, J = 8.5 Hz, 1H), 7.88 (d, J = 8.2 Hz, 1H), 7.76 (ddd, J = 8.4, 6.9, 1.4 Hz, 1H), 7.63 (dd, J = 8.5, 1.4 Hz, 1H), 7.61 – 7.50 (m, 3H), 6.75 – 6.63 (m, 2H), 5.87 (ddd, $^{2}J_{HF}$ = 48.6 Hz, J = 7.1, 3.8 Hz, 1H), 4.52 (s, 1H), 3.97 (m, 1H), 3.79 (ddd, $^{3}J_{HF}$ = 20.1 Hz, $^{2}J_{HH}$ = 13.4 Hz, J = 6.3 Hz, 1H), 1.30 (s, 12H).

^{13}C NMR (101 MHz, CD_2Cl_2): δ 158.2 (d, ^{2}J = 24.4 Hz), 150.6, 137.5, 136.6, 136.6, 130.3, 129.5, 128.2, 128.1, 128.1, 127.2, 118.2 (d, ^{3}J = 6.1 Hz), 112.5, 93.7 (d, ^{1}J = 174.8 Hz), 83.6, 48.0 (d, ^{2}J = 22.4 Hz), 25.1.

^{19}F NMR (376 MHz, CD_2Cl_2): δ −190.85.

^{11}B NMR (128 MHz, CD_2Cl_2): δ 30.69.

HRMS (NSI): exact mass calculated for $[M+H]^+$ ($C_{23}H_{27}N_2O_2FB$) requires *m/z* 392.2154, found *m/z* 393.2202.

The enantiomeric purity of the product was determined by HPLC analysis: 93:7 e.r. (Chiralpak IA, hexane/*i*-PrOH = 90/10, flow rate 0.5 mL/min, λ = 250 nm), t_r (major) = 17.5 min, t_r (minor) = 18.9 min;

$[\alpha]_D^{20}$ = + 43.4 (*c* 0.15, $CHCl_3$).

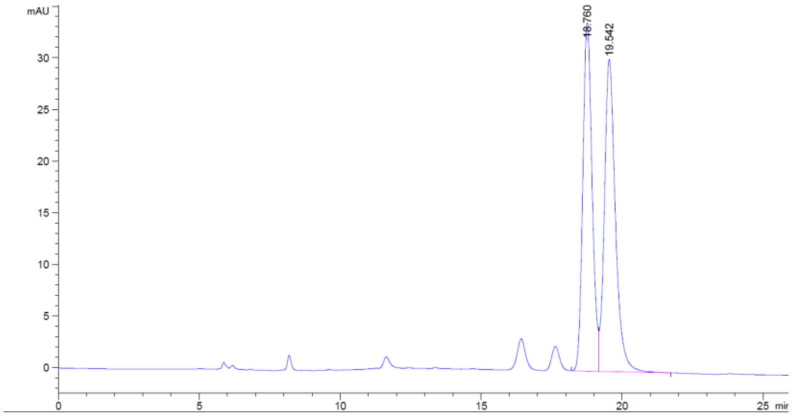


```
=====================================================================
                       Area Percent Report
=====================================================================

iorted By              :      Signal
fultiplier             :      1.0000
)ilution               :      1.0000
Jse Multiplier & Dilution Factor with ISTDs


iignal 1: VWD1 A, Wavelength=250 nm

'eak RetTime Type  Width     Area      Height     Area
 #   [min]         [min]   [mAU*s]     [mAU]       %
----|-------|----|-------|----------|----------|--------|
  1  18.760 BV    0.3529  772.33557   33.74578  48.4127
  2  19.542 VB    0.4107  822.97955   30.23723  51.5873
```

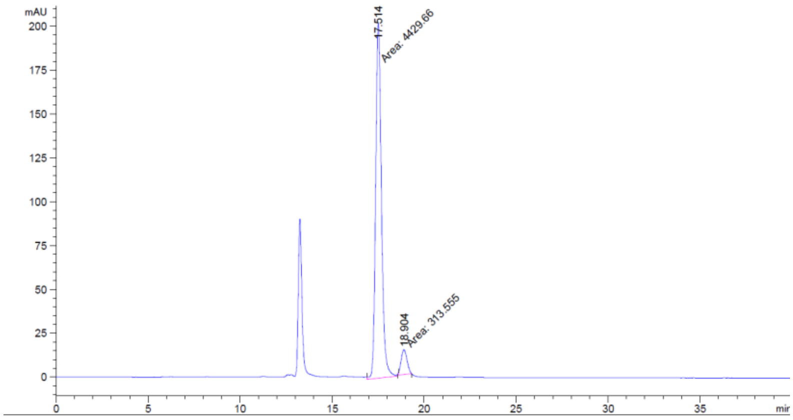


```
=================================================================
                      Area Percent Report
=================================================================

orted By             :      Signal
ultiplier            :      1.0000
ilution              :      1.0000
se Multiplier & Dilution Factor with ISTDs


ignal 1: VWD1 A, Wavelength=250 nm

eak RetTime Type  Width     Area      Height     Area
 #   [min]        [min]   [mAU*s]     [mAU]        %
---|-------|----|-------|----------|----------|--------|
  1  17.514 MM    0.3651 4429.66260  202.23083  93.3894
  2  18.904 MM    0.3693  313.55548   14.15067   6.6106
```

(R)-4-Bromo-*N*-(2-fluoro-2-(quinolin-2-yl)ethyl)aniline **7g**

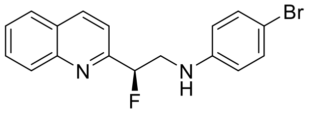

Prepared according to General Procedure A using 2-(1-fluorovinyl)quinoline (17.3 mg, 0.10 mmol, 1.0 equiv), 4-bromoaniline (51.6 mg, 0.30 mmol, 3.0 equiv), (*S*)-TRIP catalyst (15.1 mg, 0.02 mmol, 20 mol%), and THF (200 µL, 0.5 M) at −20 °C for 5 d. The reaction mixture was subjected to the purification outlined in General Procedure A (silica gel, EtOAc:petroleum ether, 5:95 to 10:90) to afford the desired product as a pale yellow oil (24.4 mg, 71%).

υ_{max} (film): 3403 (br), 3026, 2928, 2851, 1599, 813 cm^{-1}.

^{1}H NMR (400 MHz, CDCl$_3$): δ 8.23 (d, *J* = 8.5 Hz, 1H), 8.09 (d, *J* = 8.4 Hz, 1H), 7.85 (d, *J* = 8.1 Hz, 1H), 7.76 (ddd, *J* = 8.4, 6.9, 1.4 Hz, 1H), 7.63 (dd, *J* = 8.5, 1.5 Hz, 1H), 7.58 (ddd, *J* = 8.1, 6.9, 1.1 Hz, 1H), 7.26 – 7.22 (m, 1H), 6.62 – 6.56 (m, 2H), 5.87 (ddd, $^{2}J_{HF}$ = 48.7 Hz, *J* = 7.1, 3.8 Hz, 2H), 4.28 (t, *J* = 5.8 Hz, 1H), 3.91 (dddd, $^{3}J_{HF}$ = 25.4 Hz, $^{2}J_{HH}$ = 14.0 Hz, *J* = 7.3, 3.8 Hz, 1H), 3.78 – 3.65 (m, 1H).

^{13}C NMR (101 MHz, CDCl$_3$): δ 157.9 (d, ^{2}J = 24.5 Hz), 146.7, 137.3, 132.2, 132.1, 130.2, 129.4, 127.9, 127.0, 117.9 (d, ^{3}J = 6.9 Hz), 115.0, 109.8, 94.1, 93.3 (d, ^{1}J = 175.0 Hz), 48.5 (d, ^{2}J = 22.4 Hz).

^{19}F NMR (376 MHz, CDCl$_3$): δ −191.44.

HRMS (NSI): exact mass calculated for [M+H]$^{+}$ ($C_{17}H_{15}N_2FBr$) requires *m/z* 345.0403, found *m/z* 345.0400.

The enantiomeric purity of the product was determined by HPLC analysis: 90:10 e.r. (Chiralpak IA, hexane/*i*-PrOH = 50/50, flow rate 0.3 mL/min, λ = 250 nm), t_r (major) = 14.1 min, t_r (minor) = 15.0 min;

$[\alpha]_D^{20}$ = +58.6 (*c* 1.22, $CHCl_3$).

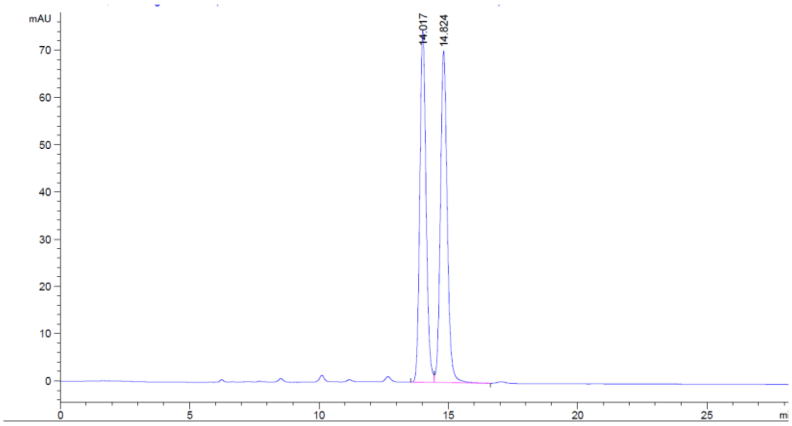

Area Percent Report

Sorted By : Signal
Multiplier : 1.0000
Dilution : 1.0000
Use Multiplier & Dilution Factor with ISTDs

Signal 1: VWD1 A, Wavelength=250 nm

Peak #	RetTime [min]	Type	Width [min]	Area [mAU*s]	Height [mAU]	Area %
1	14.017	BV	0.2586	1251.85400	74.50861	49.3576
2	14.824	VB	0.2801	1284.44275	70.30343	50.6424

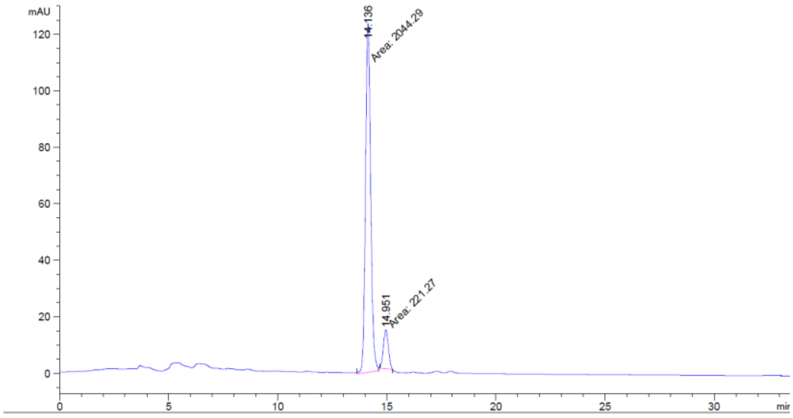

Area Percent Report

Sorted By : Signal
Multiplier : 1.0000
Dilution : 1.0000
Use Multiplier & Dilution Factor with ISTDs

Signal 1: VWD1 A, Wavelength=250 nm

Peak #	RetTime [min]	Type	Width [min]	Area [mAU*s]	Height [mAU]	Area %
1	14.136	MM	0.2758	2044.29187	123.52139	90.2333
2	14.951	MM	0.2681	221.27008	13.75688	9.7667

(R)-2-Fluoro-*N*-(2-fluoro-2-(quinolin-2-yl)ethyl)aniline **7h**

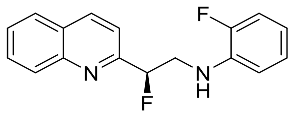

Prepared according to General Procedure B using 2-(1-fluorovinyl)quinoline (17.3 mg, 0.10 mmol, 1.0 equiv), 2-fluoroaniline (33.3 mg, 0.30 mmol, 3.0 equiv), (*S*)-TRIP catalyst (15.1 mg, 0.02 mmol, 20 mol%), and CPME (200 μL, 0.5 M). The reaction mixture was subjected to the purification outlined in General Procedure B (silica gel, EtOAc:petroleum ether, 5:95 to 10:90) to afford the desired product as a pale yellow oil (13.1 mg, 46%).

υ_{max} (film): 3422 (br), 3065, 2928, 2857, 1620, 1516, 1506, 1190, 829, 743 cm^{-1}.

^{1}H NMR (400 MHz, $CDCl_3$): δ 8.24 (d, J = 8.5 Hz, 1H), 8.10 (d, J = 8.8 Hz, 1H), 7.85 (d, J = 7.2 Hz, 1H), 7.76 (ddd, J = 8.4, 6.9, 1.4 Hz, 1H), 7.65 (dd, J = 8.5, 1.6 Hz, 1H), 7.57 (ddd, J = 8.1, 6.9, 1.1 Hz, 1H), 7.04 – 6.91 (m, 2H), 6.84 (td, J = 8.5, 1.4 Hz, 1H), 6.69 – 6.55 (m, 1H), 5.91 (ddd, $^{2}J_{HF}$ = 48.6 Hz, J = 7.1, 3.8 Hz, 1H), 4.56 (s, 1H), 3.97 (dddd, $^{3}J_{HF}$ = 25.3 Hz, $^{2}J_{HH}$ = 14.1 Hz, J = 7.1, 3.7 Hz, 1H), 3.84 – 3.71 (m, 1H).

^{13}C NMR (101 MHz, $CDCl_3$): δ 158.0 (d, ^{2}J = 24.6 Hz), 151.9 (d, ^{1}J = 238.8 Hz), 147.6 (d, ^{4}J = 1.8 Hz), 137.3, 136.2 (d, ^{3}J = 11.0 Hz), 130.1, 129.4, 127.9, 127.0, 124.7, 124.7, 117.9 (d, ^{3}J = 6.9 Hz), 117.4 (d, ^{3}J = 7.1 Hz), 114.7 (d, ^{2}J = 18.7 Hz), 112.7 (d, ^{4}J = 2.7 Hz), 93.3 (d, ^{1}J = 175.4 Hz), 48.2 (d, ^{2}J = 22.4 Hz).

^{19}F NMR (376 MHz, $CDCl_3$): δ −136.12, −191.53.

HRMS (NSI): exact mass calculated for $[M+H]^+$ ($C_{17}H_{15}N_2F_2$) requires *m/z* 285.1203, found *m/z* 285.1202.

The enantiomeric purity of the product was determined by HPLC analysis: 89:11 e.r. (Chiralcel OJ-H, hexane/*i*-PrOH = 50/50, flow rate 0.5 mL/min, λ = 250 nm), t_r (major) = 19.7 min, t_r (minor) = 22.0 min;

$[\alpha]_D^{20}$ = +3.4 (*c* 0.65, $CHCl_3$).

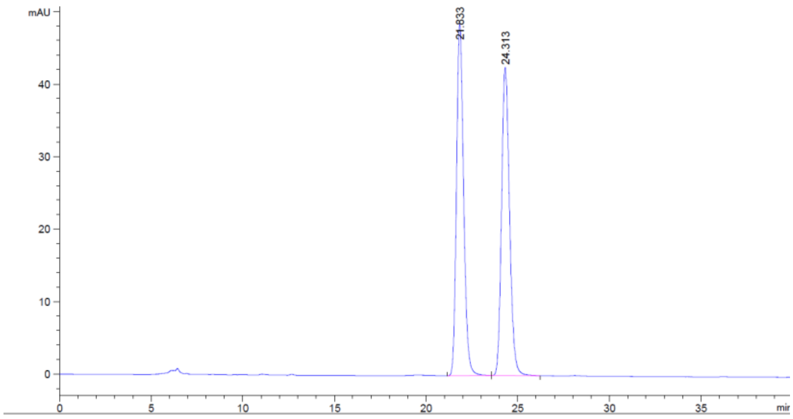


```
=====================================================================
                        Area Percent Report
=====================================================================

;orted By             :      Signal
4ultiplier            :      1.0000
)ilution              :      1.0000
Jse Multiplier & Dilution Factor with ISTDs


;ignal 1: VWD1 A, Wavelength=250 nm
```

Peak #	RetTime [min]	Type	Width [min]	Area [mAU*s]	Height [mAU]	Area %
1	21.833	BB	0.4178	1311.95435	48.45583	50.5078
2	24.313	BB	0.4666	1285.57263	42.48009	49.4922

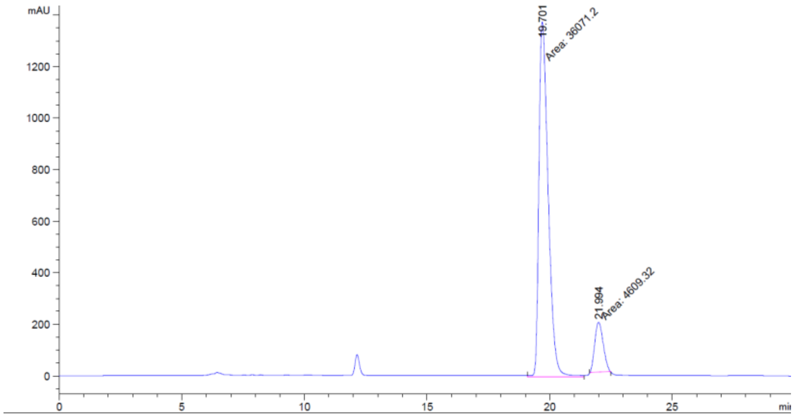


```
=====================================================================
                        Area Percent Report
=====================================================================

;orted By             :      Signal
4ultiplier            :      1.0000
)ilution              :      1.0000
Jse Multiplier & Dilution Factor with ISTDs


;ignal 1: VWD1 A, Wavelength=250 nm
```

Peak #	RetTime [min]	Type	Width [min]	Area [mAU*s]	Height [mAU]	Area %
1	19.701	MM	0.4365	3.60712e4	1377.26685	88.6695
2	21.994	MM	0.3972	4609.32080	193.43065	11.3305

(R)-3-Fluoro-*N*-(2-fluoro-2-(quinolin-2-yl)ethyl)-4-methylaniline **7i**

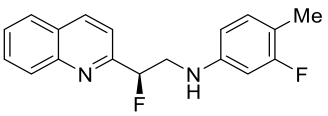

Prepared according to General Procedure B using 2-(1-fluorovinyl)quinoline (17.3 mg, 0.10 mmol, 1.0 equiv), 3-fluoro-4-methylaniline (37.5 mg, 0.30 mmol, 3.0 equiv), (*S*)-TRIP catalyst (15.1 mg, 0.02

mmol, 20 mol%), and CPME (200 µL, 0.5 M). The reaction mixture was subjected to the purification outlined in General Procedure B (silica gel, EtOAc:petroleum ether, 5:95 to 10:90) to afford the desired product as a pale yellow oil (29.2 mg, 98%).

υ_{max} (film): 3433 (br), 3048, 2924, 2862, 1636, 1597, 1520, 833 cm^{-1}.

^{1}H NMR (500 MHz, CDCl$_3$): δ 8.23 (d, J = 8.5 Hz, 1H), 8.09 (d, J = 8.5 Hz, 1H), 7.84 (d, J = 8.1 Hz, 1H), 7.78 – 7.71 (m, 1H), 7.66 – 7.60 (m, 1H), 7.57 (t, J = 7.4 Hz, 1H), 6.82 (t, J = 9.1 Hz, 1H), 6.52 (dd, J = 13.3, 2.7 Hz, 1H), 6.41 (dd, J = 8.7, 1.2 Hz, 1H), 5.87 (ddd, $^{2}J_{HF}$ = 48.7 Hz, J = 7.2, 3.7 Hz, 1H), 4.12 (s, 1H), 3.94 – 3.82 (m, 1H), 3.74 – 3.62 (m, 1H), 2.15 (s, 3H).

^{13}C NMR (126 MHz, CDCl$_3$): δ 162.2 (d, ^{1}J = 242.4 Hz), 157.9 (d, ^{2}J = 24.6 Hz), 147.4, 147.3 (d, ^{3}J = 10.6 Hz), 137.3, 131.8 (d, ^{3}J = 7.1 Hz), 130.1, 129.3, 127.9, 127.8, 127.0, 117.8 (d, ^{3}J = 6.5 Hz), 113.6 (d, ^{2}J = 17.7 Hz), 109.15 (d, ^{4}J = 2.4 Hz), 100.4 (d, ^{2}J = 26.1 Hz), 93.2 (d, ^{1}J = 174.8 Hz), 48.7 (d, ^{2}J = 22.4 Hz), 13.7 (d, ^{4}J = 3.3 Hz).

^{19}F NMR (470 MHz, CDCl$_3$): δ −116.69, −191.35.

HRMS (ESI): exact mass calculated for $[M+H]^+$ ($C_{18}H_{17}ON_2F_2$) requires *m/z* 299.1354, found *m/z* 299.1351.

The enantiomeric purity of the product was determined by HPLC analysis: 98:2 e.r. (Chiralpak IA, hexane/*i*-PrOH = 90/10, flow rate 0.3 mL/min, λ = 250 nm), t_r (major) = 16.3 min, t_r (minor) = 17.1 min;

$[\alpha]_D^{20}$ = +14.6 (*c* 1.6, CHCl$_3$).

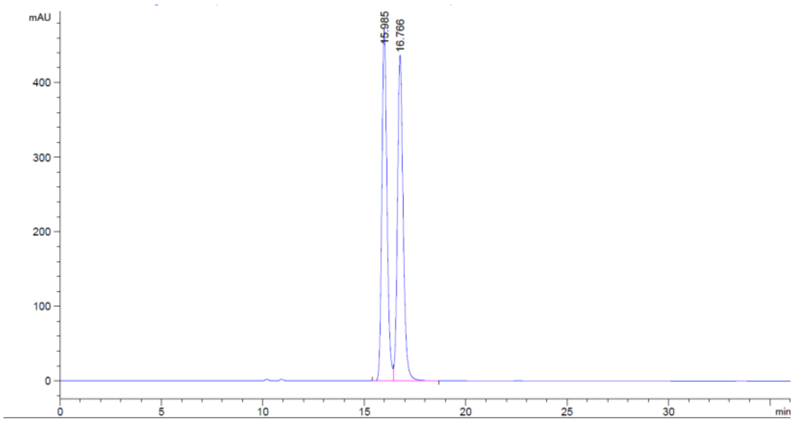

Area Percent Report

Sorted By : Signal
Multiplier : 1.0000
Dilution : 1.0000
Use Multiplier & Dilution Factor with ISTDs

Signal 1: VWD1 A, Wavelength=250 nm

Peak #	RetTime [min]	Type	Width [min]	Area [mAU*s]	Height [mAU]	Area %
1	15.985	BV	0.2709	8308.93359	471.99512	49.3547
2	16.766	VB	0.2976	8526.21680	436.84406	50.6453

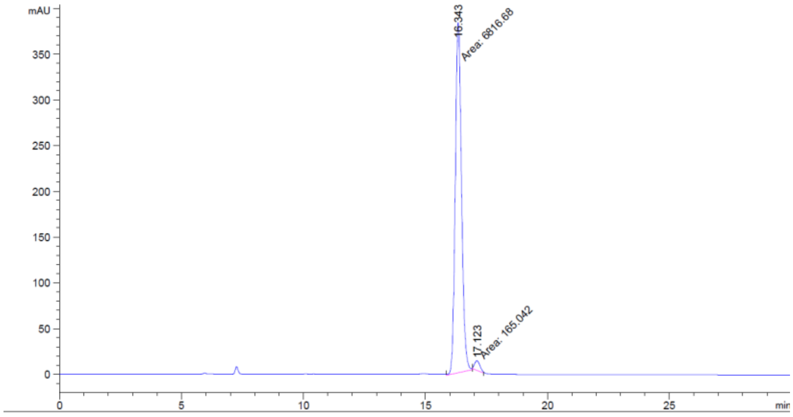


```
==================================================================
                      Area Percent Report
==================================================================

3orted By              :      Signal
4ultiplier             :      1.0000
)ilution               :      1.0000
Jse Multiplier & Dilution Factor with ISTDs


3ignal 1: VWD1 A, Wavelength=250 nm

?eak RetTime Type  Width     Area      Height     Area
  #   [min]        [min]   [mAU*s]     [mAU]        %
----|-------|----|-------|----------|----------|--------|
   1  16.343 MM    0.2963 6816.67725  383.37057  97.6361
   2  17.123 MM    0.2606  165.04242   10.55658   2.3639
```

(R)-3-Fluoro-*N*-(2-fluoro-2-(quinolin-2-yl)ethyl)-4-methoxyaniline **7j**

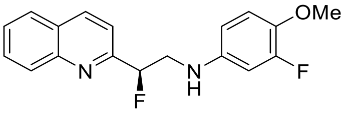

Prepared according to General Procedure A using 2-(1-fluorovinyl)quinoline (17.3 mg, 0.10 mmol, 1.0 equiv), 3-fluoro-4-methoxyaniline (42.3 mg, 0.30 mmol, 3.0 equiv), (*S*)-TRIP catalyst (15.1 mg, 0.02 mmol, 20 mol%), and THF (200 µL, 0.5 M). The reaction mixture was subjected to the purification outlined in General Procedure A (silica gel, EtOAc:petroleum ether, 5:95 to 10:90) to afford the desired product as a pale yellow oil (24.6 mg, 72%).

υ_{max} (film): 3398 (br), 3056, 2948, 2932, 2833, 1519, 1227 cm^{-1}.

^{1}H NMR (500 MHz, CDCl$_3$): δ 8.23 (d, J = 8.5 Hz, 1H), 8.09 (d, J = 8.5 Hz, 1H), 7.84 (d, J = 8.1 Hz, 1H), 7.78 – 7.71 (m, 1H), 7.66 – 7.60 (m, 1H), 7.57 (t, J = 7.4 Hz, 1H), 6.82 (t, J = 9.1 Hz, 1H), 6.52 (dd, J = 13.3, 2.7 Hz, 1H), 6.41 (dd, J = 8.7, 1.2 Hz, 1H), 5.87 (ddd, $^{2}J_{HF}$ = 48.7 Hz, J = 7.2, 3.7 Hz, 1H), 4.12 (s, 1H), 3.94 – 3.82 (m, 1H), 3.80 (s, 3H), 3.74 – 3.62 (m, 1H).

^{13}C NMR (126 MHz, CDCl$_3$): δ 158.0 (d, ^{2}J = 24.6 Hz), 153.7 (d, ^{1}J = 244.2 Hz), 147.5, 142.8 (d, ^{3}J = 9.1 Hz), 140.0 (d, ^{3}J = 11.3 Hz), 137.3, 130.1, 129.4, 127.9, 127.9, 127.0, 117.8 (d, ^{3}J = 6.5 Hz), 116.1 (d, ^{4}J = 3.1 Hz), 108.7 (d, ^{4}J = 3.2 Hz), 102.7 (d, ^{2}J = 22.0 Hz), 93.3 (d, ^{1}J = 174.7 Hz), 57.7, 49.3 (d, ^{2}J = 22.5 Hz).

^{19}F NMR (376 MHz, CDCl$_3$): δ −133.38, −191.48.

HRMS (NSI): exact mass calculated for [M+H]$^{+}$ ($C_{18}H_{17}ON_2F_2$) requires *m/z* 315.1303, found *m/z* 315.1306.

The enantiomeric purity of the product was determined by HPLC analysis: 93:7 e.r. (Chiralcel OJ-H, hexane/*i*-PrOH = 70/30, flow rate 0.5 mL/min, λ = 250 nm), t_r (major) = 92.3 min, t_r (minor) = 97.7 min;

$[\alpha]_D^{20}$ = +7.9 (*c* 1.2, $CHCl_3$).

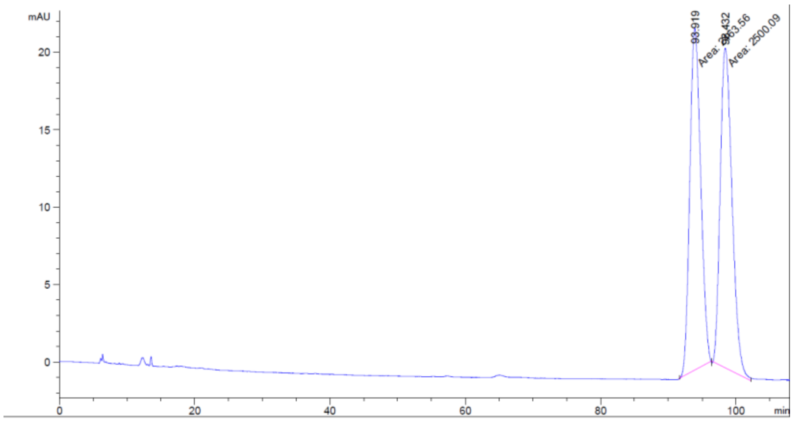


```
=================================================================
                    Area Percent Report
=================================================================

Sorted By             :      Signal
Multiplier            :      1.0000
Dilution              :      1.0000
Use Multiplier & Dilution Factor with ISTDs


Signal 1: VWD1 A, Wavelength=250 nm

Peak RetTime Type  Width     Area      Height     Area
  #   [min]        [min]   [mAU*s]     [mAU]        %
----|-------|----|-------|----------|----------|--------|
   1  93.919 MM    1.8602 2463.55908   22.07241  49.6320
   2  98.432 MM    2.0162 2500.09351   20.66677  50.3680
```

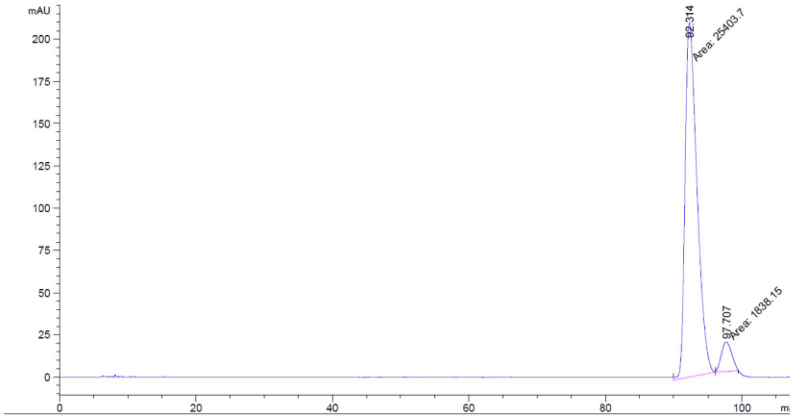


```
=================================================================
                    Area Percent Report
=================================================================

Sorted By             :      Signal
Multiplier            :      1.0000
Dilution              :      1.0000
Use Multiplier & Dilution Factor with ISTDs


Signal 1: VWD1 A, Wavelength=250 nm

Peak RetTime Type  Width     Area      Height     Area
  #   [min]        [min]   [mAU*s]     [mAU]        %
----|-------|----|-------|----------|----------|--------|
   1  92.314 MM    2.0188 2.54037e4  209.72574  93.2525
   2  97.707 MM    1.7613 1838.15466  17.39389   6.7475
```

(R)-*N*-(2-Fluoro-2-(quinolin-2-yl)ethyl)benzo[*b*]thiophen-5-amine **7k**

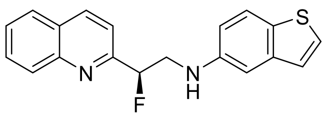

Prepared according to General Procedure A using 2-(1-fluorovinyl)quinoline (17.3 mg, 0.10 mmol, 1.0 equiv), benzo[*b*]thiophen-5-amine (44.7 mg, 0.30 mmol, 3.0 equiv), (*S*)-TRIP catalyst (15.1 mg, 0.02 mmol, 20 mol%), and THF (200 µL, 0.5 M) at −20 °C for 5 d. The reaction mixture was subjected to the purification outlined in General Procedure A (silica gel, EtOAc:petroleum ether, 5:95 to 10:90) to afford the desired product as a pale yellow solid (18.7 mg, 58%).

υ_{max} (film): 3389 (br), 3058, 3021, 2924, 2848, 1599, 1504, 1437, 1065, 831, 749, 691 cm^{-1}.

^{1}H NMR (500 MHz, MeOD): δ 8.30 (d, *J* = 8.5 Hz, 1H), 8.07 (d, *J* = 8.5 Hz, 1H), 7.88 (d, *J* = 8.1 Hz, 1H), 7.82 – 7.73 (m, 1H), 7.65 (d, *J* = 8.4 Hz, 1H), 7.57 (t, *J* = 7.2 Hz, 1H), 7.54 (d, *J* = 8.7 Hz, 1H), 7.36 (d, *J* = 5.4 Hz, 1H), 7.08 (d, *J* = 5.7 Hz, 2H), 6.81 (dd, *J* = 8.7, 2.2 Hz, 1H), 5.86 (ddd, $^{2}J_{HF}$ = 48.7 Hz, *J* = 7.5, 3.4 Hz, 1H), 3.86 (ddd, $^{3}J_{HF}$ = 27.5 Hz, $^{2}J_{HH}$ = 14.6 Hz, *J* = 3.4 Hz, 1H), 3.71 (ddd, $^{3}J_{HF}$ = 20.2 Hz, $^{2}J_{HH}$ = 14.6 Hz, *J* = 7.5 Hz, 1H). (N-H proton not observed)

^{13}C NMR (126 MHz, MeOD): δ 159.7 (d, ^{2}J = 23.9 Hz), 148.4, 146.9, 142.4, 138.9, 131.3, 130.7, 129.3, 129.3, 129.1, 128.1, 127.5, 124.4, 123.5, 119.3 (d, ^{3}J = 6.0 Hz), 115.3, 106.2, 94.4 (d, ^{1}J = 174.9 Hz), 50.1 (d, ^{2}J = 23.2 Hz).

^{19}F NMR (376 MHz, MeOD): δ −190.65.

HRMS (NSI): exact mass calculated for [M+H]$^{+}$ ($C_{19}H_{16}N_2FS$) requires *m/z* 323.1013, found *m/z* 323.1016.

The enantiomeric purity of the product was determined by HPLC analysis: 94:6 e.r. (Chiralpak IA, hexane/*i*-PrOH = 50/50, flow rate 0.5 mL/min, λ = 250 nm), t_r (major) = 14.9 min, t_r (minor) = 15.8 min;

$[\alpha]_D^{20}$ = +9.0 (*c* 0.94, $CHCl_3$).

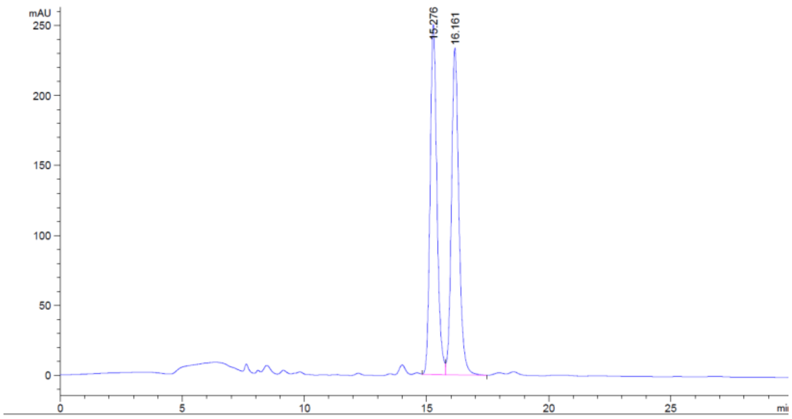

Area Percent Report

Sorted By : Signal
Multiplier : 1.0000
Dilution : 1.0000
Use Multiplier & Dilution Factor with ISTDs

Signal 1: VWD1 A, Wavelength=250 nm

Peak #	RetTime [min]	Type	Width [min]	Area [mAU*s]	Height [mAU]	Area %
1	15.276	VV	0.2986	4870.37109	250.01653	49.5778
2	16.161	VB	0.3233	4953.32080	233.38797	50.4222

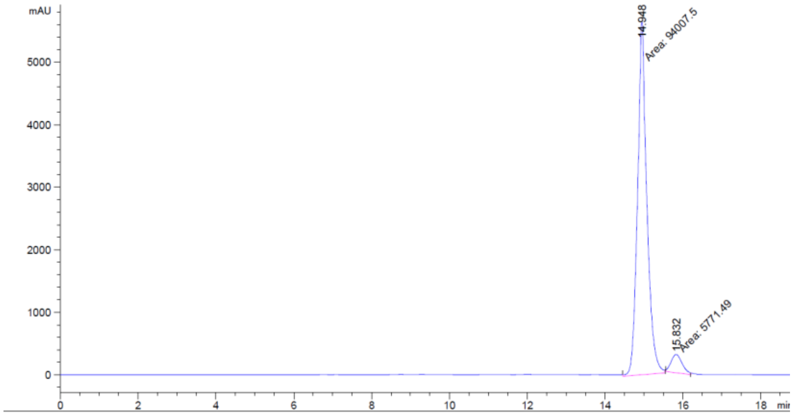

Area Percent Report

Sorted By : Signal
Multiplier : 1.0000
Dilution : 1.0000
Use Multiplier & Dilution Factor with ISTDs

Signal 1: VWD1 A, Wavelength=250 nm

Peak #	RetTime [min]	Type	Width [min]	Area [mAU*s]	Height [mAU]	Area %
1	14.948	MM	0.2780	9.40075e4	5635.32910	94.2157
2	15.832	MM	0.3288	5771.49170	292.51791	5.7843

(R)-*N*-(2-Fluoro-2-(quinolin-2-yl)ethyl)-2,3-dihydrobenzofuran-5-amine **7l**

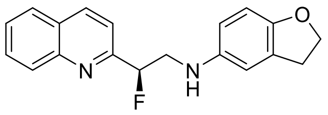

Prepared according to General Procedure A using 2-(1-fluorovinyl)quinoline (17.3 mg, 0.10 mmol, 1.0 equiv), 2,3-dihydrobenzofuran-5-amine (40.5 mg, 0.30 mmol, 3.0 equiv), (*S*)-TRIP catalyst (15.1 mg,

0.02 mmol, 20 mol%), and THF (200 µL, 0.5 M) at −20 °C for 5 d. The reaction mixture was subjected to the purification outlined in General Procedure A (silica gel, EtOAc:petroleum ether, 5:95 to 10:100) to afford the desired product as a pale yellow oil (11.4 mg, 37%).

υ_{max} (film): 3376 (br), 3051, 2954, 2921, 2887, 2850, 1491, 1216 cm^{-1}.

^{1}H NMR (400 MHz, CDCl$_3$): δ 8.22 (d, *J* = 8.5 Hz, 1H), 8.09 (dd, *J* = 8.6, 1.1 Hz, 1H), 7.84 (dd, *J* = 8.2, 1.4 Hz, 1H), 7.75 (ddd, *J* = 8.4, 6.8, 1.5 Hz, 1H), 7.63 (dd, *J* = 8.5, 1.5 Hz, 1H), 7.57 (ddd, *J* = 8.1, 6.8, 1.2 Hz, 1H), 6.70 – 6.62 (m, 2H), 6.51 (dd, *J* = 8.5, 2.5 Hz, 1H), 5.89 (ddd, $^{2}J_{HF}$ = 48.7 Hz, *J* = 7.4, 3.7 Hz, 1H), 4.55 – 4.39 (m, 2H), 3.86 (ddd, $^{3}J_{HF}$ = 26.5 Hz, $^{2}J_{HH}$ = 13.9 Hz, *J* = 3.7 Hz, 1H), 3.67 (ddd, $^{3}J_{HF}$ = 19.6 Hz, $^{2}J_{HH}$ 13.9 Hz, *J* = 7.4 Hz, 1H), 3.19 – 3.01 (m, 2H).

^{13}C NMR (101 MHz, CDCl$_3$): δ 158.3 (d, ^{2}J = 24.8 Hz), 153.2, 147.5, 141.9, 137.2, 130.0, 129.3, 128.0, 127.8, 126.9, 118.0, 117.9, 113.6, 111.5, 109.5, 93.4 (d, ^{1}J = 174.2 Hz), 71.1, 50.2 (d, ^{2}J = 22.2 Hz), 30.4.

^{19}F NMR (376 MHz, CDCl$_3$): δ −191.64.

HRMS (NSI): exact mass calculated for [M+H]$^+$ ($C_{19}H_{18}ON_2F$) requires *m/z* 309.1398, found *m/z* 309.1401.

The enantiomeric purity of the product was determined by HPLC analysis: 89:11 e.r. (Chiralcel OJ-H, hexane/*i*-PrOH = 50/50, flow rate 0.5 mL/min, λ = 250 nm), t_r (major) = 55.0 min, t_r (minor) = 60.2 min;

$[\alpha]_D^{20}$ = +2.5 (*c* 0.57, CHCl$_3$).

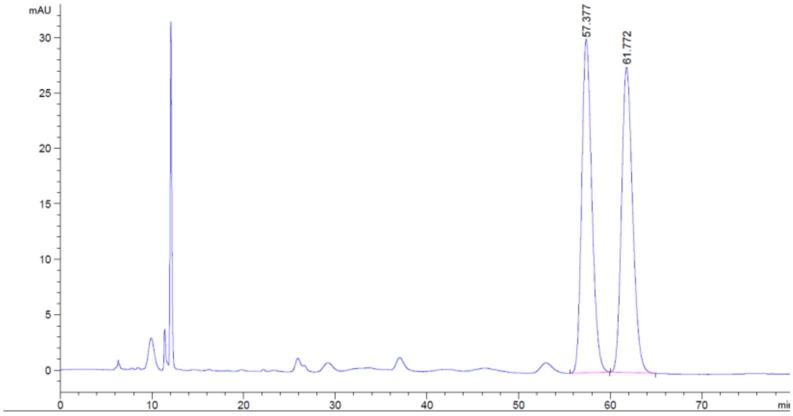


```
=====================================================================
                         Area Percent Report
=====================================================================

Sorted By             :      Signal
Multiplier            :      1.0000
Dilution              :      1.0000
Use Multiplier & Dilution Factor with ISTDs


Signal 1: VWD1 A, Wavelength=250 nm

Peak RetTime Type  Width     Area      Height     Area
  #   [min]        [min]   [mAU*s]     [mAU]        %
----|-------|----|-------|----------|----------|--------|
   1  57.377 BB    1.1785 2316.01807   30.10777  50.0123
   2  61.772 BB    1.2849 2314.87866   27.54219  49.9877
```

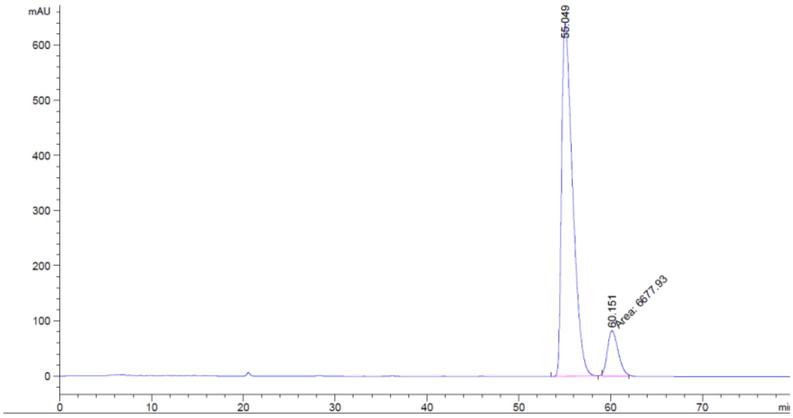


```
=====================================================================
                         Area Percent Report
=====================================================================

Sorted By             :      Signal
Multiplier            :      1.0000
Dilution              :      1.0000
Use Multiplier & Dilution Factor with ISTDs


Signal 1: VWD1 A, Wavelength=250 nm
```

Peak #	RetTime [min]	Type	Width [min]	Area [mAU*s]	Height [mAU]	Area %
1	55.049	BB	1.2743	5.37853e4	640.15222	88.9554
2	60.151	MM	1.3544	6677.92578	82.17638	11.0446

(R)-2-(1-Fluoro-2-(indolin-1-yl)ethyl)quinoline **7m**

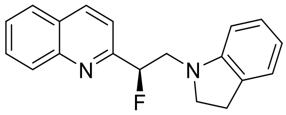

Prepared according to General Procedure B using 2-(1-fluorovinyl)quinoline (17.3 mg, 0.10 mmol, 1.0 equiv), indoline (35.7 mg, 0.30 mmol, 3.0 equiv), (*S*)-TRIP catalyst (15.1 mg, 0.02 mmol, 20 mol%), and CPME (200 µL, 0.5 M). The reaction mixture was subjected to the purification outlined in General Procedure B (silica gel, EtOAc:petroleum ether, 5:95 to 10:100) to afford the desired product as a pale yellow solid (26.9 mg, 92%).

υ_{max} (film): 3048, 3024, 2949, 2922, 2845, 1607, 1599, 1489, 1244, 742 cm^{-1}.

^{1}H NMR (400 MHz, CDCl$_3$): δ 8.22 (d, J = 8.5 Hz, 1H), 8.12 (d, J = 8.4 Hz, 1H), 7.84 (dd, J = 8.2, 0.9 Hz, 1H), 7.75 (ddd, J = 8.4, 6.9, 1.4 Hz, 1H), 7.67 (dd, J = 8.5, 1.4 Hz, 1H), 7.57 (ddd, J = 8.1, 6.9, 1.1 Hz, 1H), 7.09 – 7.00 (m, 2H), 6.65 (td, J = 7.4, 0.8 Hz, 1H), 6.56 (d, J = 7.8 Hz, 1H), 5.98 (ddd, $^{2}J_{HF}$ = 48.7 Hz, J = 6.8, 2.9 Hz, 1H), 3.86 (ddd, $^{3}J_{HF}$ = 28.6 Hz, $^{2}J_{HH}$ = 15.1 Hz, J = 2.9 Hz, 1H), 3.72 (ddd, $^{3}J_{HF}$ = 24.0 Hz, $^{2}J_{HH}$ = 15.1 Hz, J = 6.8 Hz, 1H), 3.56 (q, J = 8.3 Hz, 1H), 3.48 (qd, J = 8.7, 0.8 Hz, 1H), 2.98 (t, J = 8.4 Hz, 2H).

^{13}C NMR (101 MHz, CDCl$_3$): δ 158.5 (d, ^{2}J = 24.8 Hz), 152.3, 147.6, 137.1, 130.0, 129.6, 129.4, 127.9, 127.8, 127.5, 126.8, 124.5, 118.1 (d, ^{3}J = 6.2 Hz), 117.8, 106.9, 94.7 (d, ^{1}J = 176.7 Hz), 54.9, 54.8 (d, ^{2}J = 20.9 Hz), 28.9.

^{19}F NMR (376 MHz, CDCl$_3$): δ −187.59.

HRMS (NSI): exact mass calculated for $[M+H]^+$ ($C_{19}H_{18}N_2F$) requires *m/z* 293.1449, found *m/z* 293.1450.

The enantiomeric purity of the product was determined by HPLC analysis: 92:8 e.r. (Chiralcel OJ-H, hexane/*i*-PrOH = 50/50, flow rate 0.5 mL/min, λ = 250 nm), t_r (major) = 24.9 min, t_r (minor) = 24.0 min;

$[\alpha]_D^{20}$ = +21.7 (*c* 1.35, $CHCl_3$).

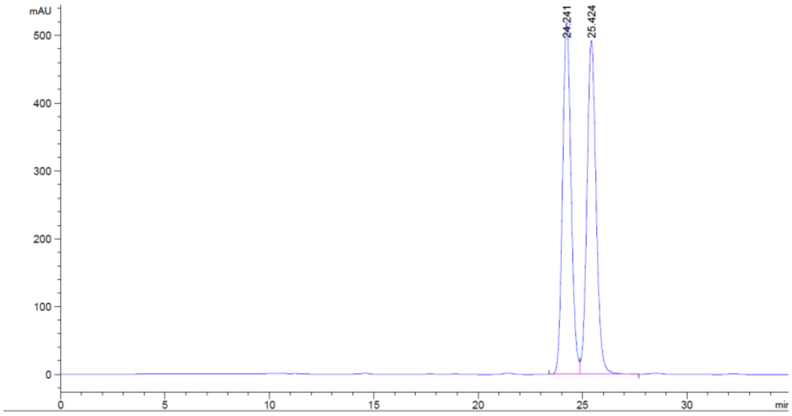

Area Percent Report

3orted By : Signal
4ultiplier : 1.0000
Jilution : 1.0000
Jse Multiplier & Dilution Factor with ISTDs

3ignal 1: VWD1 A, Wavelength=250 nm

?eak #	RetTime [min]	Type	Width [min]	Area [mAU*s]	Height [mAU]	Area %
1	24.241	BV	0.4392	1.47304e4	518.54968	49.4921
2	25.424	VB	0.4713	1.50327e4	492.28079	50.5079

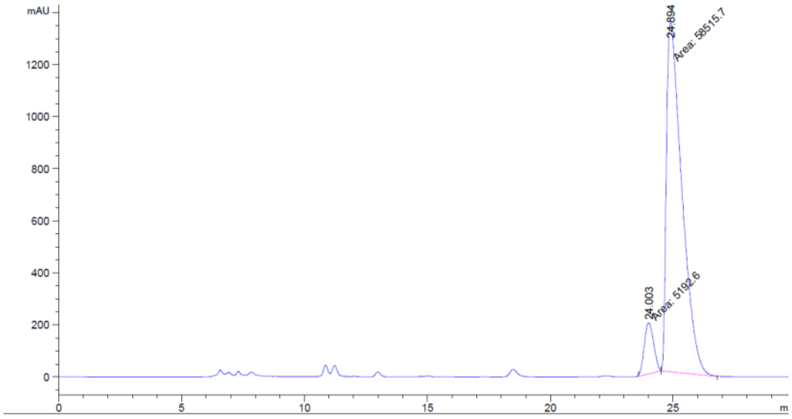

Area Percent Report

iorted By : Signal
Iultiplier : 1.0000
)ilution : 1.0000
Jse Multiplier & Dilution Factor with ISTDs

iignal 1: VWD1 A, Wavelength=250 nm

'eak #	RetTime [min]	Type	Width [min]	Area [mAU*s]	Height [mAU]	Area %
1	24.003	MM	0.4411	5192.60156	196.21417	8.1506
2	24.894	MM	0.7268	5.85157e4	1341.82629	91.8494

(R)-*N*-(2-Fluoro-2-(3-methylquinolin-2-yl)ethyl)aniline **7n**

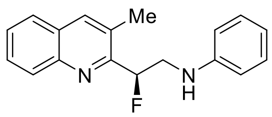

Prepared according to General Procedure B using 3-methyl-2-(1-fluorovinyl)quinoline (18.8 mg. 0.10 mmol, 1.0 equiv), aniline (27 µL, 0.30 mmol, 3.0 equiv), (*S*)-TRIP catalyst (15.1 mg, 0.02 mmol, 20 mol%), and CPME (200 µL, 0.5 M) at −10 °C for 3 d. The reaction mixture was subjected to the purification outlined the General Procedure B (silica gel, EtOAc:petroleum ether, 5:95 to 1:100) to afford the desired product as a pale yellow solid (13.8 mg, 50%).

υ_{max} (film): 1603, 1506, 1497, 1447, 1325, 1261, 750, 692 cm^{-1}.

^{1}H NMR (500 MHz, $CDCl_3$): δ 8.12 (d, *J* = 8.4 Hz, 1H), 7.96 (s, 1H), 7.76 (d, *J* = 8.1 Hz, 1H), 7.69 (t, *J* = 7.6 Hz, 1H), 7.55 (t, *J* = 7.5 Hz, 1H), 7.21 (t, *J* = 7.8 Hz, 2H), 6.74 (dd, *J* = 10.9, 7.9 Hz, 3H), 6.00 (ddd, $^{2}J_{HF}$ = 48.1 Hz, *J* = 7.2, 4.9 Hz, 1H), 4.41 (s, 1H), 4.12 – 3.91 (m, 2H), 2.58 (s, 3H).

^{13}C NMR (126 MHz, $CDCl_3$): δ 155.2 (d, ^{3}J = 18.1 Hz), 147.7, 146.0, 137.3, 129.9, 129.5, 129.4, 129.0, 128.2, 127.2, 126.8, 117.9, 113.2, 90.2 (d, ^{1}J = 172.8 Hz), 46.2 (d, ^{2}J = 24.6 Hz), 18.6 (d, ^{4}J = 3.3 Hz).

^{19}F NMR (376 MHz, $CDCl_3$): δ −183.72.

HRMS (ESI): exact mass calculated for $[M+H]^+$ ($C_{18}H_{18}FN_2$) requires *m/z* 281.1449, found *m/z* 281.1441.

The enantiomeric purity of the product was determined by HPLC analysis: 91:9 e.r. (Chiralcel OJ-H, hexane/*i*-PrOH = 50/50, flow rate 1.0 mL/min, λ = 250 nm), t_r (major) = 28.8 min, t_r (minor) = 45.1 min;

$[\alpha]_D^{20}$ = −71.9 (*c* 0.21, in $CHCl_3$).

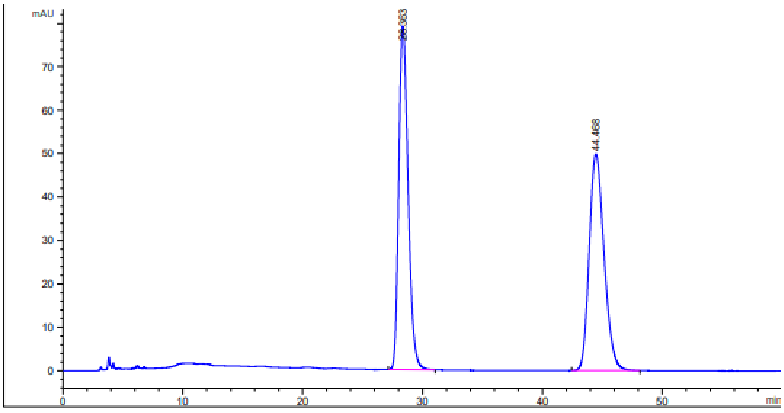

Area Percent Report

Sorted By : Signal
Multiplier : 1.0000
Dilution : 1.0000
Use Multiplier & Dilution Factor with ISTDs

Signal 1: VWD1 A, Wavelength=250 nm

Peak #	RetTime [min]	Type	Width [min]	Area [mAU*s]	Height [mAU]	Area %
1	28.363	BB	0.8188	4269.78467	79.12798	49.9060
2	44.468	BB	1.2874	4285.86523	49.82888	50.0940

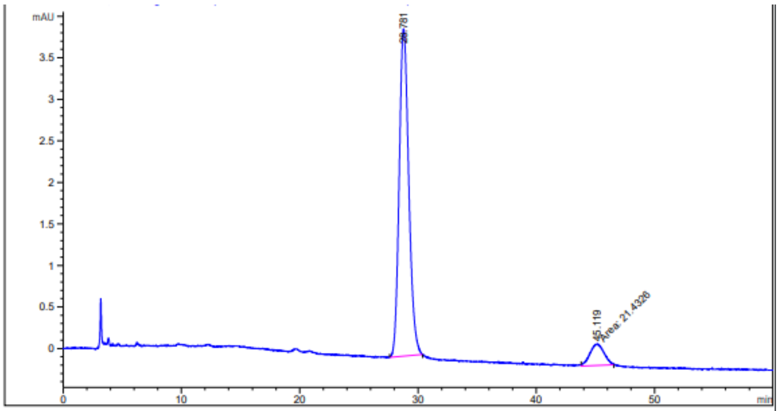

Area Percent Report

Sorted By : Signal
Multiplier : 1.0000
Dilution : 1.0000
Use Multiplier & Dilution Factor with ISTDs

Signal 1: VWD1 A, Wavelength=250 nm

Peak #	RetTime [min]	Type	Width [min]	Area [mAU*s]	Height [mAU]	Area %
1	28.781	BB	0.8186	221.00998	3.94218	91.1597
2	45.119	MM	1.3857	21.43265	2.57792e-1	8.8403

(R)-*N*-(2-Fluoro-2-(6-fluoroquinolin-2-yl)ethyl)aniline **7o**

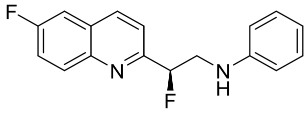

Prepared according to General Procedure B using 6-fluoro-2-(1-fluorovinyl)quinoline (19.1 mg, 0.10 mmol, 1.0 equiv), aniline (27 µL, 0.30 mmol, 3.0 equiv), (*S*)-TRIP catalyst (15.1 mg, 0.02 mmol, 20

mol%), and CPME (200 μL, 0.5 M) at −10 °C for 3 d. The reaction mixture was subjected to the purification outlined in General Procedure B (silica gel, EtOAc:petroleum ether, 5:95 to 10:100) to afford the desired product as a pale brown oil (18.9 mg, 67%).

υ_{max} (film): 1603, 1506, 1323, 1260, 1231, 1142, 1061, 872, 833, 750, 692 cm^{-1}.

^{1}H NMR (400 MHz, $CDCl_3$): δ 8.17 (d, J = 8.6 Hz, 1H), 8.10 (dd, J = 9.2, 5.3 Hz, 1H), 7.65 (d, J = 8.5 Hz, 1H), 7.52 (td, J = 8.7, 2.8 Hz, 1H), 7.46 (dd, J = 8.8, 2.8 Hz, 1H), 7.19 (t, J = 7.8 Hz, 2H), 6.78 – 6.70 (m, 3H), 5.88 (ddd, $^{2}J_{HF}$ = 48.6 Hz, J = 7.3, 3.6 Hz, 1H), 3.94 (ddd, $^{3}J_{HF}$ = 26.4 Hz, $^{2}J_{HH}$ = 14.1 Hz, J = 3.7 Hz, 1H), 3.72 (ddd, $^{3}J_{HF}$ = 20.0 Hz, $^{2}J_{HH}$ = 14.1 Hz, J = 7.3 Hz, 1H).

^{13}C NMR (101 MHz, $CDCl_3$): δ 160.7 (d, ^{1}J = 248.7 Hz), 157.6 (d, ^{4}J = 2.5 Hz), 157.4 (d, ^{4}J = 2.9 Hz), 147.6, 144.6, 136.6 (d, ^{3}J = 5.5 Hz), 131.9 (d, ^{3}J = 9.2 Hz), 129.5, 120.4 (d, ^{2}J = 25.8 Hz), 118.7 (d, ^{3}J = 6.4 Hz), 118.2, 113.4, 110.8 (d, ^{2}J = 21.7 Hz), 93.2 (d, ^{1}J = 174.5 Hz), 48.5 (d, ^{2}J = 22.4 Hz).

^{19}F NMR (376 MHz, $CDCl_3$): δ −112.80, −191.35.

HRMS (ESI): exact mass calculated for $[M+H]^+$ ($C_{17}H_{15}F_2N_2$) requires *m/z* 285.1198, found *m/z* 285.1190.

The enantiomeric purity of the product was determined by HPLC analysis: 94:6 e.r. (Chiralpak IA, hexane/*i*-PrOH = 50/50, flow rate 0.5 mL/min, λ = 250 nm), t_r (major) = 11.4 min, t_r (minor) = 12.1 min;

$[\alpha]_D^{20}$ = +40.0 (*c* 0.10, $CHCl_3$).

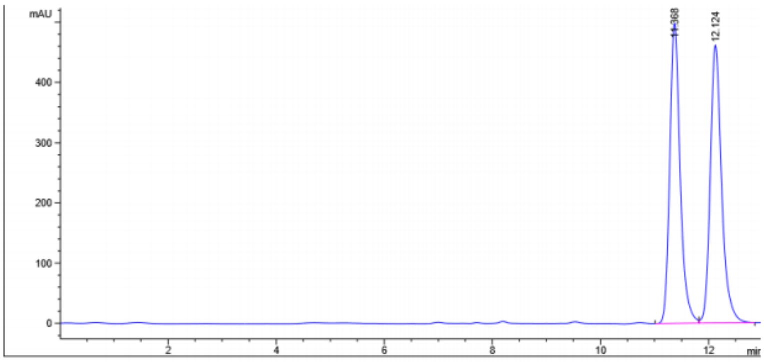


```
=====================================================================
                        Area Percent Report
=====================================================================

Sorted By             :      Signal
Multiplier:                  :      1.0000
Dilution:                    :      1.0000
Use Multiplier & Dilution Factor with ISTDs

Signal 1: DAD1 A, Sig=254,4 Ref=360,100

Peak RetTime Type  Width      Area      Height     Area
  #   [min]        [min]    [mAU*s]     [mAU]        %
----|-------|----|-------|----------|----------|--------|
   1  11.368 VV    0.2018 6660.14502  498.86203  49.7094
   2  12.124 VB    0.2223 6738.01025  461.47482  50.2906
```

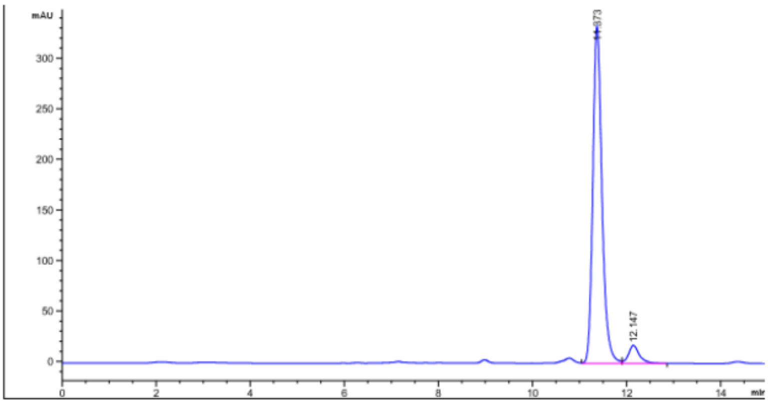


```
=====================================================================
                         Area Percent Report
=====================================================================

Sorted By             :      Signal
Multiplier            :      1.0000
Dilution              :      1.0000
Use Multiplier & Dilution Factor with ISTDs


Signal 1: VWD1 A, Wavelength=250 nm

Peak RetTime Type  Width     Area      Height     Area
  #   [min]        [min]   [mAU*s]     [mAU]        %
----|-------|----|-------|----------|----------|--------|
   1  11.373 VV    0.2056 4506.97461  333.68134  94.0452
   2  12.147 VB    0.2389  285.37711   17.71371   5.9548
```

(R)-*N*-(2-(6-Bromoquinolin-2-yl)-2-fluoroethyl)aniline **7p**

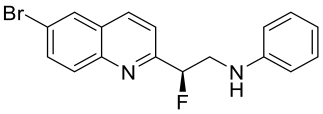

Prepared according to General Procedure B using 6-bromo-2-(1-fluorovinyl)quinoline (25.2 mg, 0.10 mmol, 1.0 equiv), aniline (27 µL, 0.30 mmol, 3.0 equiv), (*S*)-TRIP catalyst (15.1 mg, 0.02 mmol, 20 mol%), and CPME (200 µL, 0.5 M) at −10 °C for 3 d. The reaction mixture was subjected to the purification outlined in General Procedure B (silica gel, EtOAc:petroleum ether, 5:95 to 10:100) to afford the desired product as a pale brown oil (33.1 mg, 96%).

υ_{max} (film): 1603, 1508, 1491, 1059, 831, 750, 692 cm^{-1}.

^{1}H NMR (400 MHz, CDCl$_3$): δ 8.13 (d, *J* = 8.5 Hz, 1H), 8.00 (d, *J* = 2.2 Hz, 1H), 7.96 (d, *J* = 9.0 Hz, 1H), 7.81 (dd, *J* = 9.0, 2.2 Hz, 1H), 7.65 (d, *J* = 8.5 Hz, 1H), 7.19 (t, *J* = 7.8 Hz, 2H), 6.78 – 6.69 (m, 3H), 5.87 (ddd, $^2J_{HF}$ = 48.7 Hz, *J* = 7.3, 3.6 Hz, 1H), 4.25 (s, 1H), 3.95 (ddd, $^3J_{HF}$ = 26.4 Hz, $^2J_{HH}$ = 14.1 Hz, *J* = 3.6 Hz, 1H), 3.71 (ddd, $^3J_{HF}$ = 20.9 Hz, $^2J_{HH}$ = 14.1 Hz, *J* = 7.3 Hz, 1H).

^{13}C NMR (101 MHz, CDCl$_3$): δ 158.6 (d, ^2J = 24.8 Hz), 147.5, 146.1 (d, ^4J = 2.4 Hz), 136.2, 133.5, 131.1, 129.9, 129.5, 128.9, 120.8, 118.8 (d, ^3J = 6.6 Hz), 118.3, 113.4, 93.2 (d, ^1J = 174.8 Hz), 48.5 (d, ^2J = 22.2 Hz).

^{19}F NMR (376 MHz, CDCl$_3$): δ −191.78.

HRMS (ESI): exact mass calculated for [M+H]$^+$ ($C_{17}H_{15}BrFN_2$) requires *m/z* 345.0397, found *m/z* 345.0393.

The enantiomeric purity of the product was determined by HPLC analysis: 93:7 e.r. (Chiralpak IA, hexane/*i*-PrOH = 50/50, flow rate 0.5 mL/min, λ = 250 nm), t_r (major) = 12.7 min, t_r (minor) = 13.4 min;

$[\alpha]_D^{20}$ = +11.8 (*c* 1.15, $CHCl_3$).

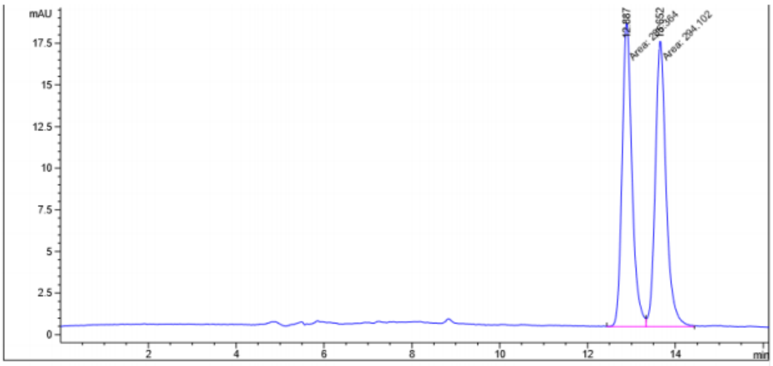

Area Percent Report

Sorted By : Signal
Multiplier: : 1.0000
Dilution: : 1.0000
Use Multiplier & Dilution Factor with ISTDs

Signal 1: DAD1 A, Sig=254,4 Ref=360,100

Peak #	RetTime [min]	Type	Width [min]	Area [mAU*s]	Height [mAU]	Area %
1	12.887	MF	0.2617	286.36404	18.24028	49.3335
2	13.652	FM	0.2859	294.10202	17.14728	50.6665

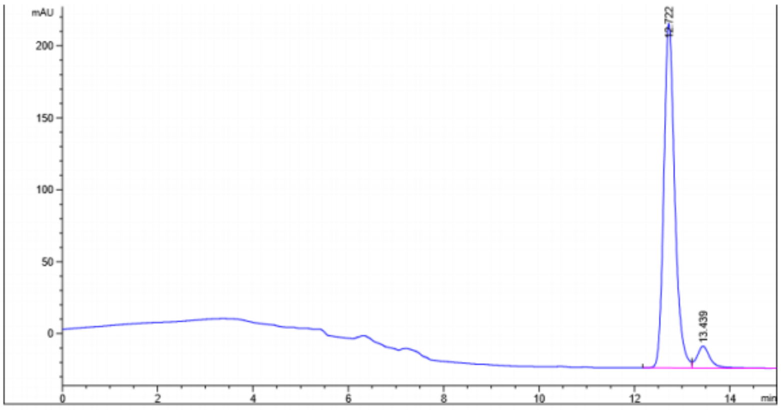

Area Percent Report

Sorted By : Signal
Multiplier : 1.0000
Dilution : 1.0000
Use Multiplier & Dilution Factor with ISTDs

Signal 1: VWD1 A, Wavelength=250 nm

Peak #	RetTime [min]	Type	Width [min]	Area [mAU*s]	Height [mAU]	Area %
1	12.722	BV	0.2429	3816.47534	239.22580	92.9299
2	13.439	VBA	0.2828	290.35831	15.18612	7.0701

(R)-*N*-(2-(4-Bromoquinolin-2-yl)-2-fluoroethyl)aniline **7q**

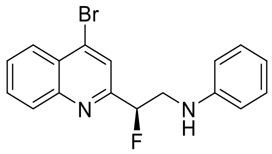

Prepared according to General Procedure A using 4-bromo-2-(1-fluorovinyl)quinoline (25.2 mg, 0.10 mmol, 1.0 equiv), aniline (27 µL, 0.30 mmol, 3.0 equiv), (*S*)-TRIP catalyst (15.1 mg, 0.02 mmol, 20 mol%), and THF (200 µL, 0.5 M). The reaction mixture was subjected to the purification outlined in General Procedure A (silica gel, EtOAc:petroleum ether, 5:95 to 10:90) to afford the desired product as a pale yellow solid (33.1 mg, 96%).

υ_{max} (film): 1603, 1508, 1491, 1319, 1261, 1186, 1059, 880, 831, 750, 692 cm^{-1}.

^{1}H NMR (500 MHz, $CDCl_3$): δ 8.20 (dd, *J* = 8.4, 1.4 Hz, 1H), 8.09 (d, *J* = 8.4 Hz, 1H), 7.94 (d, *J* = 1.5 Hz, 1H), 7.80 (ddd, *J* = 8.4, 6.9, 1.4 Hz, 1H), 7.66 (ddd, *J* = 8.3, 6.9, 1.2 Hz, 1H), 7.19 (t, *J* = 7.7 Hz, 2H), 6.78 – 6.71 (m, 3H), 5.86 (ddd, $^2J_{HF}$ = 48.6 Hz, *J* = 7.3, 3.6 Hz, 1H), 3.95 (ddd, $^3J_{HF}$ = 26.4 Hz, $^2J_{HH}$ = 14.2 Hz, *J* = 3.6 Hz, 1H), 3.78 – 3.66 (m, 1H).

^{13}C NMR (126 MHz, $CDCl_3$): δ 158.0 (d, ^2J = 25.0 Hz), 148.1, 147.5, 135.4, 131.0, 129.8, 129.5, 128.2, 127.4, 127.0, 122.0 (d, ^3J = 7.5 Hz), 118.3, 113.5, 92.7 (d, ^1J = 175.8 Hz), 48.5 (d, ^2J = 21.9 Hz).

^{19}F NMR (470 MHz, $CDCl_3$): δ −192.02.

HRMS (ESI): exact mass calculated for $[M+H]^+$ ($C_{17}H_{15}BrFN_2$) requires *m/z* 345.0397, found *m/z* 345.0394.

The enantiomeric purity of the product was determined by HPLC analysis: 98:2 e.r. (Chiralcel OJ-H, hexane/*i*-PrOH = 50/50, flow rate 1.0 mL/min, λ = 250 nm), t_r (major) = 24.4 min, t_r (minor) = 20.5 min;

$[\alpha]_D^{20}$ = +30.7 (*c* 0.27, $CHCl_3$).

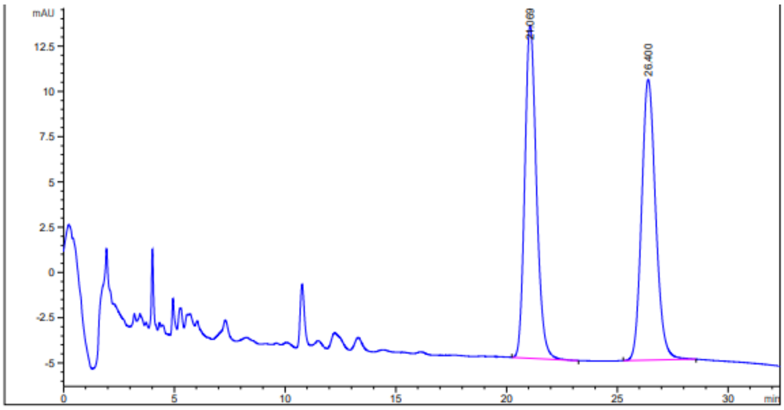


```
=====================================================================
                         Area Percent Report
=====================================================================

Sorted By             :      Signal
Multiplier            :      1.0000
Dilution              :      1.0000
Use Multiplier & Dilution Factor with ISTDs


Signal 1: VWD1 A, Wavelength=250 nm

Peak RetTime Type  Width     Area      Height     Area
  #   [min]        [min]   [mAU*s]     [mAU]        %
----|-------|----|-------|----------|----------|--------|
   1  21.069 BB    0.5653  673.27606   18.39787  49.5390
   2  26.400 BB    0.6788  685.80560   15.51562  50.4610
```

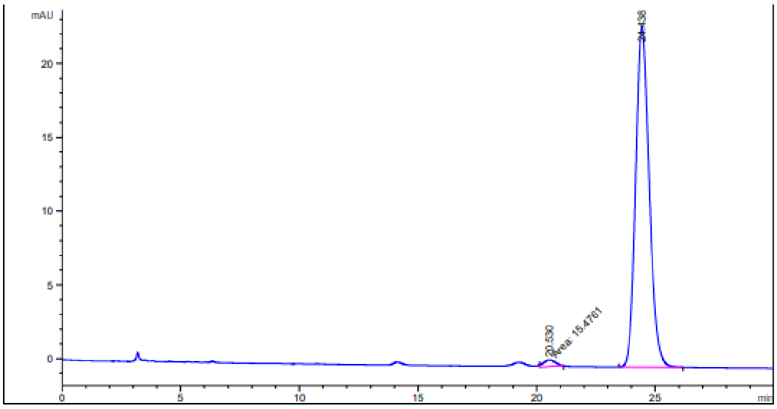


```
=====================================================================
                         Area Percent Report
=====================================================================

Sorted By             :      Signal
Multiplier            :      1.0000
Dilution              :      1.0000
Use Multiplier & Dilution Factor with ISTDs


Signal 1: VWD1 A, Wavelength=250 nm

Peak RetTime Type  Width     Area      Height     Area
  #   [min]        [min]   [mAU*s]     [mAU]        %
----|-------|----|-------|----------|----------|--------|
   1  20.530 MM    0.5559   15.47610 4.63965e-1   1.6550
   2  24.438 BB    0.6148  919.65417   23.08324  98.3450
```

(R)-*N*-(2-Fluoro-2-(quinoxalin-2-yl)ethyl)aniline **7r**

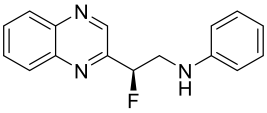

Prepared according to General Procedure B using 2-(1-fluorovinyl)quinoxaline (17.4 mg, 0.10 mmol, 1.0 equiv), aniline (27 µL, 0.30 mmol, 3.0 equiv), (*S*)-TRIP catalyst (15.1 mg, 0.02 mmol, 20 mol%),

and CPME (200 μL, 0.5 M) at −10 °C for 3 d. The reaction mixture was subjected to the purification outlined in General Procedure B (silica gel, EtOAc:petroleum ether, 5:95 to 10:90) to afford the desired product as a pale brown oil (23.3 mg, 87%)

υ_{max} (film): 1603, 1495, 1063, 750, 692 cm^{-1}.

^{1}H NMR (400 MHz, $CDCl_3$): δ 9.07 (s, 1H), 8.17 – 8.08 (m, 2H), 7.81 (qd, J = 7.0, 3.4 Hz, 2H), 7.19 (t, J = 7.9 Hz, 2H), 6.78 – 6.70 (m, 3H), 5.97 (ddd, $^2J_{HF}$ = 48.1 Hz, J = 7.1, 3.5 Hz, 1H), 4.00 (ddd, $^3J_{HF}$ = 26.1 Hz, $^2J_{HH}$ = 14.4 Hz, J = 3.6 Hz, 1H), 3.79 (ddd, $^3J_{HF}$ = 21.3 Hz, $^2J_{HH}$ = 14.4 Hz, J = 7.1 Hz, 1H).

^{13}C NMR (101 MHz, $CDCl_3$): δ 152.4 (d, ^2J = 24.2 Hz), 147.3, 142.8 (d, ^3J = 7.3 Hz), 142.5, 141.5, 130.7, 130.4, 129.6, 129.5, 129.4, 118.5, 113.5, 92.3 (d, ^1J = 174.1 Hz), 48.2 (d, ^2J = 22.0 Hz).

^{19}F NMR (376 MHz, $CDCl_3$): δ −193.73.

HRMS (ESI): exact mass calculated for $[M+H]^+$ ($C_{16}H_{15}FN_3$) requires *m/z* 268.1245, found *m/z* 268.1243.

The enantiomeric purity of the product was determined by HPLC analysis: 92:8 e.r. (Chiralcel OJ-H, hexane/*i*-PrOH = 50/50, flow rate 0.5 mL/min, λ = 250 nm), t_r (major) = 36.3 min, t_r (minor) = 42.2 min;

$[\alpha]_D^{20}$ = +5.4 (*c* 0.13, $CHCl_3$).

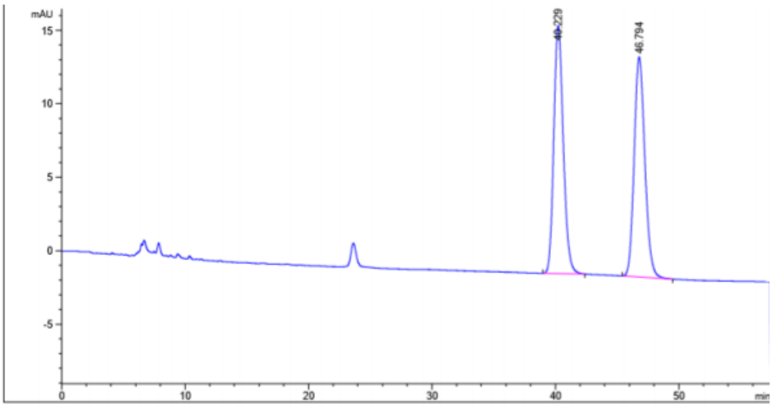


```
=====================================================================
                         Area Percent Report
=====================================================================

Sorted By             :      Signal
Multiplier            :      1.0000
Dilution              :      1.0000
Use Multiplier & Dilution Factor with ISTDs


Signal 1: VWD1 A, Wavelength=250 nm

Peak RetTime Type  Width     Area      Height     Area
  #   [min]        [min]   [mAU*s]     [mAU]        %
----|-------|----|-------|----------|----------|--------|
   1  40.229 BB    0.8238  902.49066   16.86399  49.9839
   2  46.794 BB    0.9099  903.07245   14.98448  50.0161
```

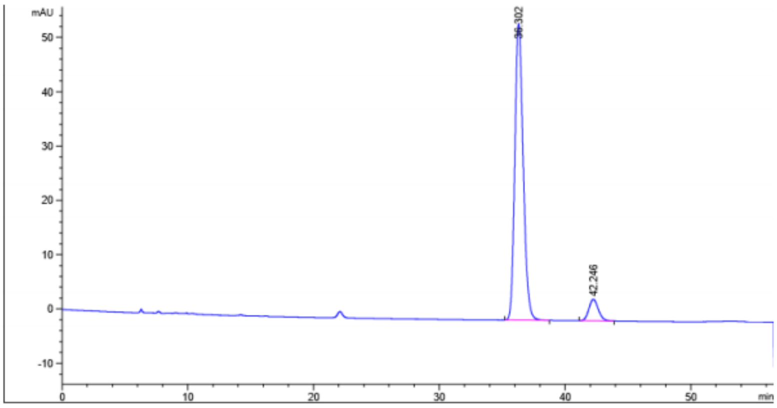


```
=====================================================================
                         Area Percent Report
=====================================================================

Sorted By             :      Signal
Multiplier            :      1.0000
Dilution              :      1.0000
Use Multiplier & Dilution Factor with ISTDs


Signal 1: VWD1 A, Wavelength=250 nm

Peak RetTime Type  Width     Area      Height     Area
  #   [min]        [min]   [mAU*s]     [mAU]        %
----|-------|----|-------|----------|----------|--------|
   1  36.302 BB    0.7149 2526.63403   54.57219  92.4429
   2  42.246 BB    0.7444  206.55081    3.94764   7.5571
```

(*R*)-*N*-(2-(Benzo[*d*]thiazol-2-yl)-2-fluoroethyl)aniline **7s**

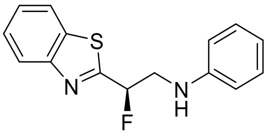

Prepared according to General Procedure B using 2-(1-fluorovinyl)benzo[*d*]thiazole (17.9 mg, 0.10 mmol, 1.0 equiv), aniline (27 µL, 0.30 mmol, 3.0 equiv), (*S*)-TRIP catalyst (15.1 mg, 0.02 mmol, 20 mol%), and CPME (200 µL, 0.5 M) at −10 °C for 3 d. The reaction mixture was subjected to the purification outlined in General Procedure B (silica gel, EtOAc:petroleum ether, 5:95 to 10:90) to afford the desired product as a pale yellow solid (20.3 mg, 75%).

υ_{max} (film): 2357, 1603, 1508, 1317, 1260, 1061, 872, 754, 692 cm^{-1}.

^{1}H NMR (400 MHz, CDCl$_3$): δ 8.07 (d, *J* = 8.2 Hz, 1H), 7.94 (d, *J* = 7.9 Hz, 1H), 7.53 (ddd, *J* = 8.3, 7.2, 1.3 Hz, 1H), 7.44 (ddd, *J* = 8.3, 7.2, 1.2 Hz, 1H), 7.22 (dd, *J* = 8.6, 7.3 Hz, 2H), 6.83 – 6.70 (m, 3H), 6.02 (ddd, $^{2}J_{HF}$ = 48.0 Hz, *J* = 7.2, 3.7 Hz, 1H), 4.02 (ddd, $^{3}J_{HF}$ = 26.1 Hz, $^{2}J_{HH}$ = 14.5 Hz, *J* = 3.7 Hz, 1H), 3.80 (ddd, $^{3}J_{HF}$ = 19.7 Hz, $^{2}J_{HH}$ = 14.5 Hz, *J* = 7.2 Hz, 1H).

^{13}C NMR (101 MHz, CDCl$_3$): δ 168.0 (d, ^{2}J = 28.0 Hz), 153.1, 147.0, 134.9, 129.6, 126.6, 125.7, 123.6, 122.0, 118.7, 113.6, 90.7 (d, ^{1}J = 173.9 Hz), 48.4 (d, ^{2}J = 22.0 Hz).

^{19}F NMR (377 MHz, CDCl$_3$): δ −181.92.

HRMS (ESI): exact mass calculated for [M+H]$^+$ ($C_{15}H_{14}FN_2S$) requires *m/z* 273.0856, found *m/z* 273.0853.

The enantiomeric purity of the product was determined by HPLC analysis: 96:4 e.r. (Chiralcel OJ-H, hexane/*i*-PrOH = 50/50, flow rate 0.5 mL/min, λ = 250 nm), t_r (major) = 39.7 min, t_r (minor) 47.3 = min;

$[\alpha]_D^{20}$ = −18.6 (*c* 0.43, $CHCl_3$).

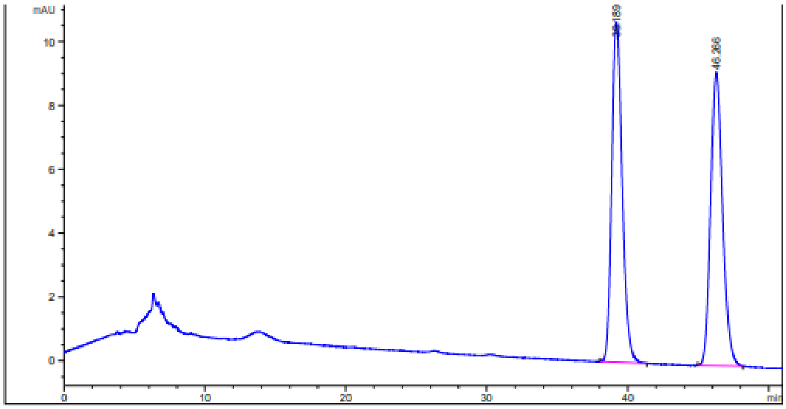

Area Percent Report

Sorted By : Signal
Multiplier : 1.0000
Dilution : 1.0000
Use Multiplier & Dilution Factor with ISTDs

Signal 1: VWD1 A, Wavelength=250 nm

Peak #	RetTime [min]	Type	Width [min]	Area [mAU*s]	Height [mAU]	Area %
1	39.189	BB	0.7733	542.54297	10.65282	50.1830
2	46.266	BB	0.8892	538.58551	9.18738	49.8170

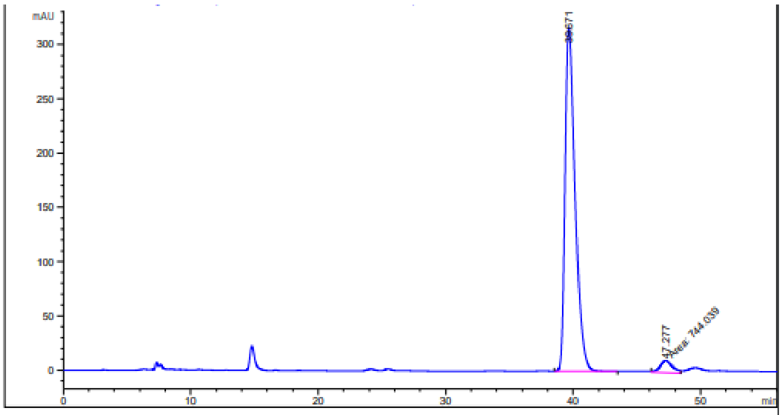

Area Percent Report

Sorted By : Signal
Multiplier : 1.0000
Dilution : 1.0000
Use Multiplier & Dilution Factor with ISTDs

Signal 1: VWD1 A, Wavelength=250 nm

Peak #	RetTime [min]	Type	Width [min]	Area [mAU*s]	Height [mAU]	Area %
1	39.671	BB	0.8407	1.75361e4	316.16418	95.9298
2	47.277	MM	1.1413	744.03857	10.86501	4.0702

Methyl (*R*)-6-(1-fluoro-2-(phenylamino)ethyl)nicotinate **7t**

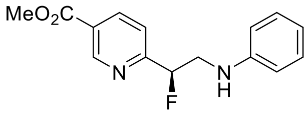

Prepared according to General Procedure B using methyl 6-(1-fluorovinyl)nicotinate (18.1 mg, 0.10 mmol, 1.0 equiv), aniline (27 µL, 0.30 mmol, 3.0 equiv), (*S*)-TRIP catalyst (15.1 mg, 0.02 mmol, 20 mol%), and CPME (200 µL, 0.5 M) at rt for 3 d. The reaction mixture was subjected to the purification outlined in General Procedure B (silica gel, EtOAc:petroleum ether, 5:95 to 10:90) to afford the desired product as a pale brown oil (6.8 mg, 25%).

υ_{max} (film): 1728, 1601, 1506, 1437, 1294, 1119, 750, 694 cm^{-1}.

^{1}H NMR (400 MHz, $CDCl_3$): δ 9.20 – 9.19 (m, 1H), 8.35 (dd, *J* = 8.2, 2.1 Hz, 1H), 7.60 (d, *J* = 8.2 Hz, 1H), 7.25 – 7.13 (m, 2H), 6.80 – 6.65 (m, 3H), 5.78 (ddd, $^{2}J_{HF}$ = 48.4 Hz, *J* = 7.2, 3.3 Hz, 1H), 4.03 – 3.82 (m, 4H), 3.61 (ddd, $^{3}J_{HF}$ = 21.3 Hz, $^{2}J_{HH}$ = 14.3 Hz, *J* = 7.2 Hz, 1H).

^{13}C NMR (101 MHz, $CDCl_3$): δ 165.6, 162.0 (d, ^{2}J = 24.7 Hz), 150.4 (d, ^{4}J = 2.7 Hz), 147.4, 138.2, 129.5, 125.6, 119.8 (d, ^{3}J = 7.4 Hz), 118.3, 113.4, 92.7 (d, ^{1}J = 175.3 Hz), 52.6, 48.4 (d, ^{2}J = 21.6 Hz).

^{19}F NMR (376 MHz, $CDCl_3$): δ −193.31.

HRMS (ESI): exact mass calculated for $[M+H]^+$ ($C_{15}H_{16}FN_2O_2$) requires *m/z* 275.1190, found *m/z* 275.1190.

The enantiomeric purity of the product was determined by HPLC analysis: 88:12 e.r. (Chiralpak IA, hexane/*i*-PrOH = 50/50, flow rate 0.5 mL/min, λ = 250 nm), t_r (major) = 13.0 min, t_r (minor) = 14.8 min;

$[\alpha]_D^{20}$ = +2.95 (*c* 0.88, $CHCl_3$).

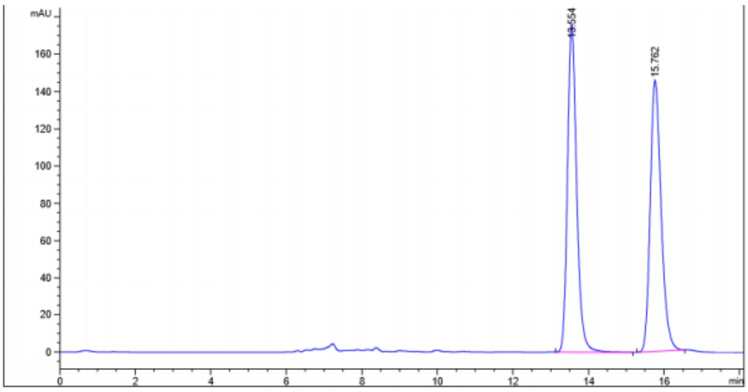


```
=====================================================================
                         Area Percent Report
=====================================================================

Sorted By             :      Signal
Multiplier            :      1.0000
Dilution              :      1.0000
Use Multiplier & Dilution Factor with ISTDs


Signal 1: VWD1 A, Wavelength=250 nm

Peak RetTime Type  Width     Area      Height     Area
  #   [min]        [min]   [mAU*s]     [mAU]       %
----|-------|----|-------|----------|----------|--------|
   1  13.554 BB    0.2588 2987.50024  176.31598  50.5592
   2  15.762 BB    0.3083 2921.41113  145.62823  49.4408
```

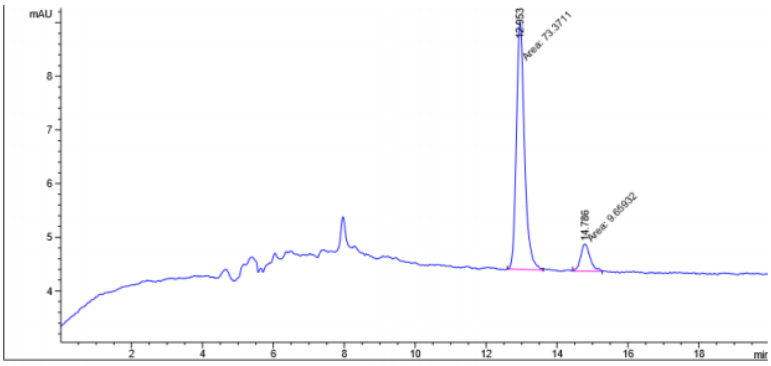


```
=====================================================================
                         Area Percent Report
=====================================================================

Sorted By             :      Signal
Multiplier:                  :      1.0000
Dilution:                    :      1.0000
Use Multiplier & Dilution Factor with ISTDs

Signal 1: DAD1 A, Sig=254,4 Ref=360,100

Peak RetTime Type  Width     Area      Height     Area
  #   [min]        [min]   [mAU*s]     [mAU]        %
----|-------|----|-------|----------|----------|--------|
   1  12.953 MM    0.2664   73.37106    4.59014  88.3665
   2  14.786 MM    0.3154    9.65932 5.10492e-1  11.6335
```

(R)-*N*-(2-Fluoro-2-(isoquinolin-1-yl)ethyl)aniline **7u**

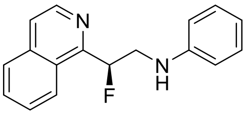

Prepared according to General Procedure B using 1-(1-fluorovinyl)isoquinoline (17.3 mg, 0.10 mmol, 1.0 equiv), aniline (27 μL, 0.30 mmol, 3.0 equiv), (*S*)-TRIP catalyst (15.1 mg, 0.02 mmol, 20 mol%), and CPME (200 μL, 0.5 M) at −10 °C for 3 d. The reaction mixture was subjected to the purification outlined in General Procedure B (silica gel, EtOAc:petroleum ether, 5:95 to 10:90) to afford the desired product as a pale brown oil (16.3 mg, 61%).

υ_{max} (film): 1603, 1504, 829, 748, 692 cm^{-1}.

^{1}H NMR (400 MHz, $CDCl_3$): δ 8.56 (d, *J* = 5.7 Hz, 1H), 8.31 (d, *J* = 8.0 Hz, 1H), 7.88 (d, *J* = 8.2 Hz, 1H), 7.77 – 7.60 (m, 3H), 7.25 – 7.16 (m, 2H), 6.79 – 6.69 (m, 3H), 6.37 (ddd, $^2J_{HF}$ = 48.4, *J* = 6.9, 5.2 Hz, 1H), 4.33 (s, 1H), 4.15 – 3.93 (m, 2H).

^{13}C NMR (101 MHz, $CDCl_3$): δ 155.2 (d, ^2J = 18.6 Hz), 147.6, 141.7, 136.8, 130.4, 129.5, 128.0, 127.7, 126.9, 124.9 (d, ^3J = 4.8 Hz), 122.0, 118.1, 113.4, 90.8 (d, ^1J = 173.5 Hz), 46.9 (d, ^2J = 24.1 Hz).

^{19}F NMR (376 MHz, $CDCl_3$): δ −180.92.

HRMS (ESI): exact mass calculated for $[M+H]^+$ ($C_{17}H_{16}FN_2$) requires *m/z* 267.1292, found *m/z* 267.1290.

The enantiomeric purity of the product was determined by HPLC analysis: 62:38 e.r. (Chiralpak IA, hexane/*i*-PrOH = 50/50, flow rate 0.5 mL/min, λ = 250 nm), t_r (major) = 14.8 min, t_r (minor) = 23.9 min;

$[\alpha]_D^{20}$ = +2.00 (*c* 0.10, $CHCl_3$).

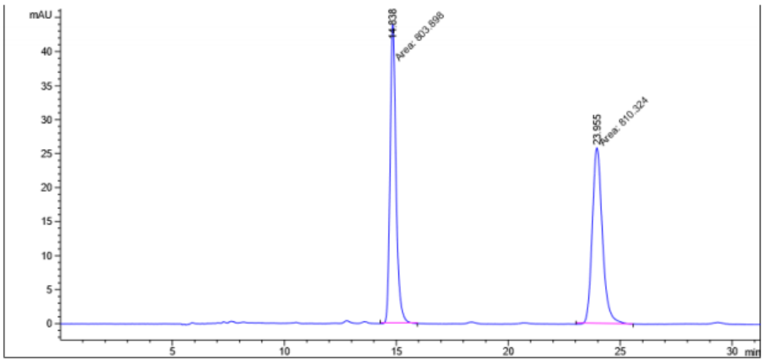


```
=====================================================================
                        Area Percent Report
=====================================================================

Sorted By             :      Signal
Multiplier:                    :      1.0000
Dilution:                      :      1.0000
Use Multiplier & Dilution Factor with ISTDs


Signal 1: DAD1 A, Sig=254,4 Ref=360,100

Peak RetTime Type  Width     Area      Height     Area
  #   [min]        [min]   [mAU*s]     [mAU]        %
----|-------|----|-------|----------|----------|--------|
   1  14.838 MM    0.3049  803.89758   43.94274  49.8009
   2  23.955 MM    0.5242  810.32446   25.76460  50.1991
```

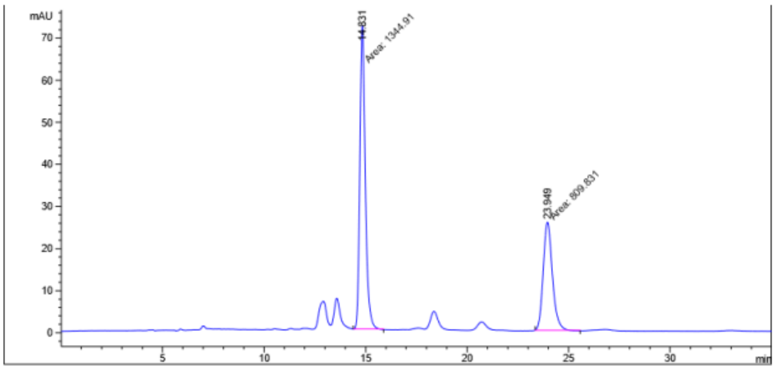


```
=====================================================================
                        Area Percent Report
=====================================================================

Sorted By             :      Signal
Multiplier:                    :      1.0000
Dilution:                      :      1.0000
Use Multiplier & Dilution Factor with ISTDs


Signal 1: DAD1 A, Sig=254,4 Ref=360,100

Peak RetTime Type  Width     Area      Height     Area
  #   [min]        [min]   [mAU*s]     [mAU]        %
----|-------|----|-------|----------|----------|--------|
   1  14.831 MM    0.3106 1344.91199   72.15852  62.4163
   2  23.949 MM    0.5258  809.83136   25.66826  37.5837
```

(*S*)-*N*-(2-Fluoro-2-(quinolin-4-yl)ethyl)aniline **7v**

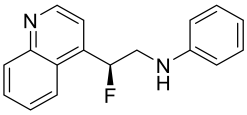

Prepared according to General Procedure B using 4-(1-fluorovinyl)quinoline (17.3 mg, 0.10 mmol, 1.0 equiv), aniline (27 µL, 0.30 mmol, 3.0 equiv), (*S*)-TRIP catalyst (15.1 mg, 0.02 mmol, 20 mol%), and CPME (200 µL, 0.5 M) at rt for 3 d. The reaction mixture was subjected to the purification outlined in General Procedure B (silica gel, EtOAc:petroleum ether, 5:95 to 10:90) to afford the desired product as a pale yellow solid (17.9 mg, 68%).

υ_{max} (film): 2361, 1601, 1509, 1323, 1250, 1069, 752 cm^{-1}.

^{1}H NMR (400 MHz, CDCl$_3$): δ 8.96 (d, J = 4.4 Hz, 1H), 8.20 (dt, J = 8.4, 1.1 Hz, 1H), 7.92 (dt, J = 8.5, 0.9 Hz, 1H), 7.77 (ddd, J = 8.4, 6.9, 1.4 Hz, 1H), 7.62 (ddd, J = 8.3, 6.9, 1.3 Hz, 1H), 7.55 (dd, J = 4.5, 0.8 Hz, 1H), 7.31 – 7.12 (m, 2H), 6.79 (tt, J = 7.3, 1.1 Hz, 1H), 6.76 – 6.65 (m, 2H), 6.50 – 6.25 (m, 1H), 4.17 (s, 1H), 3.81 (ddd, $^{3}J_{HF}$ = 29.9 Hz, $^{2}J_{HH}$ = 14.4 Hz, J = 3.0 Hz, 1H), 3.61 (ddd, $^{3}J_{HF}$ = 18.3 Hz, $^{2}J_{HH}$ = 14.4 Hz, J = 8.1 Hz, 1H).

^{13}C NMR (101 MHz, CDCl$_3$): δ 150.2, 148.2, 147.1, 143.0 (d, ^{2}J = 19.2 Hz), 130.7, 129.60, 129.53, 127.29, 122.41, 118.64, 117.68, 117.57, 113.45, 89.90 (d, ^{1}J = 175.4 Hz), 49.54 (d, ^{2}J = 23.6 Hz).

^{19}F NMR (377 MHz, CDCl$_3$): δ −190.24.

HRMS(ESI): exact mass calculated for $[M+H]^+$ ($C_{17}H_{16}FN_2$) requires *m/z* 267.1292, found *m/z* 267.1285.

The enantiomeric purity of the product was determined by HPLC analysis: 50:50 e.r. (Chiralpak IA, hexane/*i*-PrOH = 50/50, flow rate 0.5 mL/min, λ = 250 nm), t_r (major) = 5.7 min, t_r (minor) = 6.9 min;

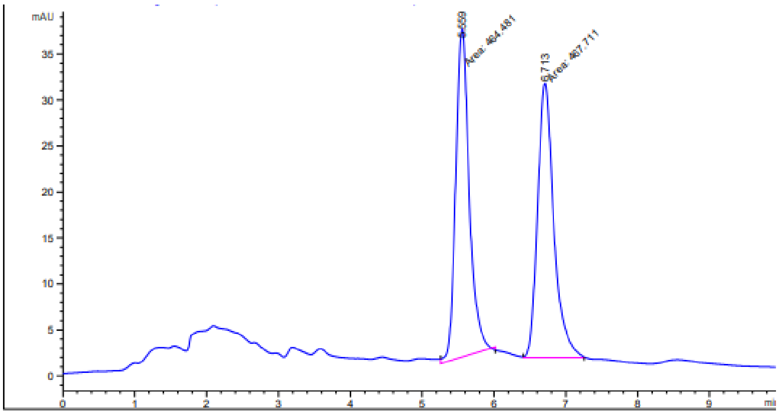


```
====================================================================
                       Area Percent Report
====================================================================

Sorted By             :      Signal
Multiplier            :      1.0000
Dilution              :      1.0000
Use Multiplier & Dilution Factor with ISTDs


Signal 1: VWD1 A, Wavelength=250 nm

Peak RetTime Type  Width     Area      Height     Area
  #   [min]        [min]   [mAU*s]     [mAU]        %
----|-------|----|-------|----------|----------|--------|
   1   5.559 MM    0.2170  464.48099   35.67741  49.8268
   2   6.713 MM    0.2617  467.71078   29.78292  50.1732
```

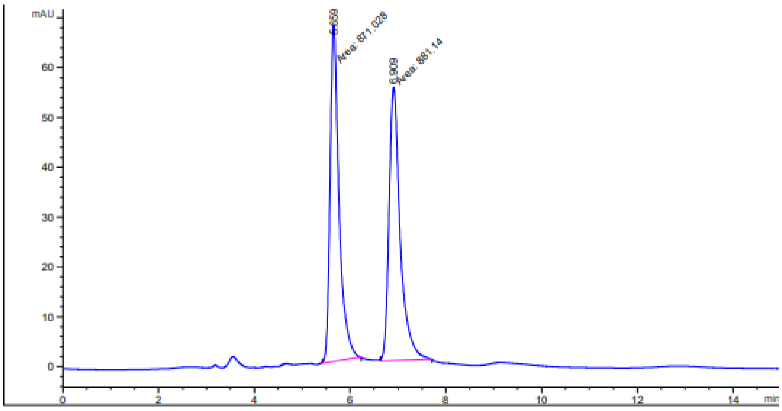

Area Percent Report

Sorted By : Signal
Multiplier : 1.0000
Dilution : 1.0000
Use Multiplier & Dilution Factor with ISTDs

Signal 1: VWD1 A, Wavelength=250 nm

Peak #	RetTime [min]	Type	Width [min]	Area [mAU*s]	Height [mAU]	Area %
1	5.659	MM	0.2156	871.02771	67.33373	49.7114
2	6.909	MM	0.2678	881.13983	54.83912	50.2886

(R)-*N*-(2-(8-Chloroquinolin-2-yl)-2-fluoroethyl)aniline **7w**

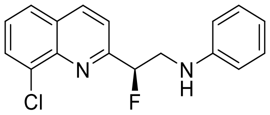

Prepared according to General Procedure B using 2-(1-fluorovinyl)-8-chloroquinoline (10.4 mg, 0.05 mmol, 1.0 equiv), aniline (14 µL, 0.15 mmol, 3.0 equiv), (*S*)-TRIP catalyst (7.5 mg, 0.01 mmol, 20 mol%), and CPME (100 µL, 0.5 M) at 40 °C. The reaction mixture was subjected to the purification outlined in General Procedure B (silica gel, EtOAc:petroleum ether, 5:95 to 10:90) to afford the desired product as a pale brown oil (9.5 mg, 50%).

υ_{max} (film): 1601, 1501, 841, 752, 692, 669 cm^{-1}.

^{1}H NMR (400 MHz, CDCl$_3$): δ 8.25 (d, *J* = 8.5 Hz, 1H), 7.87 (dd, *J* = 7.5, 1.3 Hz, 1H), 7.75 (ddd, *J* = 16.5, 8.4, 1.6 Hz, 2H), 7.49 (dd, *J* = 8.2, 7.5 Hz, 1H), 7.24 – 7.15 (m, 2H), 6.81 – 6.69 (m, 3H), 5.95 (ddd, $^{2}J_{HF}$ = 48.1 Hz, *J* = 6.2, 5.0 Hz, 1H), 4.71 (s, 1H), 4.03 – 3.77 (m, 2H).

^{13}C NMR (101 MHz, CDCl$_3$): δ 159.4 (d, ^{2}J = 25.7 Hz), 147.8, 143.6, 137.7, 133.7, 130.1, 129.5, 129.0, 126.9, 126.9, 118.7 (d, ^{3}J = 6.9 Hz), 118.1, 113.5, 92.7 (d, ^{1}J = 175.2 Hz), 48.4 (d, ^{2}J = 23.1 Hz).

^{19}F NMR (376 MHz, CDCl$_3$): δ −191.99.

HRMS (ESI): exact mass calculated for [M+H]$^{+}$ ($C_{17}H_{15}N_2ClF$) requires *m/z* 301.0902, found *m/z* 301.0898.

The enantiomeric purity of the product was determined by HPLC analysis: 51:49 e.r. (Chiralpak IA, hexane/*i*-PrOH = 50/50, flow rate 0.5 mL/min, λ = 250 nm), t_r (major) = 10.5 min, t_r (minor) = 10.1 min;

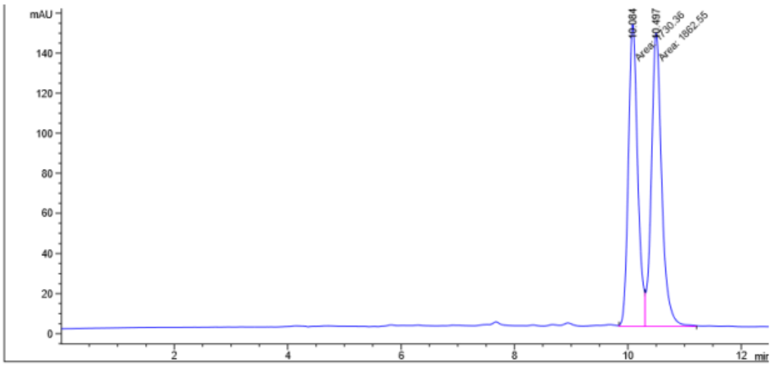

Area Percent Report

Sorted By : Signal
Multiplier: : 1.0000
Dilution: : 1.0000
Use Multiplier & Dilution Factor with ISTDs

Signal 1: DAD1 A, Sig=254,4 Ref=360,100

Peak #	RetTime [min]	Type	Width [min]	Area [mAU*s]	Height [mAU]	Area %
1	10.084	MF	0.1907	1730.35657	151.19955	48.1604
2	10.497	FM	0.2118	1862.54712	146.57484	51.8396

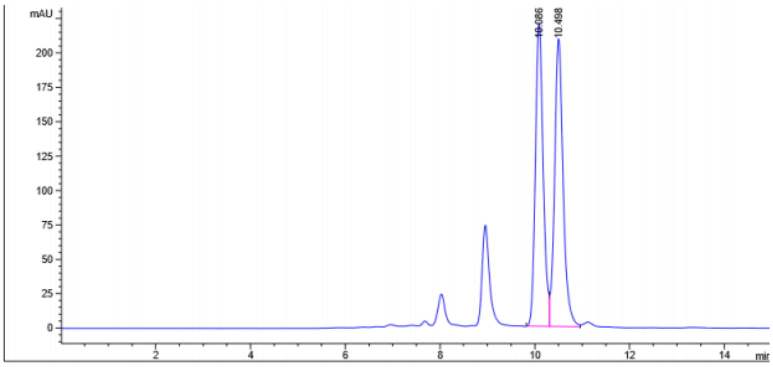

Area Percent Report

Sorted By : Signal
Multiplier: : 1.0000
Dilution: : 1.0000
Use Multiplier & Dilution Factor with ISTDs

Signal 1: DAD1 A, Sig=254,4 Ref=360,100

Peak #	RetTime [min]	Type	Width [min]	Area [mAU*s]	Height [mAU]	Area %
1	10.086	BV	0.1753	2519.43018	220.56381	49.0498
2	10.498	VV	0.1898	2617.04395	209.41792	50.9502

2-(3,3,3-Trifluoroprop-1-en-2-yl)quinoline **10**

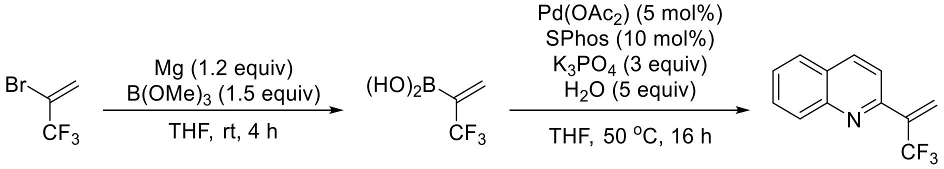

This compound was prepared according to an adapted literature procedure.[8]

An oven-dried round 100 mL round bottom flask was charged with Mg turnings (262 mg, 10.8 mmol, 1.2 equiv), capped and purged with N_2. Then THF (18 mL, 0.5 M) and freshly distilled trimethylborate (1.50 mL, 13.5 mmol, 1.5 equiv) were added sequentially and the mixture was allowed to stir at rt for 5 min. 2-Bromo-1,1,1-trifluoropropene (0.96 mL, 9.00 mmol, 1.0 equiv) was then added dropwise over 5 min, then the reaction was stirred for 4 h at rt. The reaction was quenched using 6 M HCl (50 mL) and the solution was left to stir for 5 min. The organic layer was then separated and the aqueous layer was extracted with Et_2O (3 × 20 mL). The combined organic extracts were washed with brine (50 mL), dried over $NaSO_4$, filtered, and concentrated *in vacuo*. Formation of the boronic acid was confirmed *via* ^{11}B NMR, then the crude was used directly in the next step.

NB. Calculations based upon quantitative conversion to boronic acid in the previous step. An oven-dried 2-necked 100 mL round bottomed flask equipped with a reflux condenser was charged with $Pd(OAc)_2$ (50.5 mg, 0.225 mmol, 5 mol%), SPhos (185 mg, 0.45 mmol, 10 mol%), 2-bromoquinoline (936 mg, 4.50 mmol, 1.0 equiv), the crude boronic acid (9.00 mmol, 2.0 equiv), and K_3PO_4 (2.87 g, 13.5 mmol, 3.00 equiv). The vessel was capped and purged with N_2. THF (22.5 mL, 0.2 M) and water (405 µL, 22.5 mmol, 5.0 equiv) were then added to the mixture sequentially. The reaction was then heated to 50 °C for 16 h. The reaction mixture was then cooled to rt, filtered through Celite, eluting with EtOAc and concentrated *in vacuo*. Purification of the crude *via* flash chromatography (silica gel, EtOAc:petroleum ether, 10:90) gave the desired product as a pale yellow oil (102 mg, 9%).

NB. Due to the reactive nature of this compound, an analytically pure sample was not obtained, data reported as observed. The compound was used directly in the next step.

υ_{max} (film): 2361, 2342, 1504, 1323, 1196, 1126, 1096, 1069, 907, 833, 729, 648 cm^{-1}.

^{1}H NMR (400 MHz, $CDCl_3$): δ 8.20 (d, J = 8.7 Hz, 1H), 8.11 (d, J = 8.4 Hz, 1H), 7.83 (d, J = 8.1 Hz, 1H), 7.74 (ddd, J = 8.4, 6.9, 1.4 Hz, 1H), 7.65 – 7.60 (m, 1H), 7.56 (ddd, J = 8.1, 6.9, 1.1 Hz, 1H), 6.68 (q, $^4J_{HF}$ = 1.8 Hz, 1H), 6.25 (q, $^4J_{HF}$ = 1.4 Hz, 1H).

^{13}C NMR (126 MHz, $CDCl_3$): δ 151.0, 148.0, 138.5 (q, ^2J = 27.7 Hz), 137.0, 130.2, 130.0, 127.8, 127.6, 127.3, 124.5 (q, ^1J = 273.4 Hz), 123.3 (q, ^3J = 6.3 Hz), 119.2.

^{19}F NMR (470 MHz, $CDCl_3$): δ −63.61.

HRMS (ESI): exact mass calculated for $[M+H]^+$ ($C_{12}H_9F_3N$) requires *m/z* 224.0682, found *m/z* 224.0677.

(*R*)-*N*-(3,3,3-Trifluoro-2-(quinolin-2-yl)propyl)aniline **11**

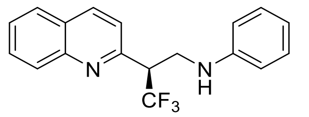

Prepared according to General Procedure B using 2-(3,3,3-trifluoroprop-1-en-2-yl)quinoline (11.2 mg, 0.05 mmol, 1.0 equiv), aniline (14 µL, 0.15 mmol, 3.0 equiv), (*S*)-TRIP catalyst (37.6 mg, 0.05 mmol, 1.0 equiv), and CPME (200 µL, 0.25 M) at −50 °C for 3 d. The reaction mixture was subjected to the purification outlined in General Procedure B (silica gel, EtOAc:petroleum ether, 5:95 to 10:90) to afford the desired product as a pale yellow solid (11.5 mg, 73%).

υ_{max} (film): 2361, 2342, 1601, 1504, 1315, 1254, 1165, 1111, 826, 752, 694 cm^{-1}.

^{1}H NMR (500 MHz, CDCl$_3$): δ 8.18 – 8.14 (m, 2H), 7.85 (dd, J = 8.2, 1.4 Hz, 1H), 7.77 (ddd, J = 8.4, 6.9, 1.5 Hz, 1H), 7.59 (ddd, J = 8.1, 6.9, 1.2 Hz, 1H), 7.40 (d, J = 8.4 Hz, 1H), 7.20 – 7.14 (m, 2H), 6.73 (tt, J = 7.3, 1.1 Hz, 1H), 6.59 (dt, J = 7.7, 1.0 Hz, 2H), 4.20 – 3.82 (m, 4H).

^{13}C NMR (126 MHz, CDCl$_3$): δ 153.4, 148.0, 147.0, 137.1, 130.1, 129.7, 129.5, 127.7, 127.7, 127.2, 126.1 (q, ^{1}J = 280.5 Hz), 122.1, 118.2, 113.2, 51.1 (q, ^{2}J = 25.0 Hz), 41.9.

^{19}F NMR (470 MHz, CDCl$_3$): δ −67.02.

HRMS (ESI): exact mass calculated for $[M+H]^+$ ($C_{18}H_{16}F_3N_2$) requires *m/z* 317.1260, found *m/z* 317.1251.

The enantiomeric purity of the product was determined by HPLC analysis: 90:10 e.r. (Chiralpak IA, hexane/*i*-PrOH = 50/50, flow rate 1.0 mL/min, λ = 250 nm), t_r (major) = 9.1 min, t_r (minor) = 11.6 min;

$[\alpha]_D^{20}$ = −80.4 (*c* 1.00, CHCl$_3$).

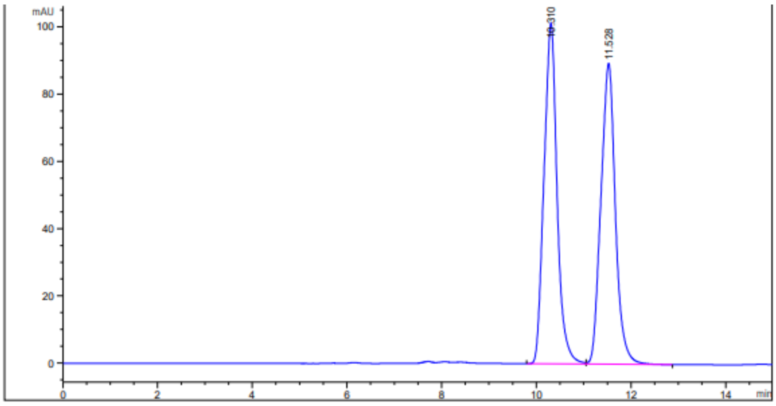


```
=====================================================================
                         Area Percent Report
=====================================================================

Sorted By             :      Signal
Multiplier            :      1.0000
Dilution              :      1.0000
Use Multiplier & Dilution Factor with ISTDs

Signal 1: VWD1 A, Wavelength=250 nm

Peak RetTime Type  Width     Area      Height     Area
  #   [min]        [min]   [mAU*s]     [mAU]        %
----|-------|----|-------|----------|----------|--------|
   1  10.310 BV    0.2817 1941.33411  101.37083  50.5532
   2  11.528 VB    0.3160 1898.84473   89.48219  49.4468
```

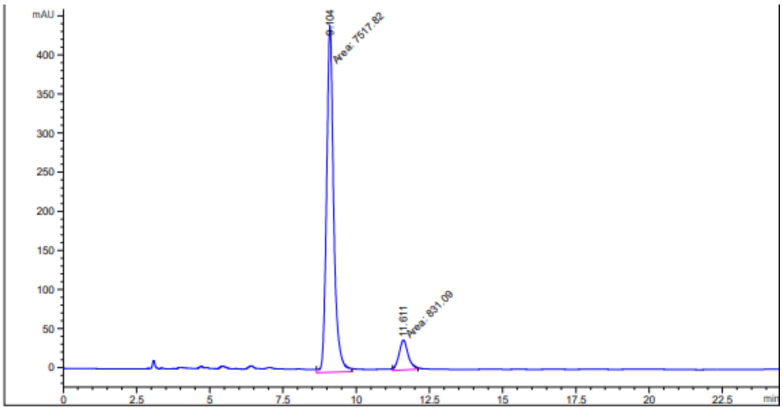


```
=====================================================================
                         Area Percent Report
=====================================================================

Sorted By             :      Signal
Multiplier            :      1.0000
Dilution              :      1.0000
Use Multiplier & Dilution Factor with ISTDs


Signal 1: VWD1 A, Wavelength=210 nm

Peak RetTime Type  Width     Area      Height     Area
  #   [min]        [min]   [mAU*s]     [mAU]        %
----|-------|----|-------|----------|----------|--------|
   1   9.104 MM    0.2829 7517.82324  442.87985  90.0455
   2  11.611 MM    0.3643  831.08960   38.02613   9.9545
```

N-(2-Fluoro-2-(quinolin-2-yl)ethyl)-*N*-phenylacetamide **16**

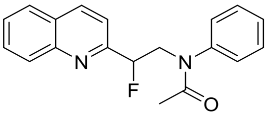

Prepared according to literature procedure.[9]

A 10 mL microwave vial was charged with *N*-(2-fluoro-2-(quinolin-2-yl)ethyl)aniline (151 mg, 0.57 mmol, 1.0 equiv). The vial was capped and purged with N_2. Dry CH_2Cl_2 (1 mL) and Ac_2O (54 µL, 0.57 mmol, 1.0 equiv) were added to the vessel and the reaction mixture was allowed to stir at rt for 16 h. The reaction was then quenched with 2 M HCl (1 mL) and the aqueous phase was extracted with CH_2Cl_2 (2 × 5 mL). The combined organic phases were dried over Na_2SO_4, filtered, and concentrated *in vacuo*. Purification of the crude *via* flash chromatography (silica gel, EtOAc:petroleum ether, 5:95 to 10:90) gave the desired product as a pale yellow oil (51.0 mg, 29%).

υ_{max} (film): 1659, 1597, 1497, 1427, 1389, 1300, 1281, 1072, 1026, 837, 756, 702 cm^{-1}.

^{1}H NMR (500 MHz, $CDCl_3$): δ 8.21 (d, *J* = 8.5 Hz, 1H), 8.03 (d, *J* = 8.5 Hz, 1H), 7.82 (d, *J* = 8.1 Hz, 1H), 7.71 (t, *J* = 7.7 Hz, 1H), 7.63 (d, *J* = 8.2 Hz, 1H), 7.55 (t, *J* = 7.5 Hz, 1H), 7.43 (t, *J* = 7.5 Hz, 2H), 7.35 (dd, *J* = 23.7, 7.4 Hz, 3H), 5.99 – 5.84 (m, 1H), 4.42 – 4.26 (m, 2H), 1.90 (s, 3H).

^{13}C NMR (126 MHz, $CDCl_3$): δ 171.2, 157.4 (d, ^2J = 22.2 Hz), 147.5, 143.2, 137.2, 130.0, 129.9, 129.4, 128.6, 128.3, 127.9, 127.8, 127.0, 118.3 (d, ^3J = 5.6 Hz), 92.0 (d, ^1J = 176.9 Hz), 53.2 (d, ^2J = 22.7 Hz), 23.0.

^{19}F NMR (470 MHz, $CDCl_3$): δ −189.42.

HRMS (ESI): exact mass calculated for $[M+H]^+$ ($C_{19}H_{18}FN_2O$) requires *m/z* 309.1398, found *m/z* 309.1388.

8. References

1. Armarego, W. L. F.; Chai, C. *Purification of Laboratory Chemicals, 7th ed.*, Elsevier, Oxford, **2013**.

2. T. Hanamoto, T. Kobayashi, *J. Org. Chem* **2003**, *68*, 6354–6359.

3. T. Dorn, C. Janiak, K. Abu-Shandi, *CrystEngComm* **2005**, *7*, 633–641.

4. Gaussian 09, Revision C.01, M. J. Frisch, G. W. Trucks, H. B. Schlegel, G. E. Scuseria, M. A. Robb, J. R. Cheeseman, G. Scalmani, V. Barone, G. A. Petersson, H. Nakatsuji, X. Li, M. Caricato, A. Marenich, J. Bloino, B. G. Janesko, R. Gomperts, B. Mennucci, H. P. Hratchian, J. V. Ortiz, A. F. Izmaylov, J. L. Sonnenberg, D. Williams-Young, F. Ding, F. Lipparini, F. Egidi, J. Goings, B. Peng, A. Petrone, T. Henderson, D. Ranasinghe, V. G. Zakrzewski, J. Gao, N. Rega, G. Zheng, W. Liang, M. Hada, M. Ehara, K. Toyota, R. Fukuda, J. Hasegawa, M. Ishida, T. Nakajima, Y. Honda, O. Kitao, H. Nakai, T. Vreven, K. Throssell, J. A. Montgomery, Jr., J. E. Peralta, F. Ogliaro, M. Bearpark, J. J. Heyd, E. Brothers, K. N. Kudin, V. N. Staroverov, T. Keith, R. Kobayashi, J. Normand, K. Raghavachari, A. Rendell, J. C. Burant, S. S. Iyengar, J. Tomasi, M. Cossi, J. M. Millam, M. Klene, C. Adamo, R. Cammi, J. W. Ochterski, R. L. Martin, K. Morokuma, O. Farkas, J. B. Foresman, and D. J. Fox, Gaussian, Inc., Wallingford CT, 2016.

5. Ignacio Funes-Ardoiz, Robert S. Paton, *GoodVibes: Version 2.0.3*, Zenodo, **2018**.

6. D, Wang, Y. Wang, J. Zhao, L. Li, L. Miao, D. Wang, H. Sun, P. Yu, *Tetrahedron* **2016**, *72*, 5762–5768.

7. S. E. Wengryniuk, A. Weickgenannt, C. Reiher, N. A. Strotman, K. Chen, M. D. Eastgate, P. S. Baran *Org. Lett.* **2013**, *15*, 792–795.

8. B. Jiang, Q.-F. Wang, C.-G. Yang, M. Xu *Tetrahedron Lett*. **2001**, *42*, 4083–4085.

9. A. Romanens, G. Bélanger, *Org. Lett.* **2015**, *17*, 322–325.

9. NMR Spectra

^{1}H NMR of 5a

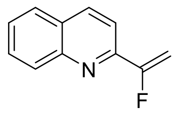

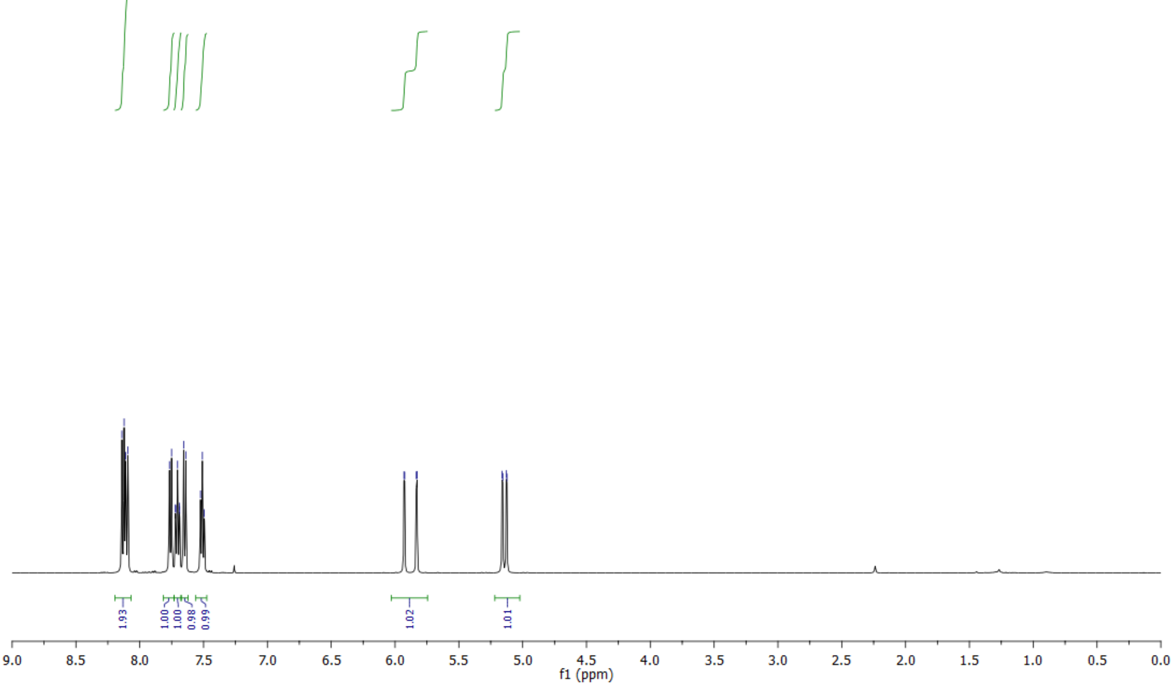

^{13}C NMR of 5a

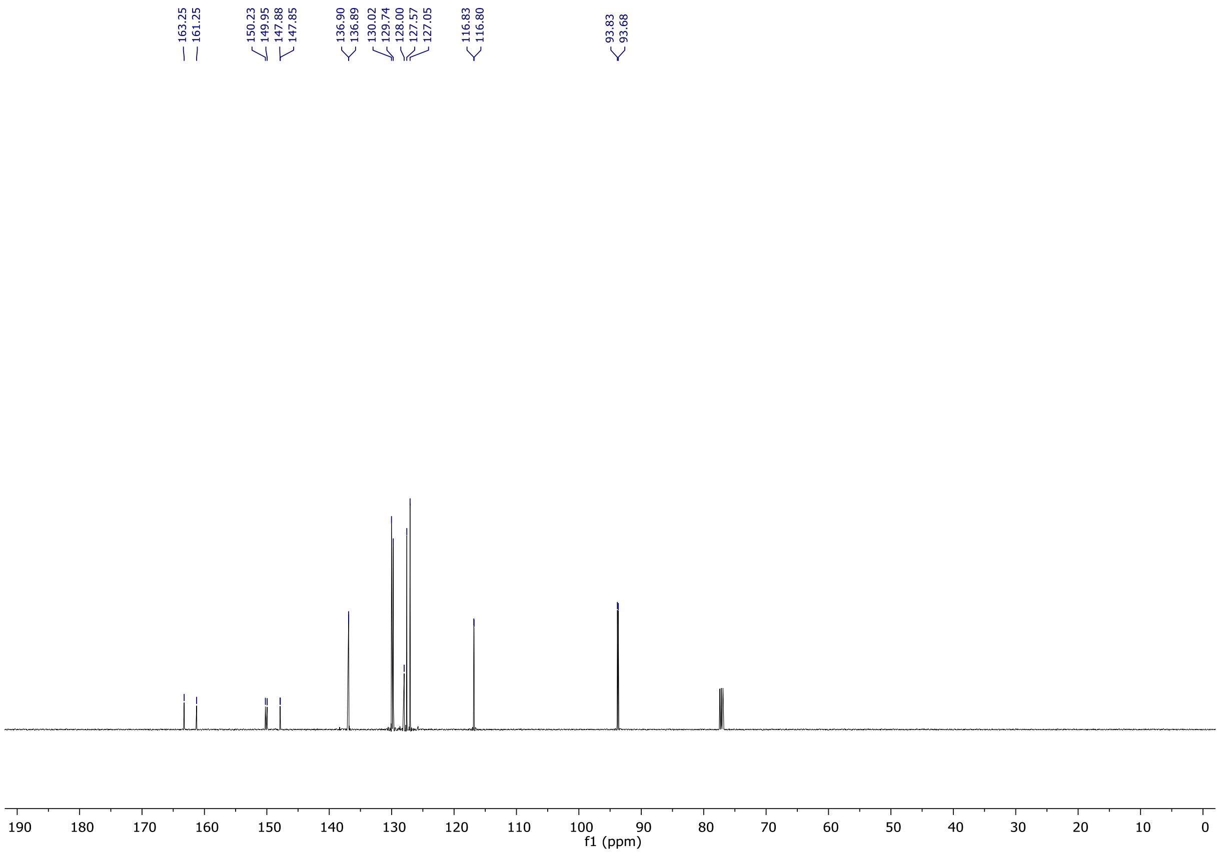

^{19}F NMR of 5a

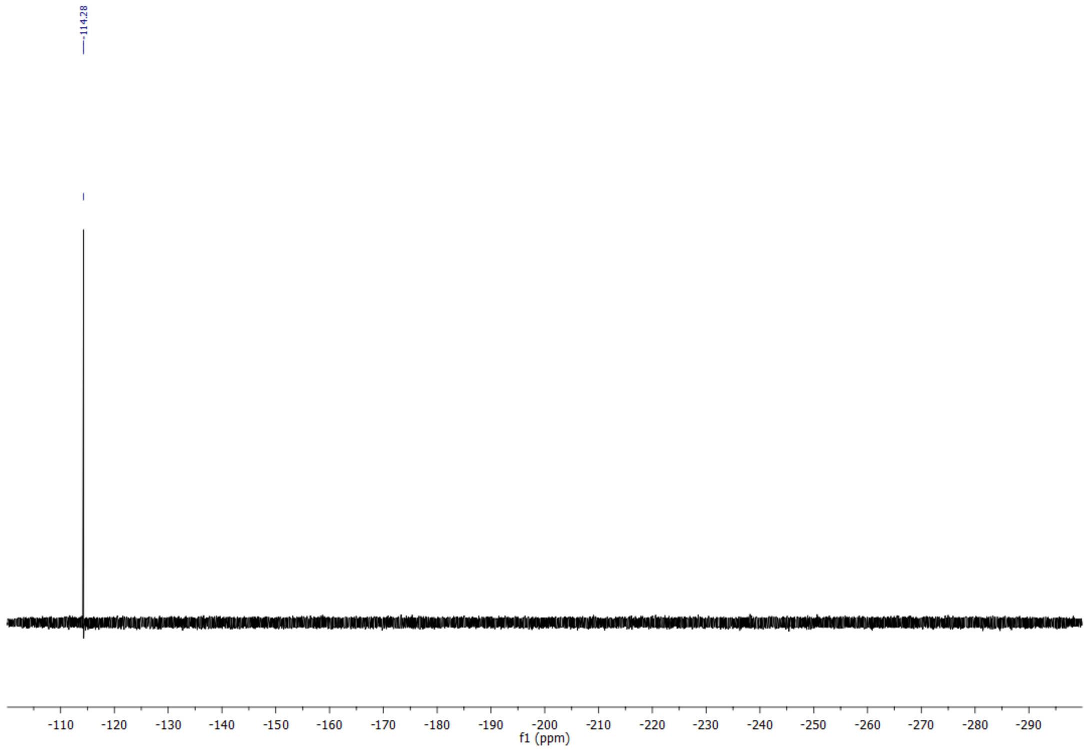

^{1}H NMR of 5b

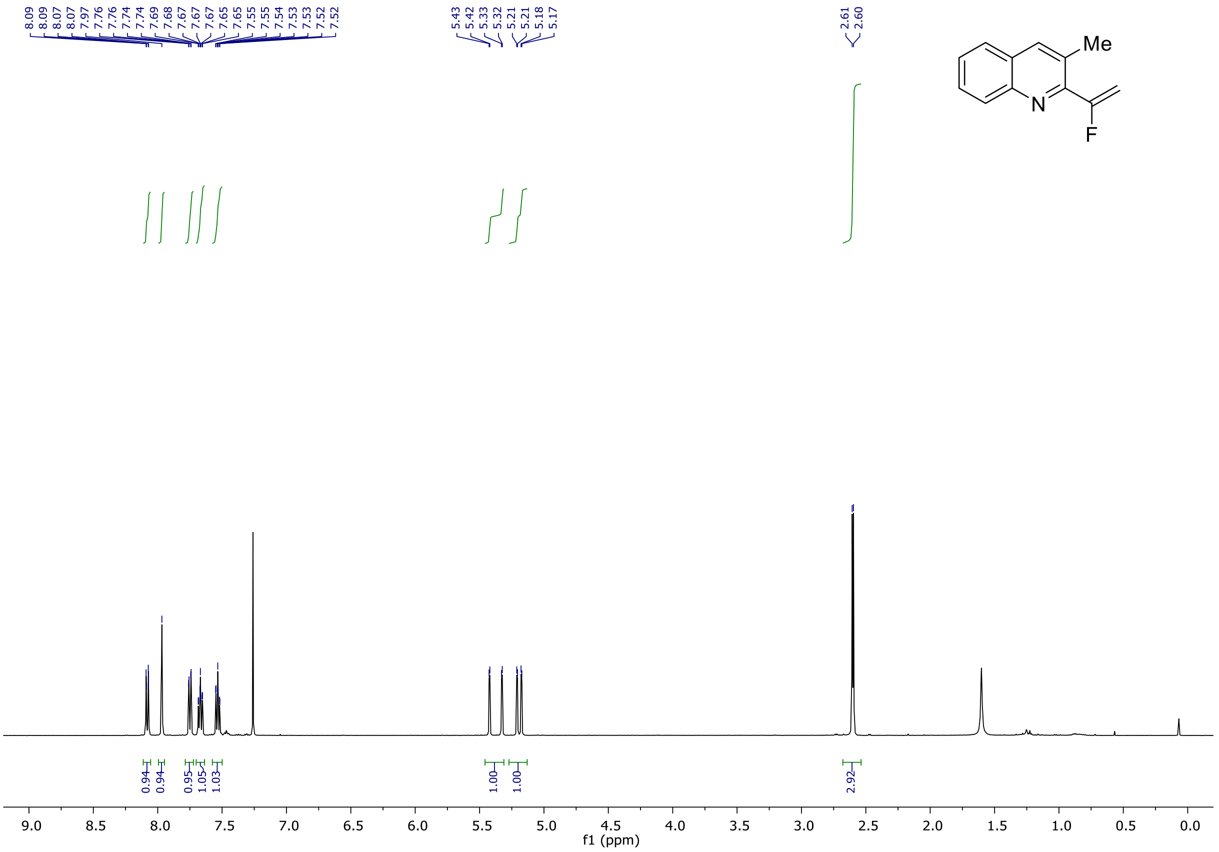

^{13}C NMR of 5b

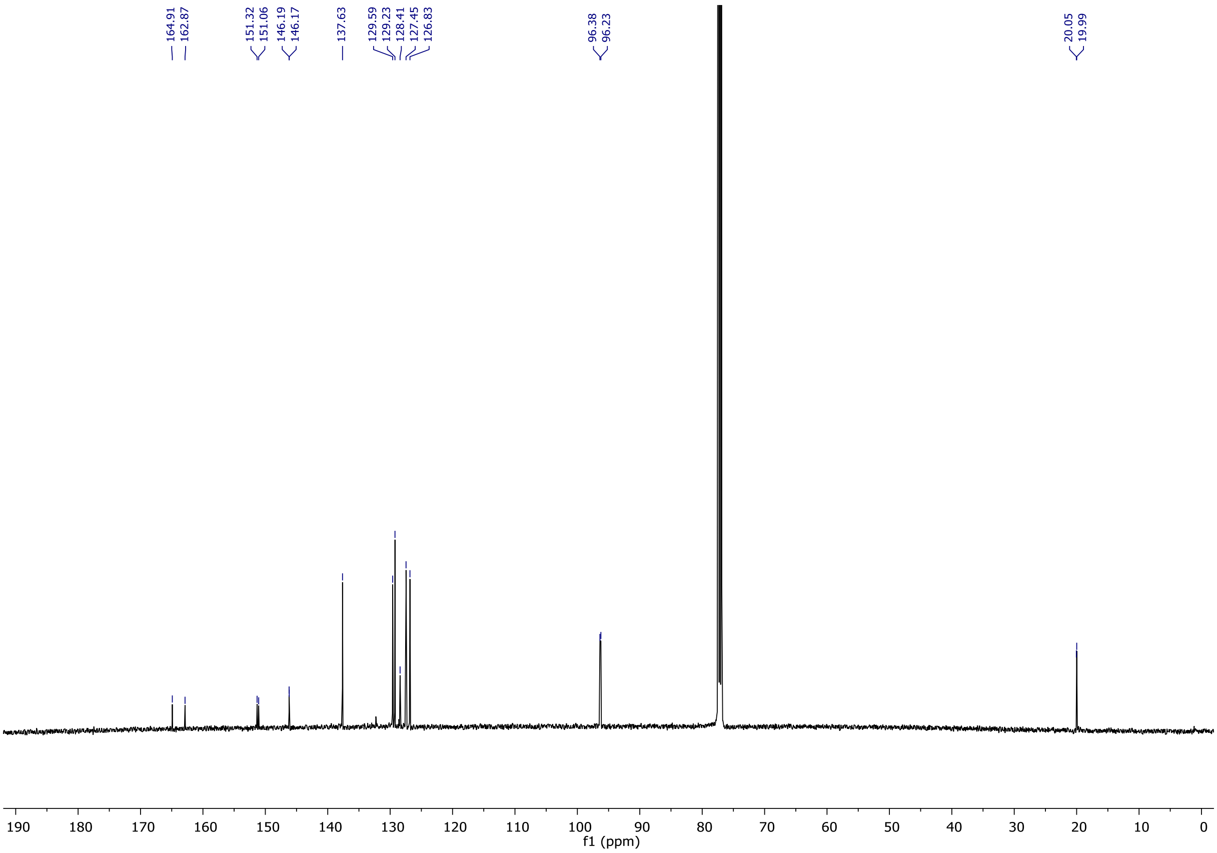

^{19}F NMR of 5b

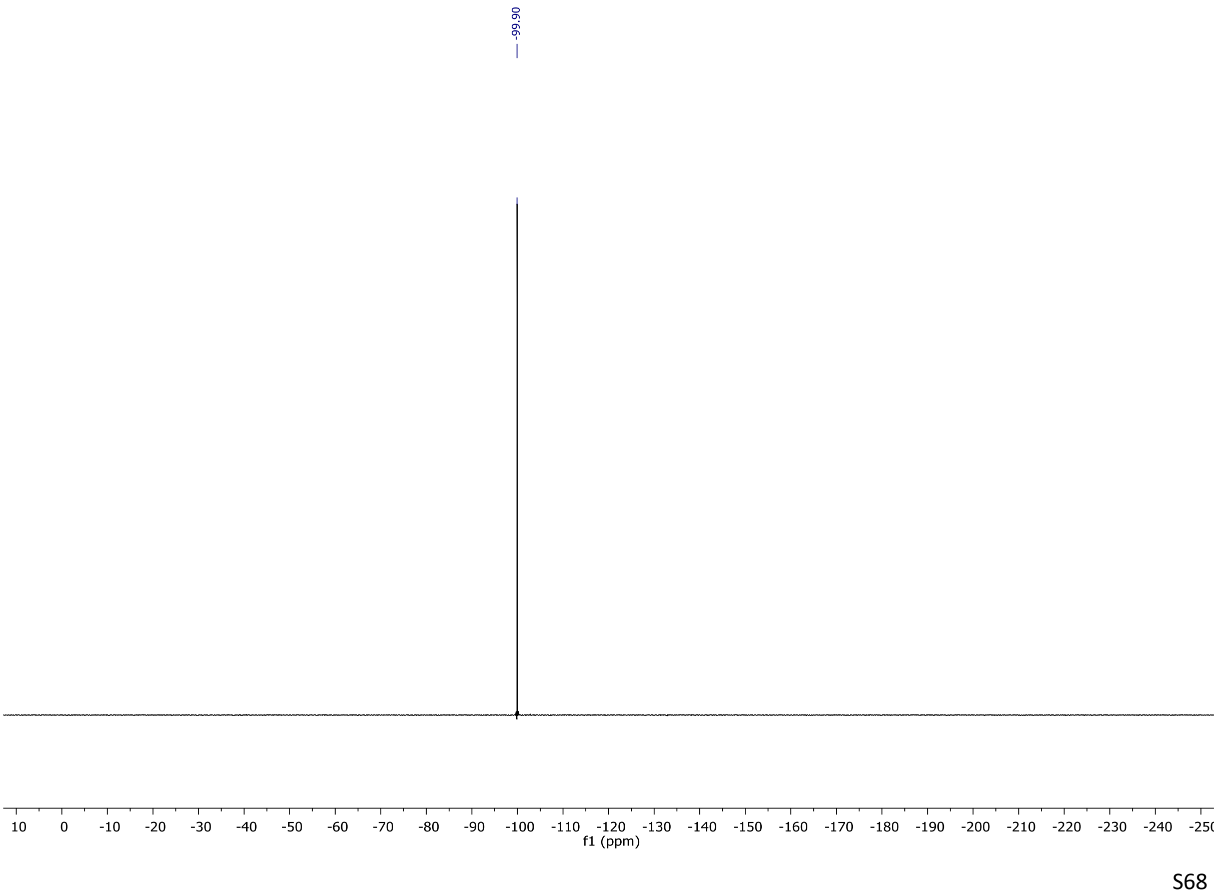

^{1}H NMR of 5c

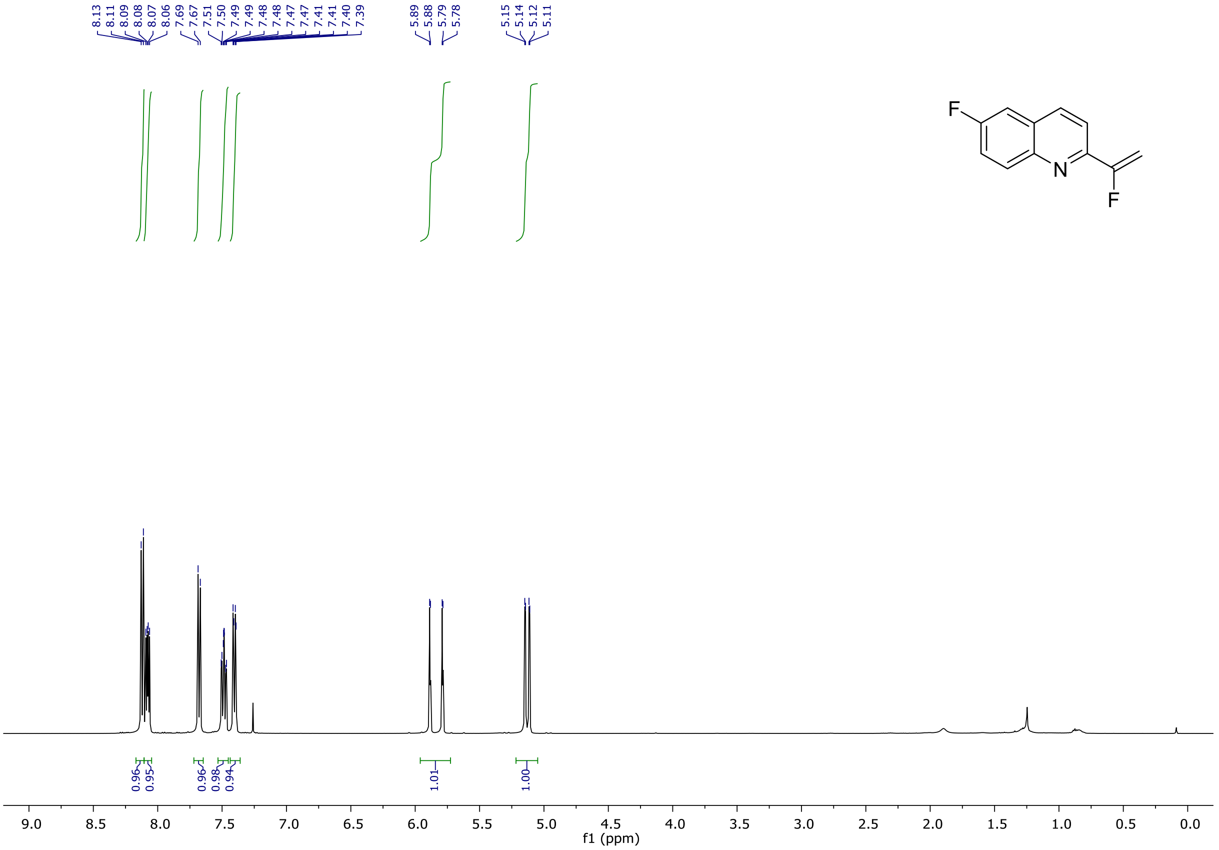

^{13}C NMR of 5c

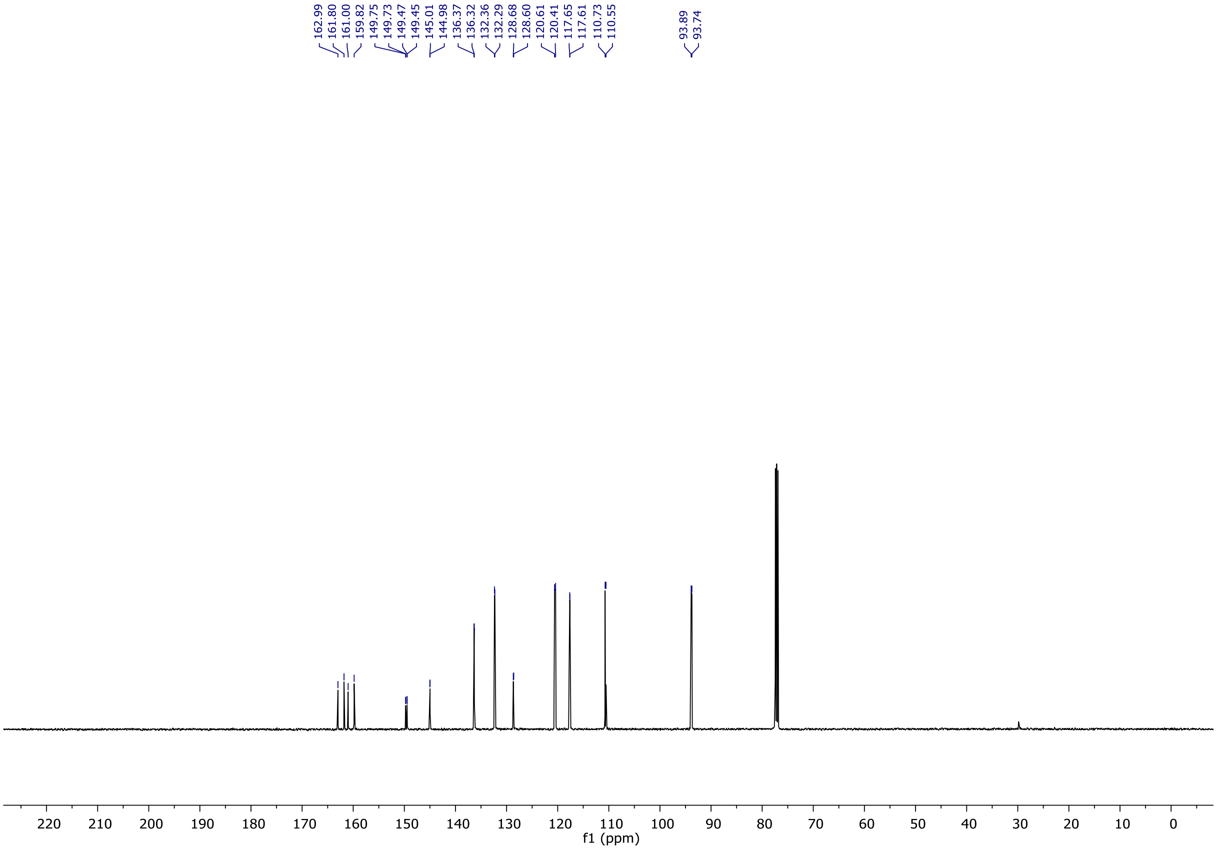

^{19}F NMR of 5c

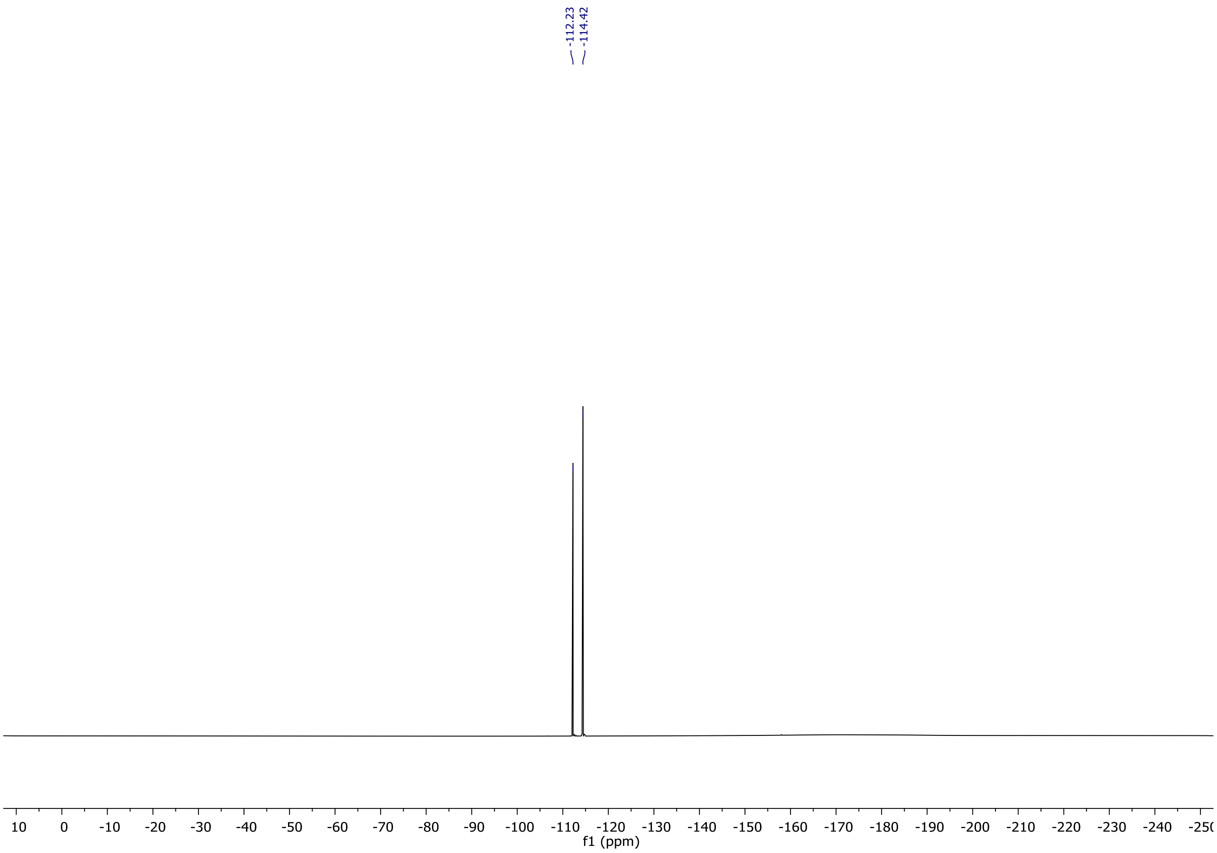

^{1}H NMR of 5d

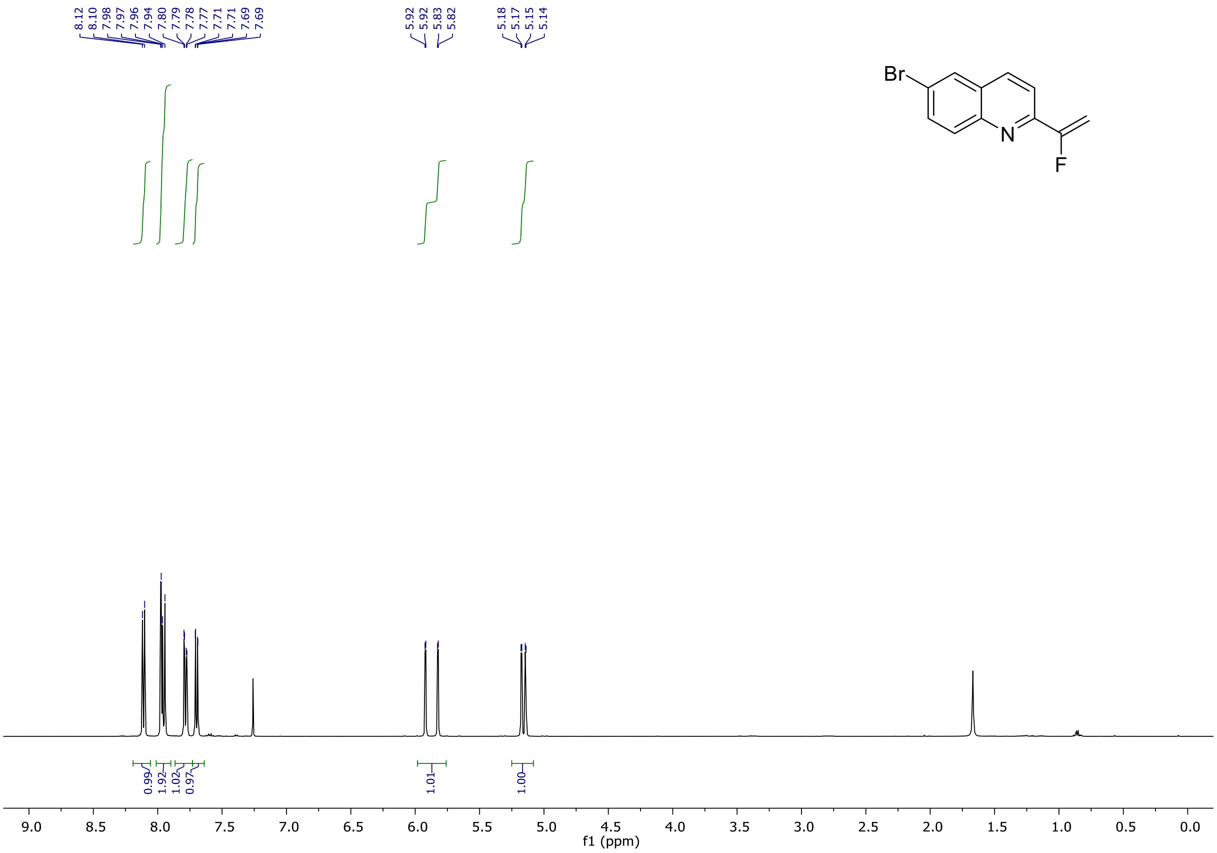

^{13}C NMR of 5d

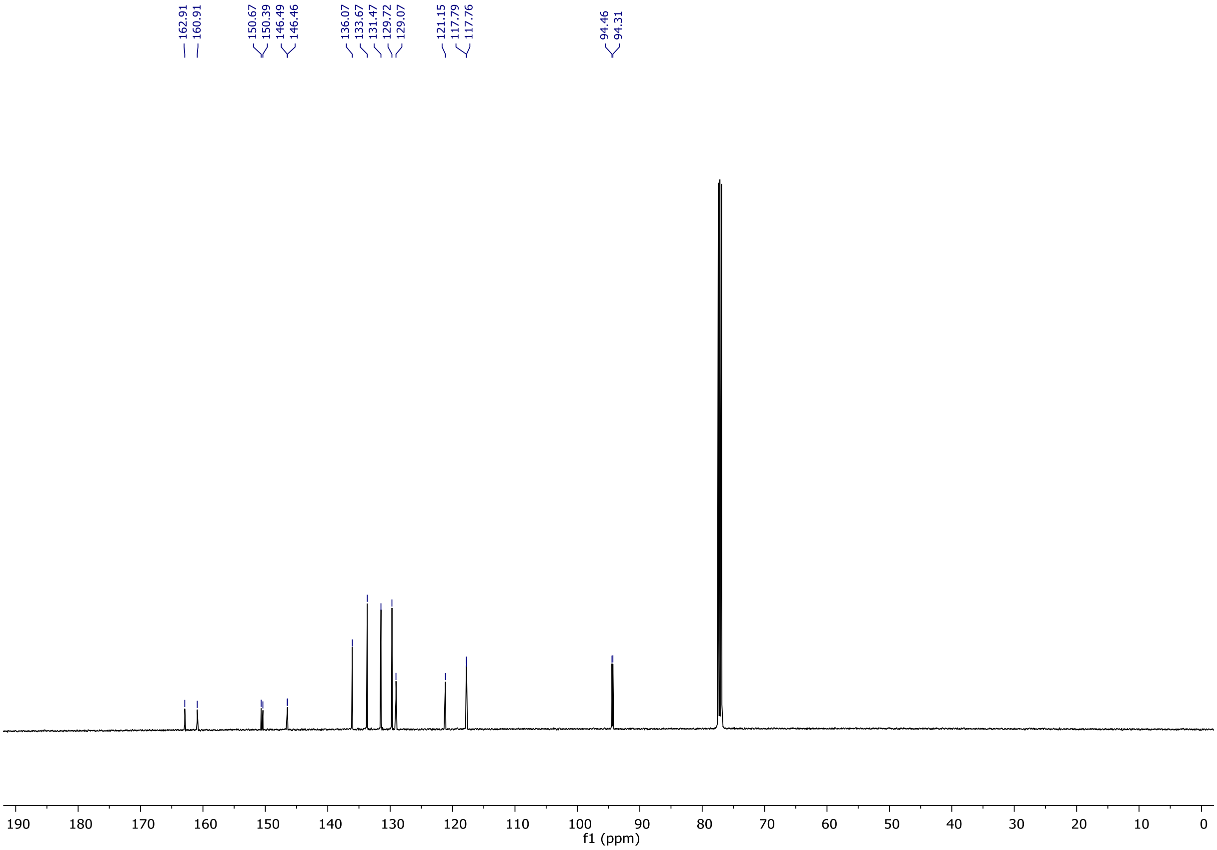

^{19}F NMR of 5d

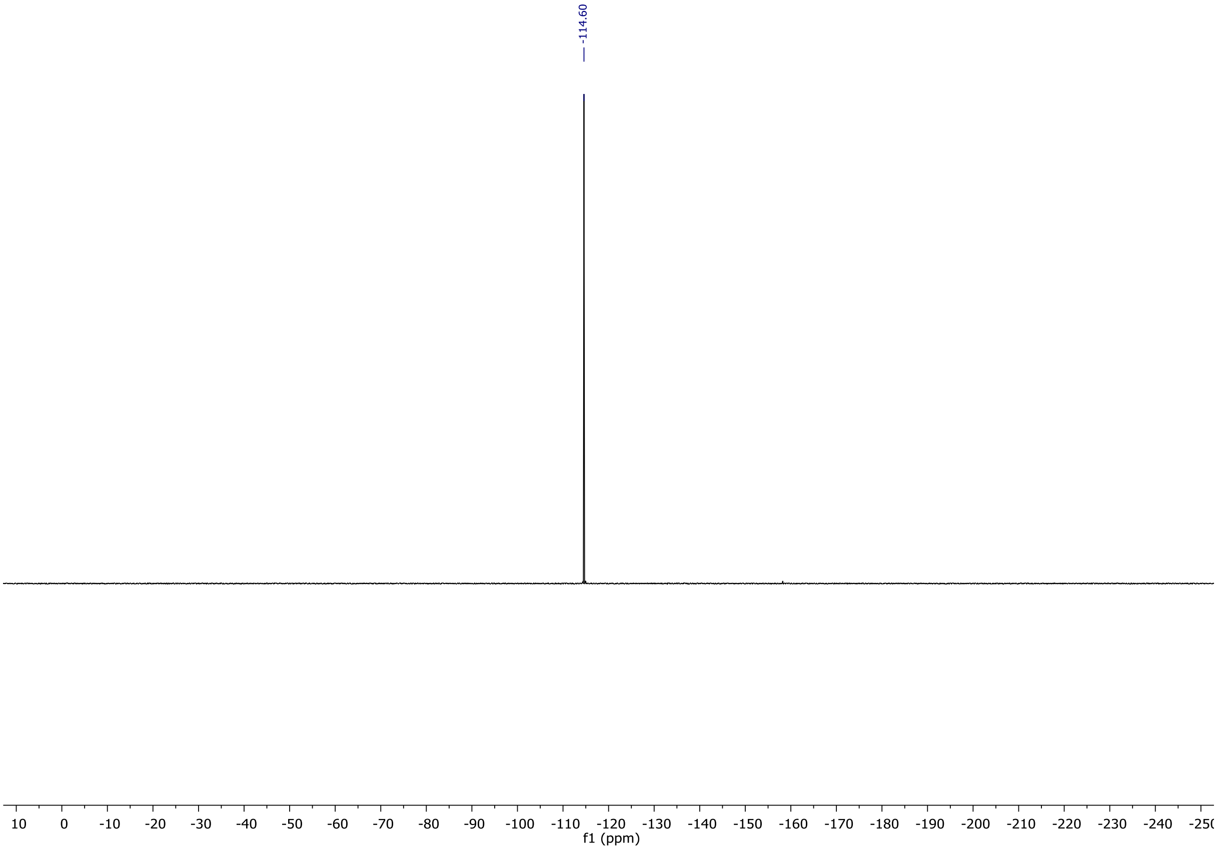

^{1}H NMR of 5e

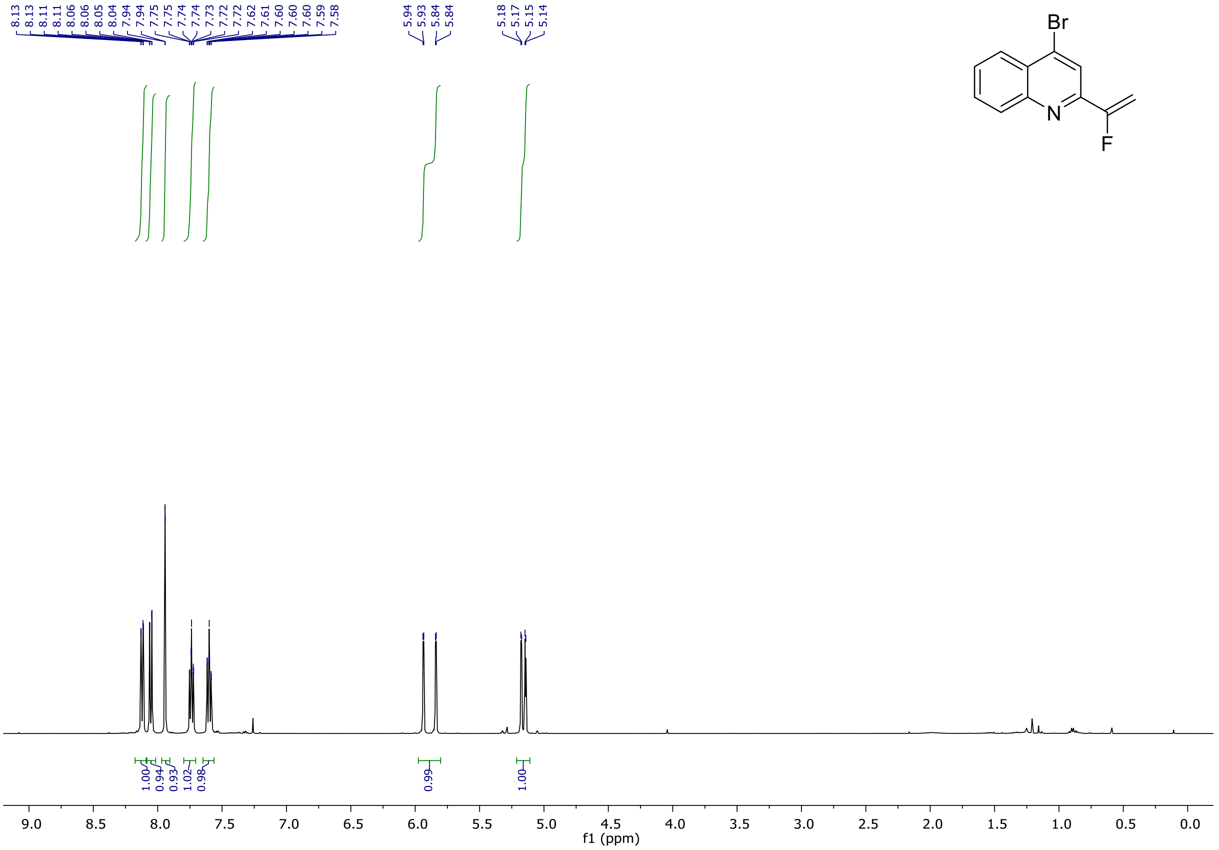

^{13}C NMR of 5e

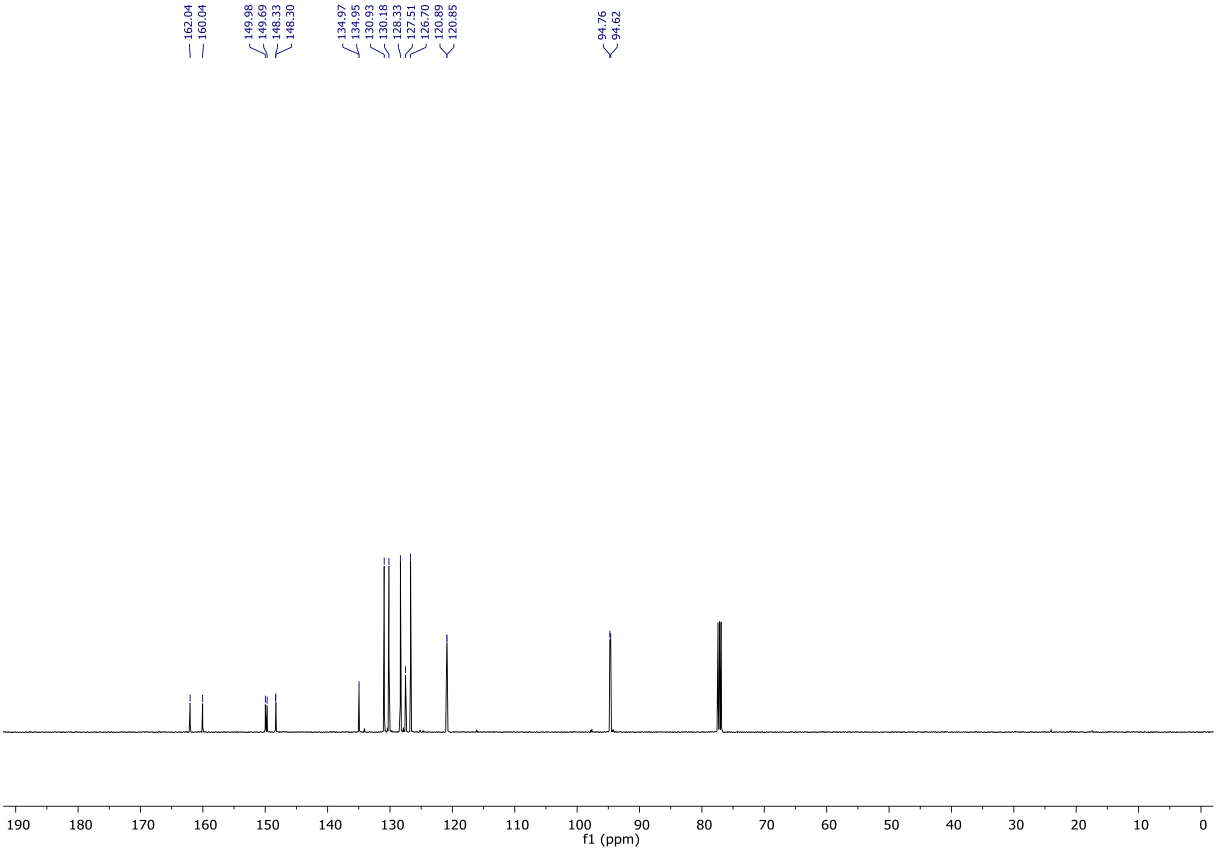

^{19}F NMR of 5e

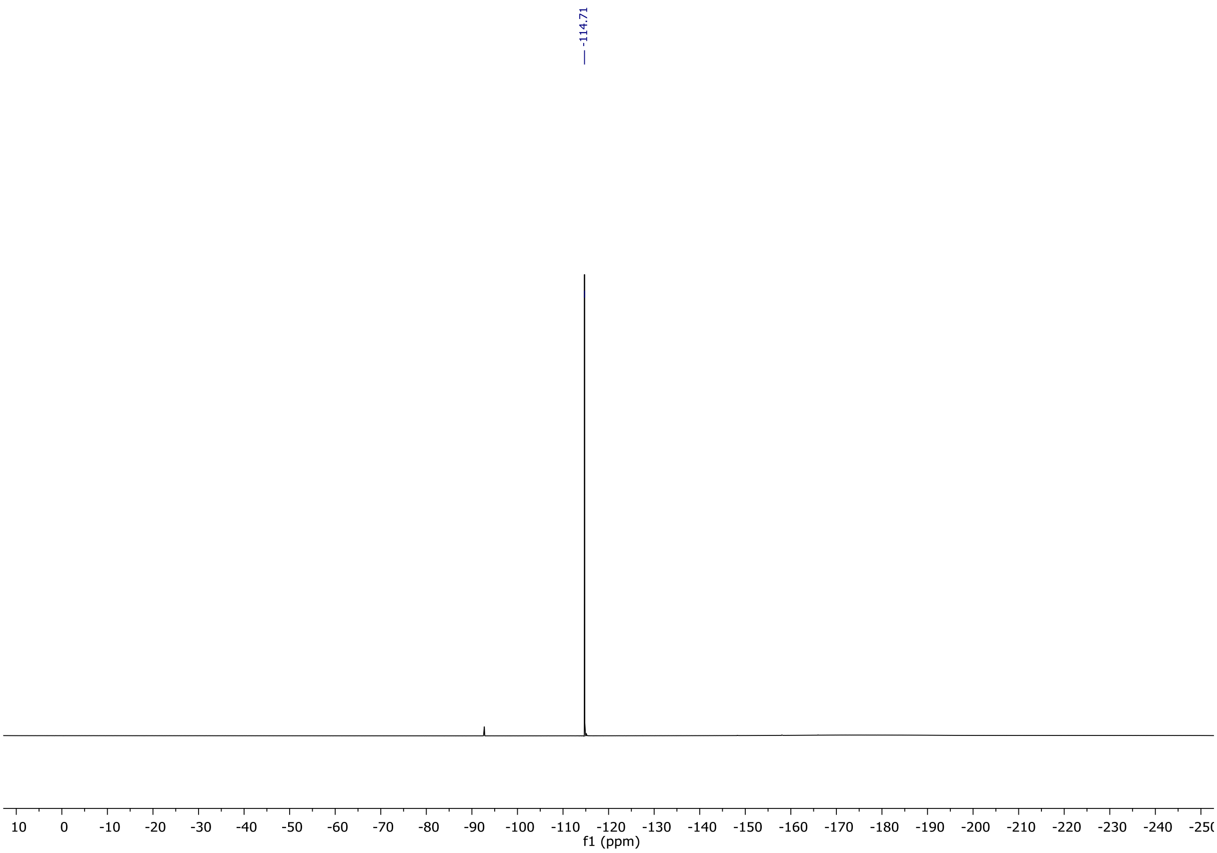

^{1}H NMR of 5f

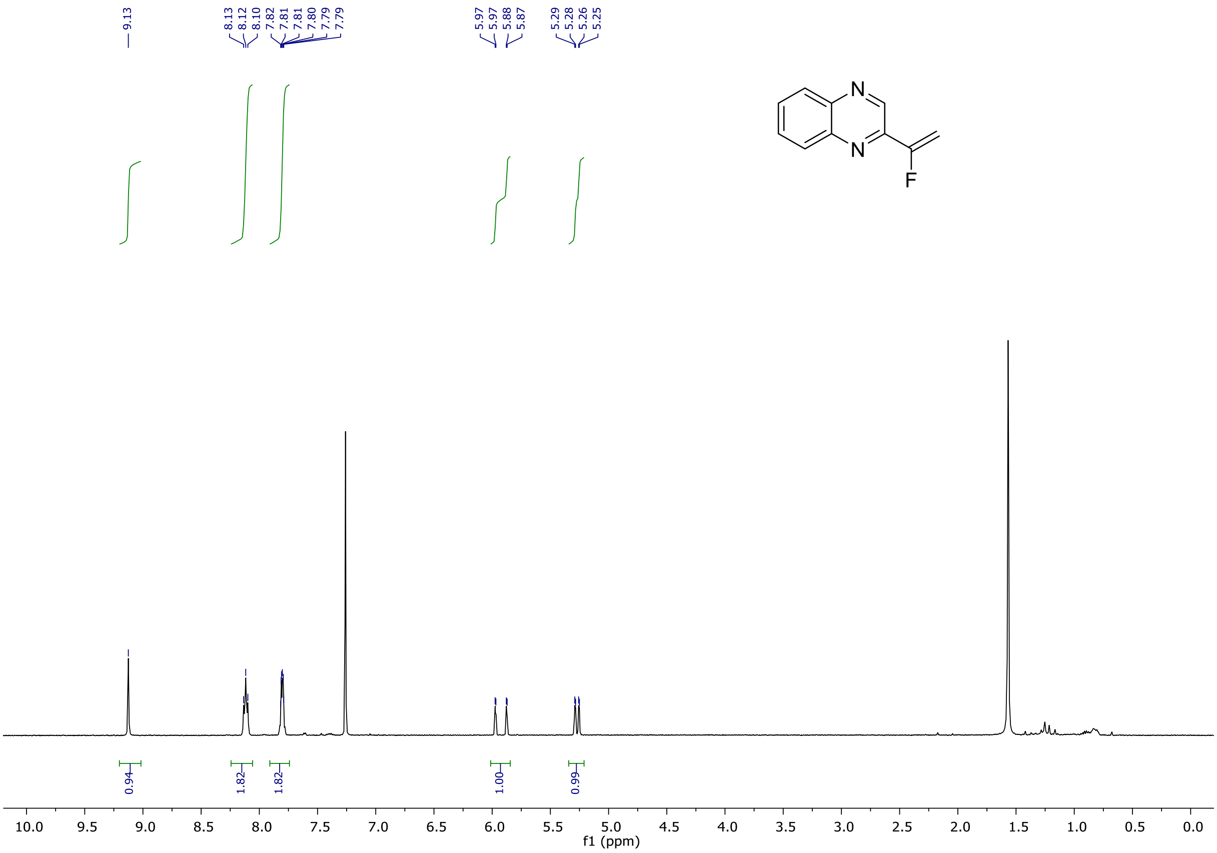

^{13}C NMR of 5f

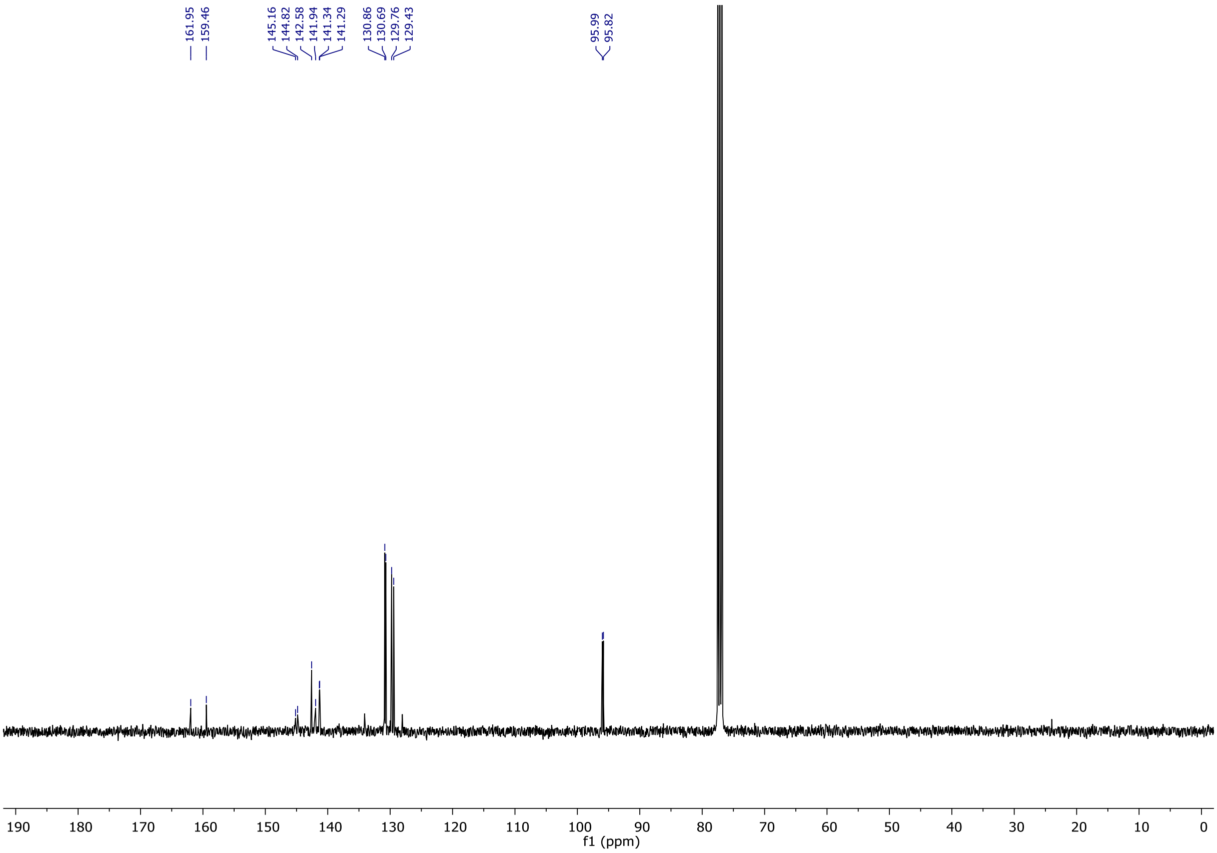

^{19}F NMR of 5f

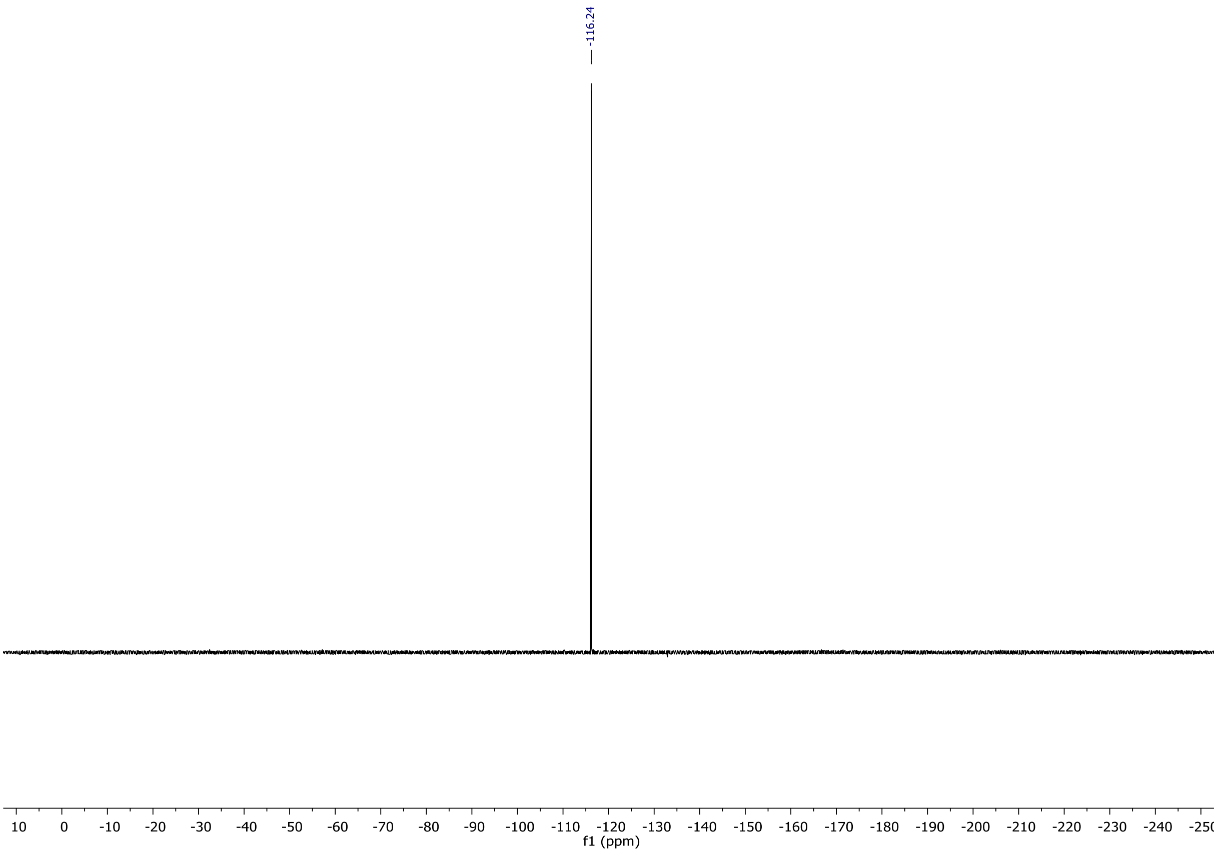

^{1}H NMR of 5g

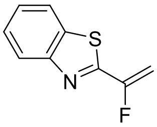

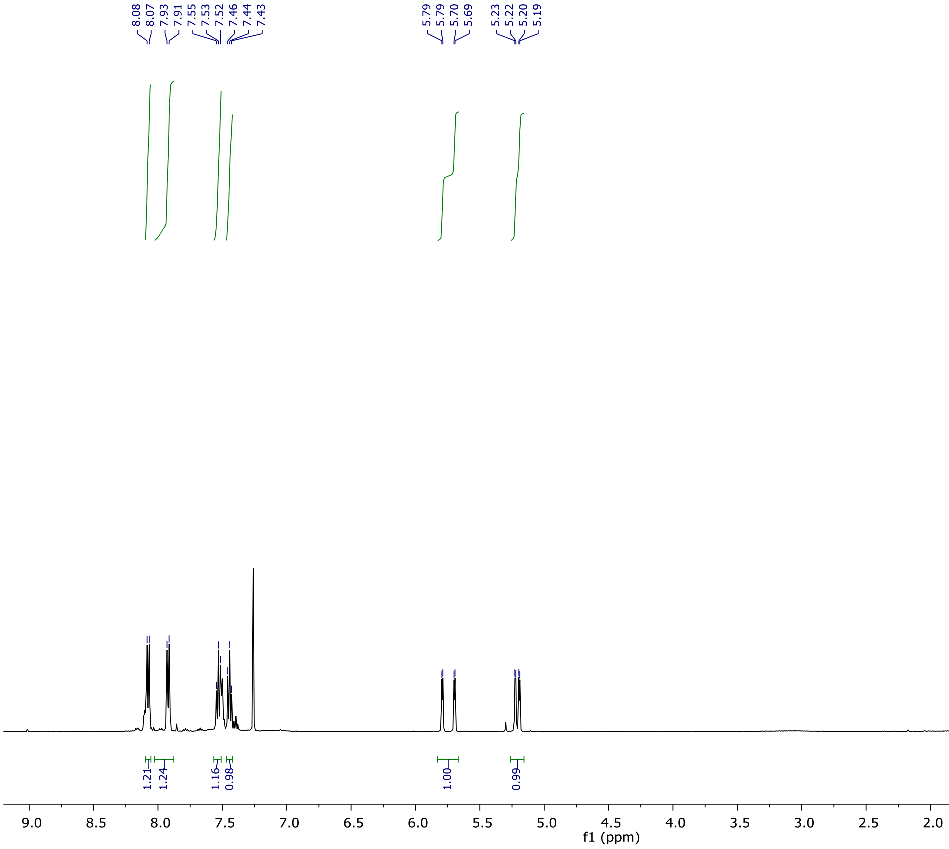

^{13}C NMR of 5g

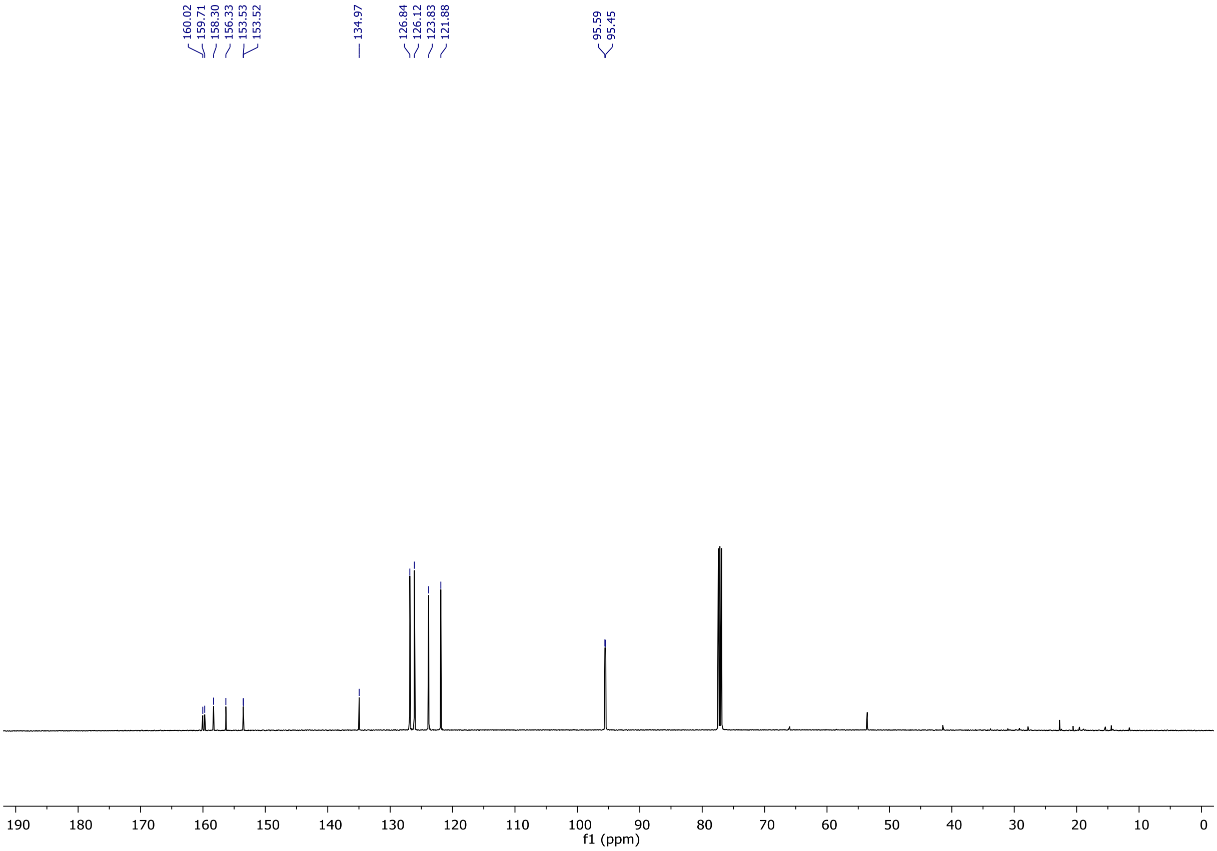

^{19}F NMR of 5g

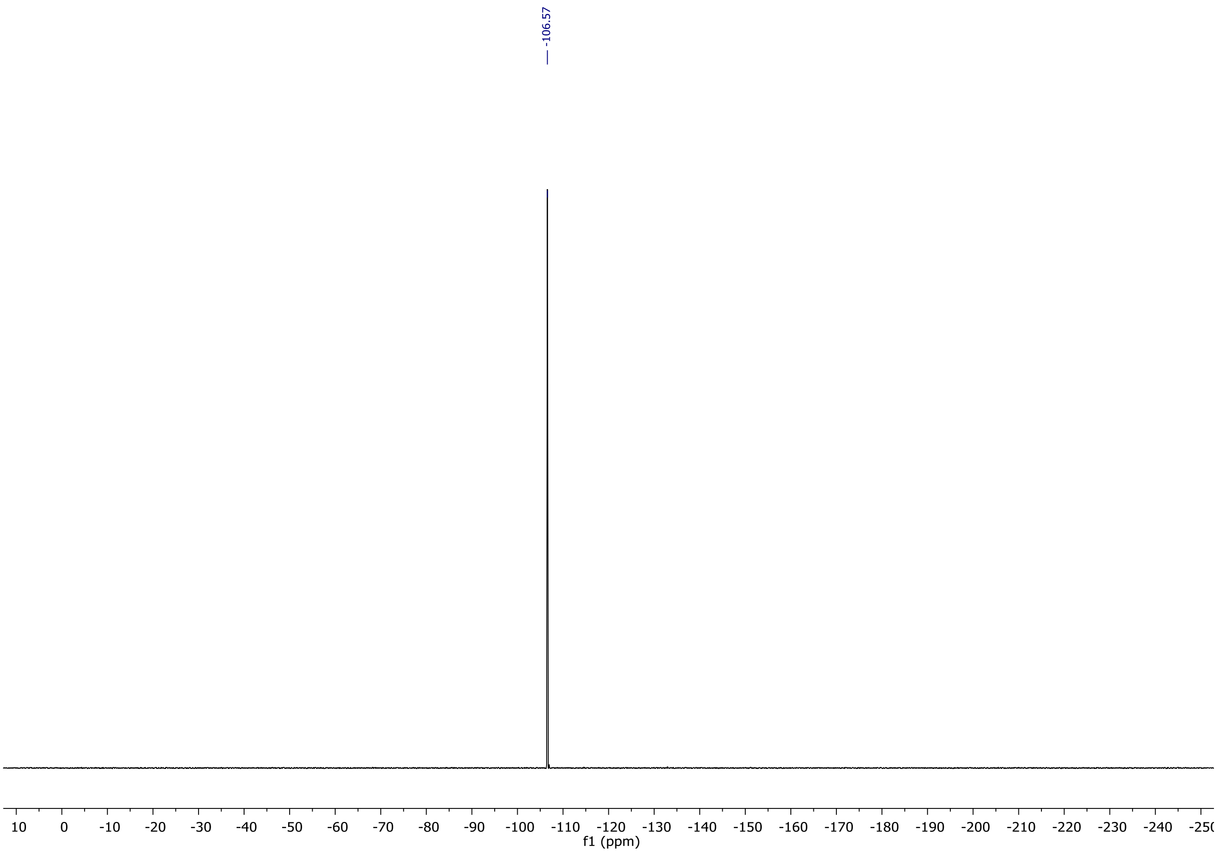

^{1}H NMR of 5h

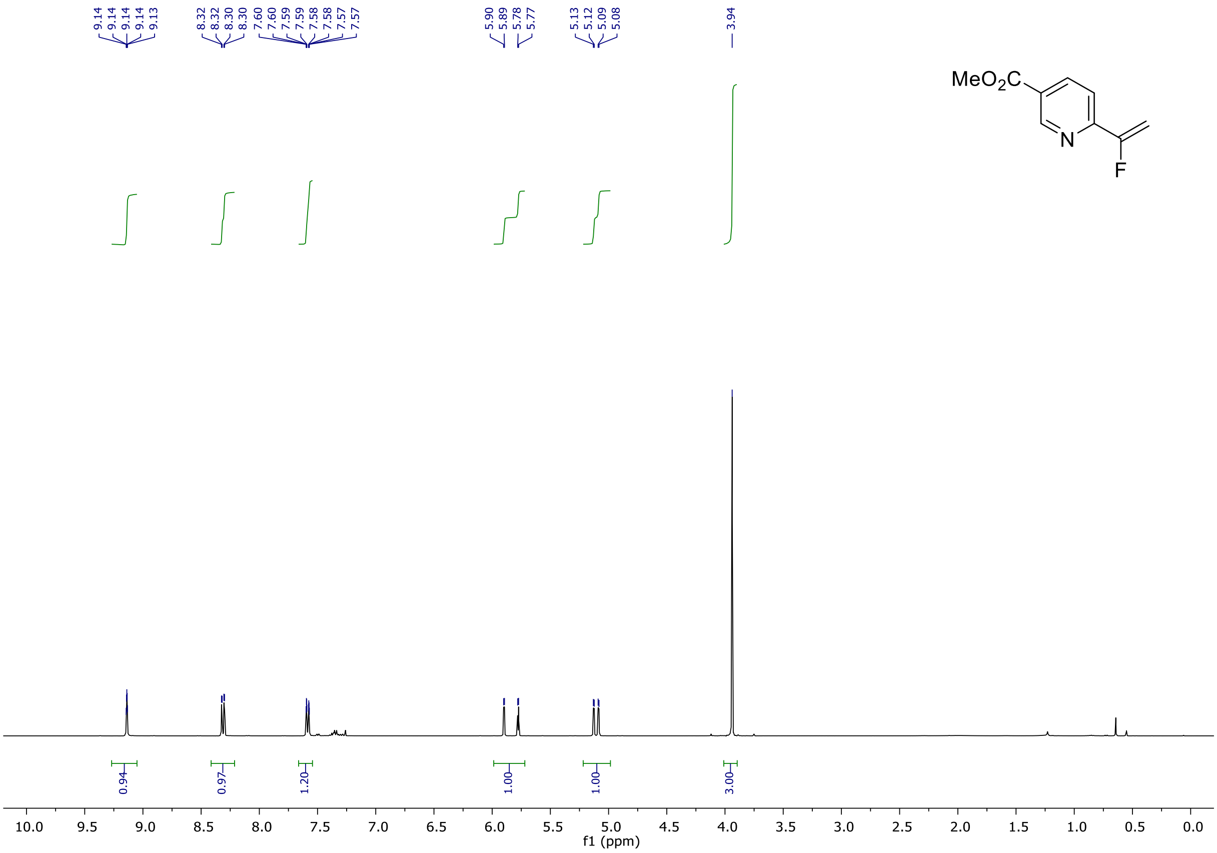

^{13}C NMR of 5h

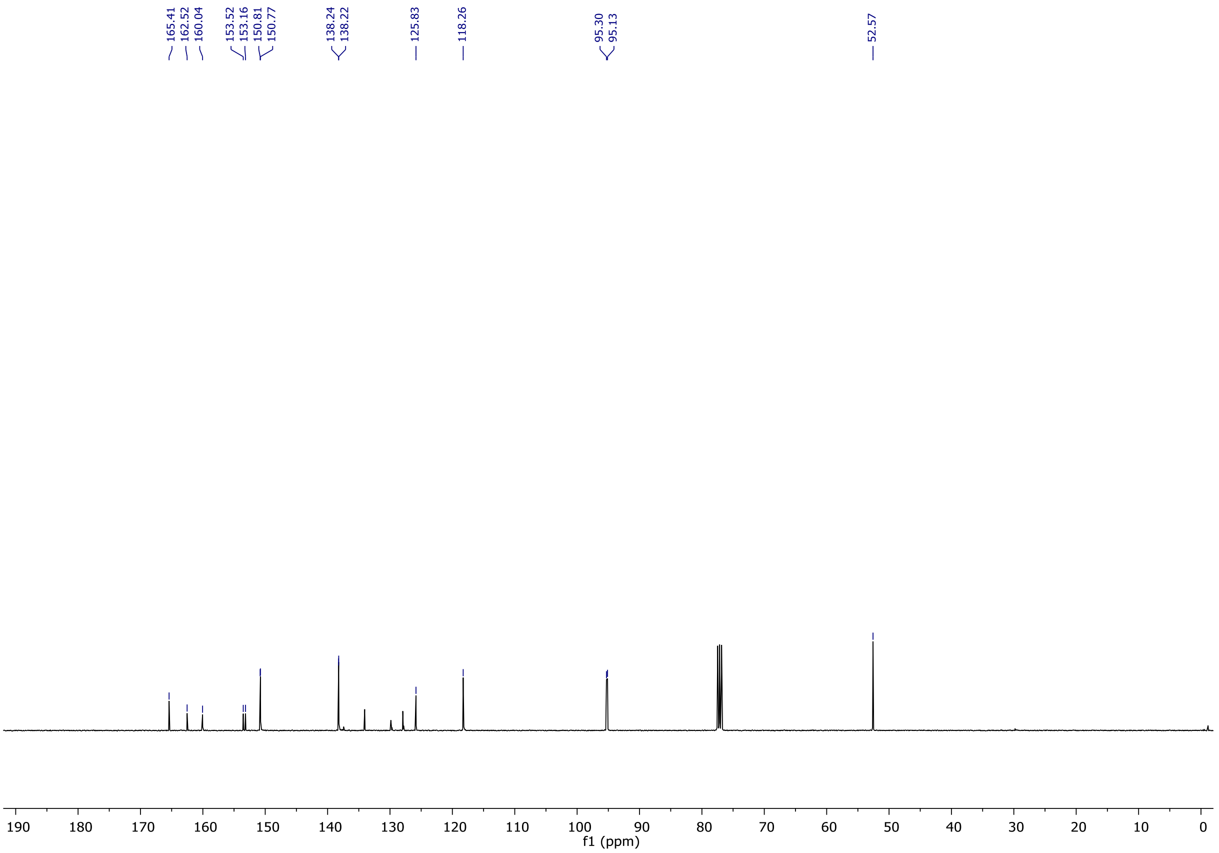

^{19}F NMR of 5h

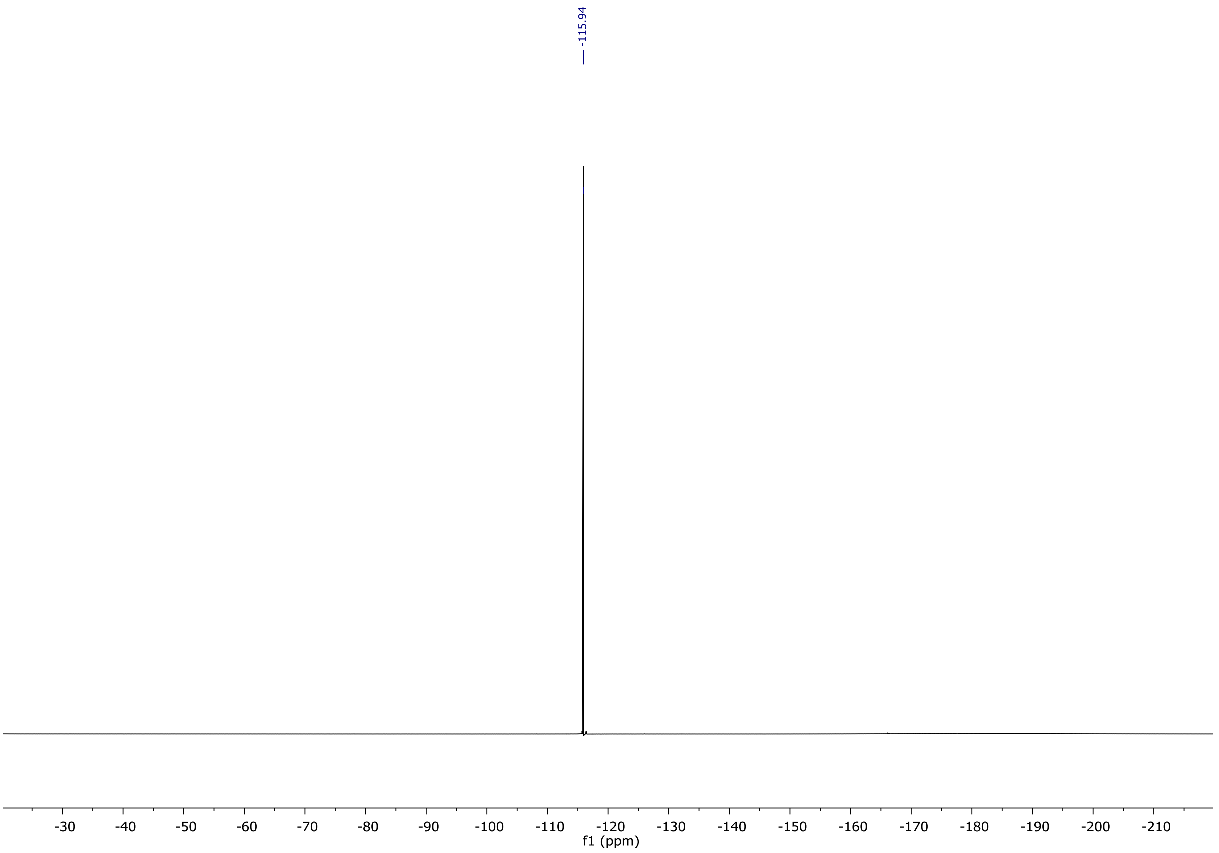

^{1}H NMR of 5i

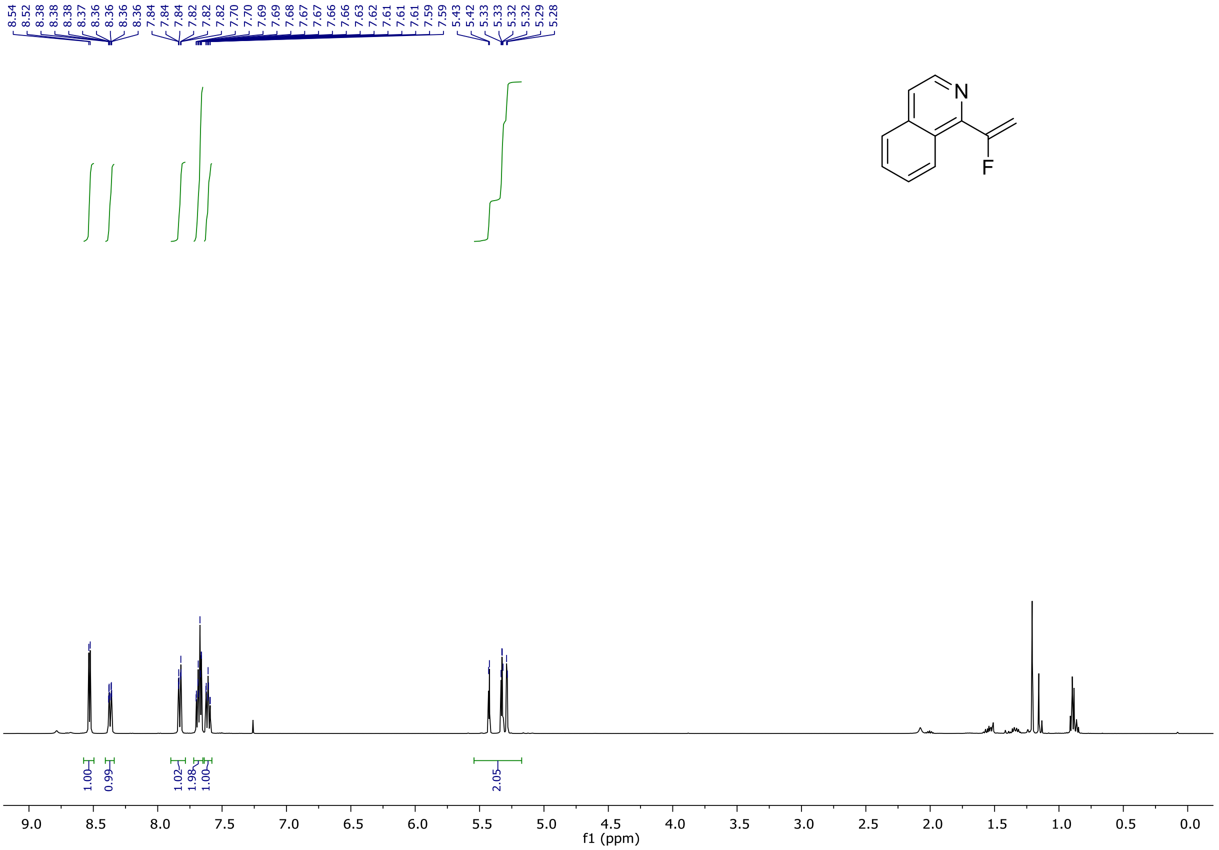

^{13}C NMR of 5i

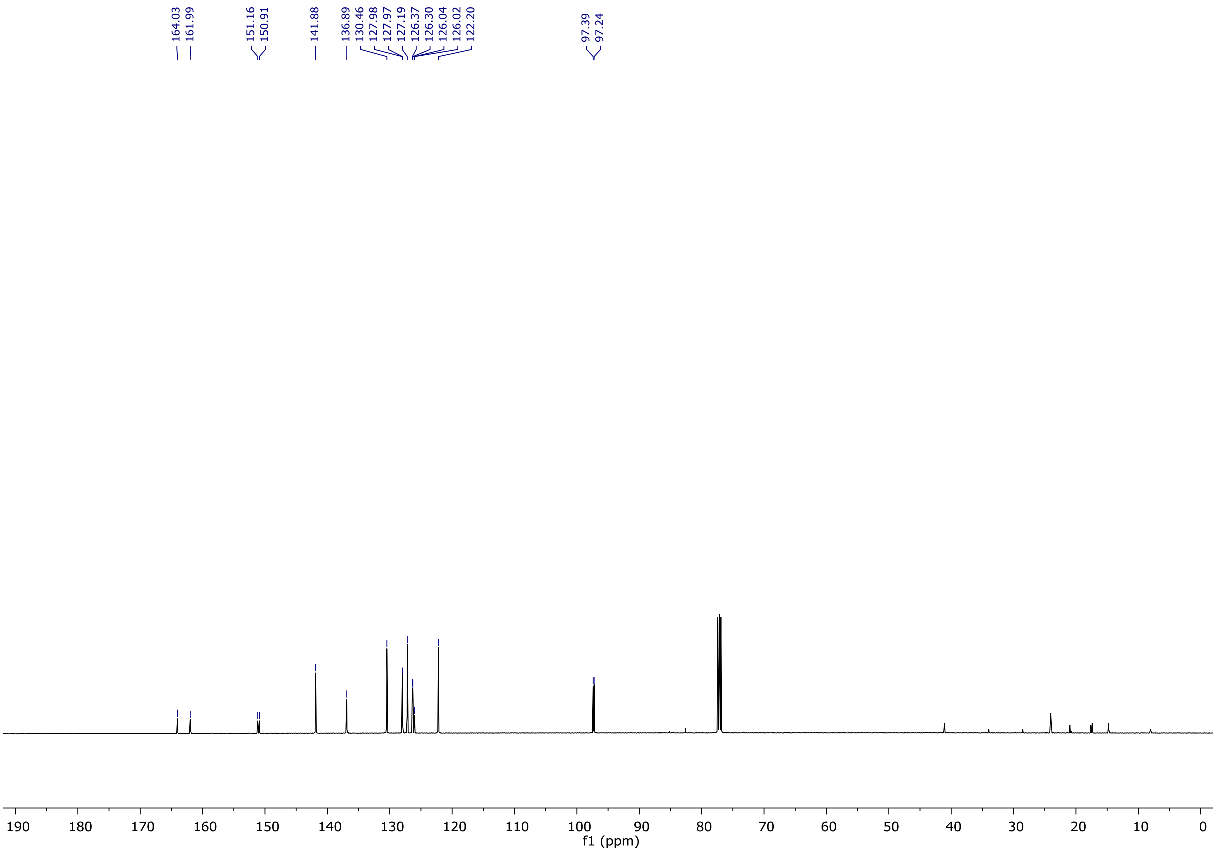

^{19}F NMR of 5i

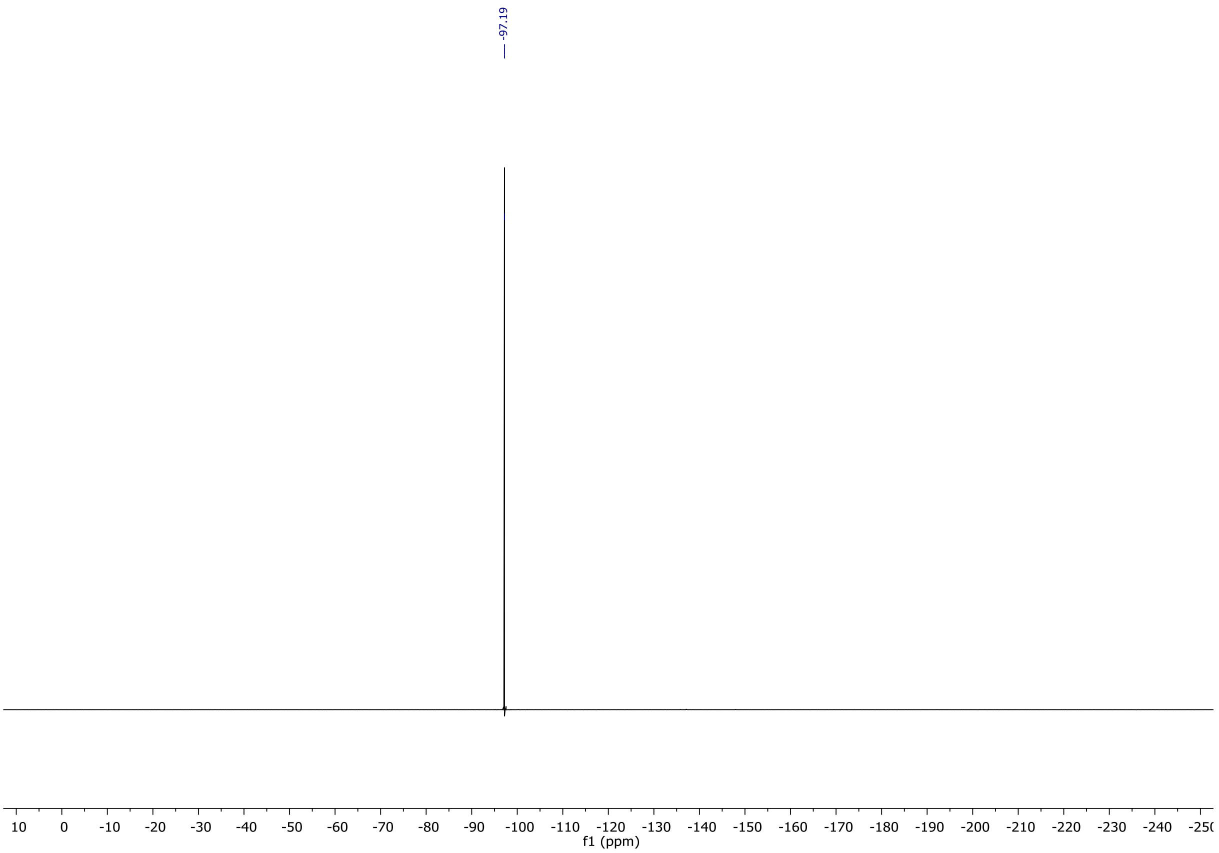

^{1}H NMR of 5j

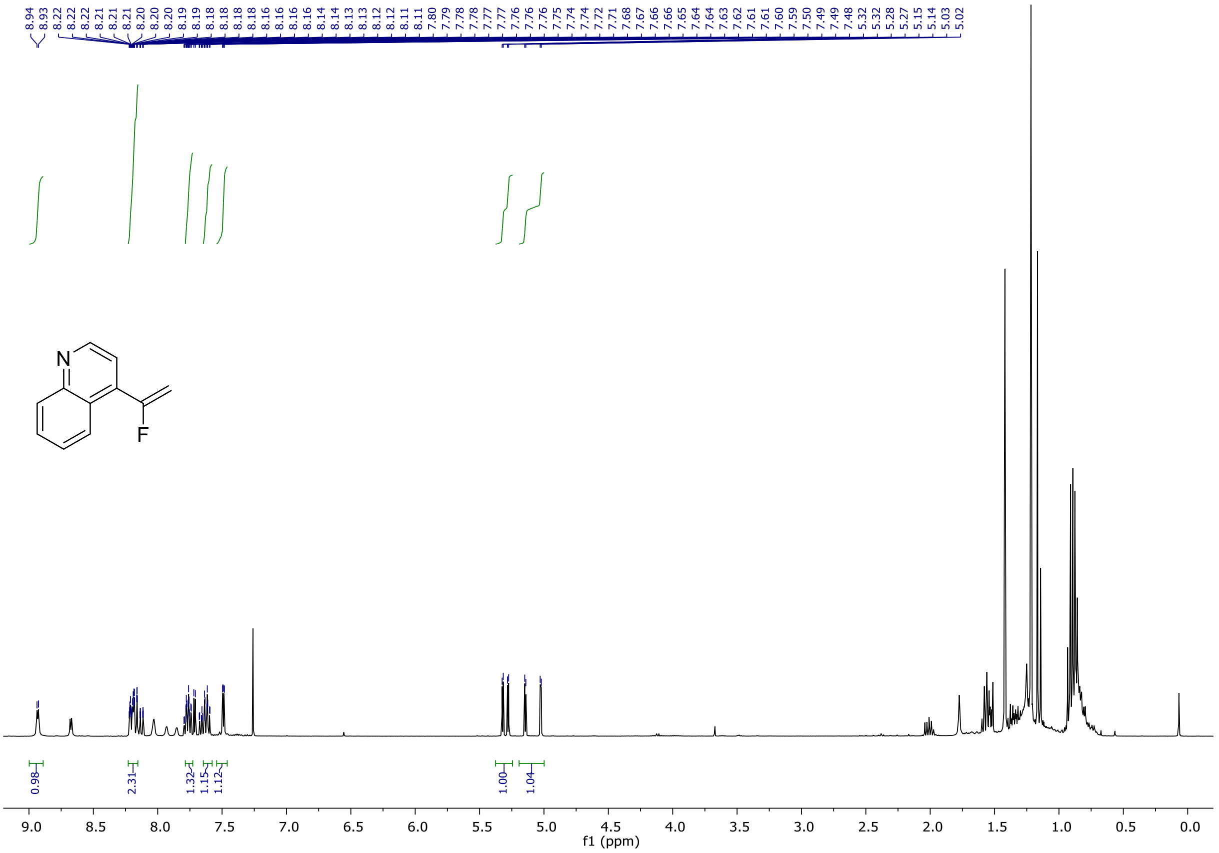

^{13}C NMR of 5j

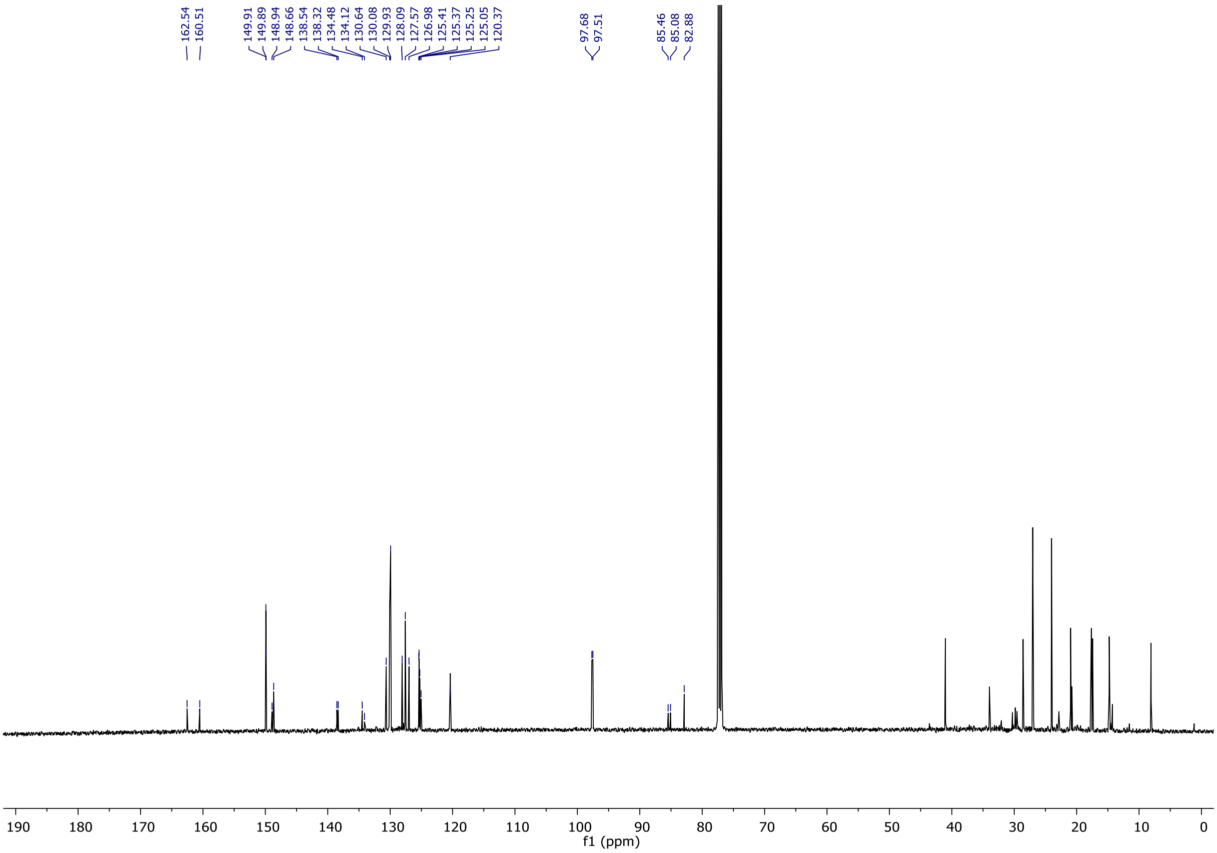

^{19}F NMR of 5j

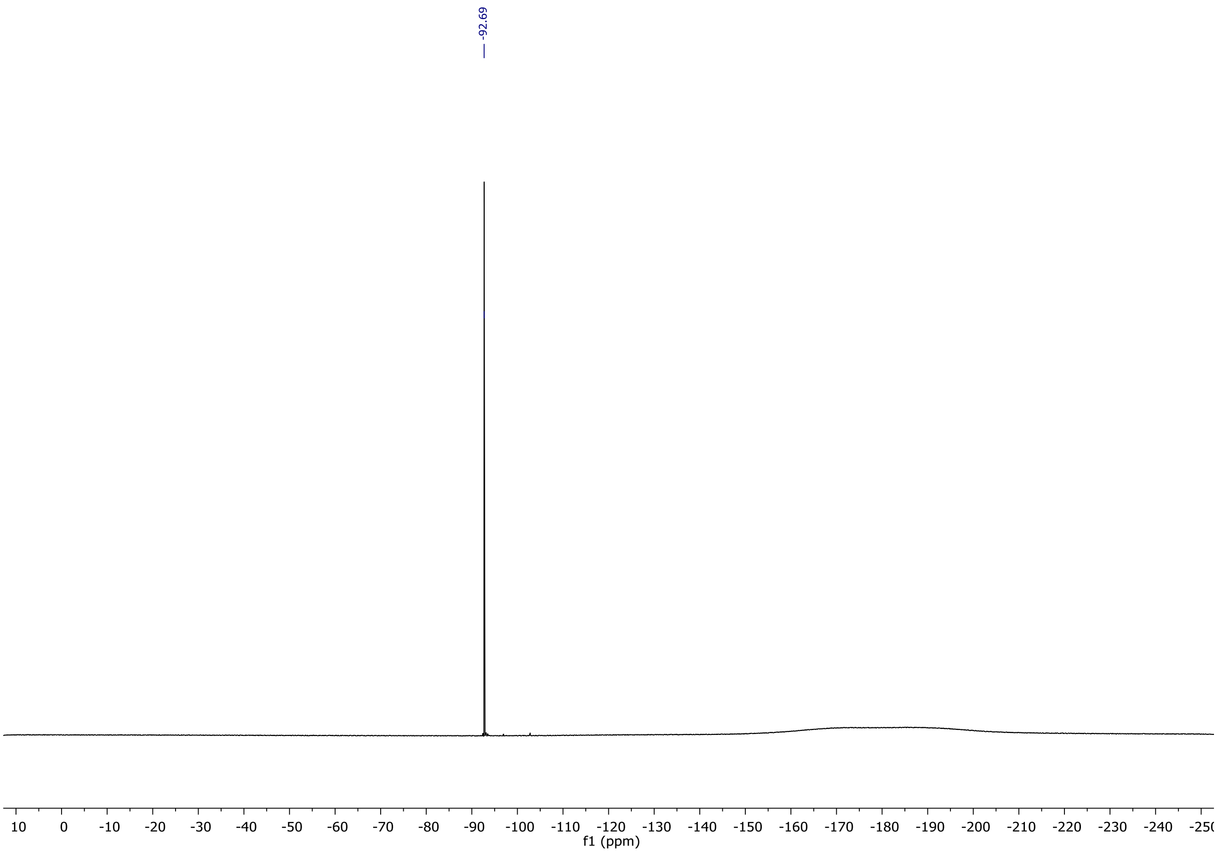

^1H NMR of 5k

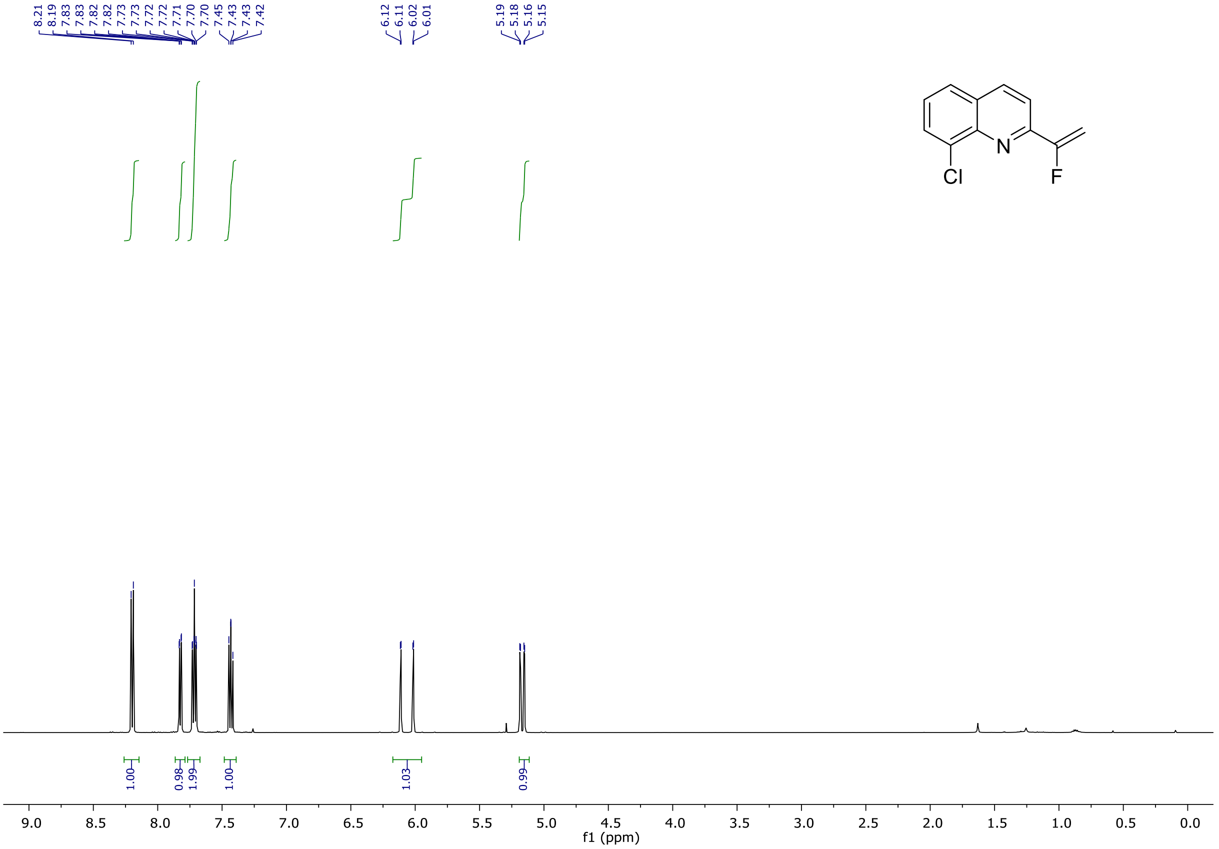

^{13}C NMR of 5k

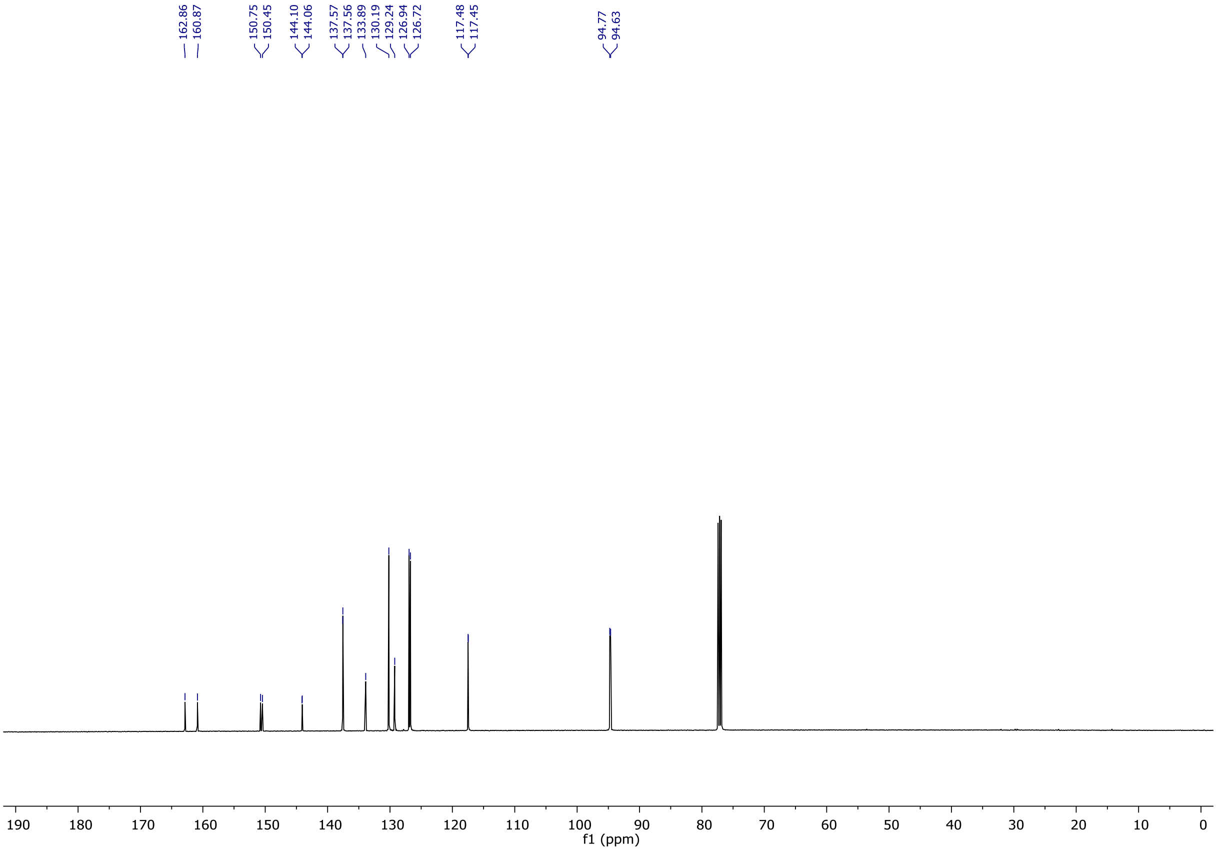

^{19}F NMR of 5k

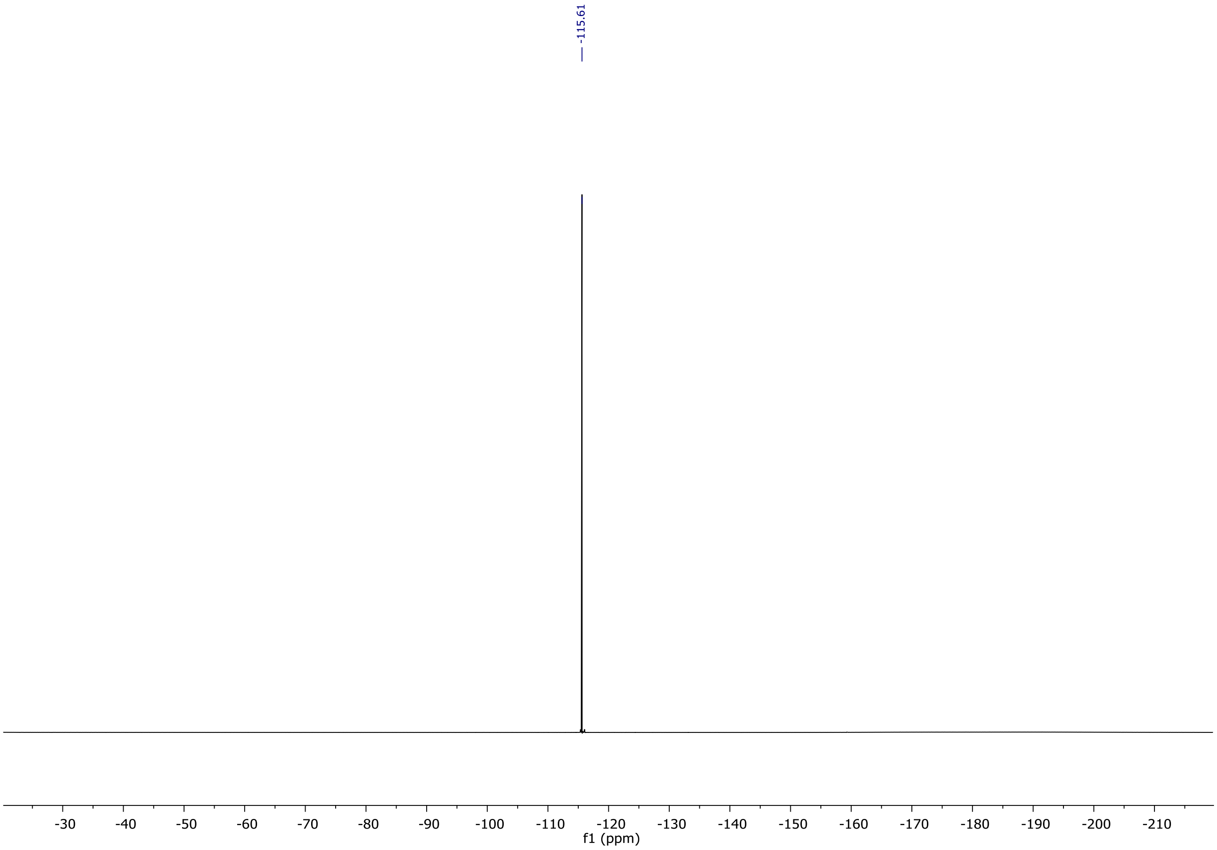

^{1}H NMR of 7a

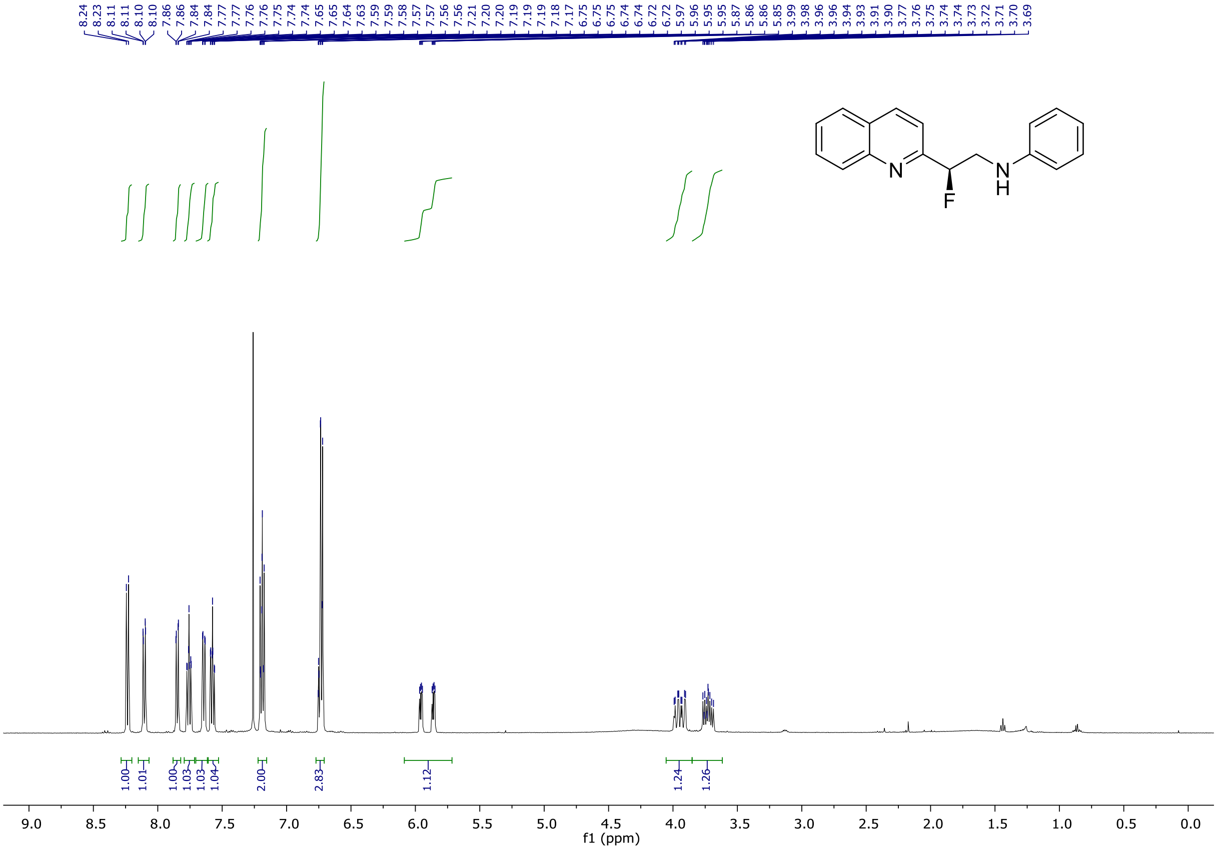

^{13}C NMR of 7a

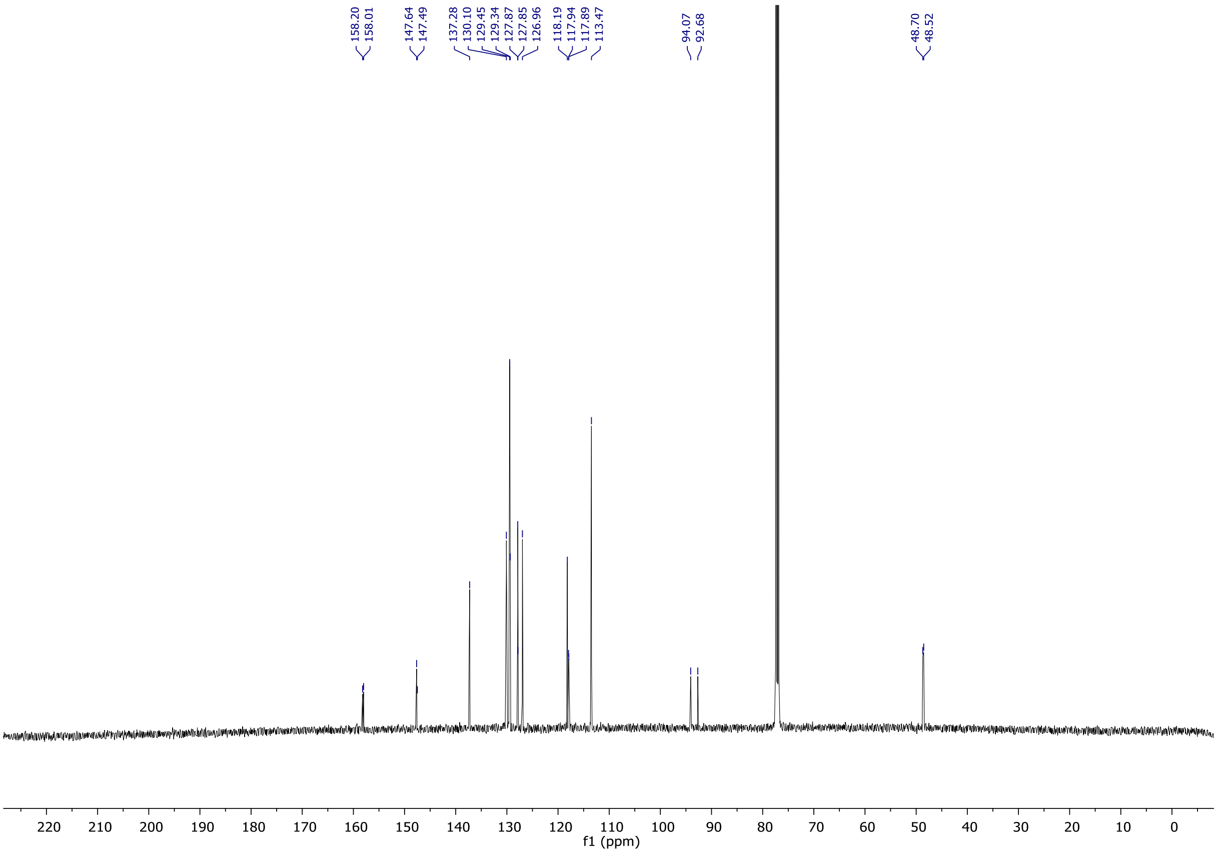

^{19}F NMR of 7a

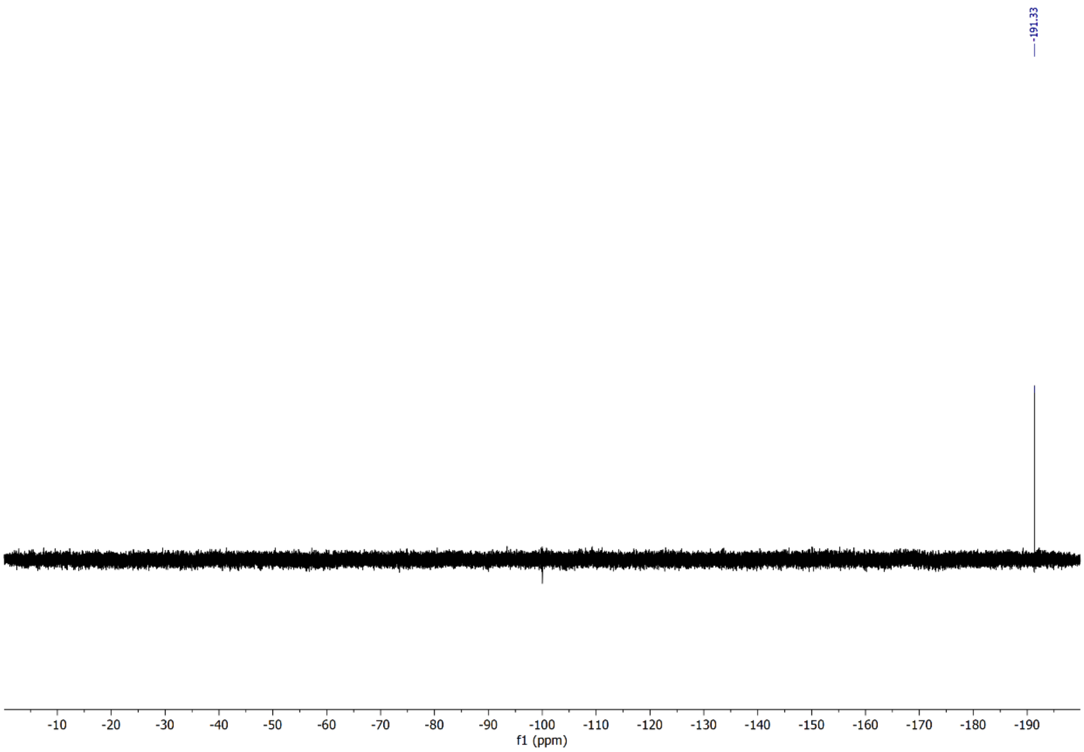

^{1}H NMR of 7b

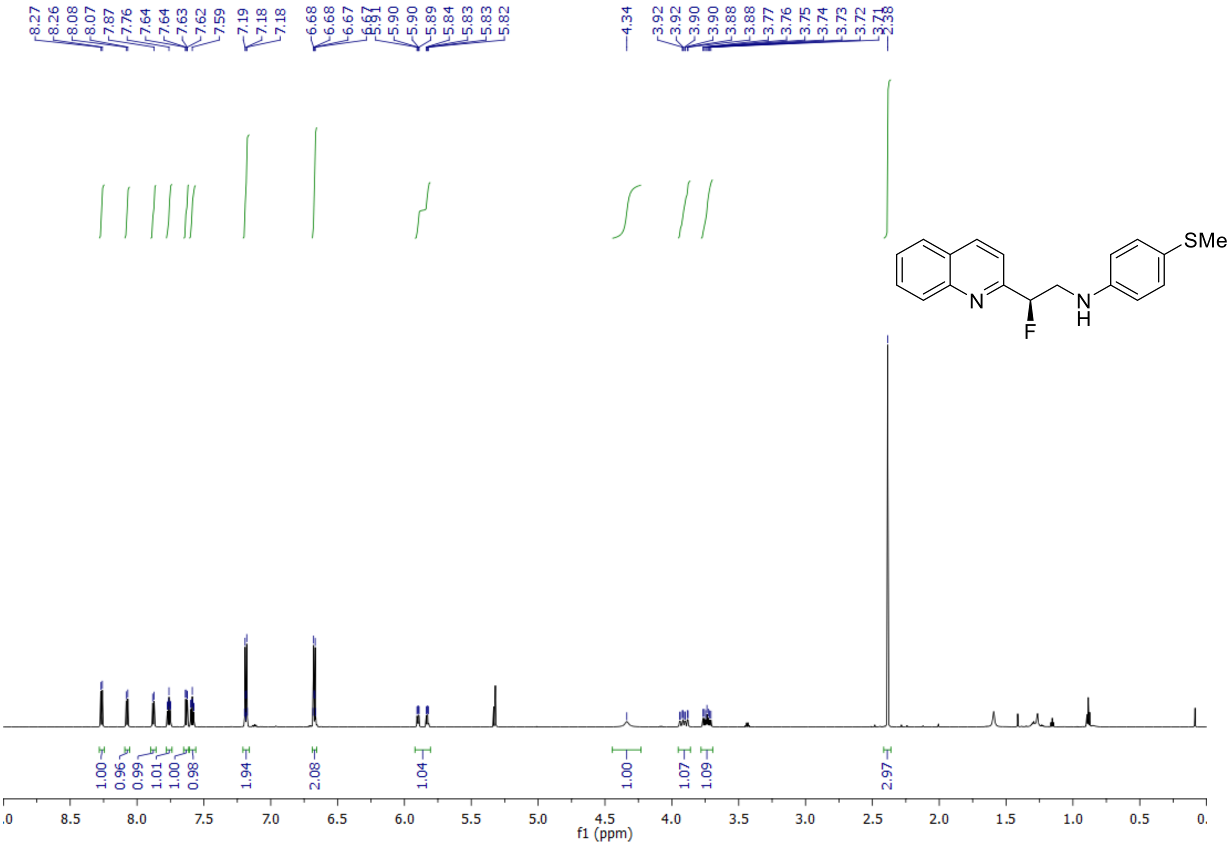

^{13}C NMR of 7b

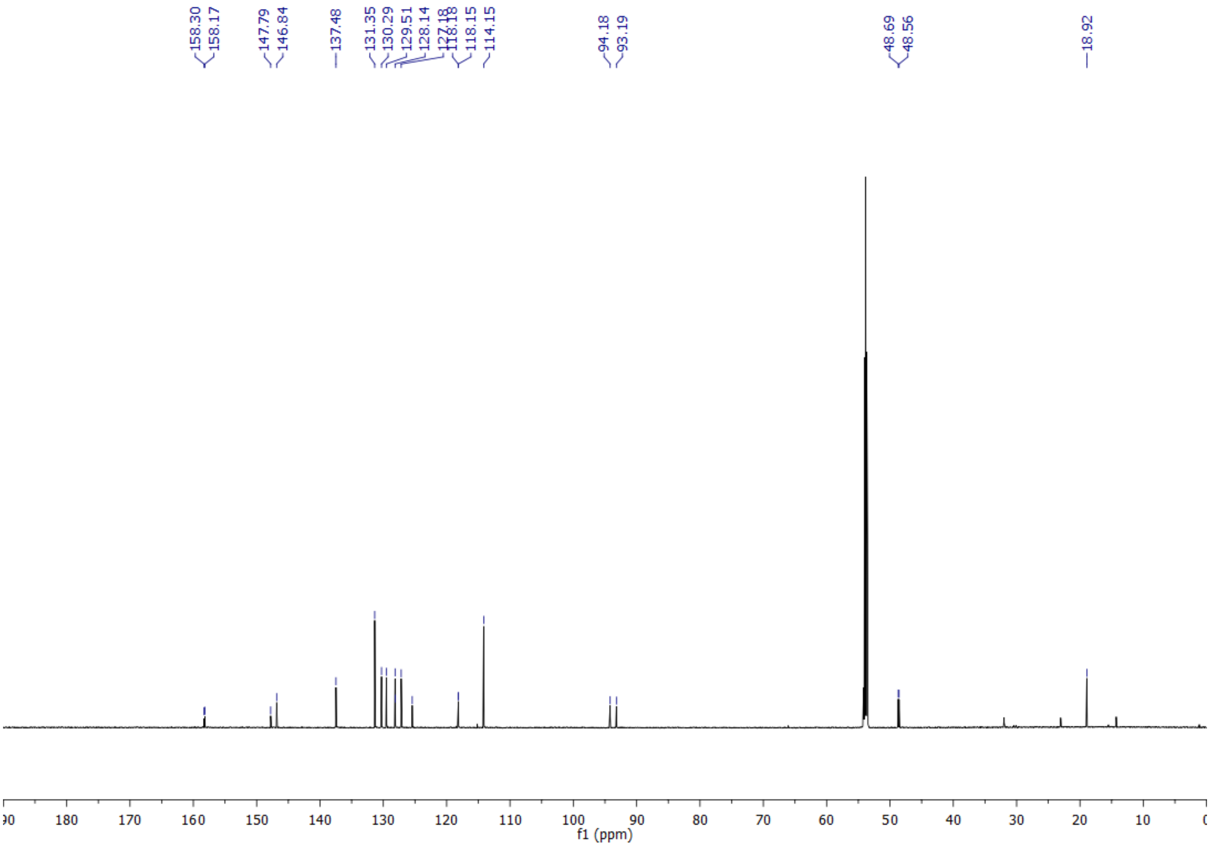

^{19}F NMR of 7b

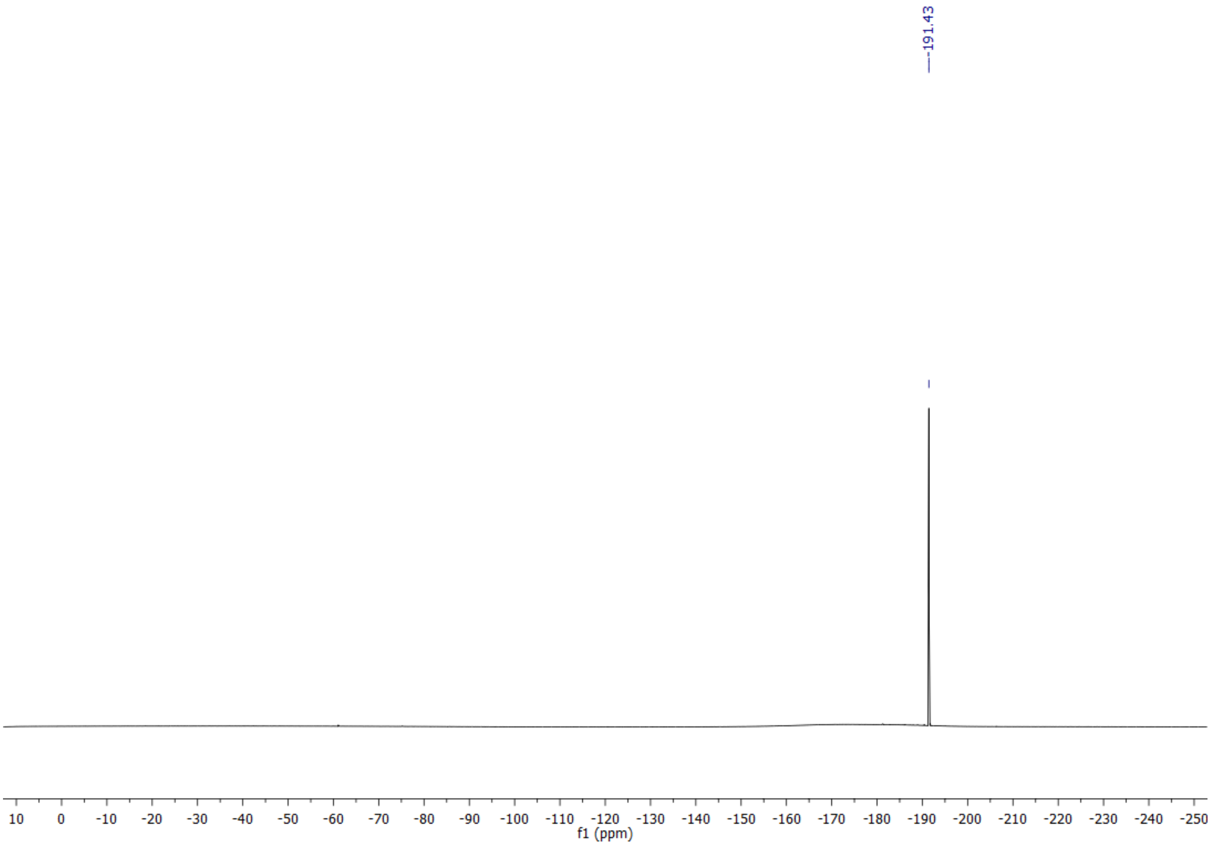

^{1}H NMR of 7c

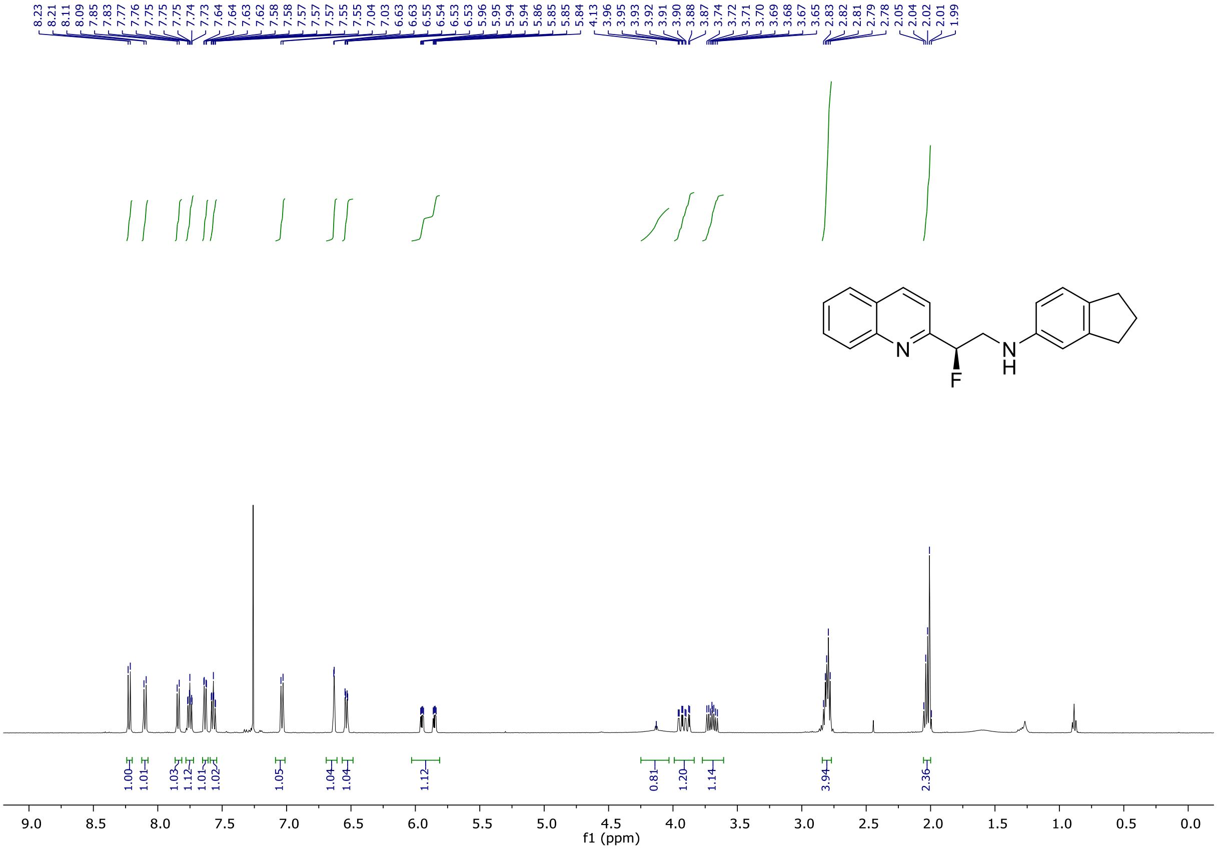

^{13}C NMR of 7c

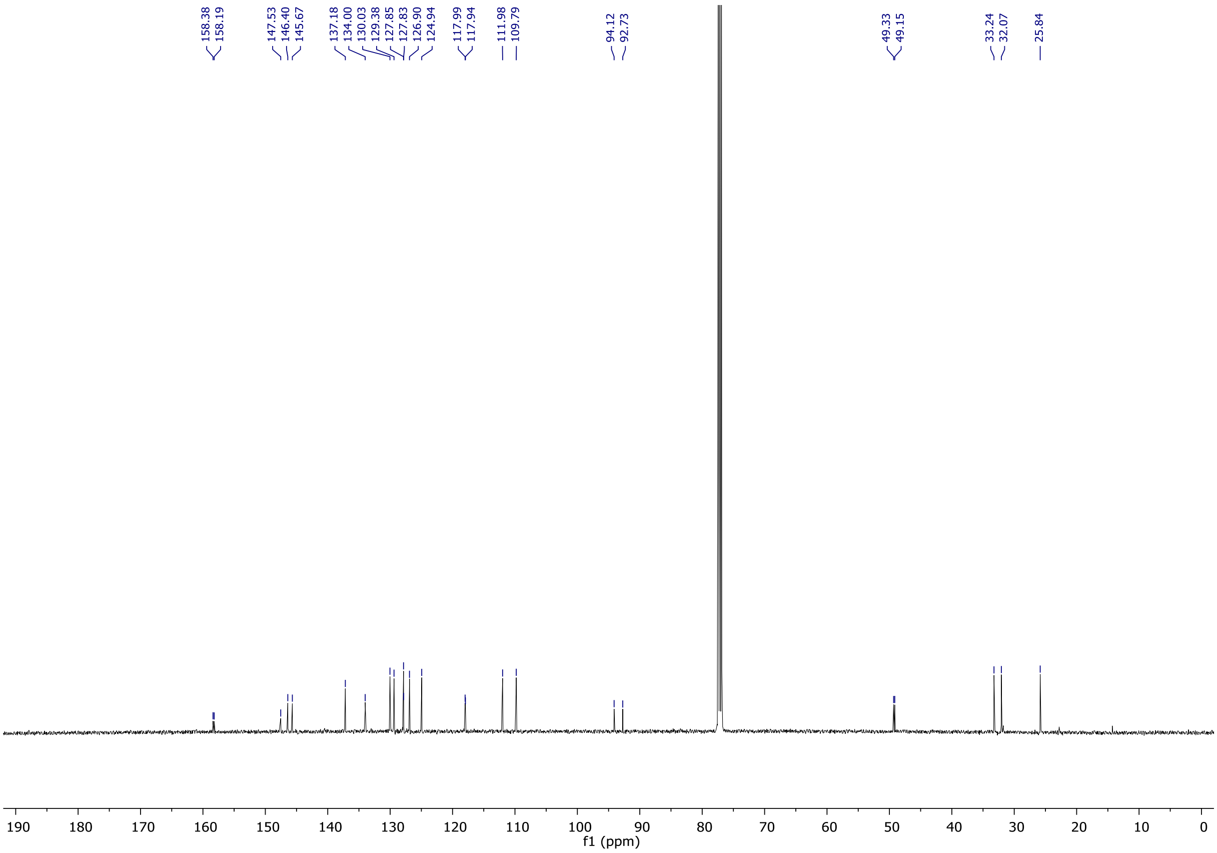

^{19}F NMR of 7c

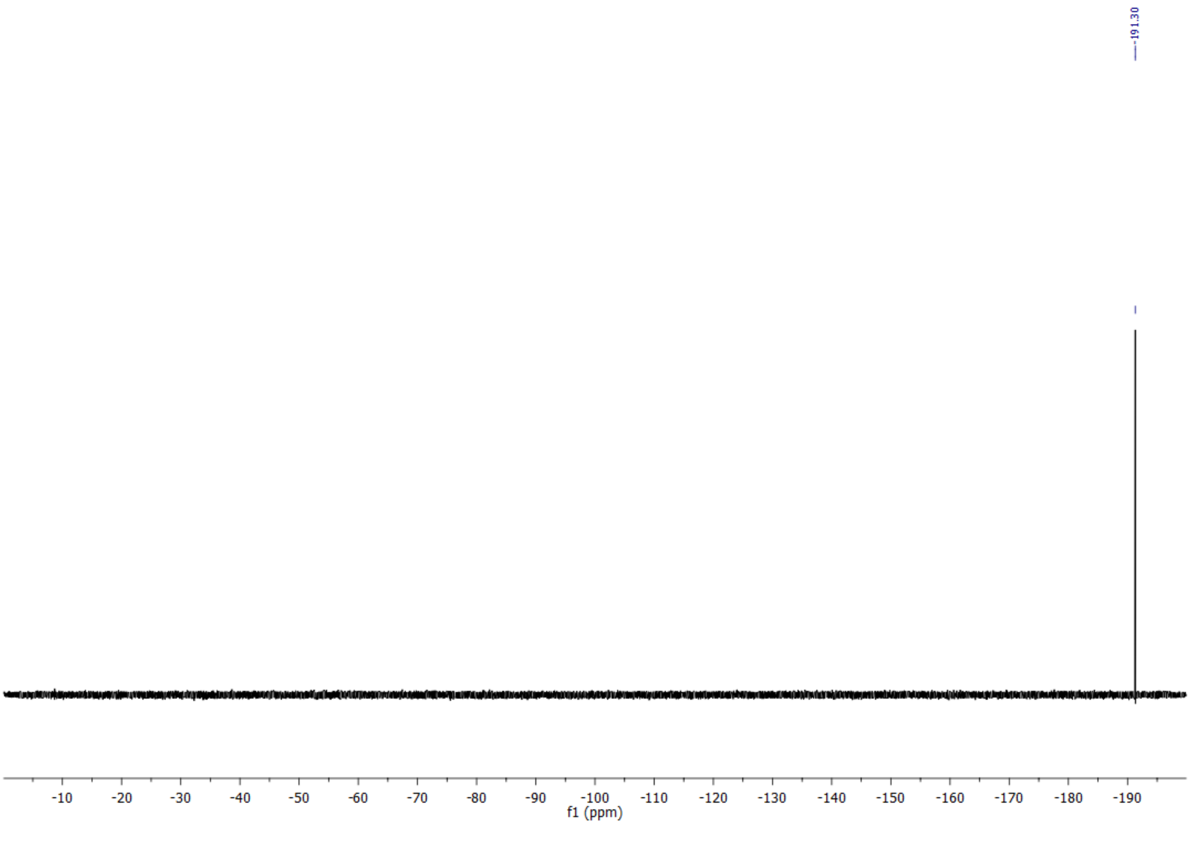

^{1}H NMR of 7d

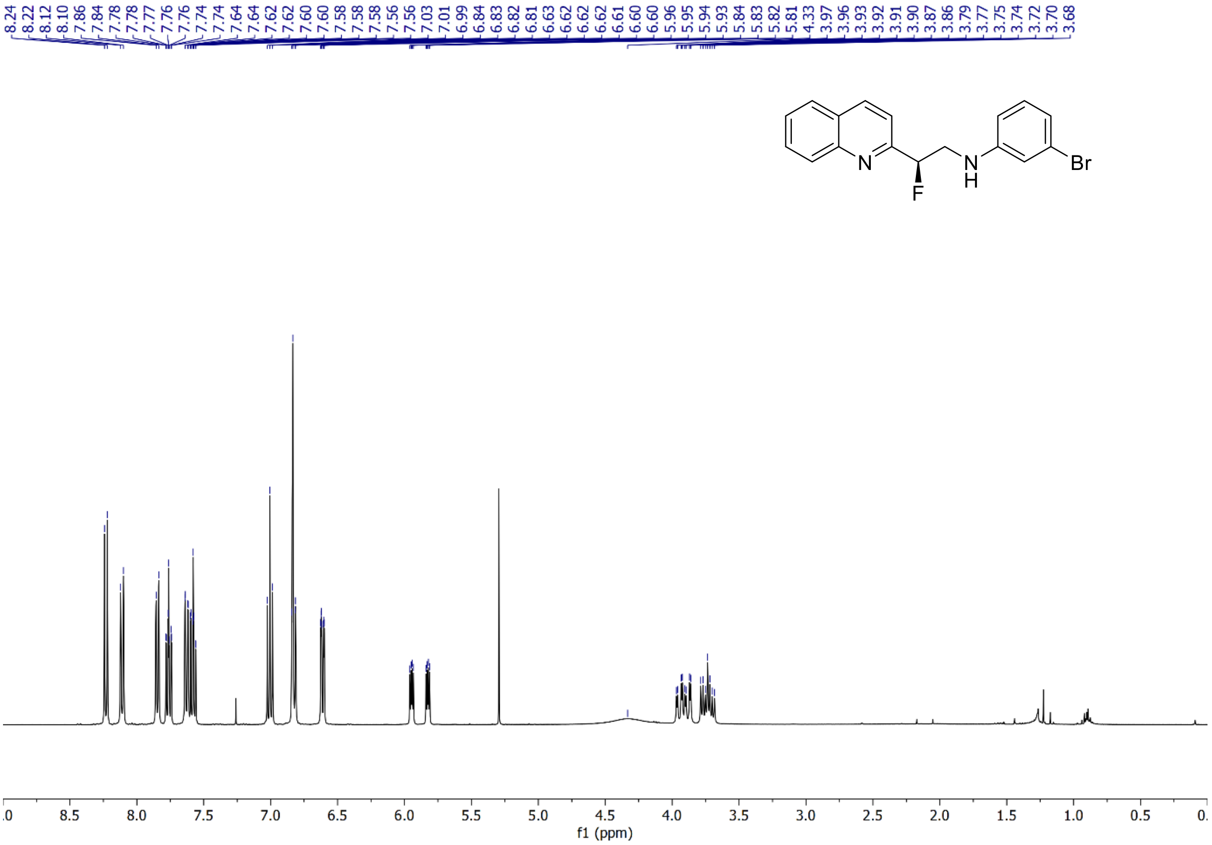

^{13}C NMR of 7d

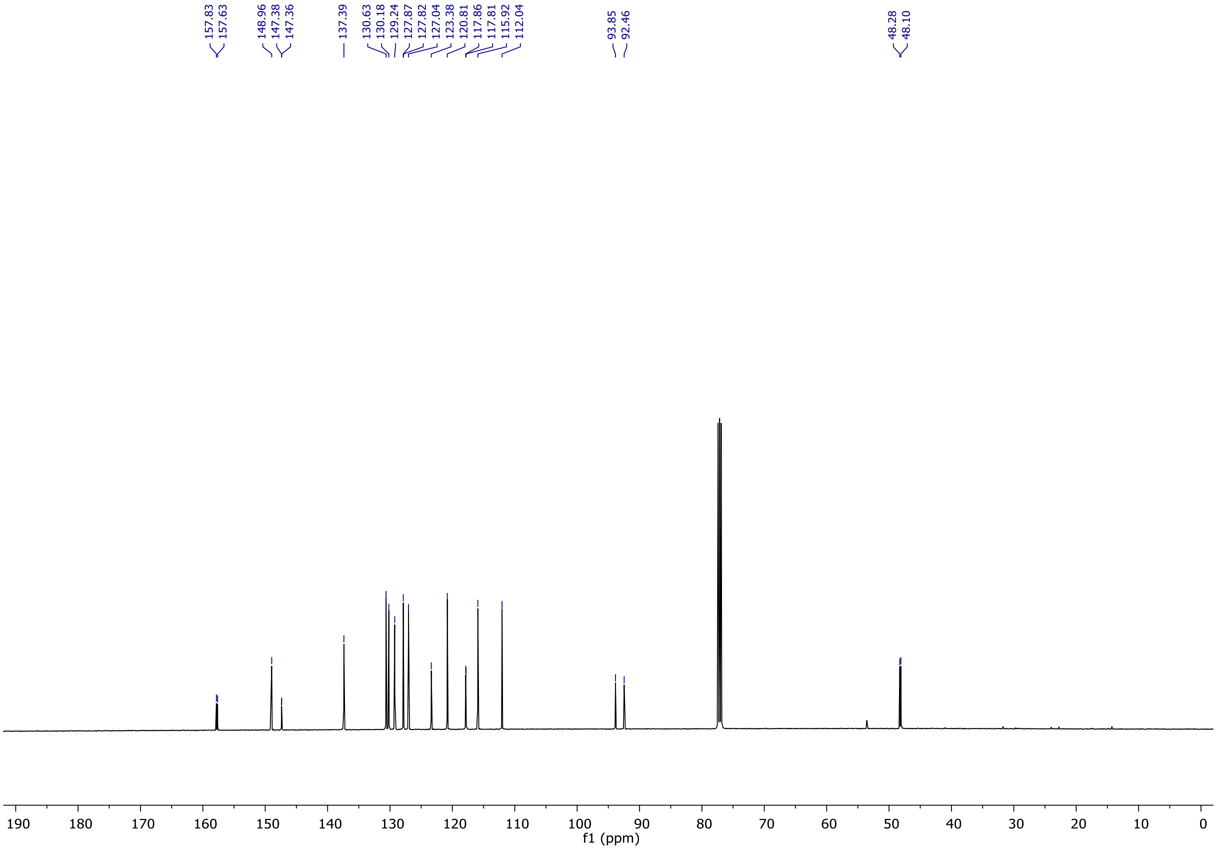

^{19}F NMR of 7d

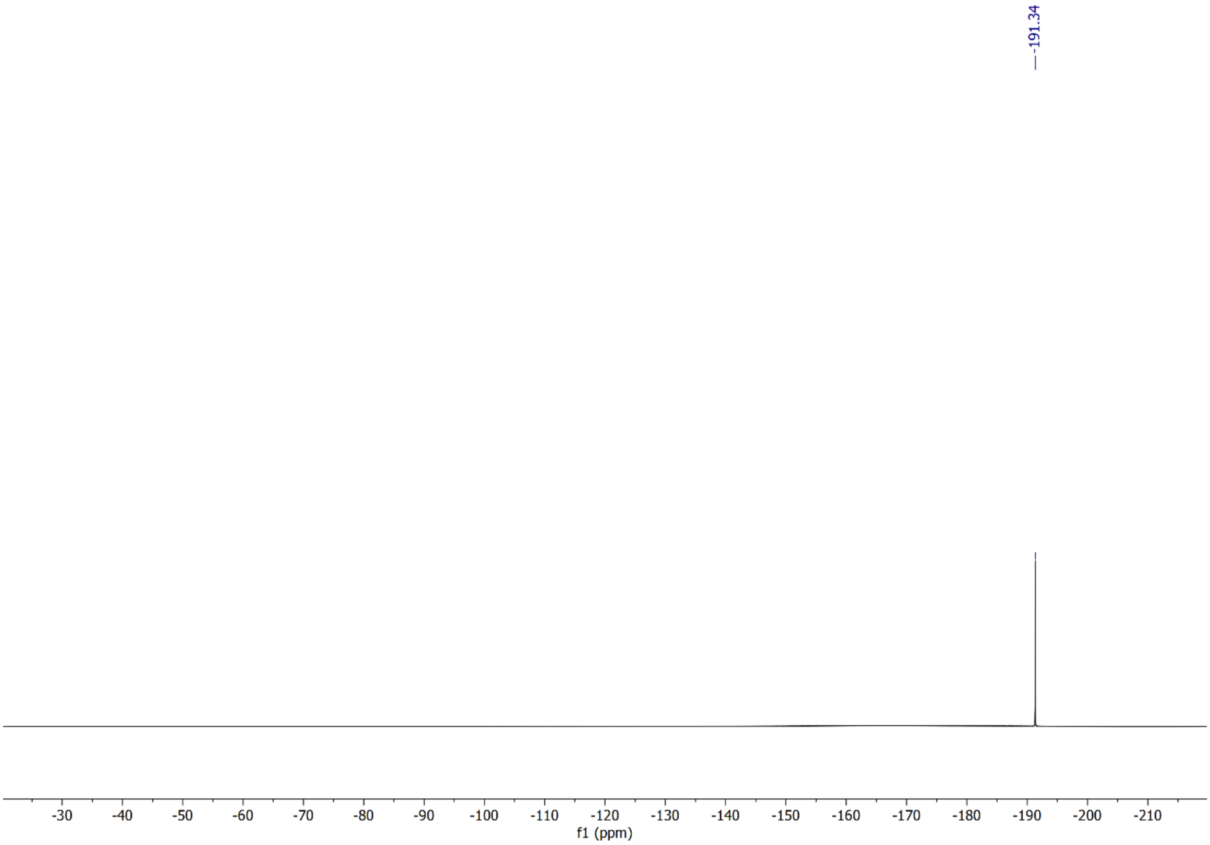

^{1}H NMR of 7e

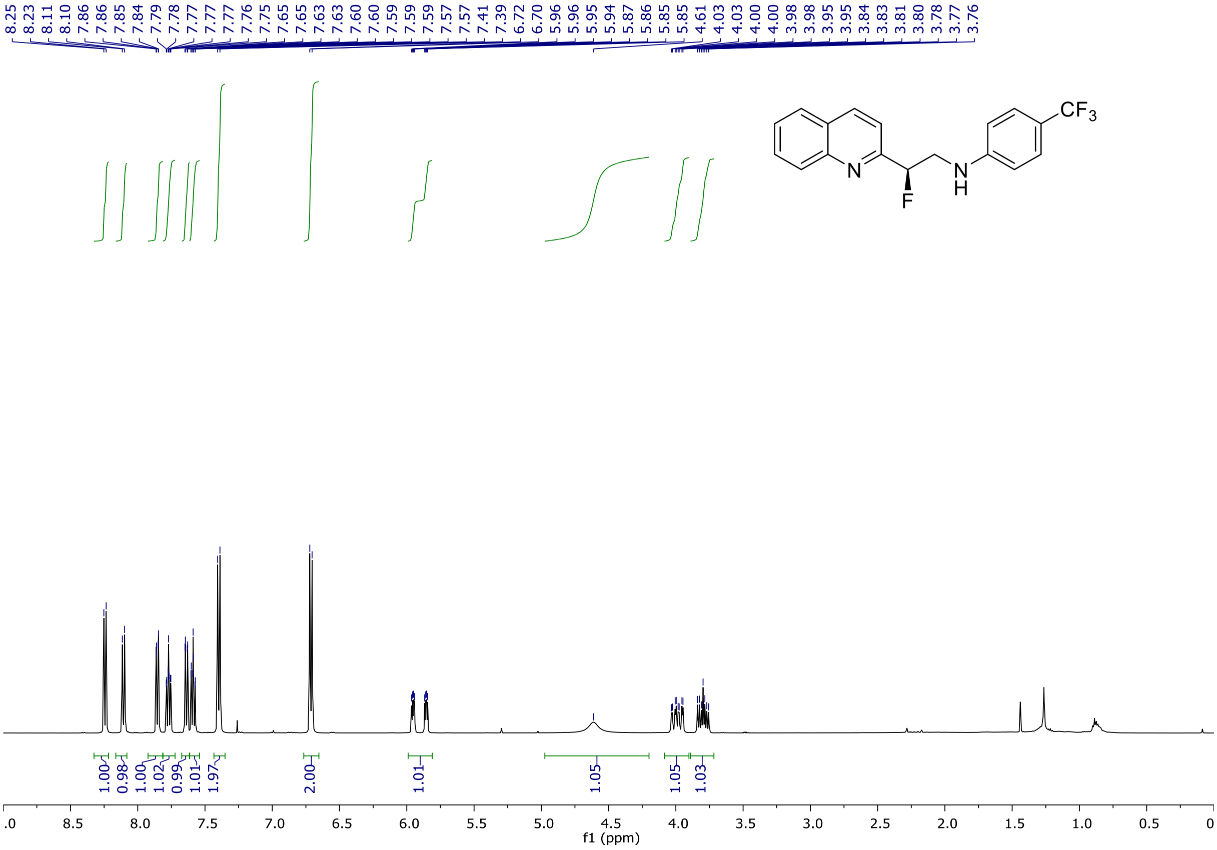

^{13}C NMR of 7e

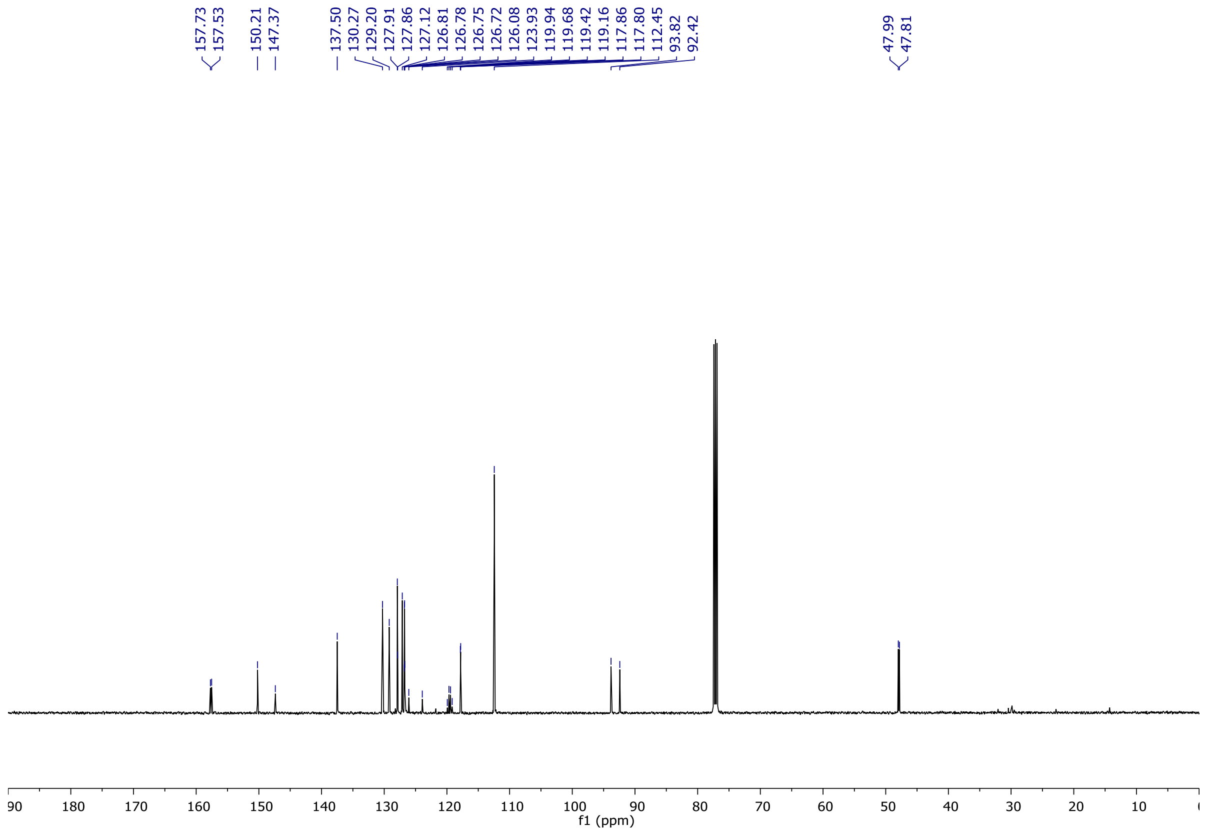

^{19}F NMR of 7e

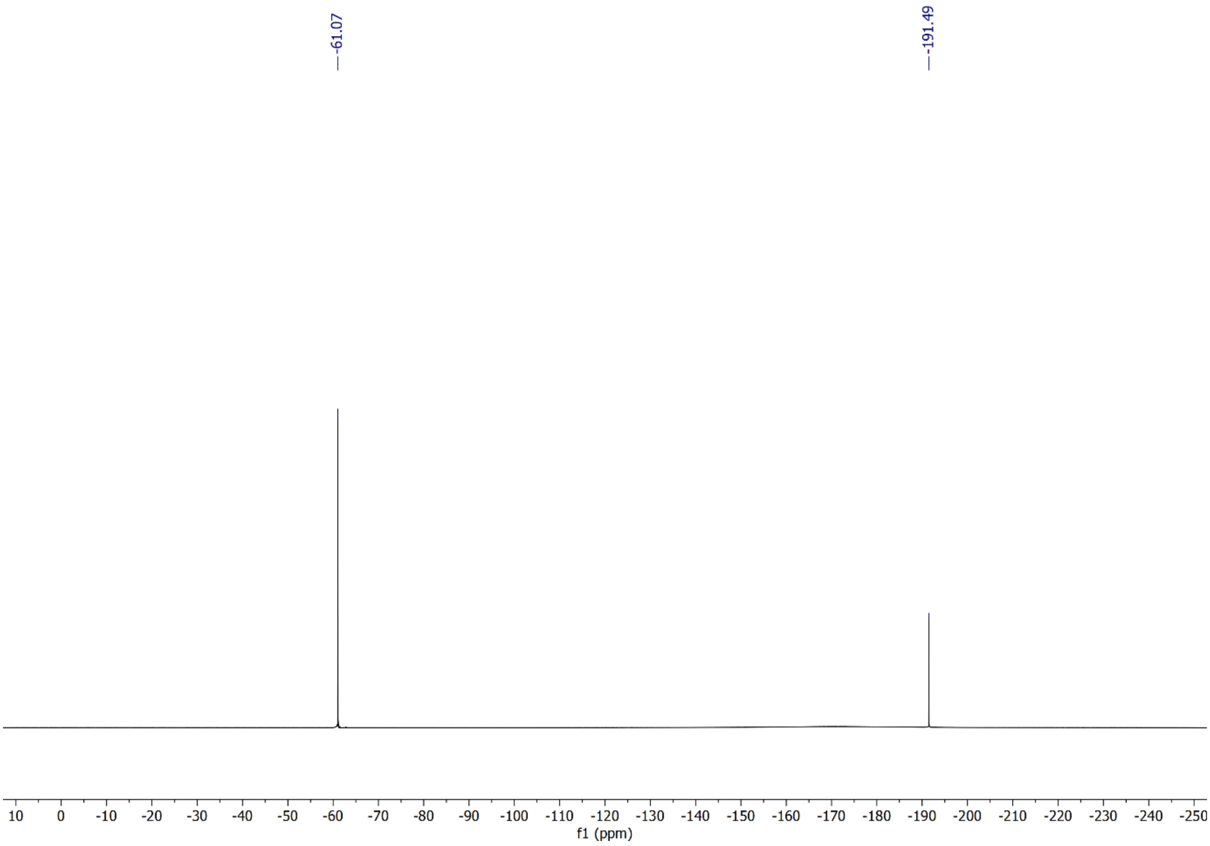

^{1}H NMR of 7f

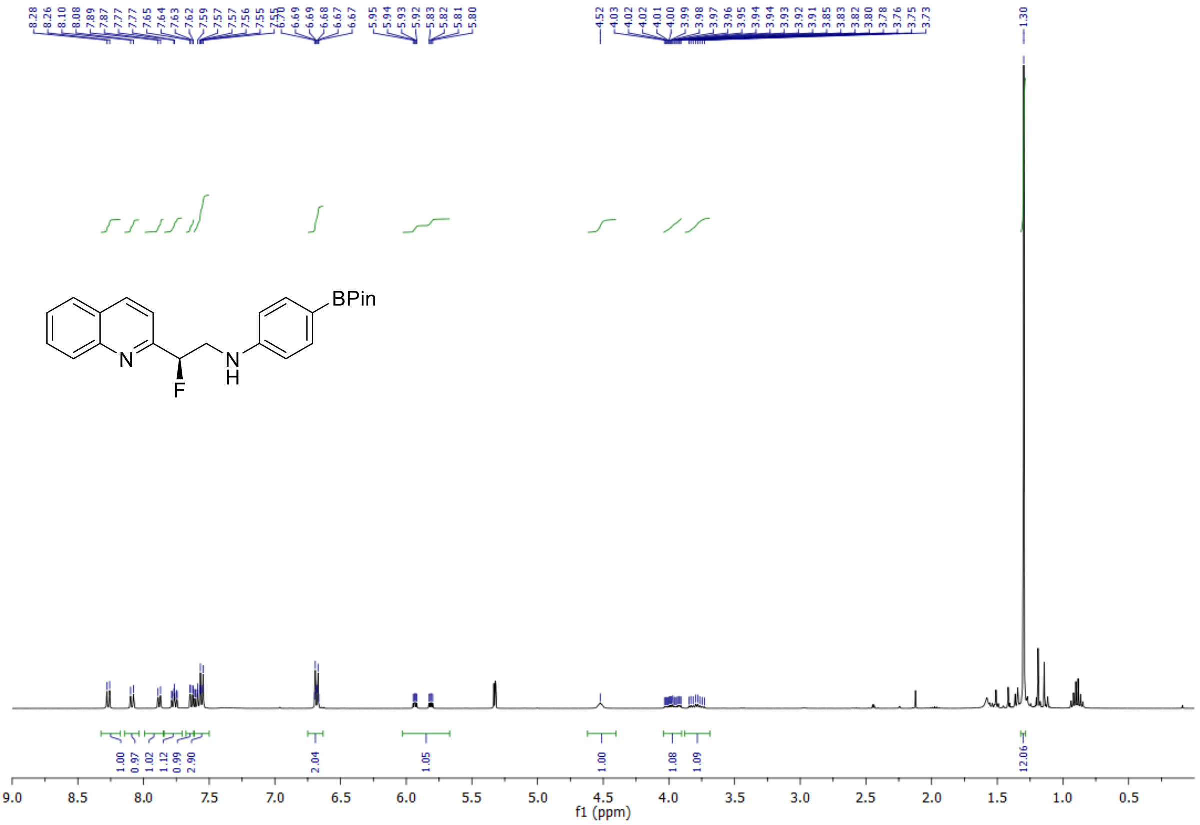

^{13}C NMR of 7f

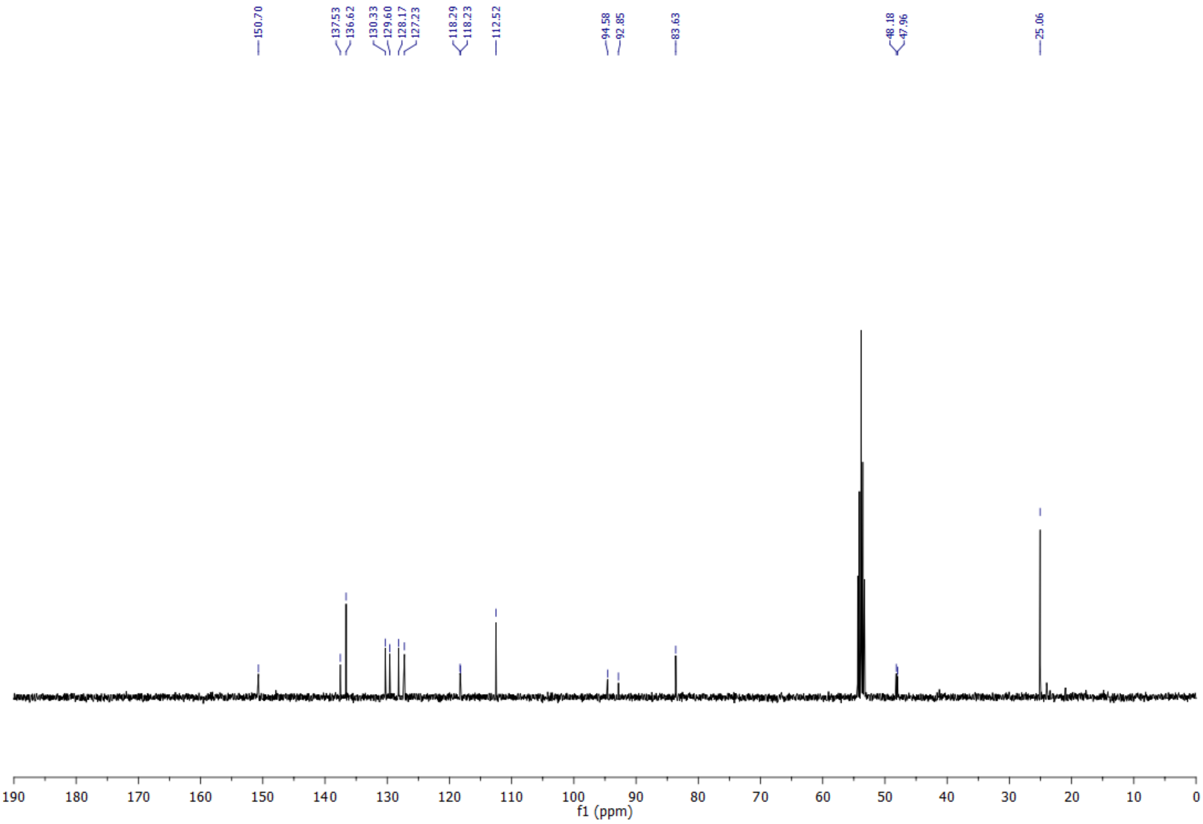

^{19}F NMR of 7f

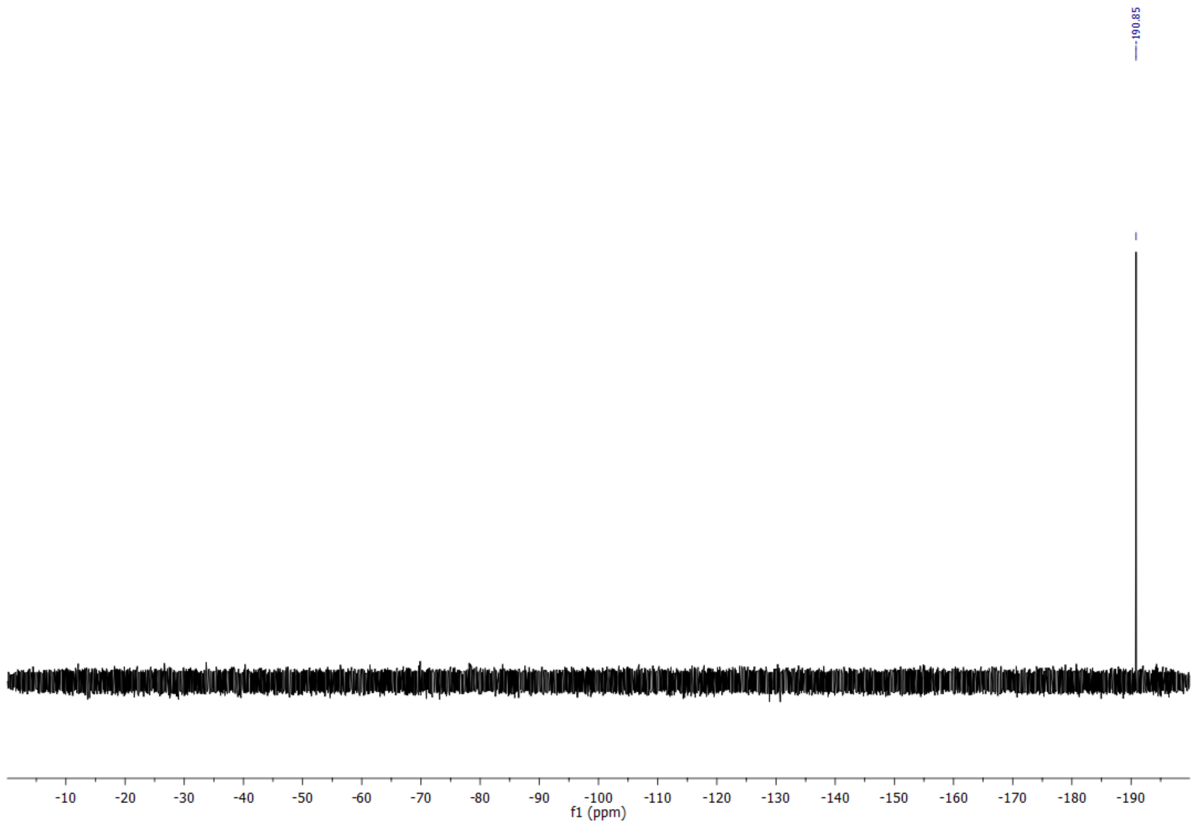

^{11} B NMR of 7f

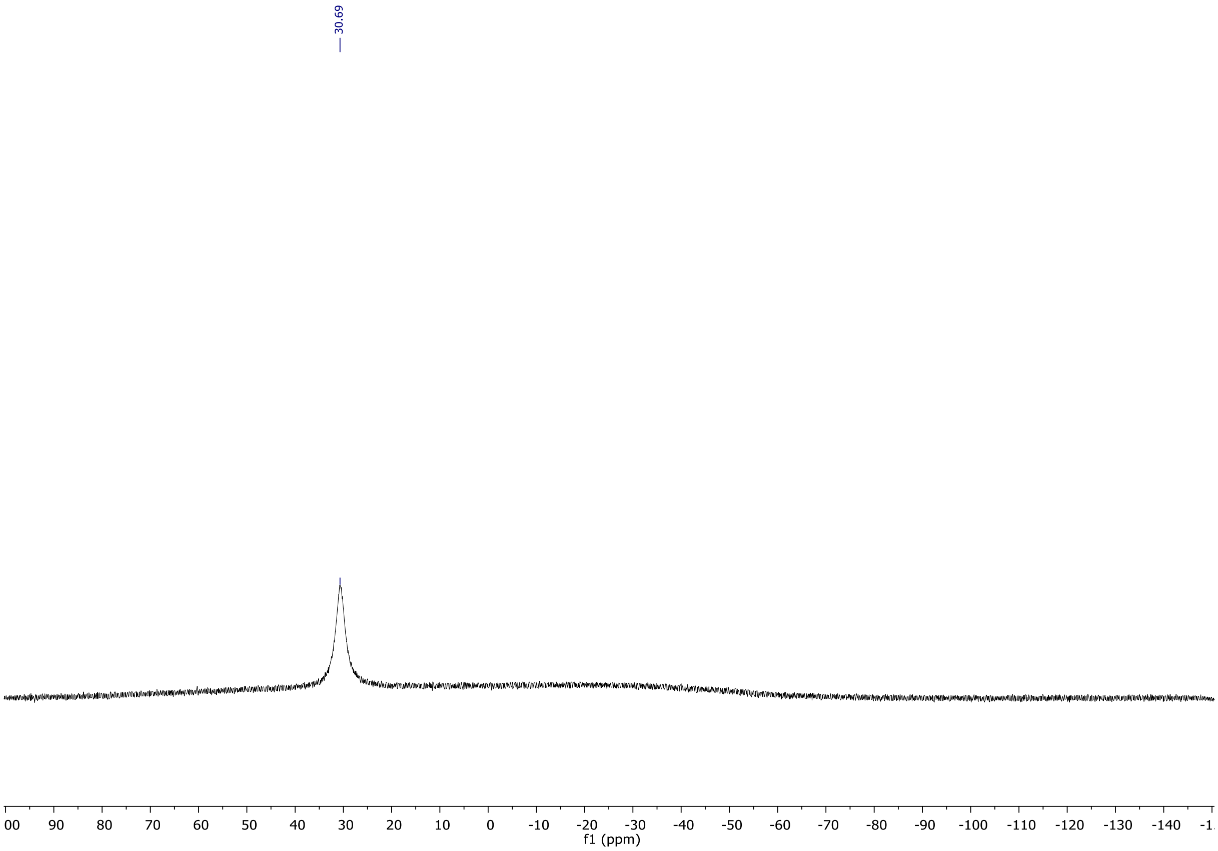

^{1}H NMR of 7g

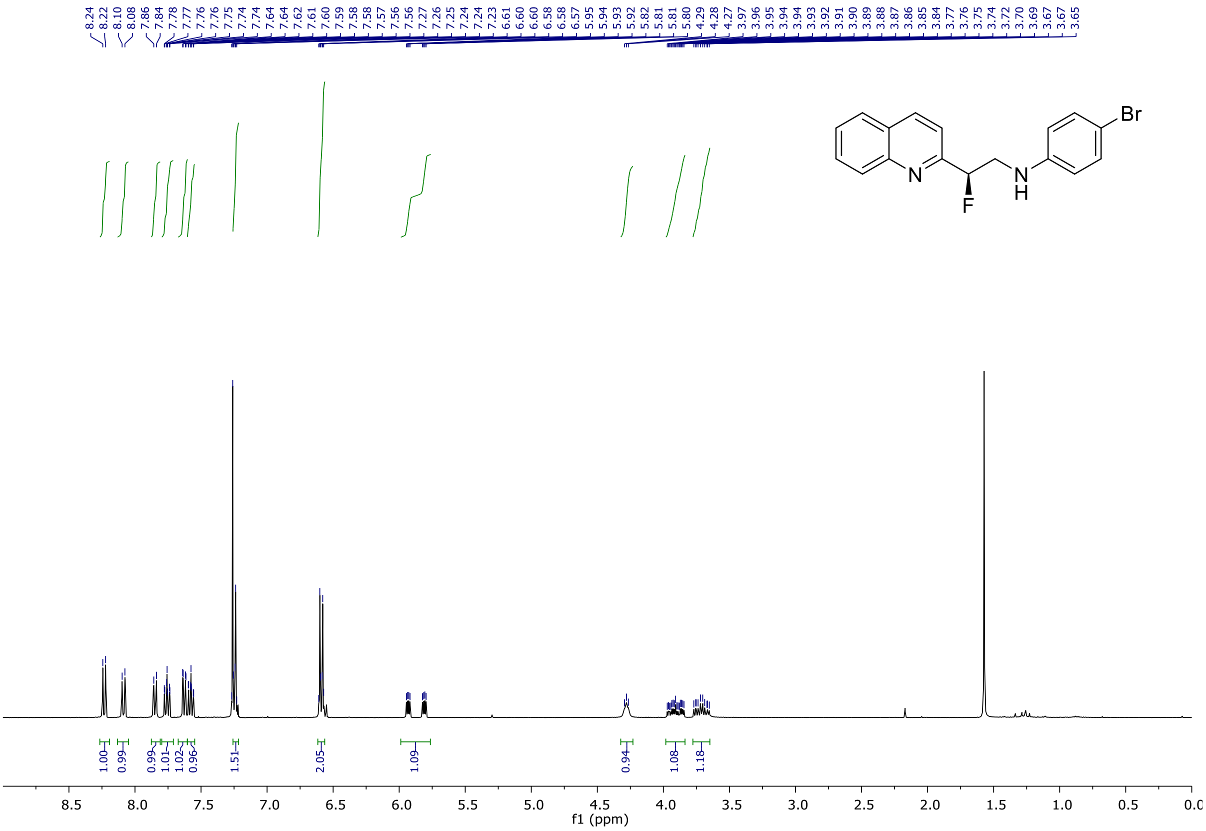

^{13}C NMR of 7g

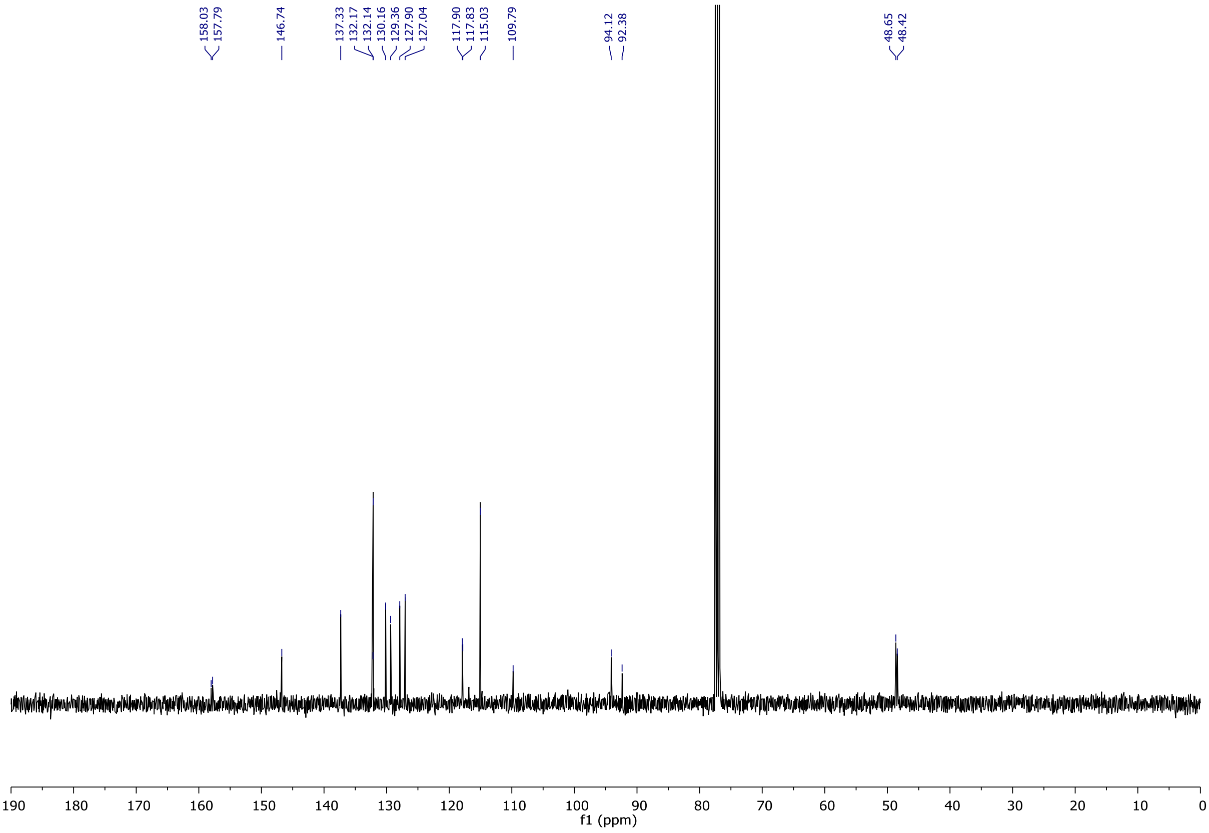

^{19}F NMR of 7g

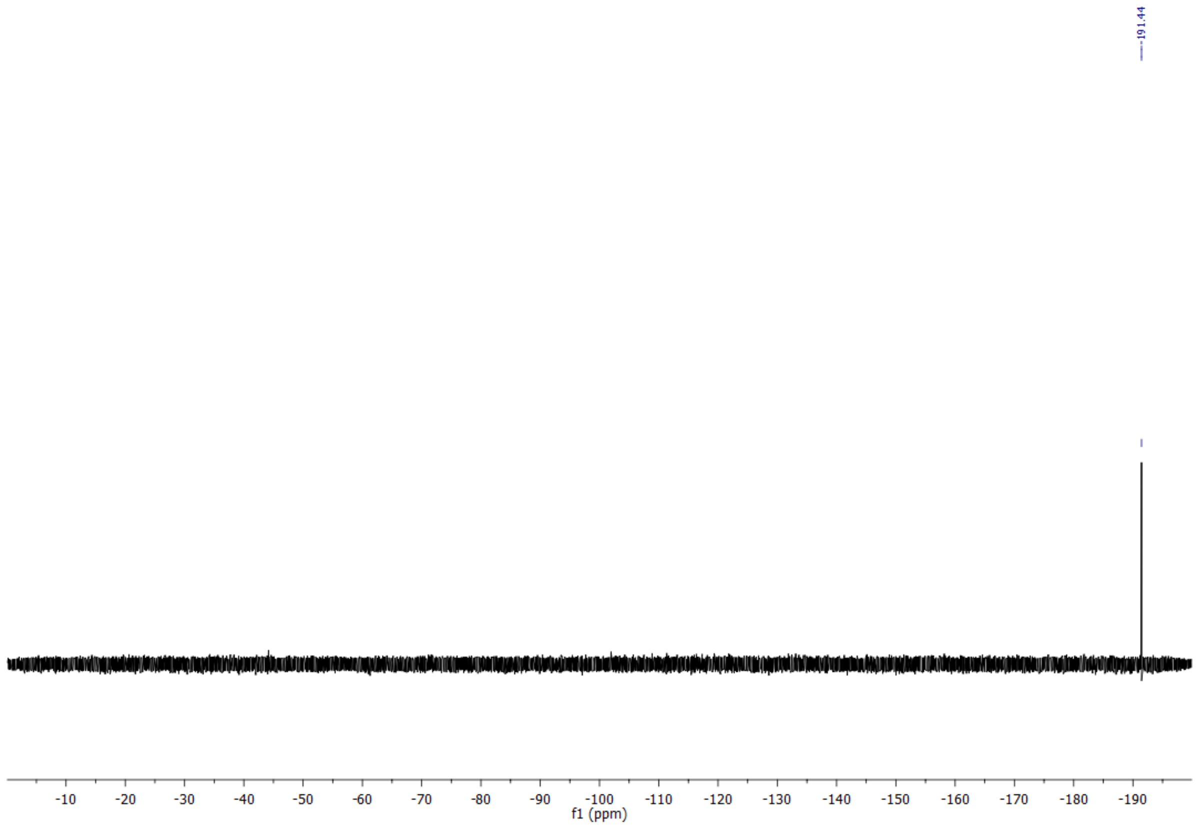

^{1}H NMR of 7h

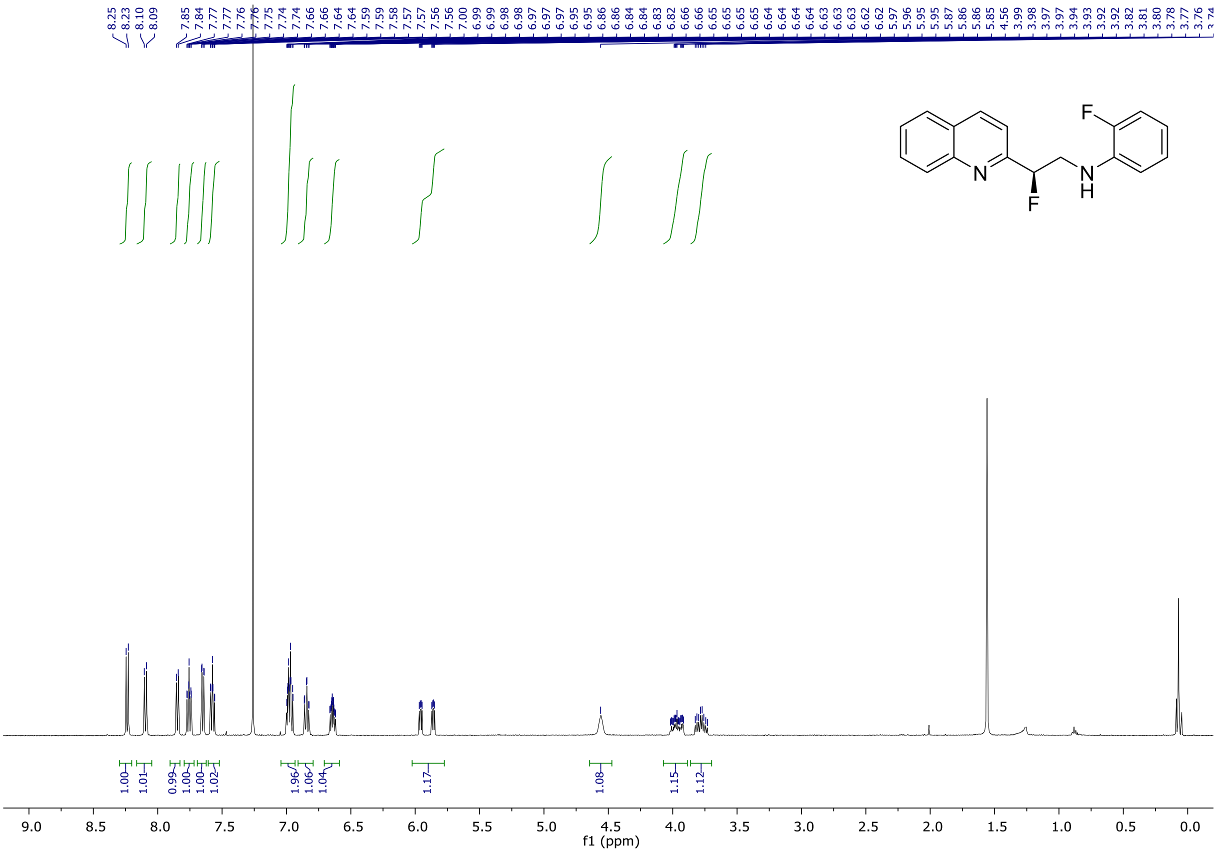

^{13}C NMR of 7h

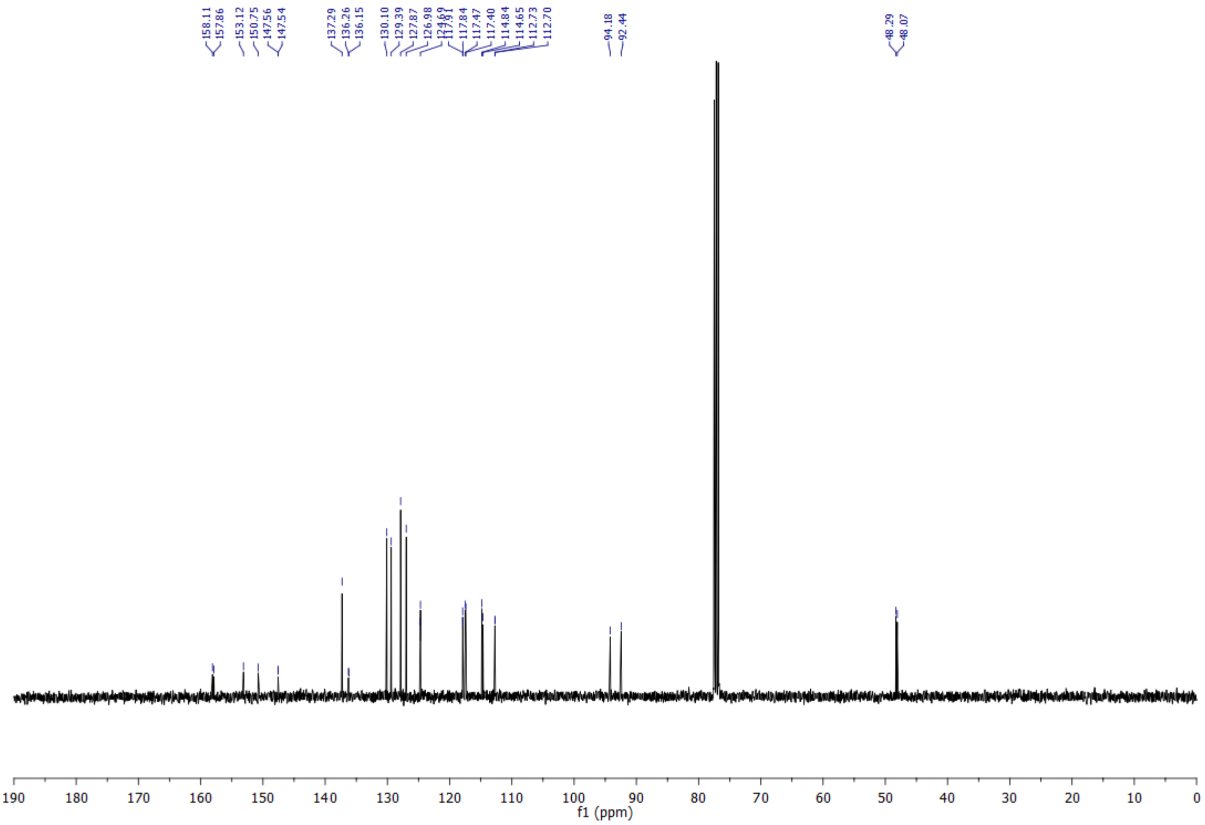

^{19}F NMR of 7h

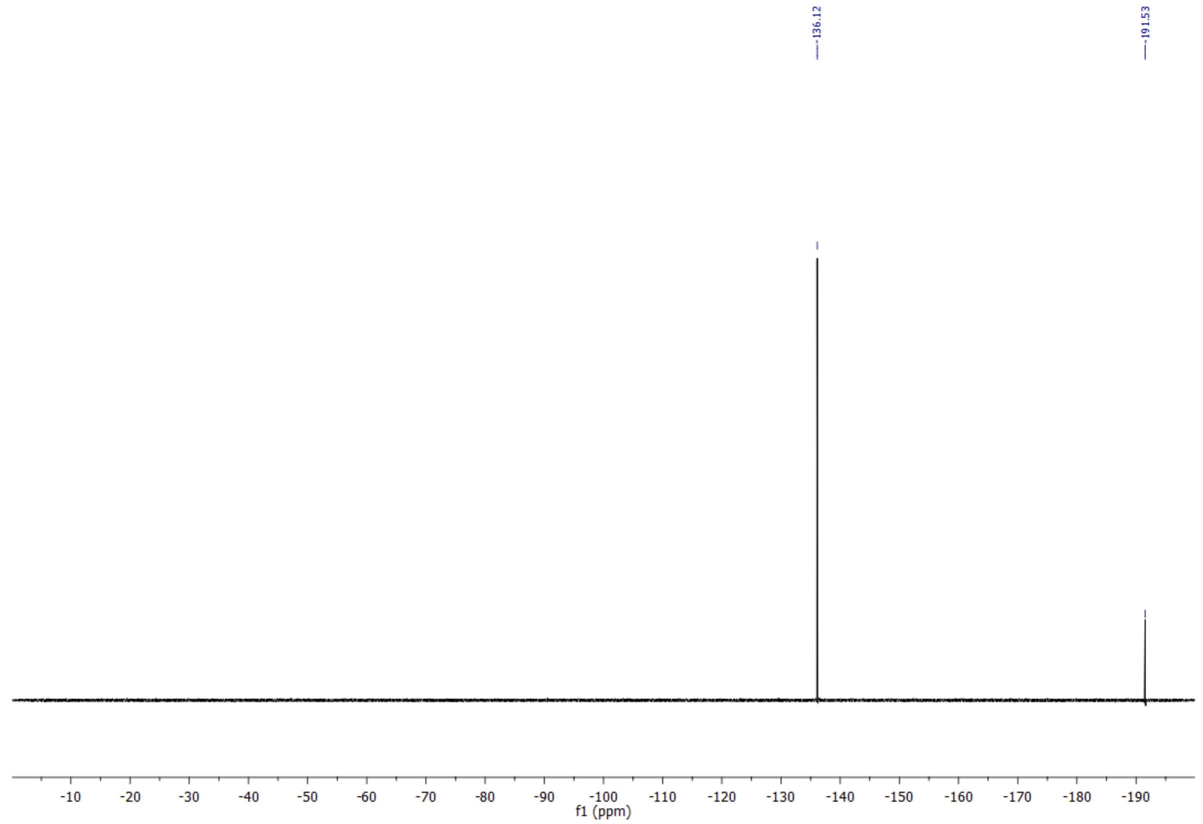

^{1}H NMR of 7i

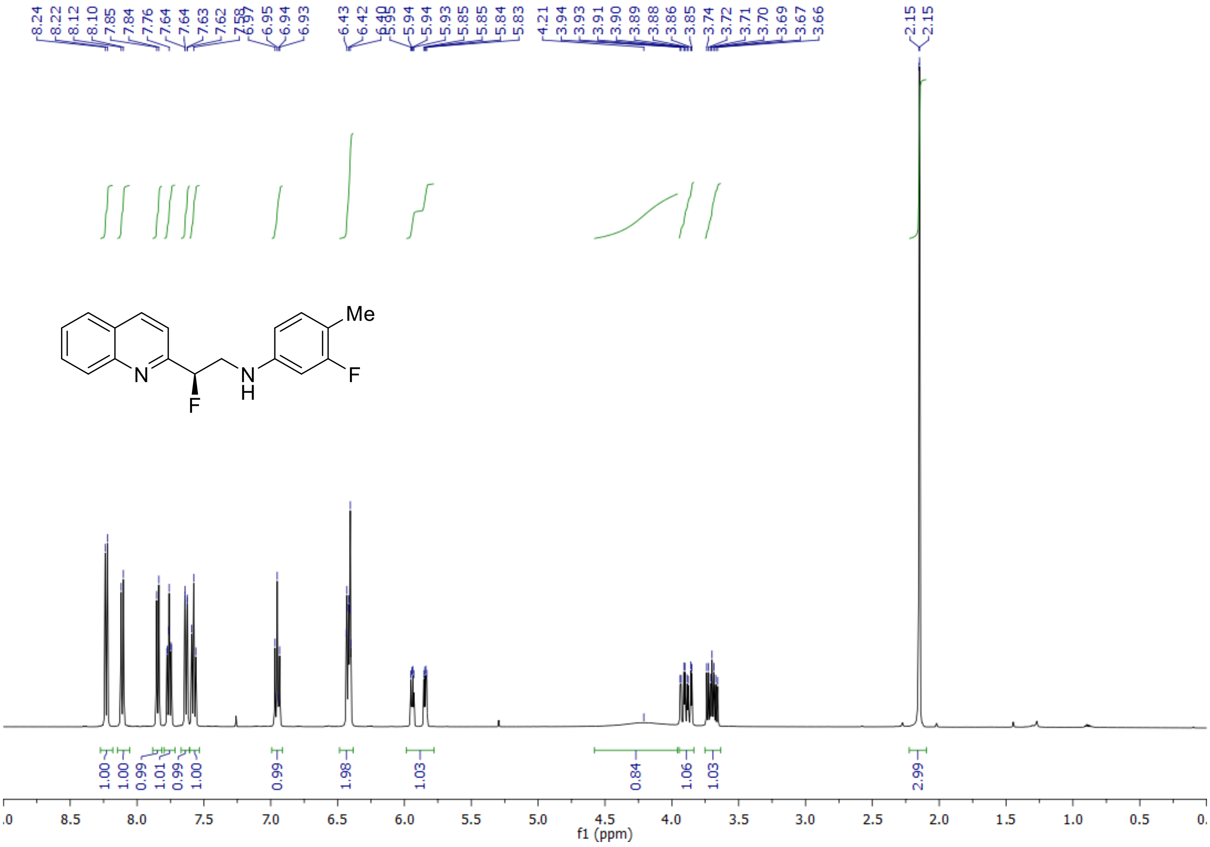

^{13}C NMR of 7i

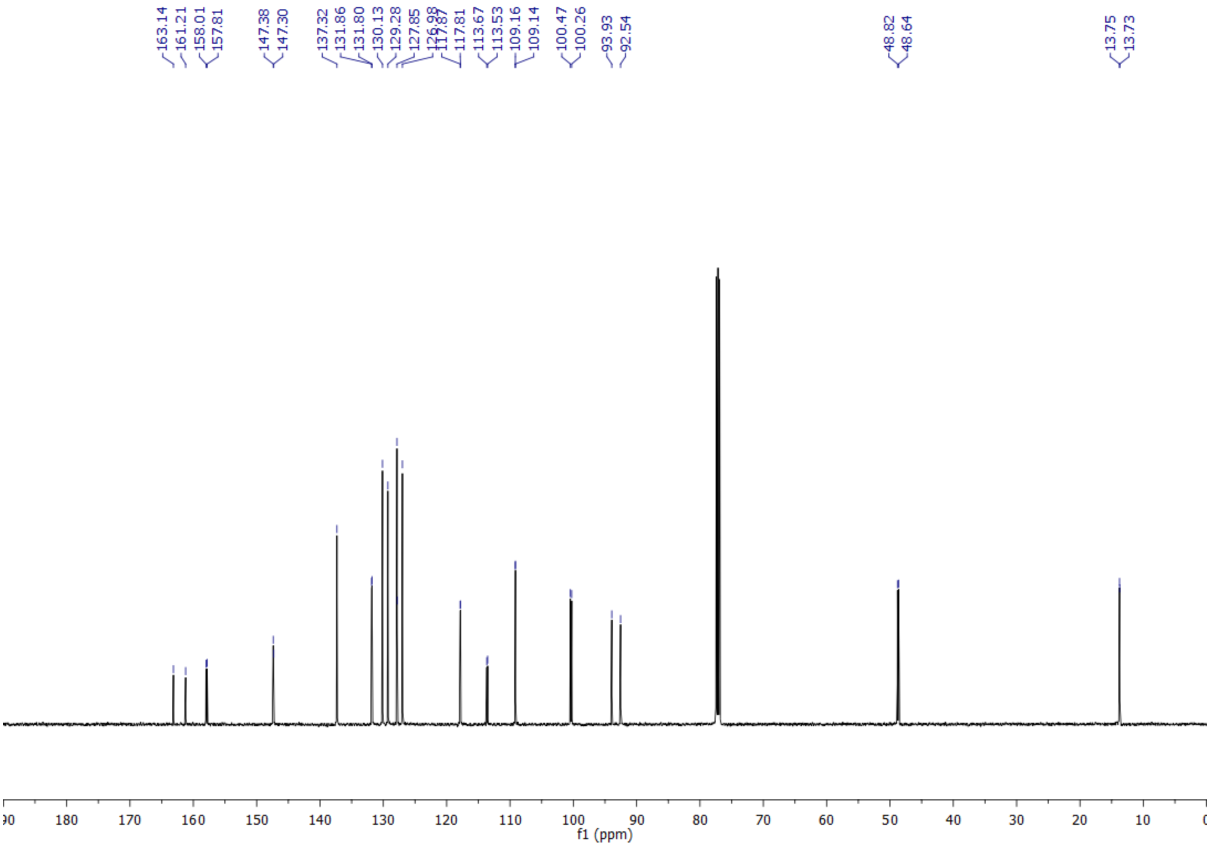

^{19}F NMR of 7i

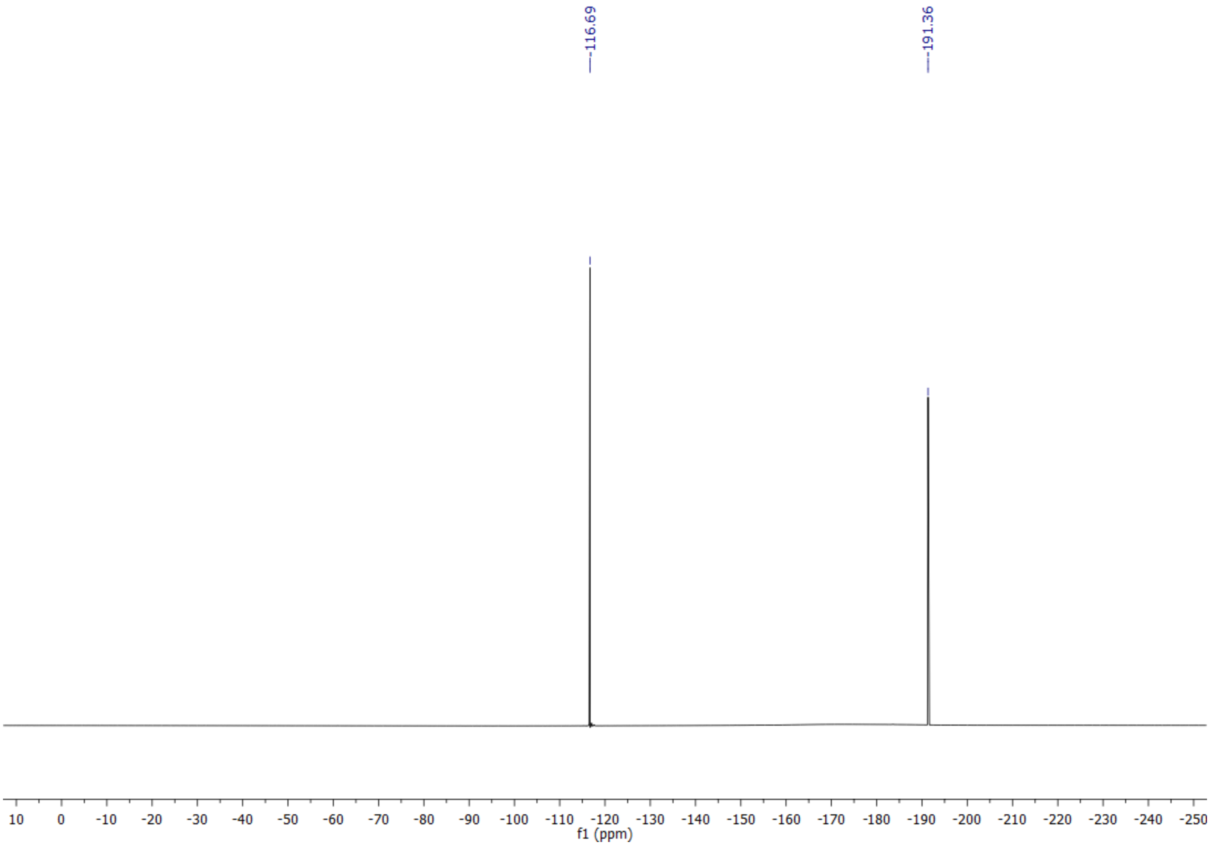

^{1}H NMR of 7j

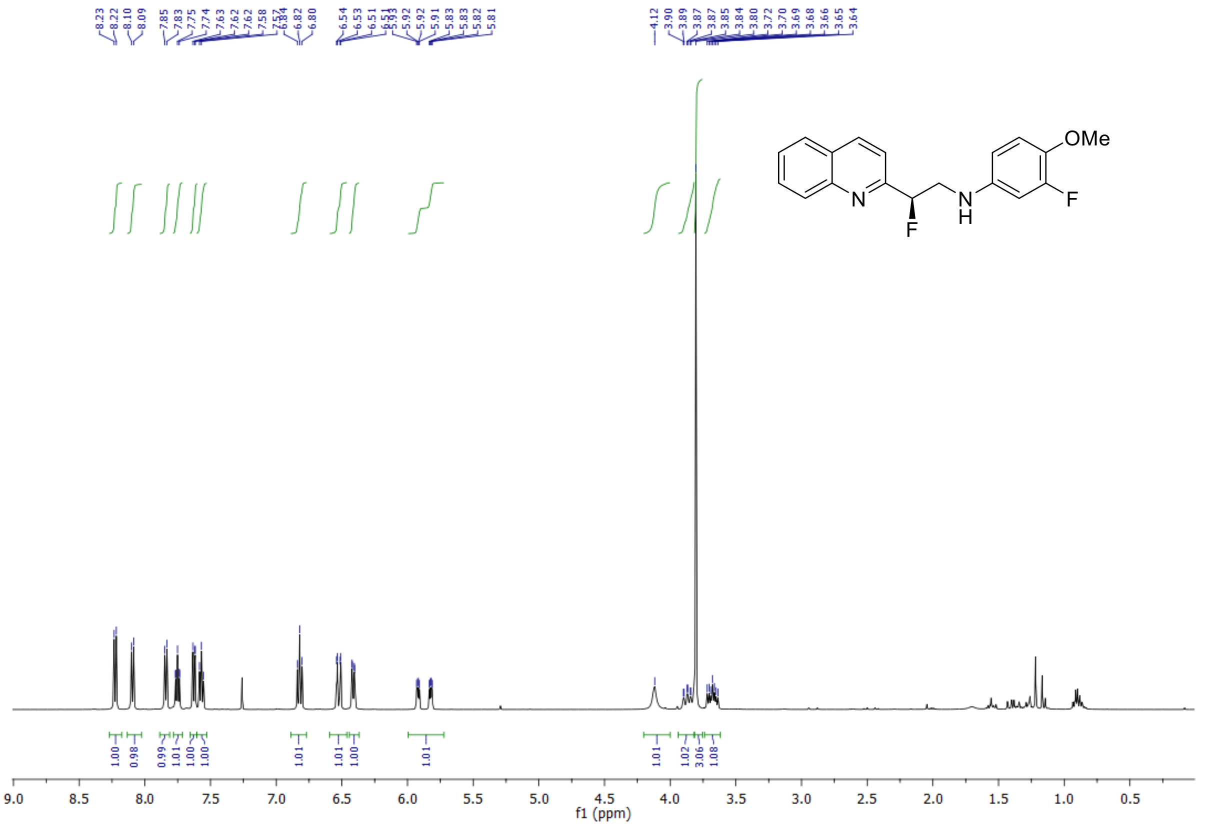

^{13}C NMR of 7j

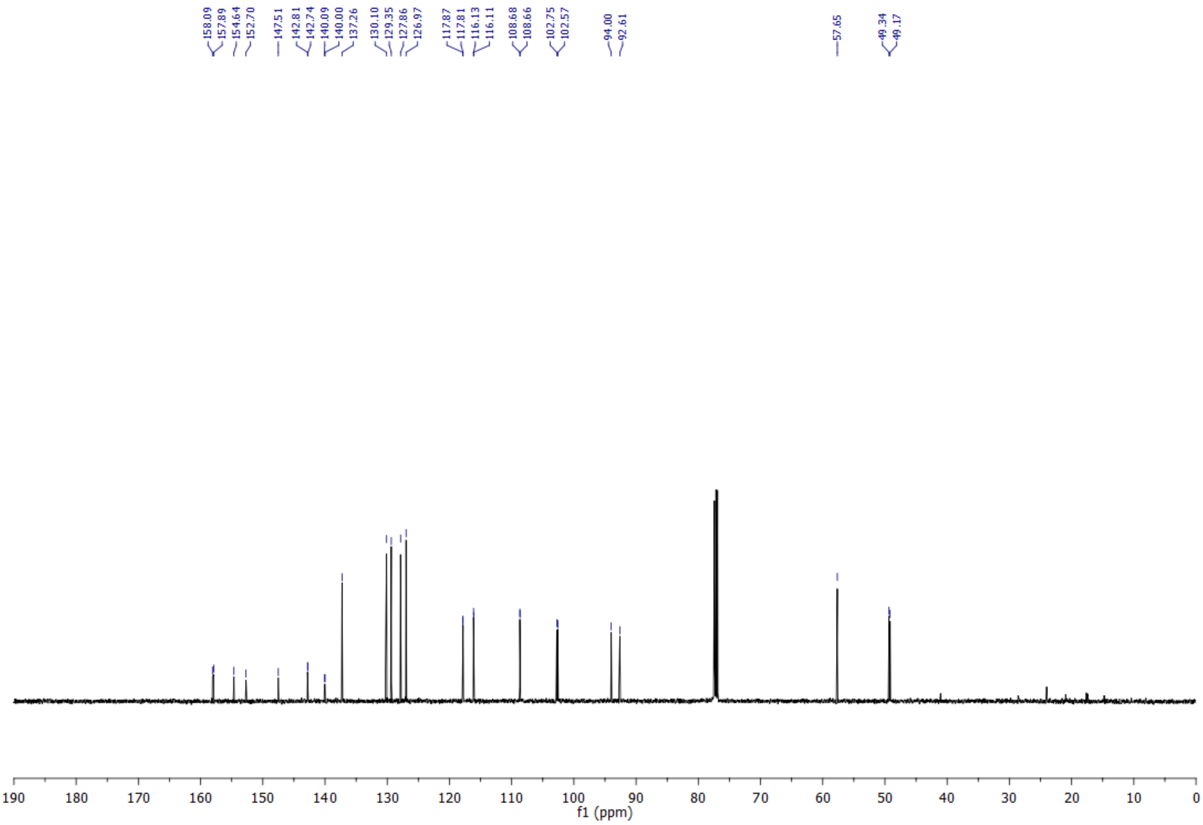

^{19}F NMR of 7j

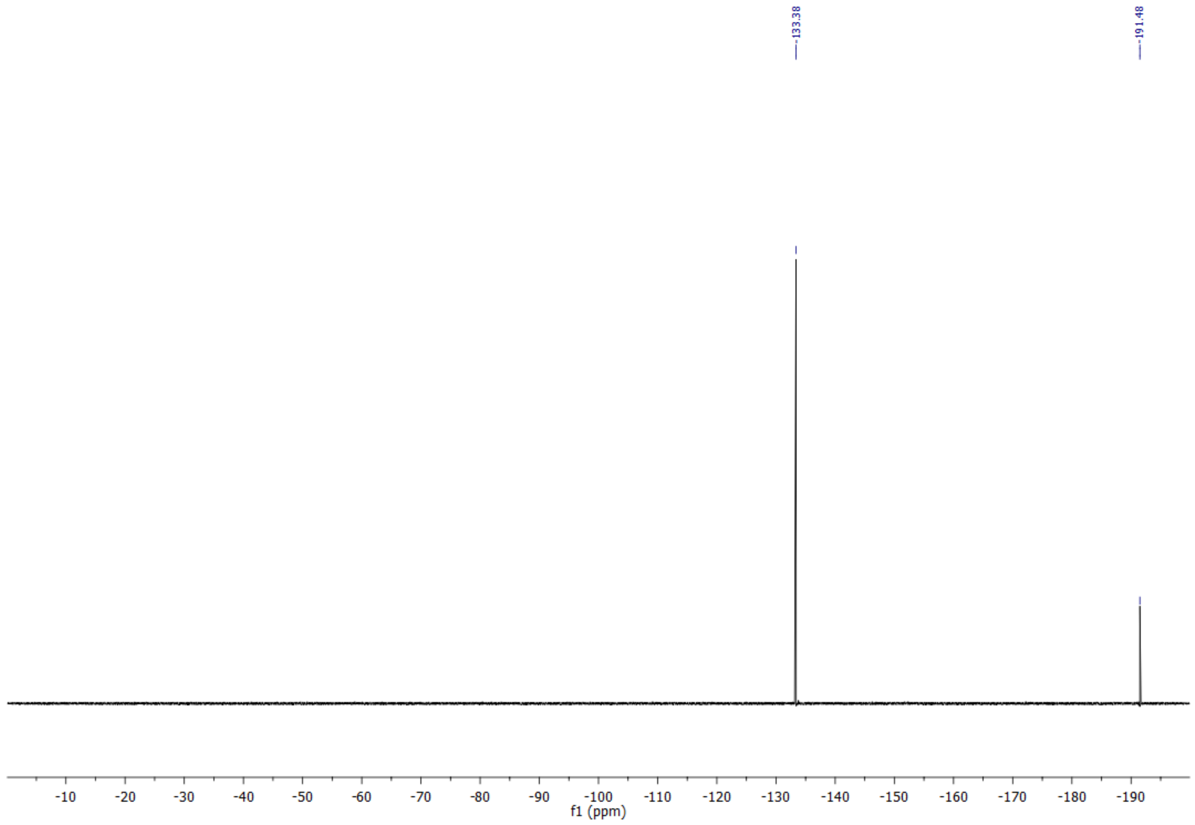

^{1}H NMR of 7k

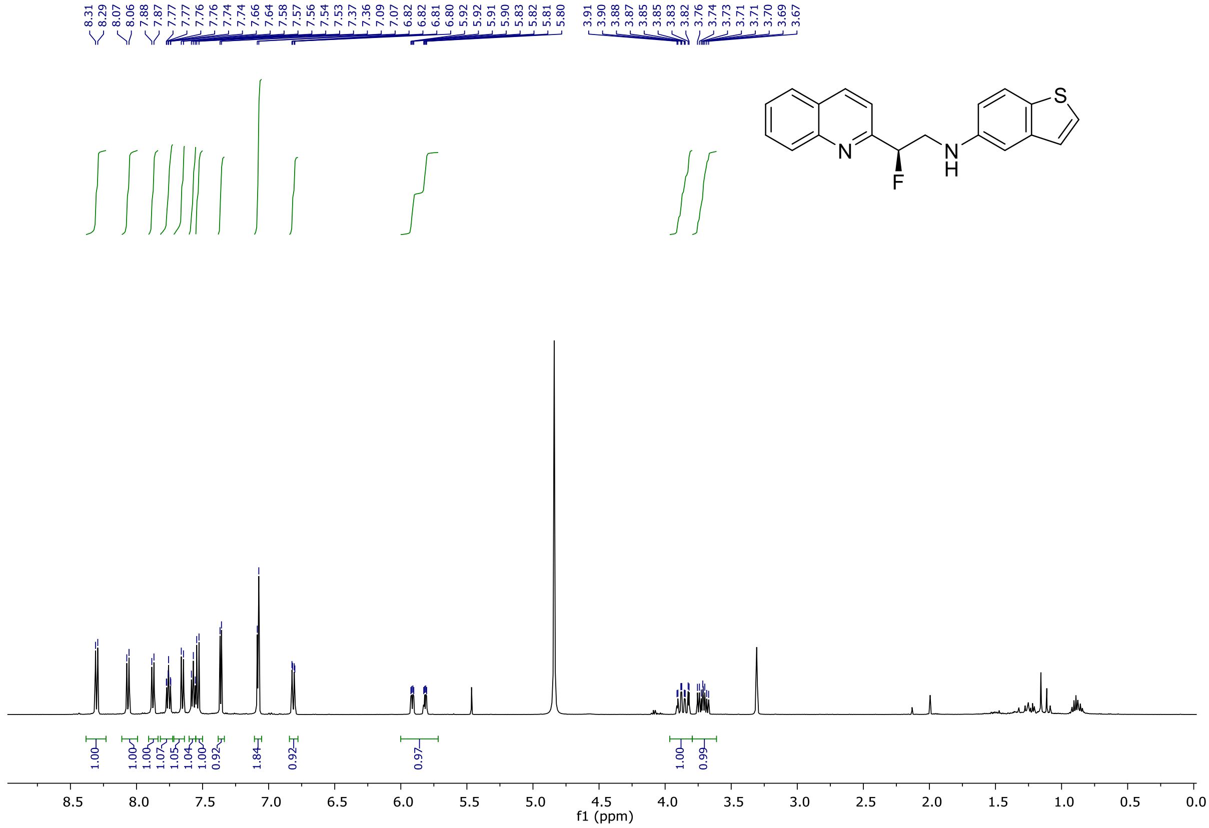

^{13}C NMR of 7k

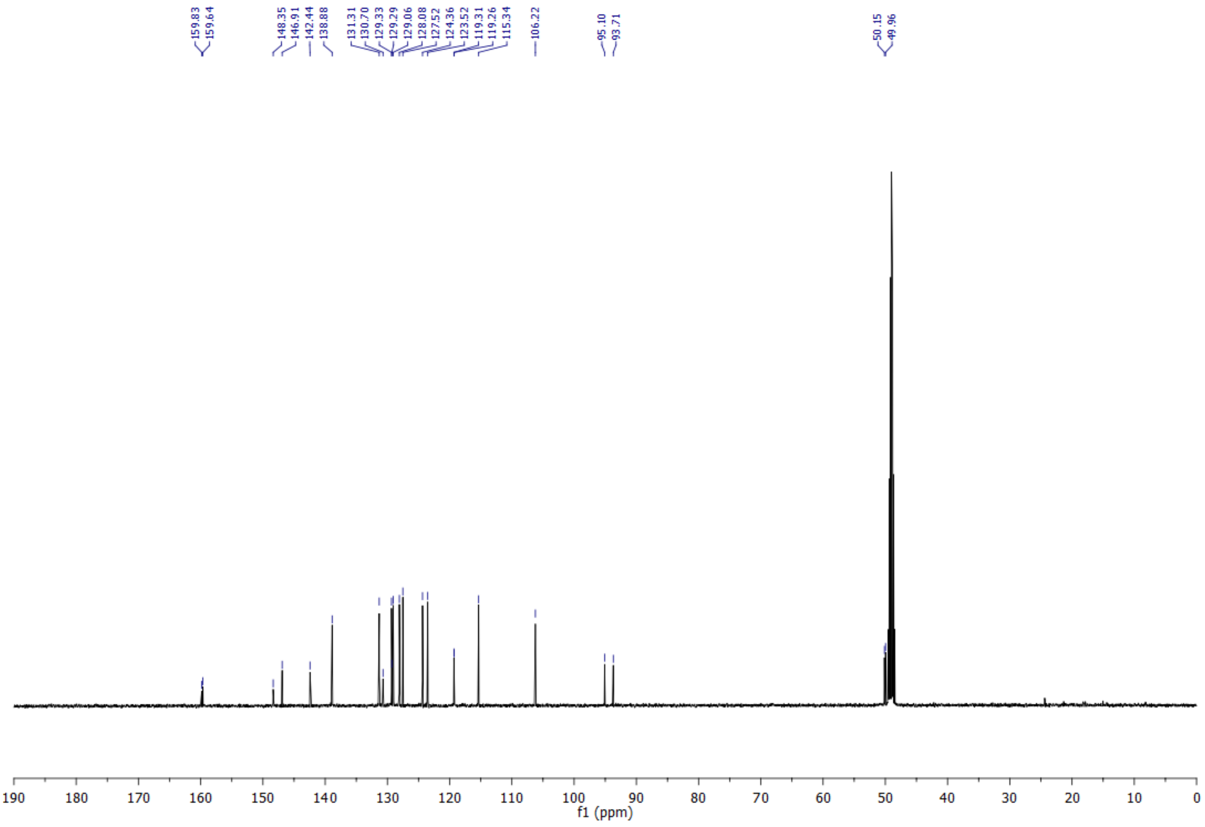

^{19}F NMR of 7k

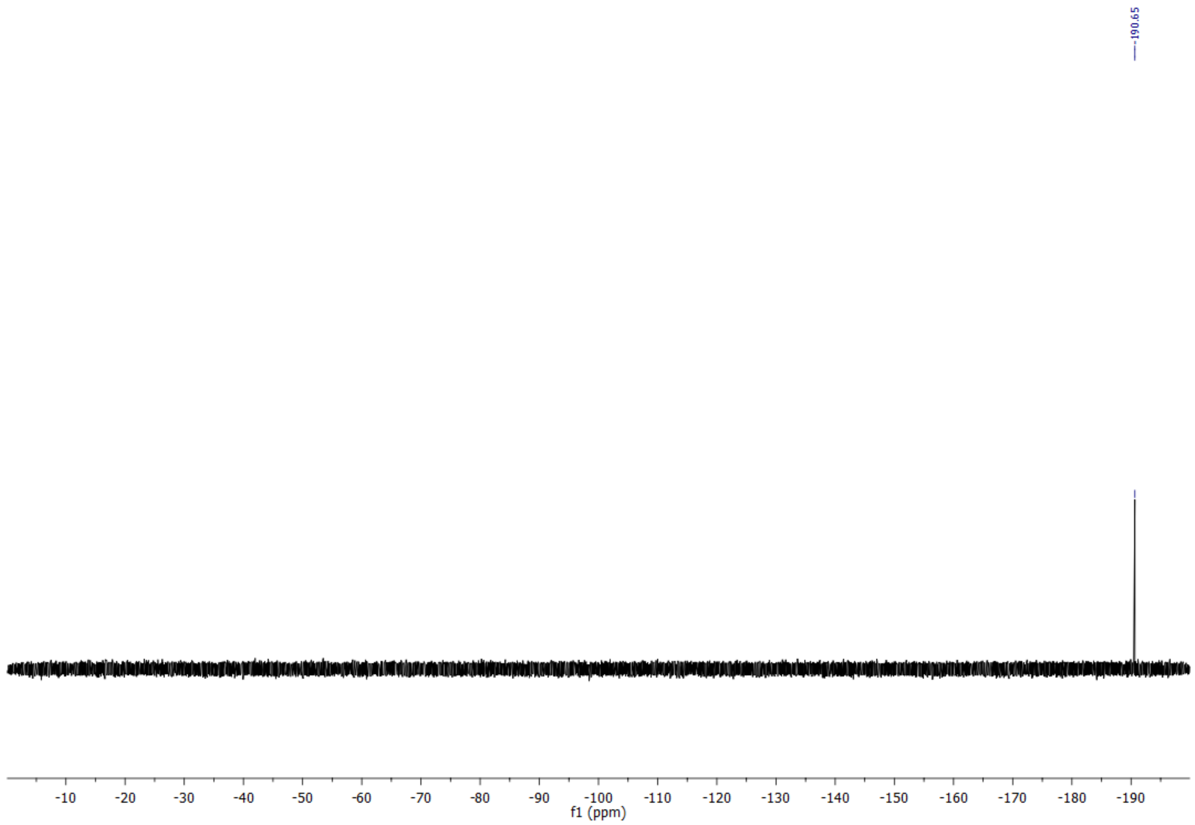

^{1}H NMR of 7l

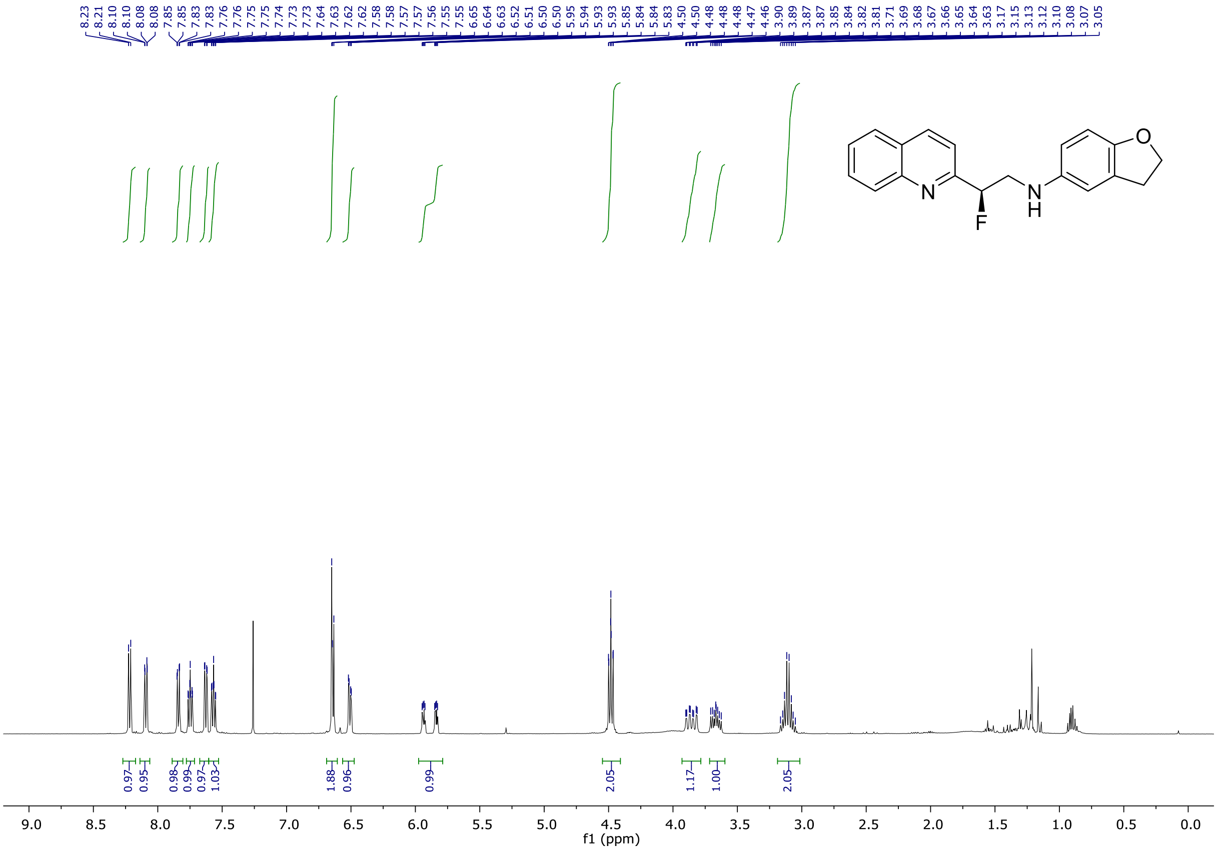

^{13}C NMR of 7l

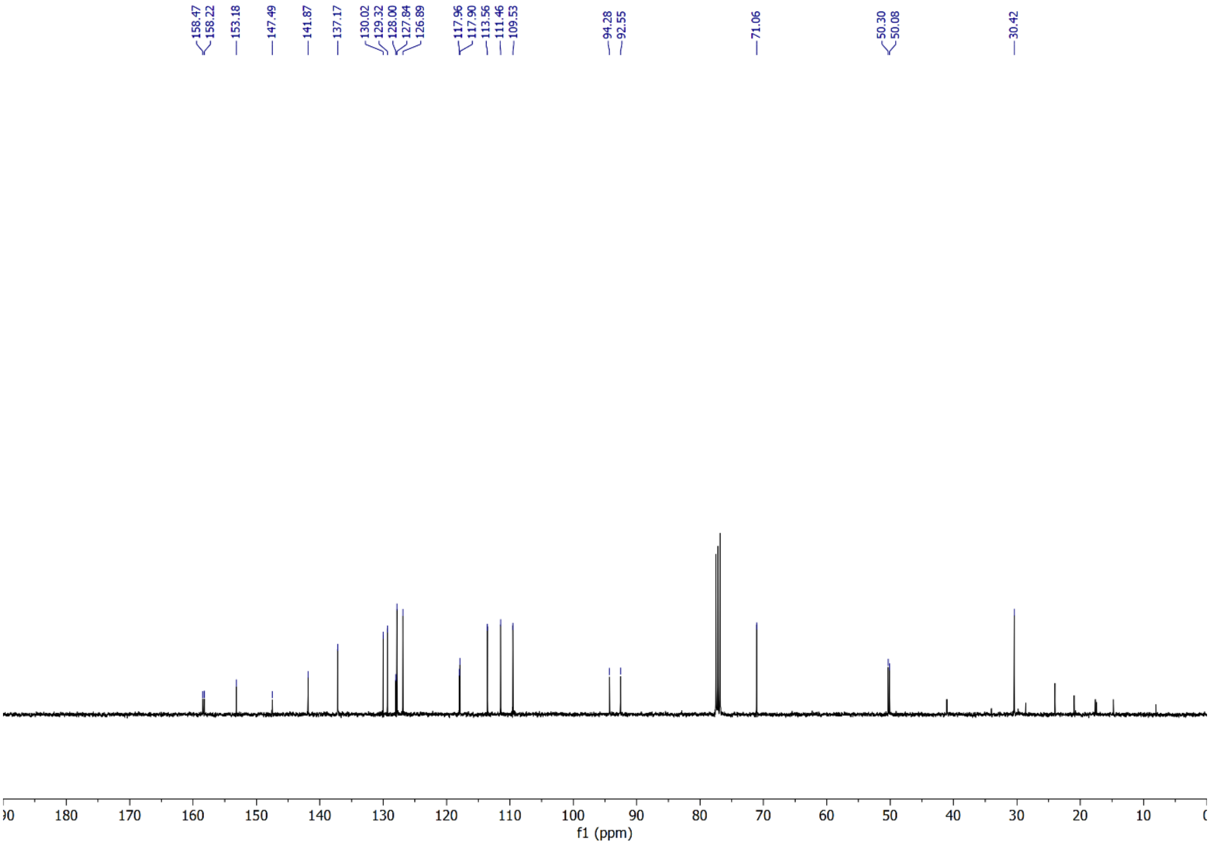

^{19}F NMR of 7l

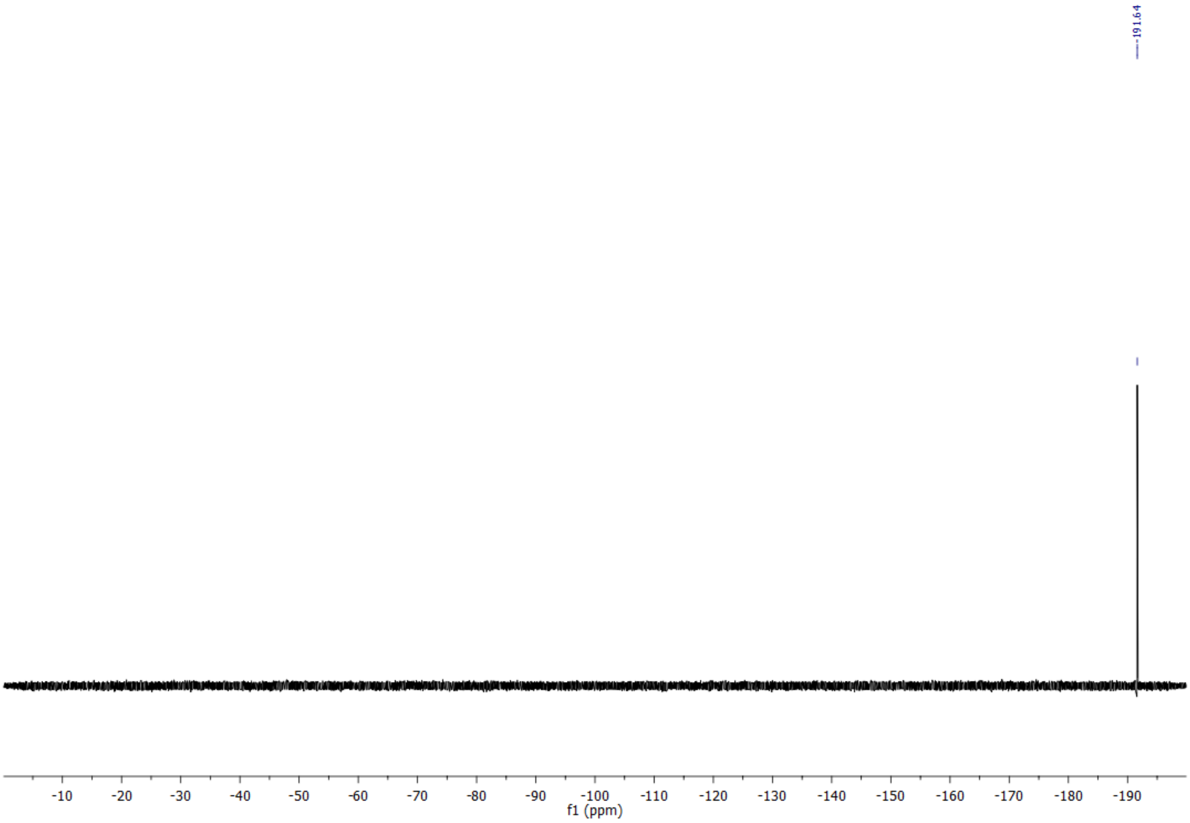

^{1}H NMR of 7m

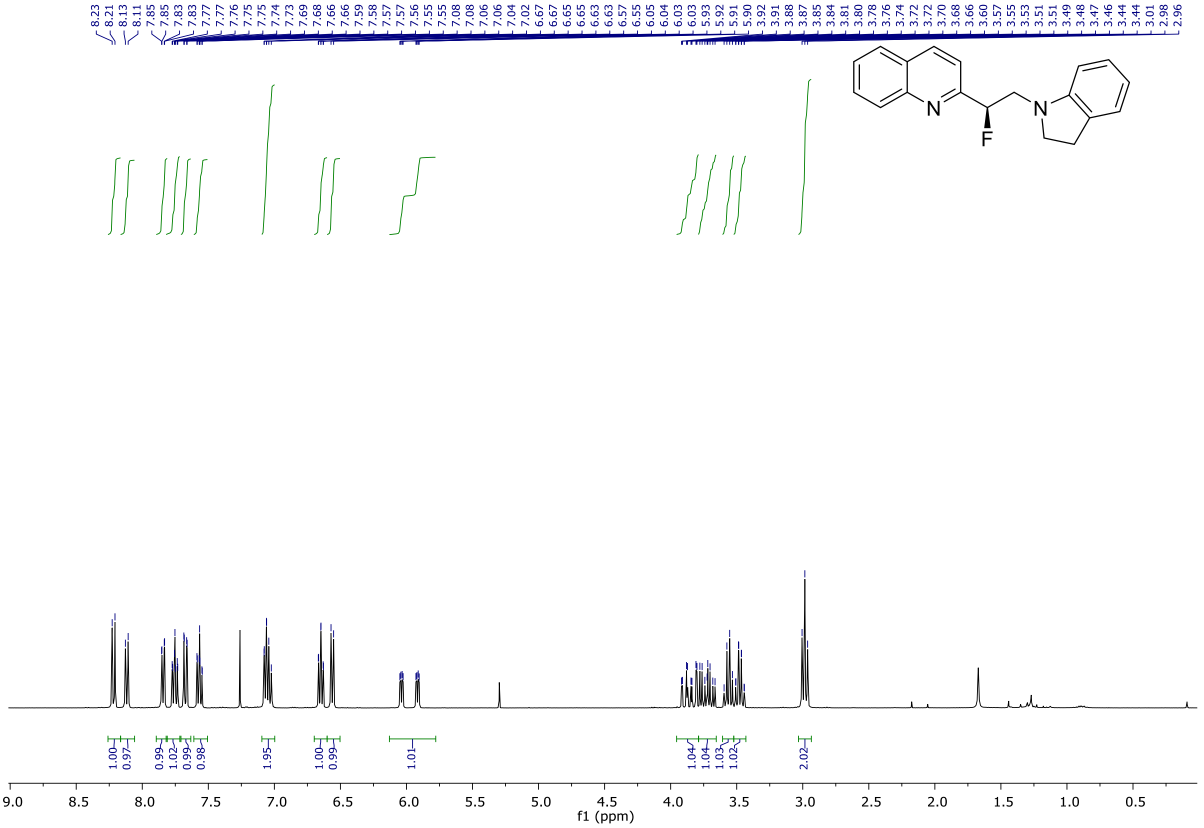

^{13}C NMR of 7m

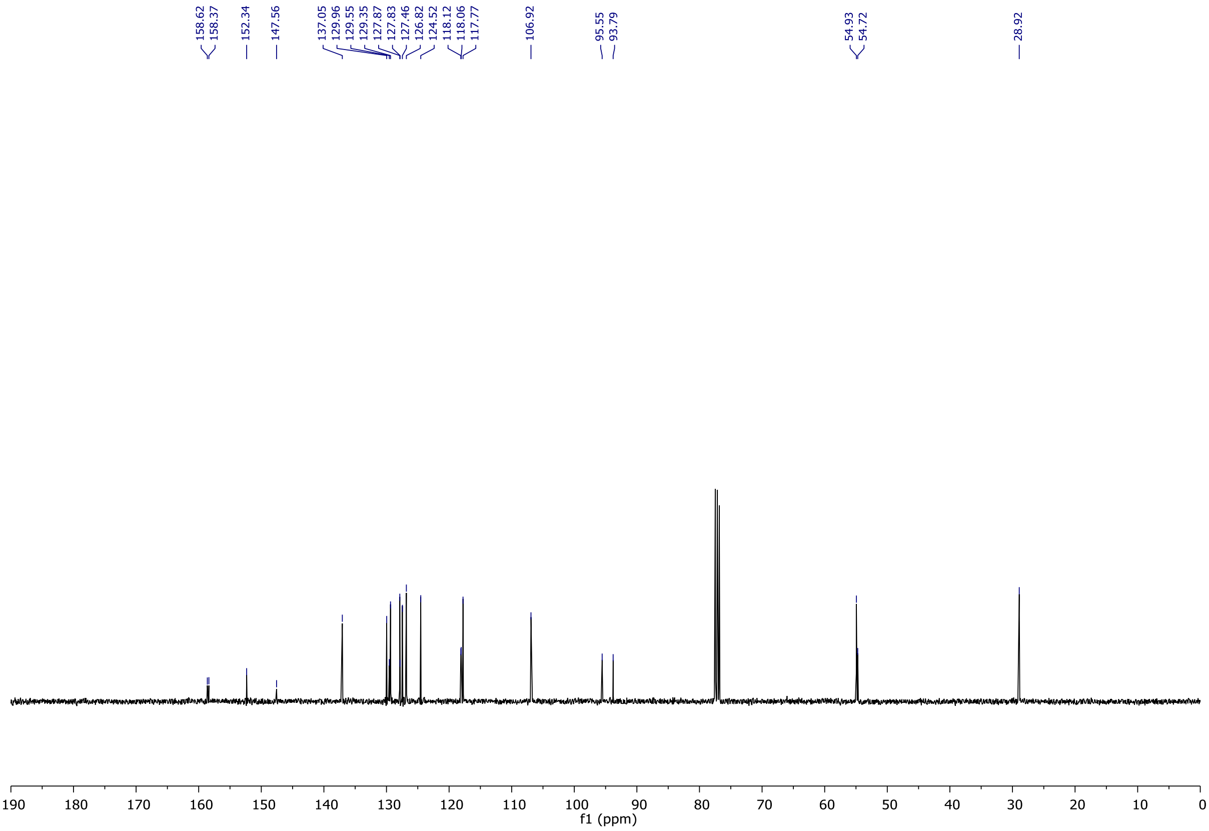

^{19}F NMR of 7m

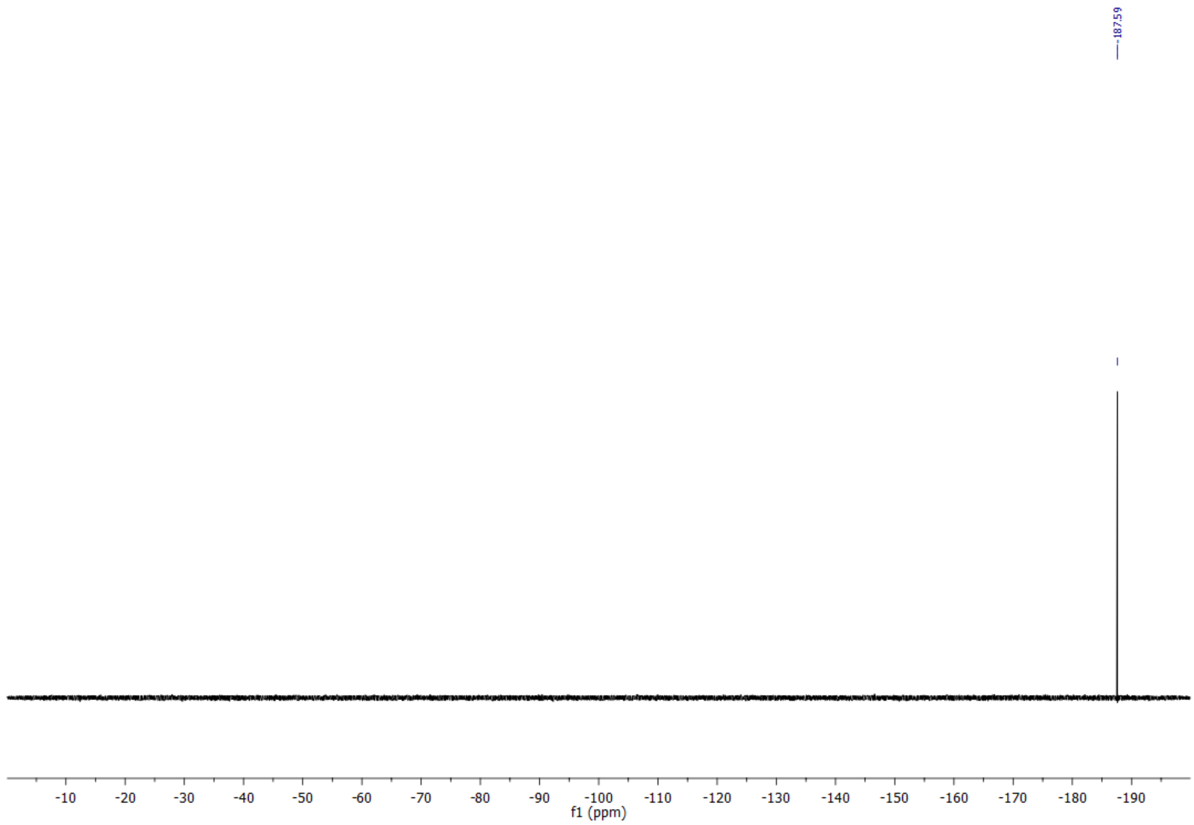

^{1}H NMR of 7n

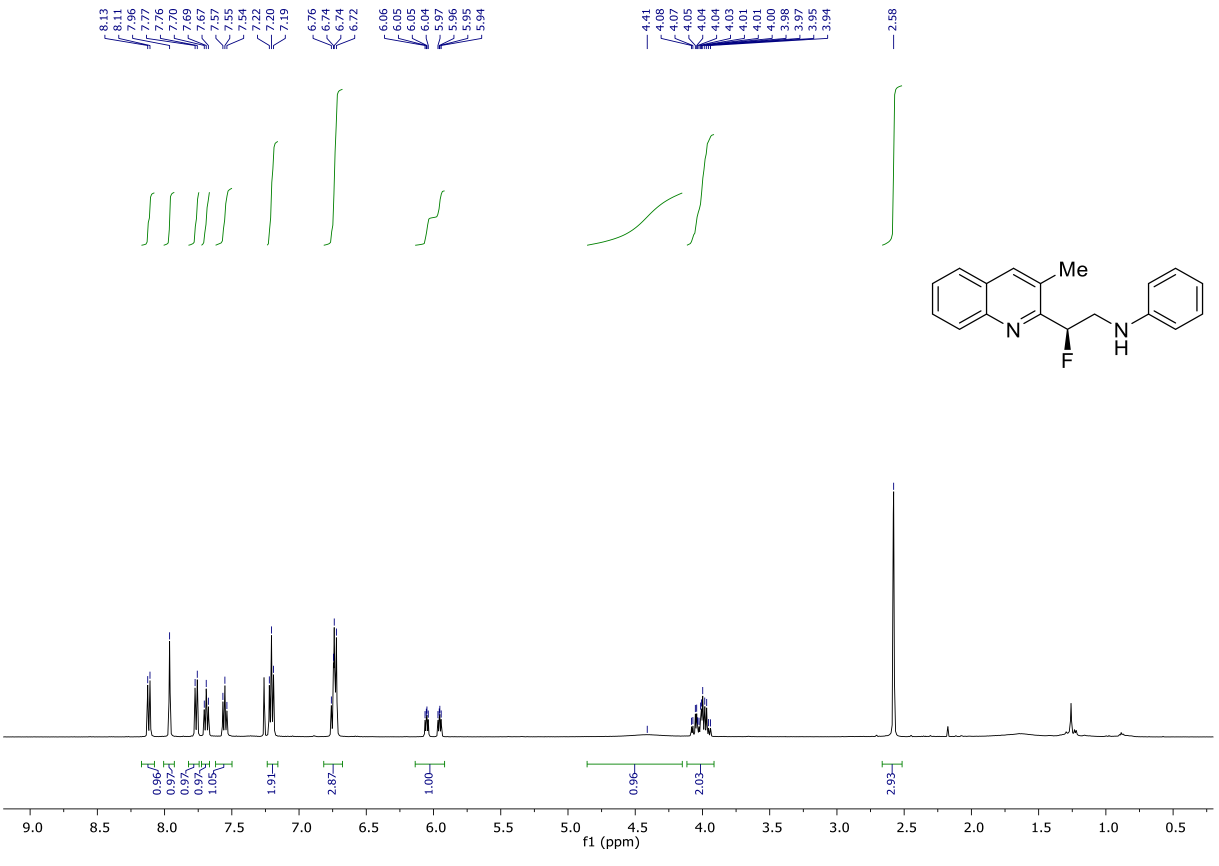

^{13}C NMR of 7n

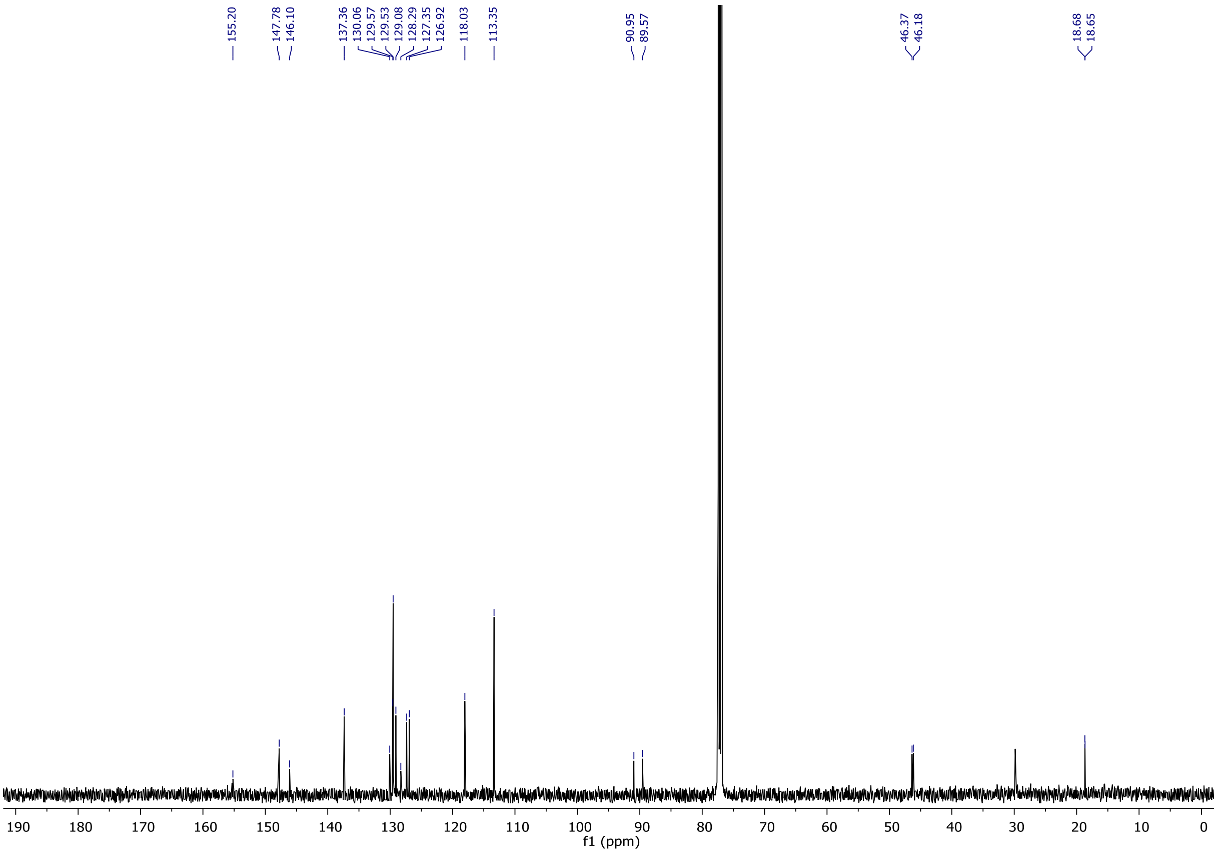

^{19}F NMR of 7n

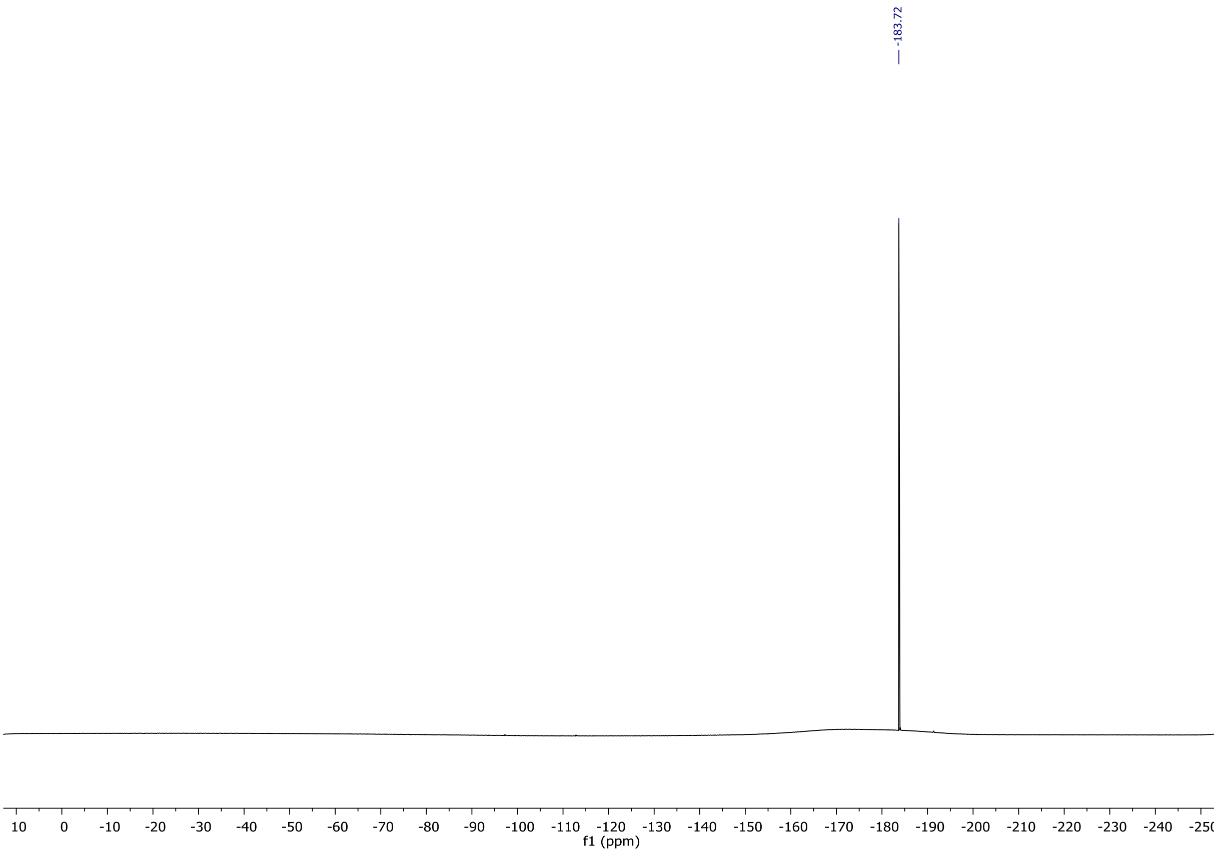

^{1}H NMR of 7o

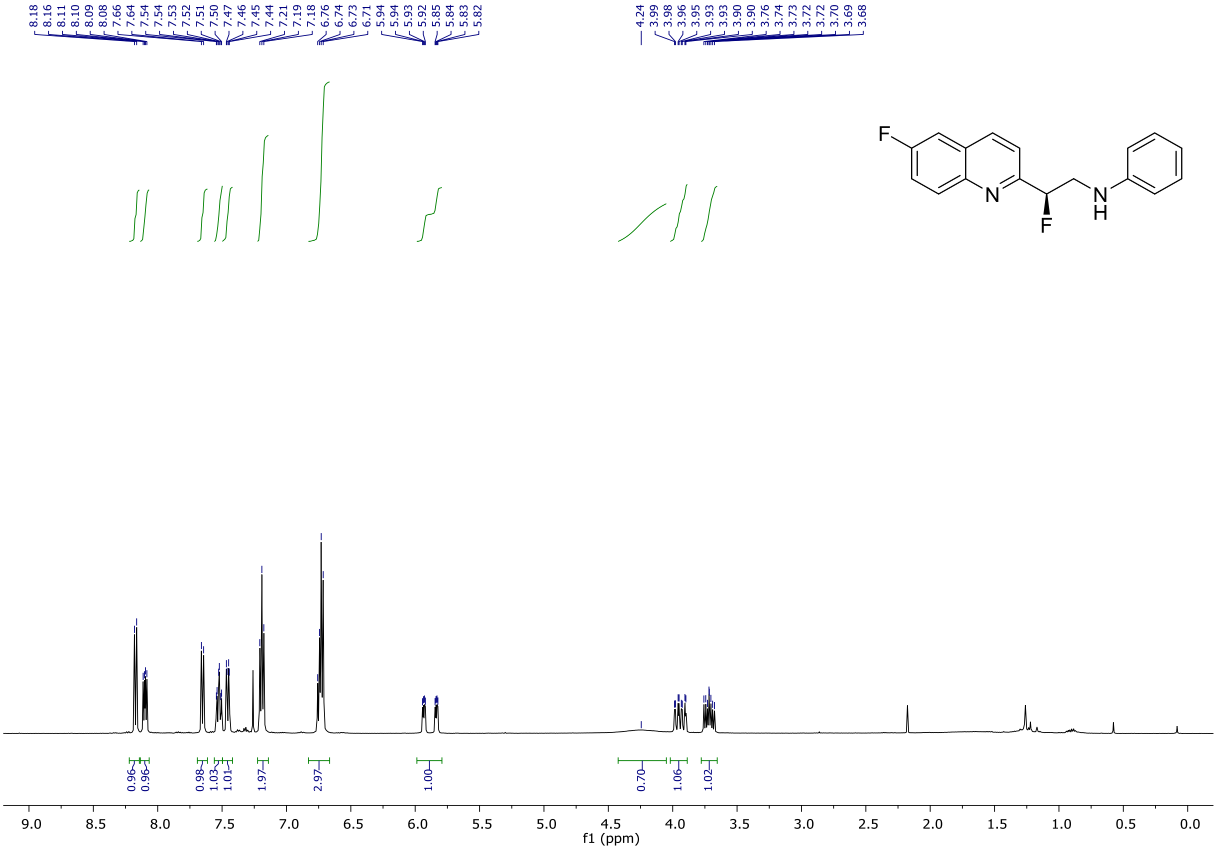

^{13}C NMR of 7o

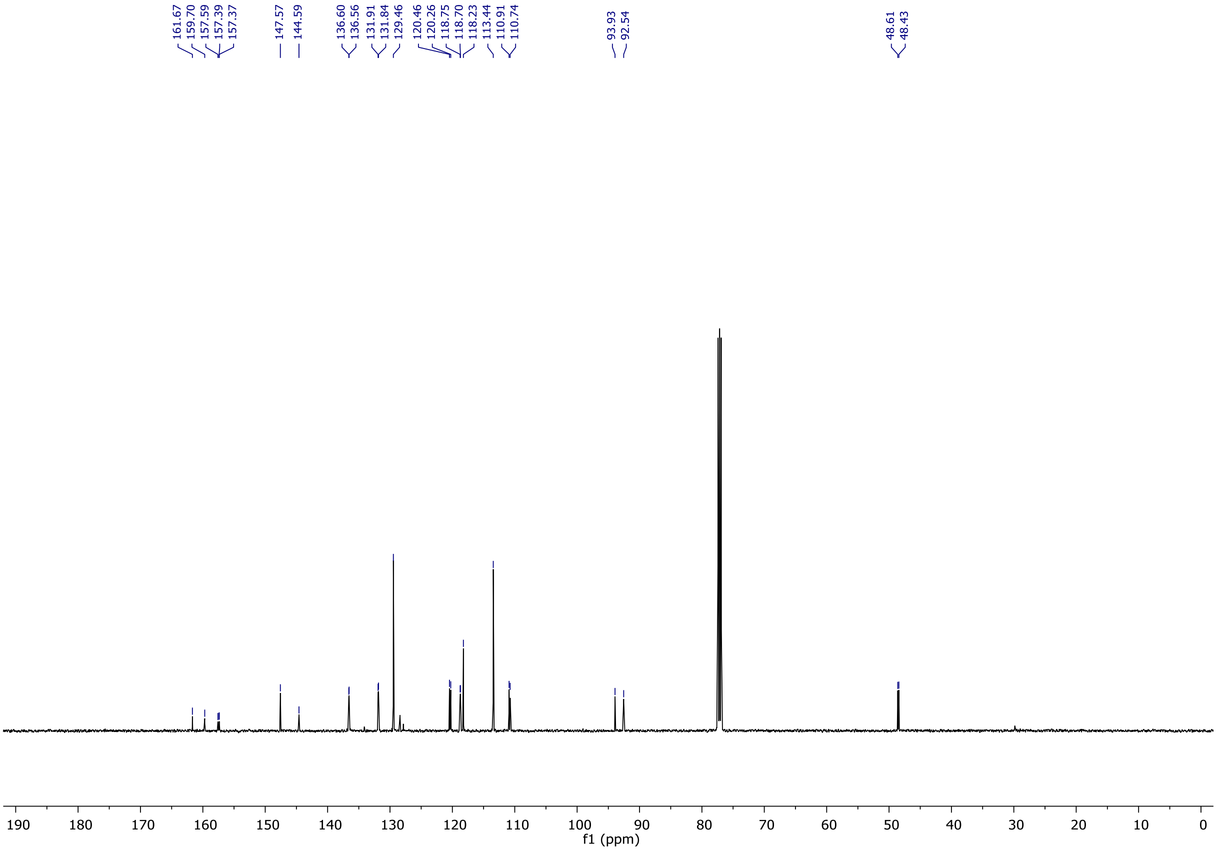

^{19}F NMR of 7o

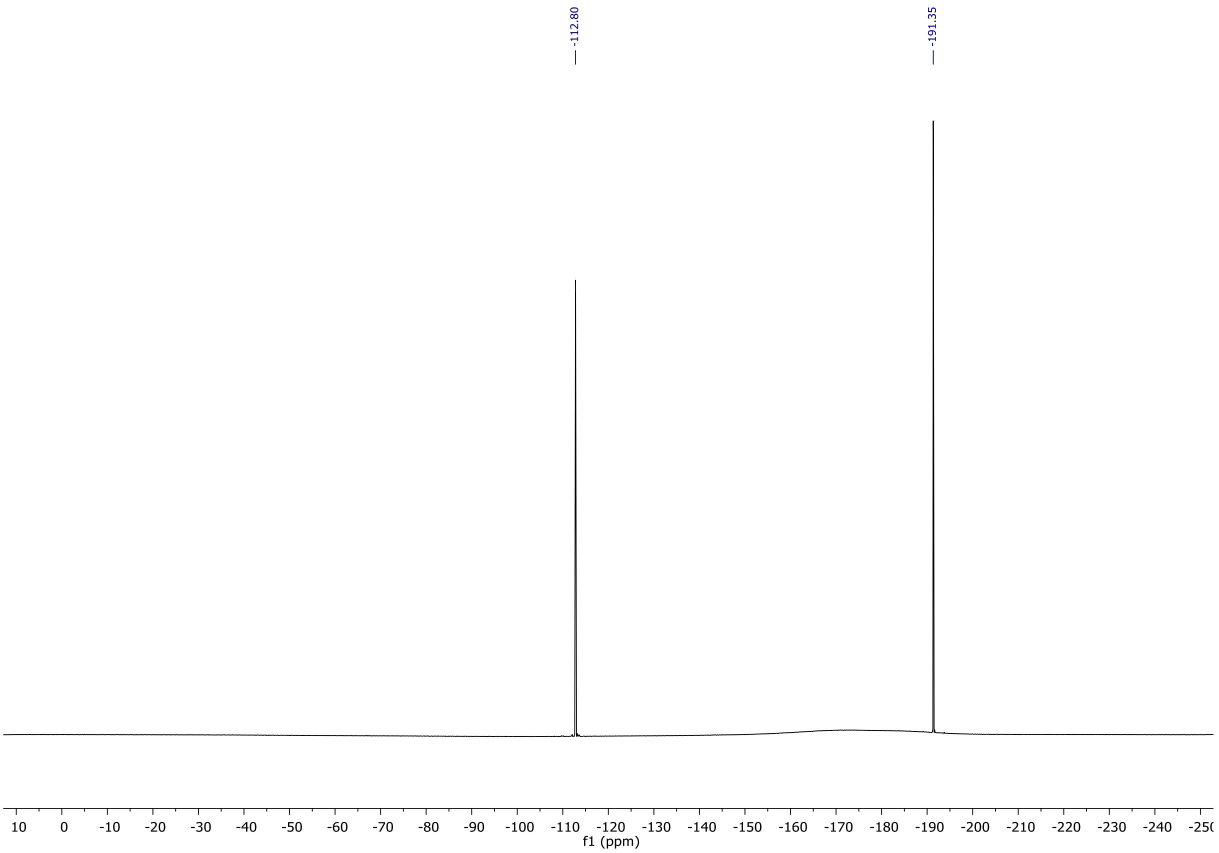

^{1}H NMR of 7p

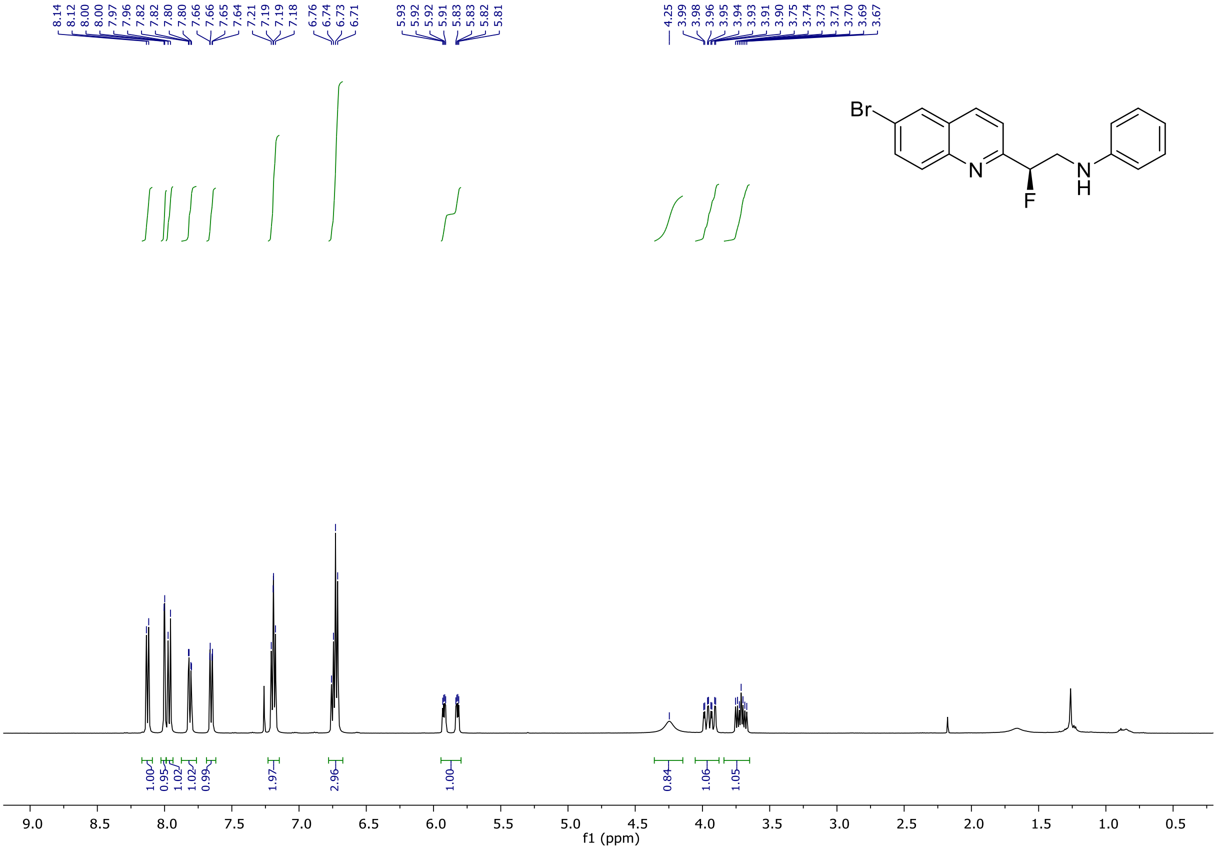

^{13}C NMR of 7p

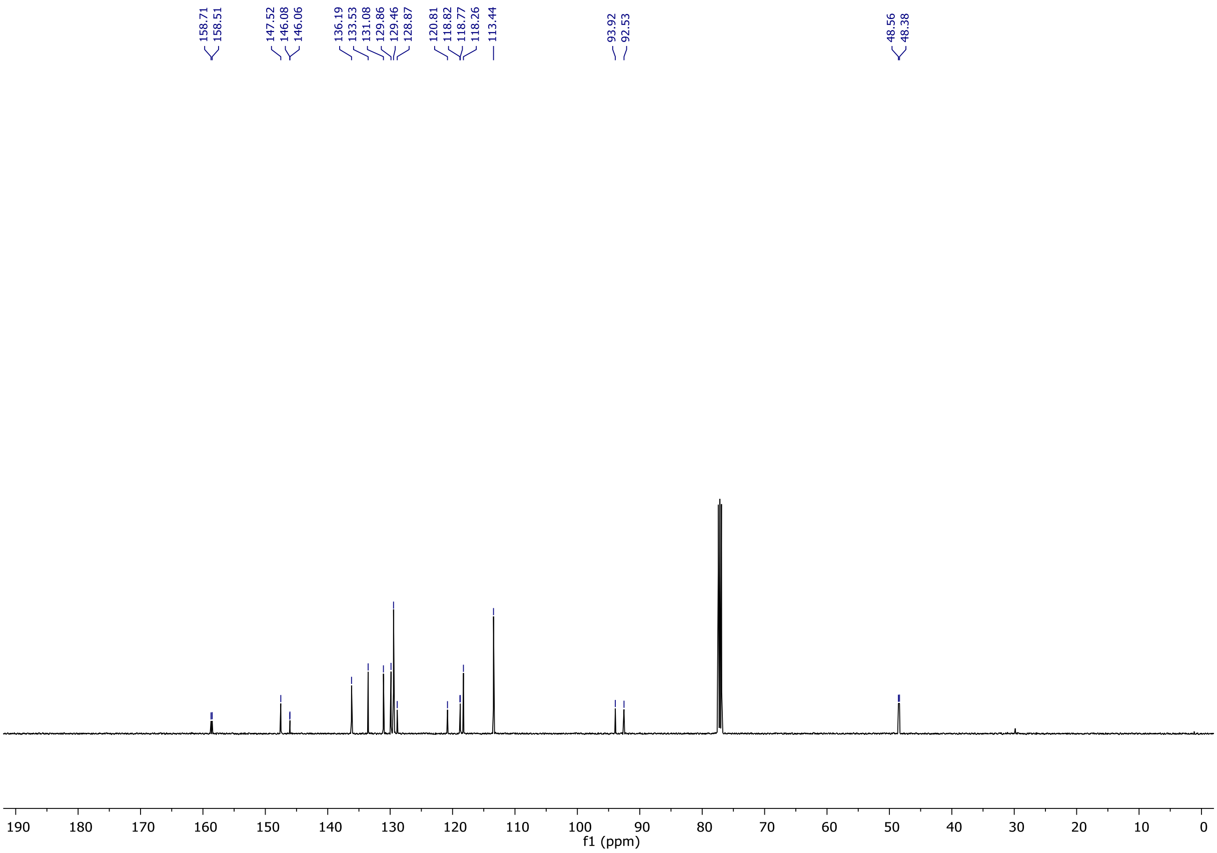

^{19}F NMR of 7p

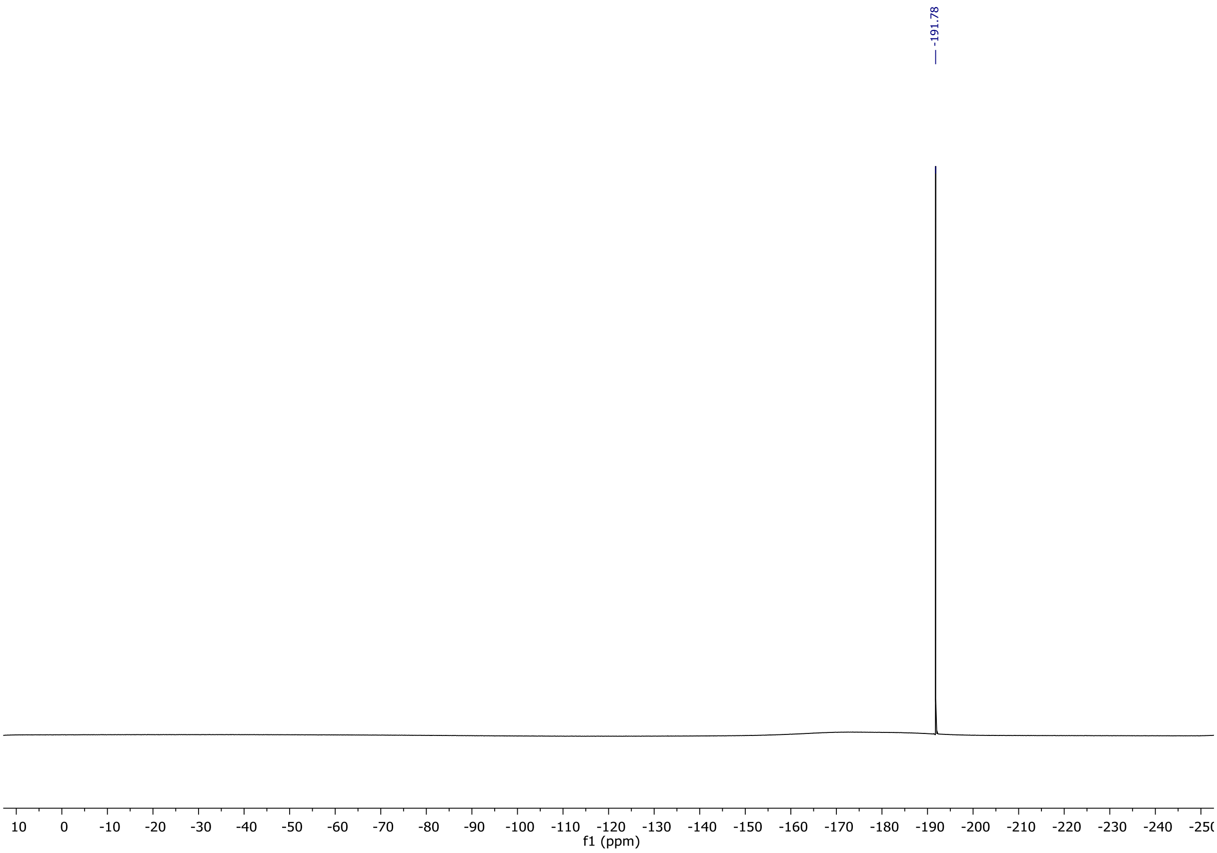

^{1}H NMR of 7q

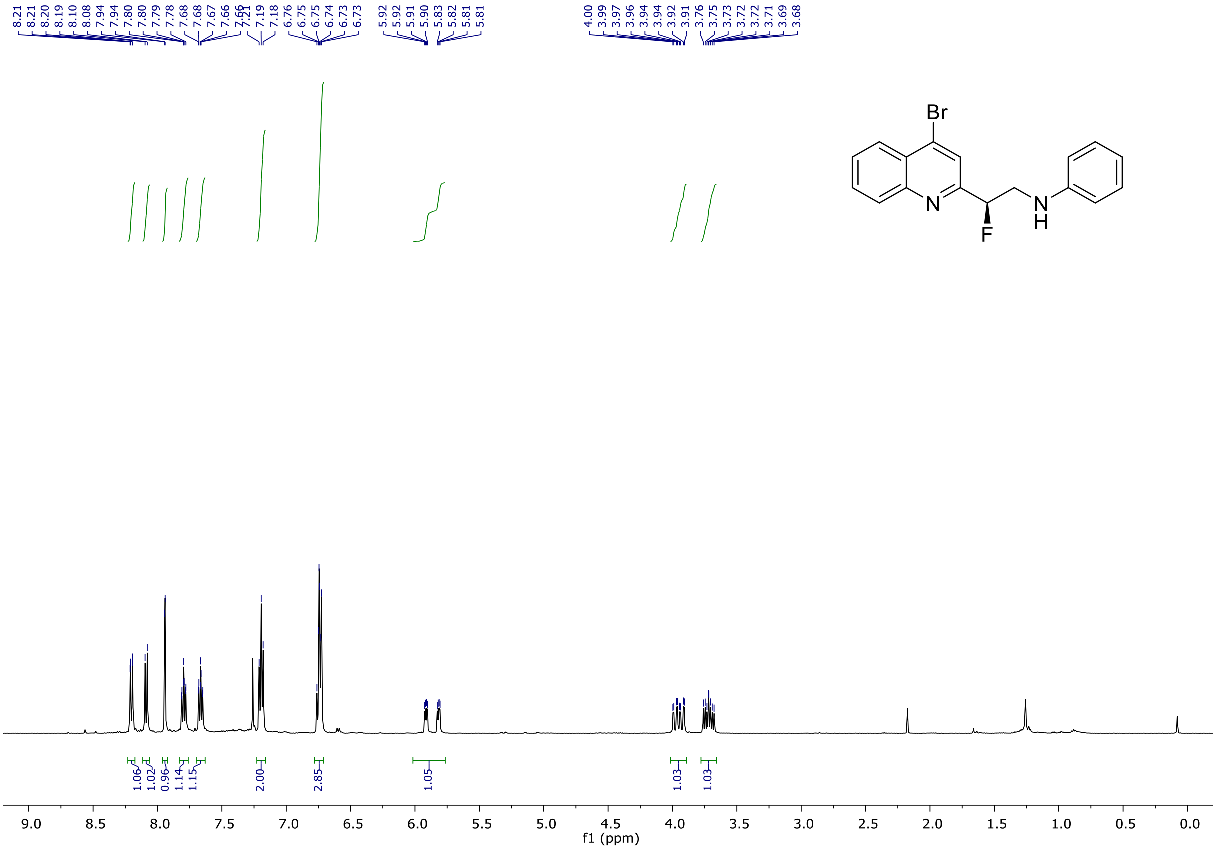

^{13}C NMR of 7q

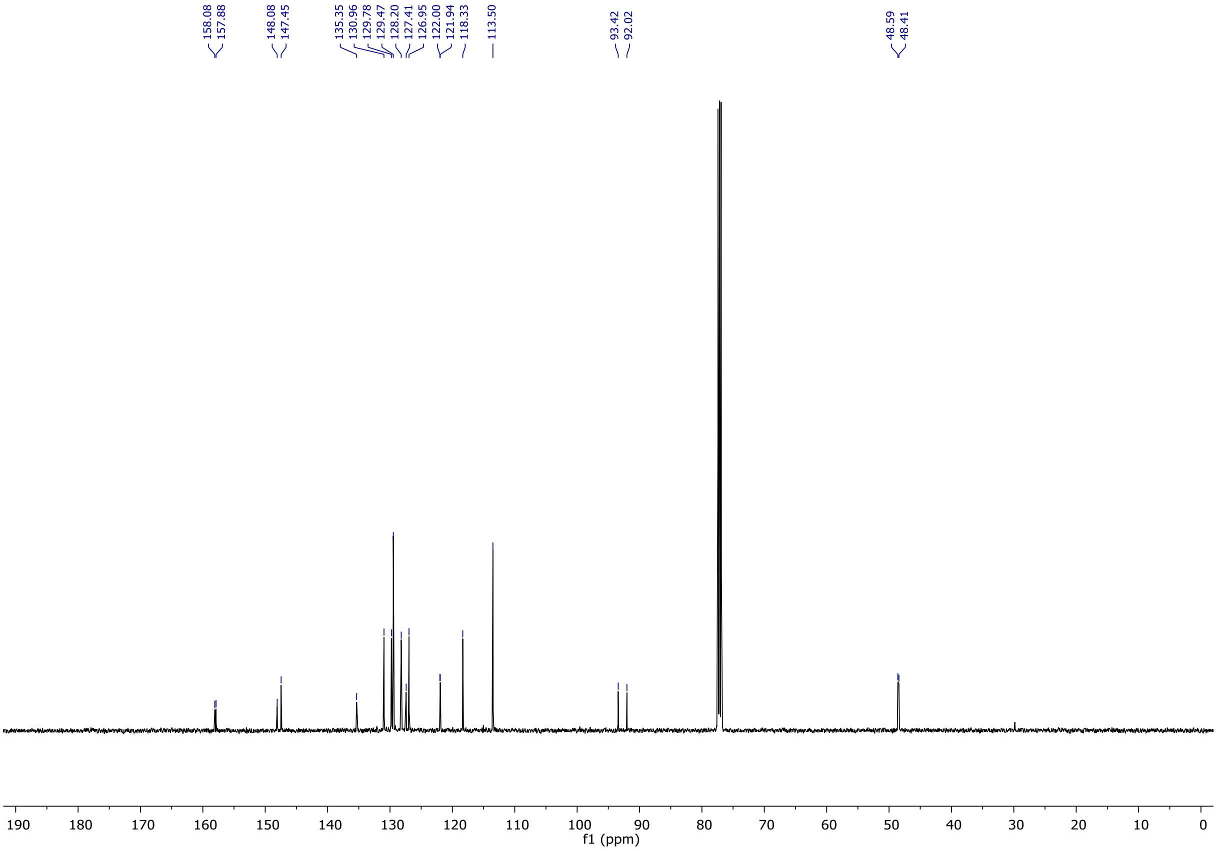

^{19}F NMR of 7q

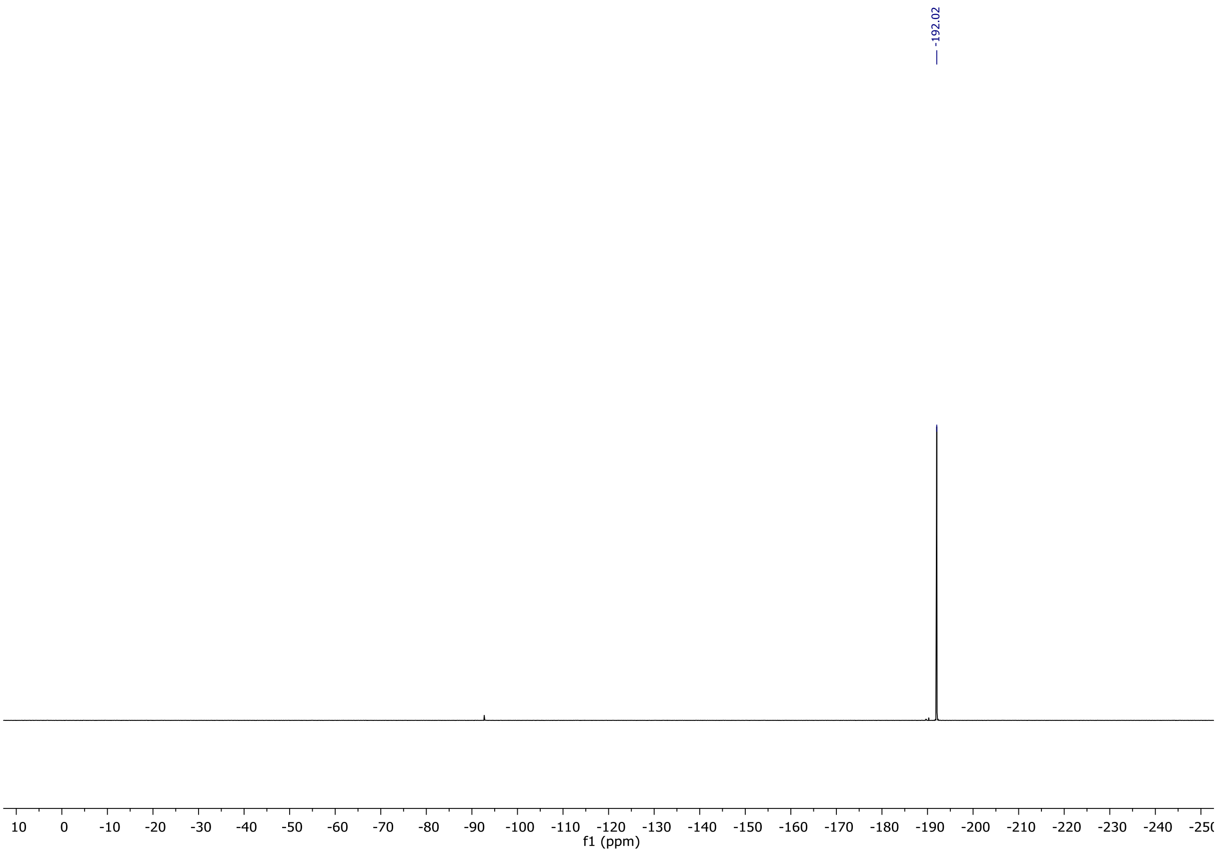

^{1}H NMR of 7r

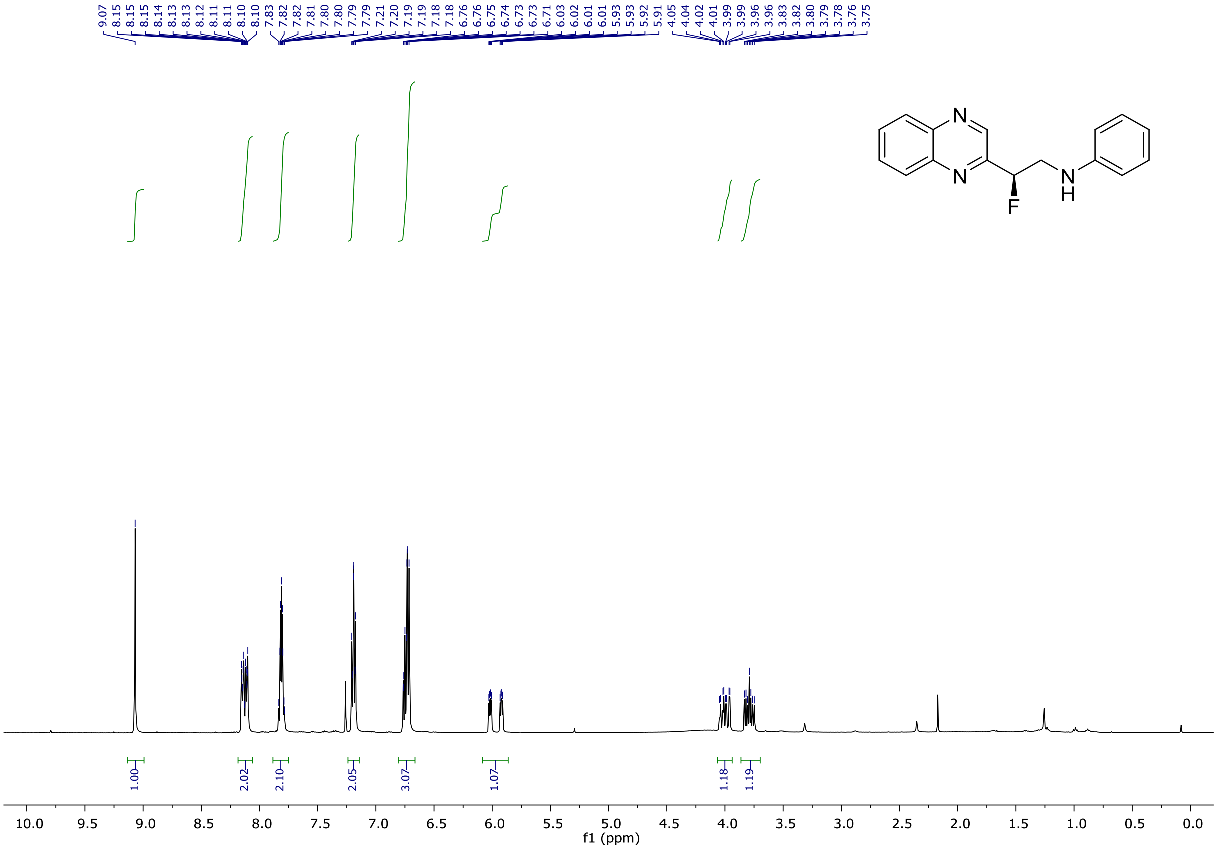

^{13}C NMR of 7r

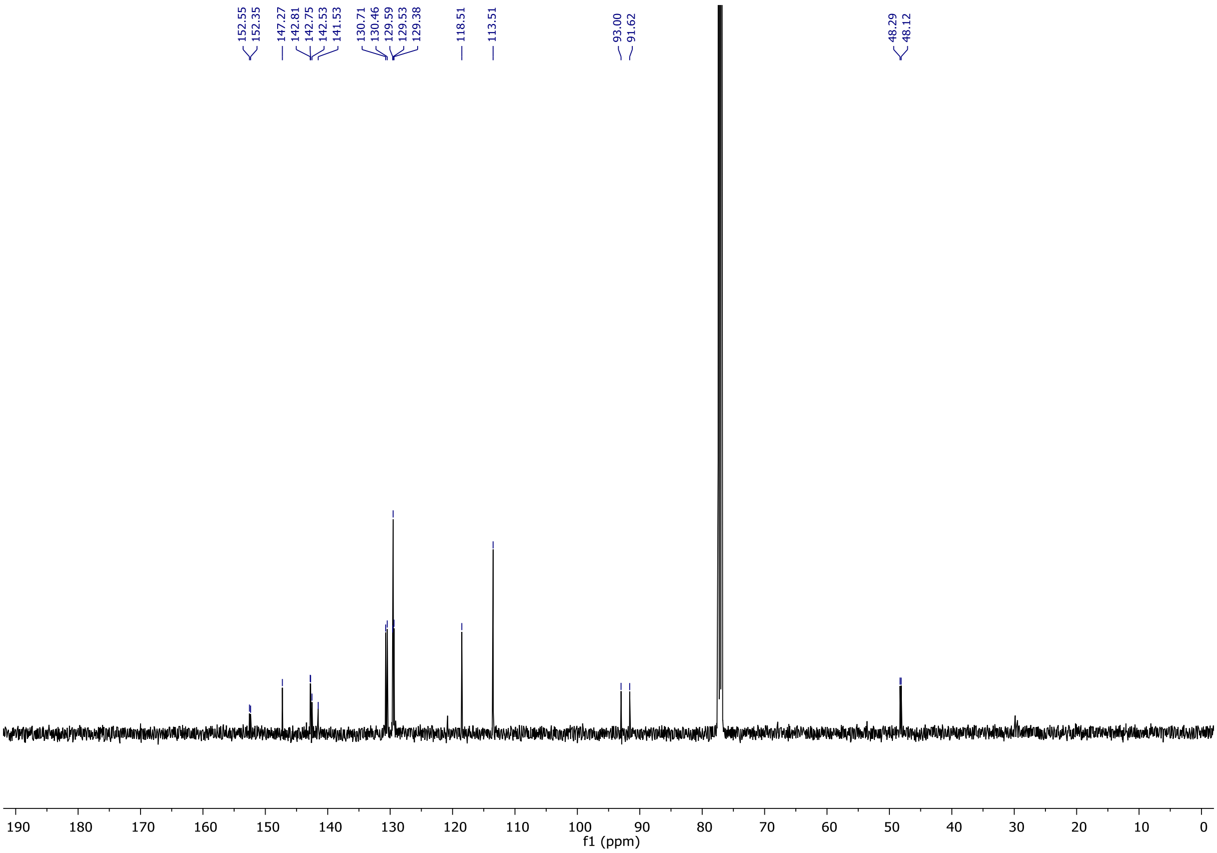

^{19}F NMR of 7r

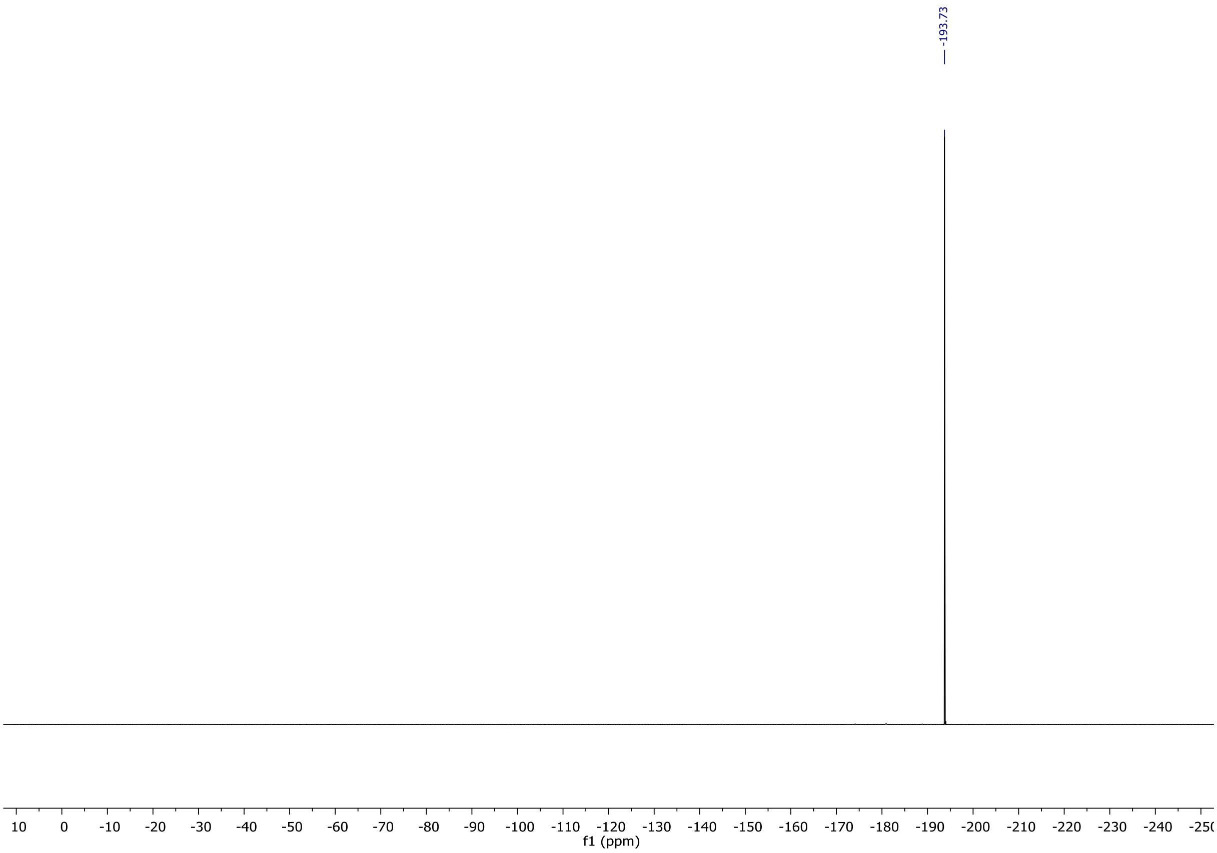

^{1}H NMR of 7s

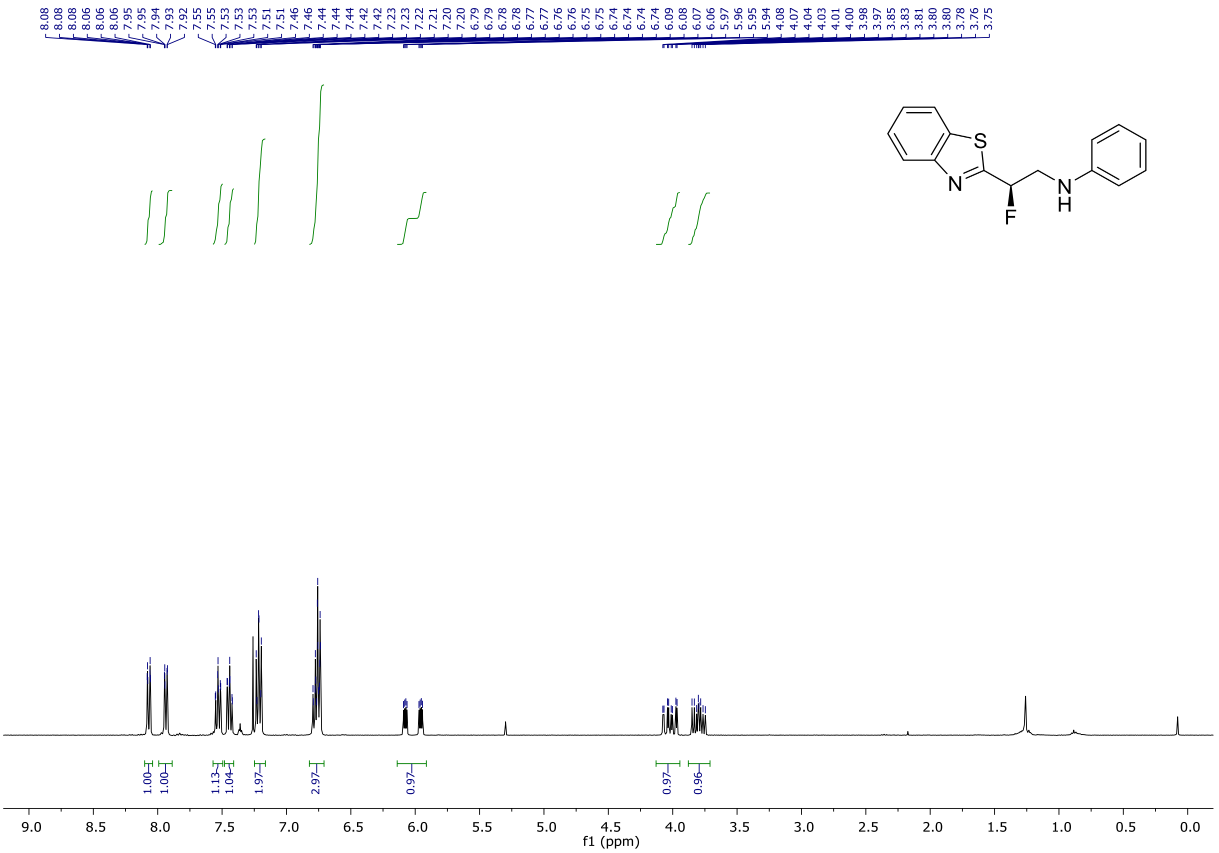

^{13}C NMR of 7s

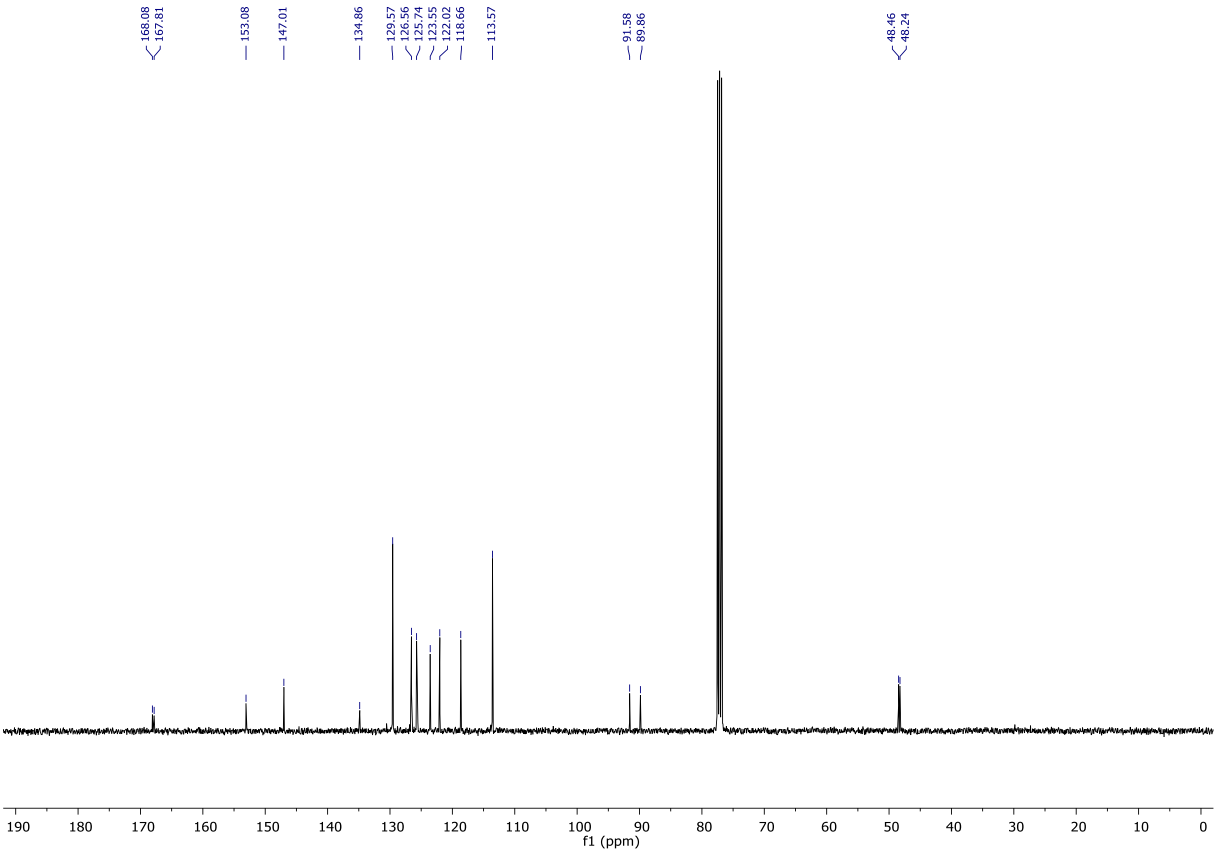

^{19}F NMR of 7s

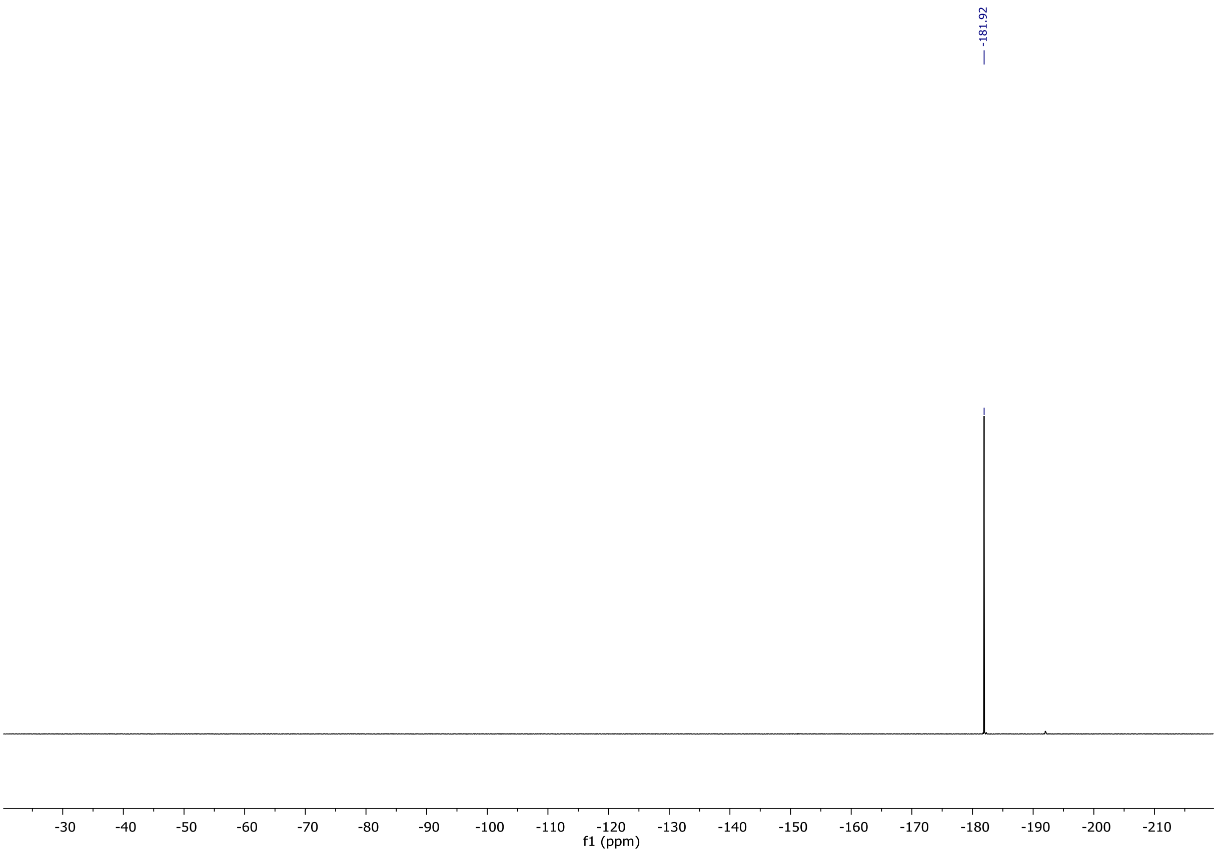

^{1}H NMR of 7t

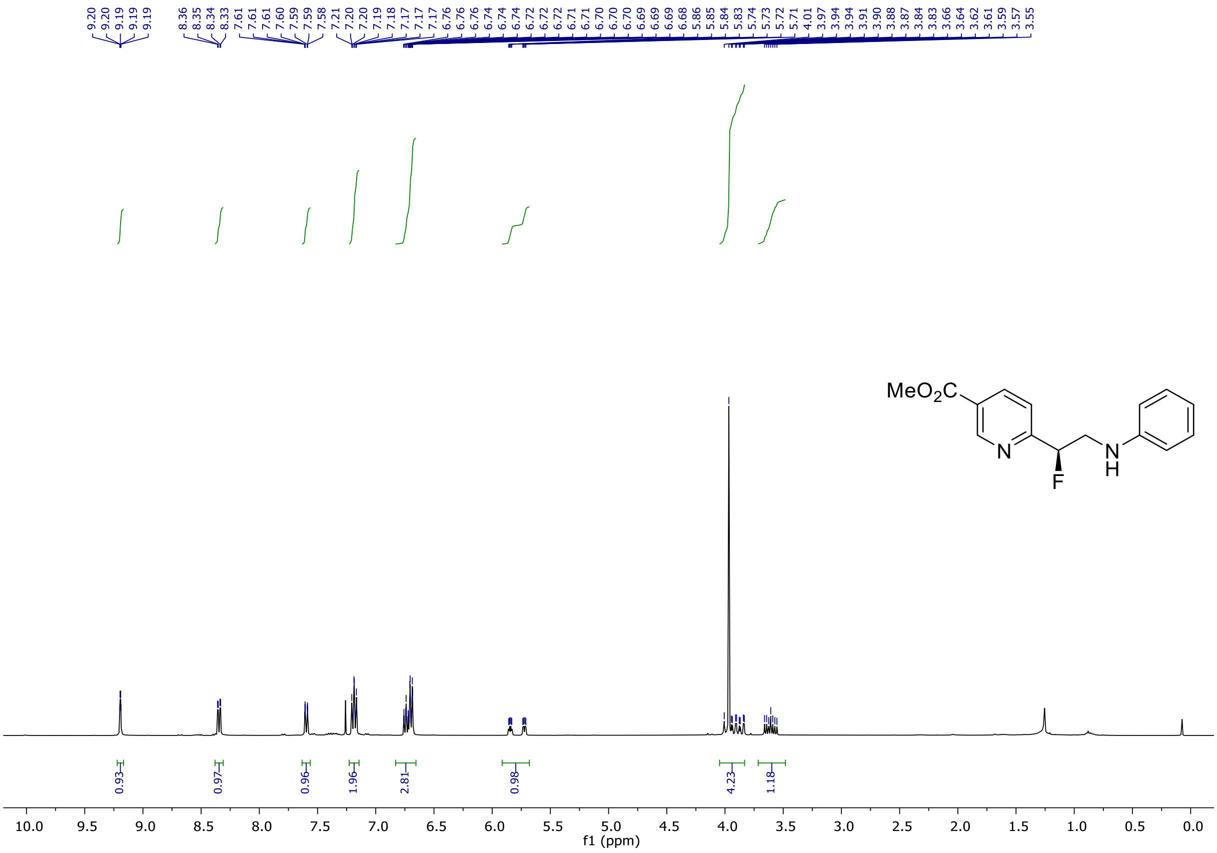

^{13}C NMR of 7t

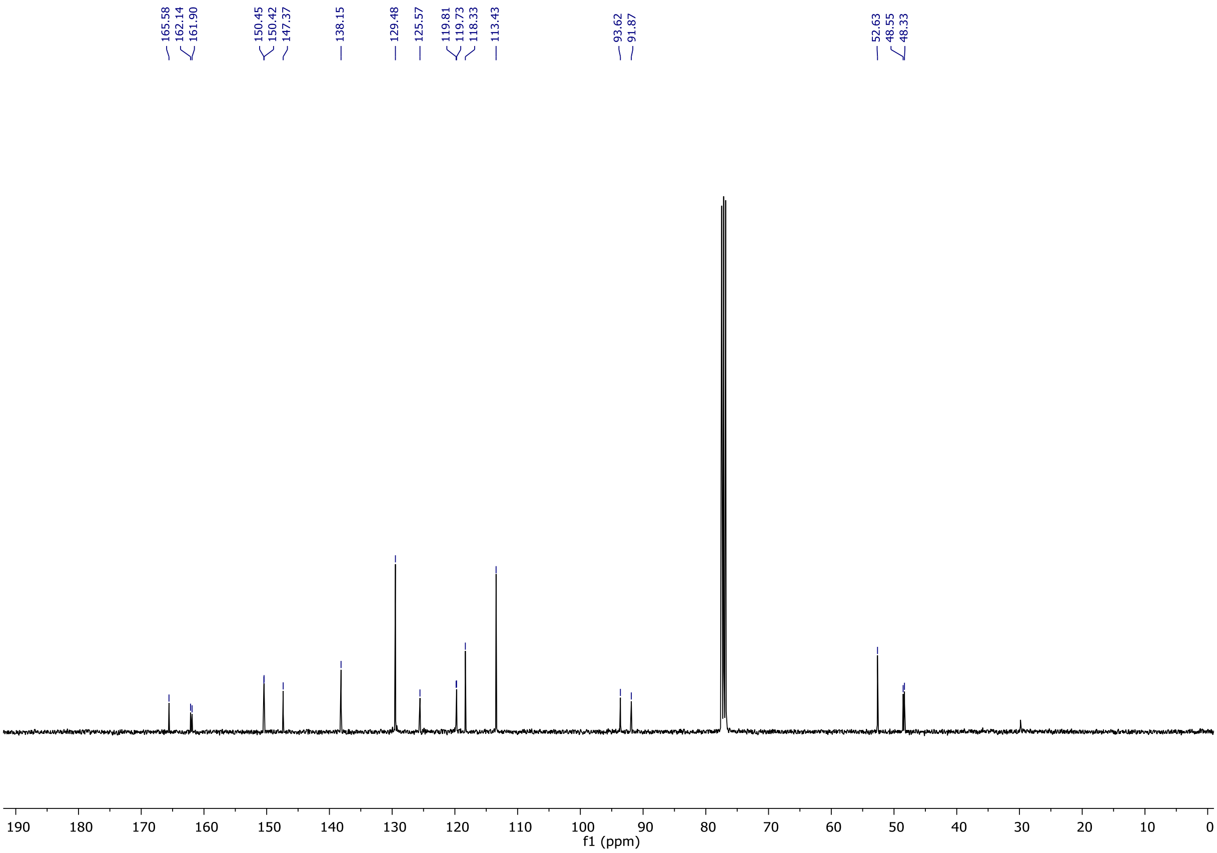

^{19}F NMR of 7t

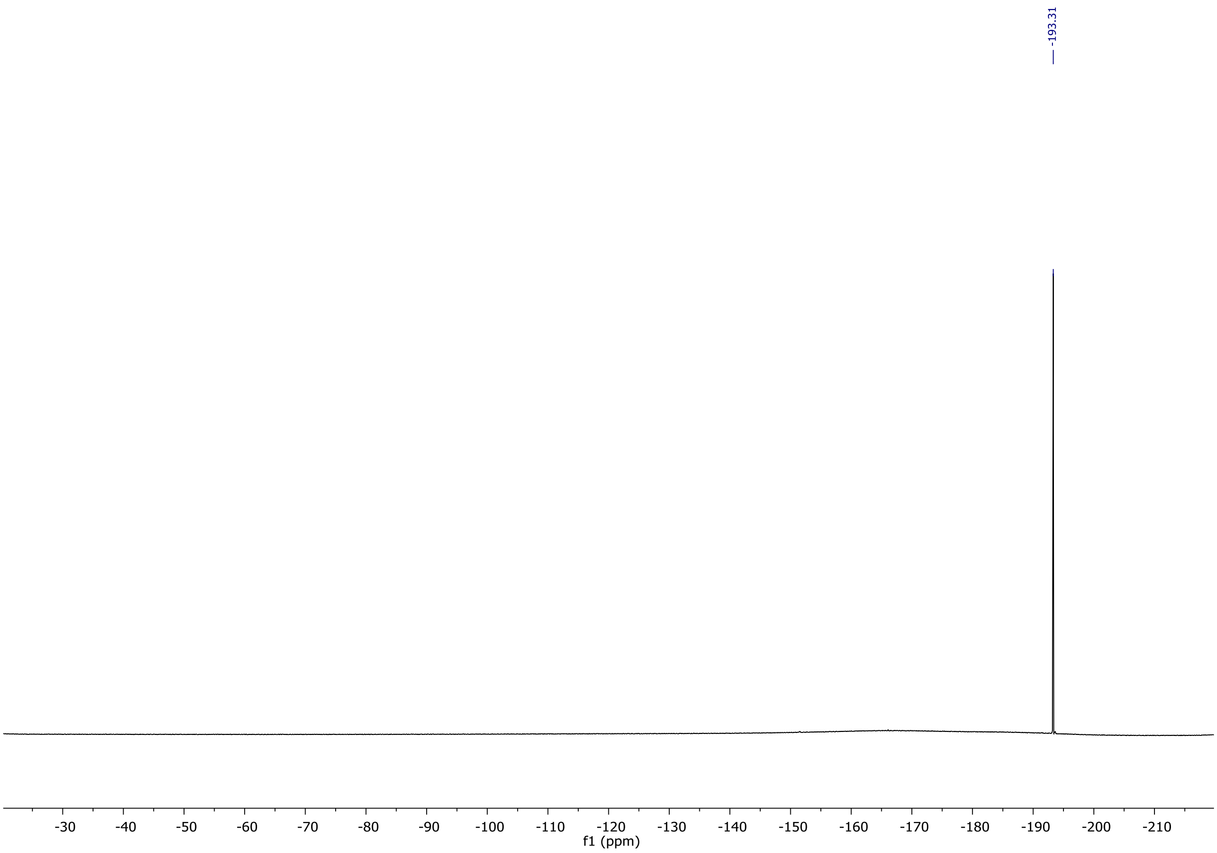

^{1}H NMR of 7u

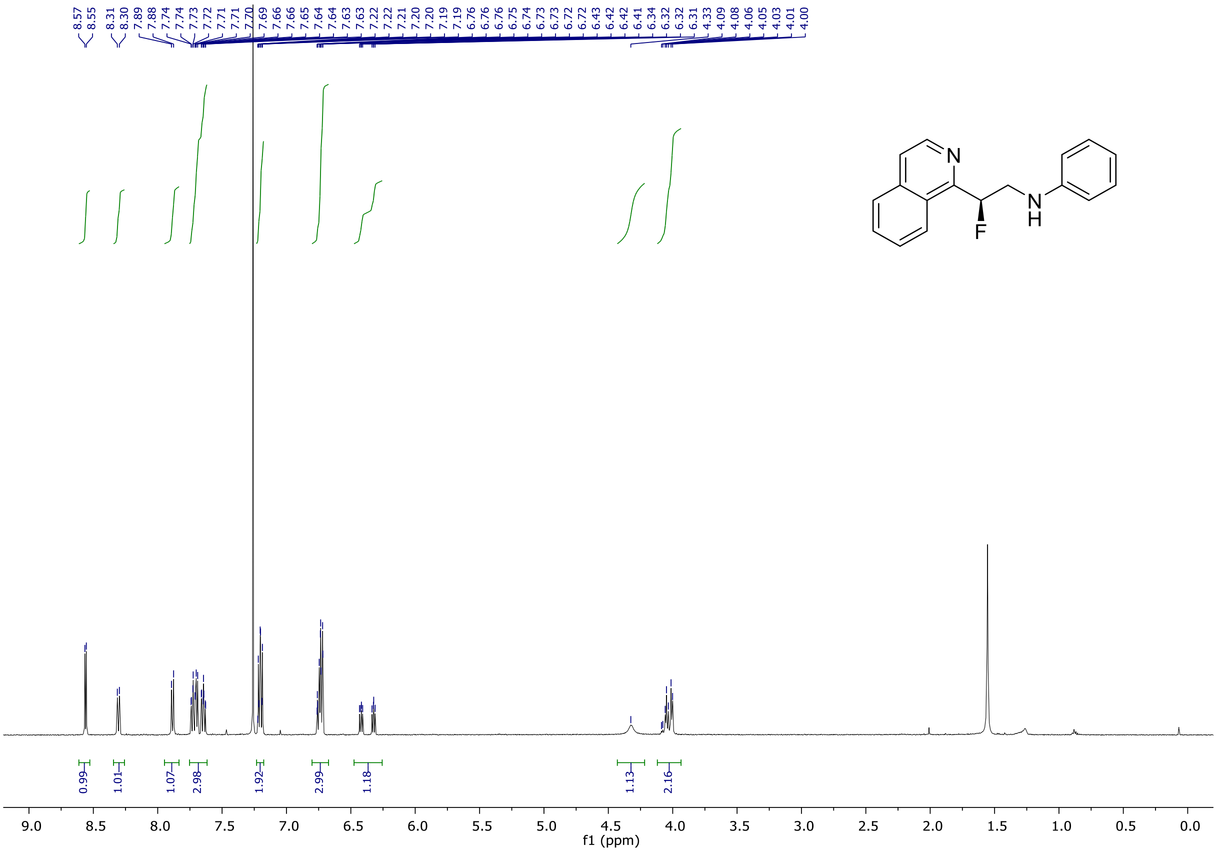

^{13}C NMR of 7u

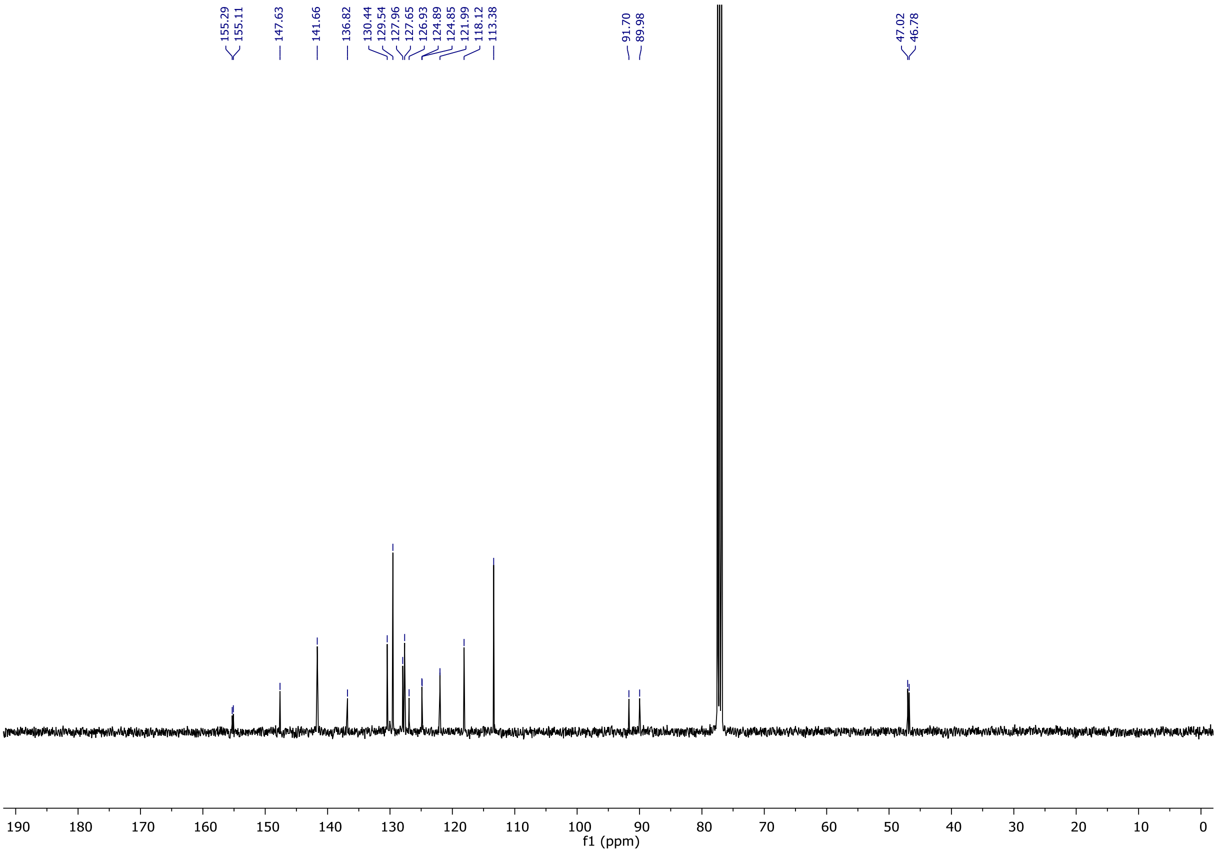

^{19}F NMR of 7u

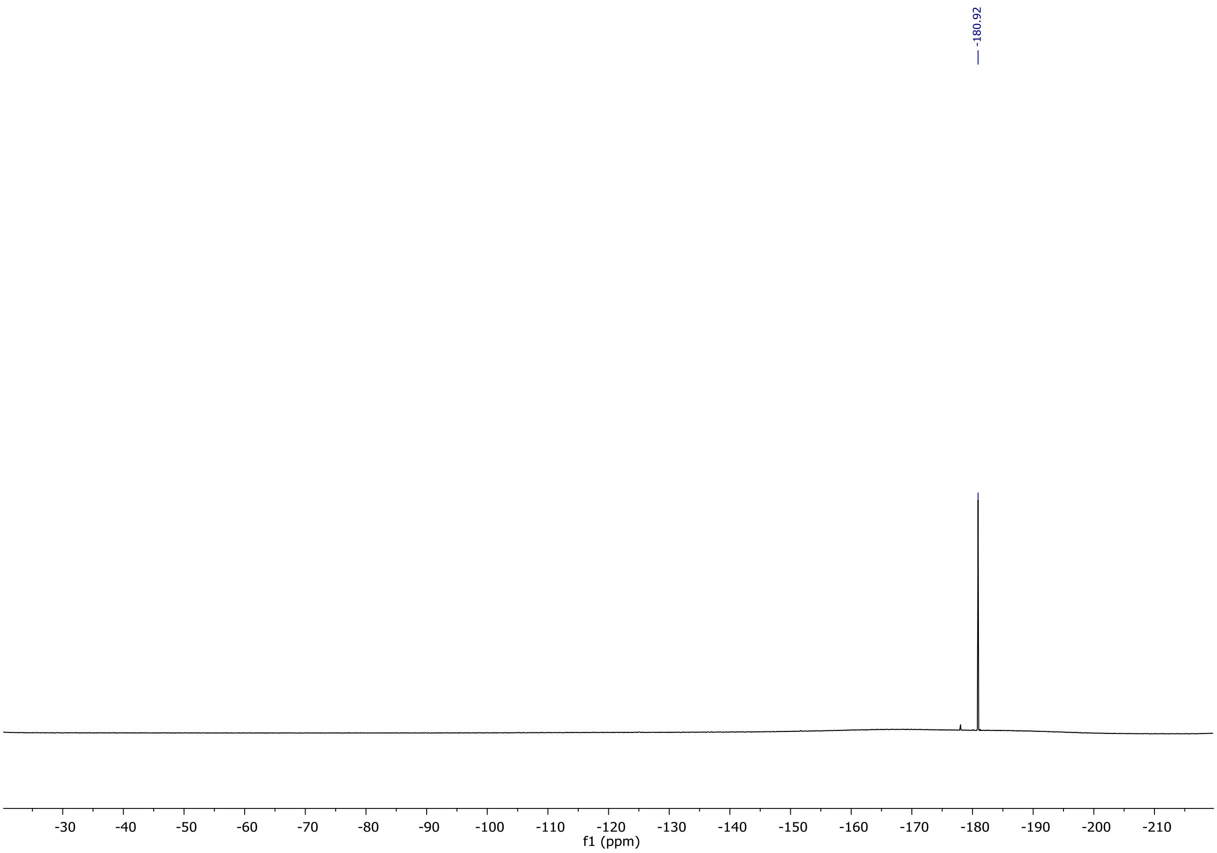

^{1}H NMR of 7v

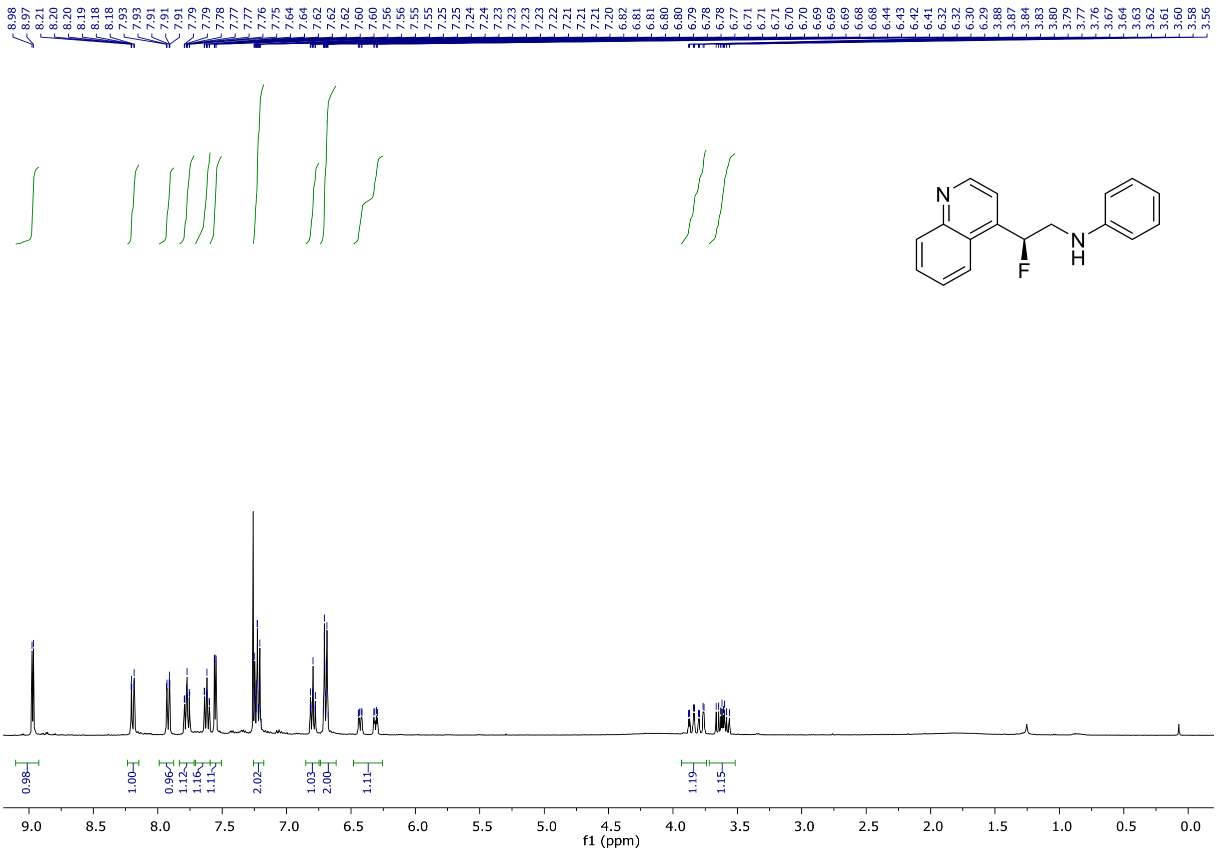

^{13}C NMR of 7v

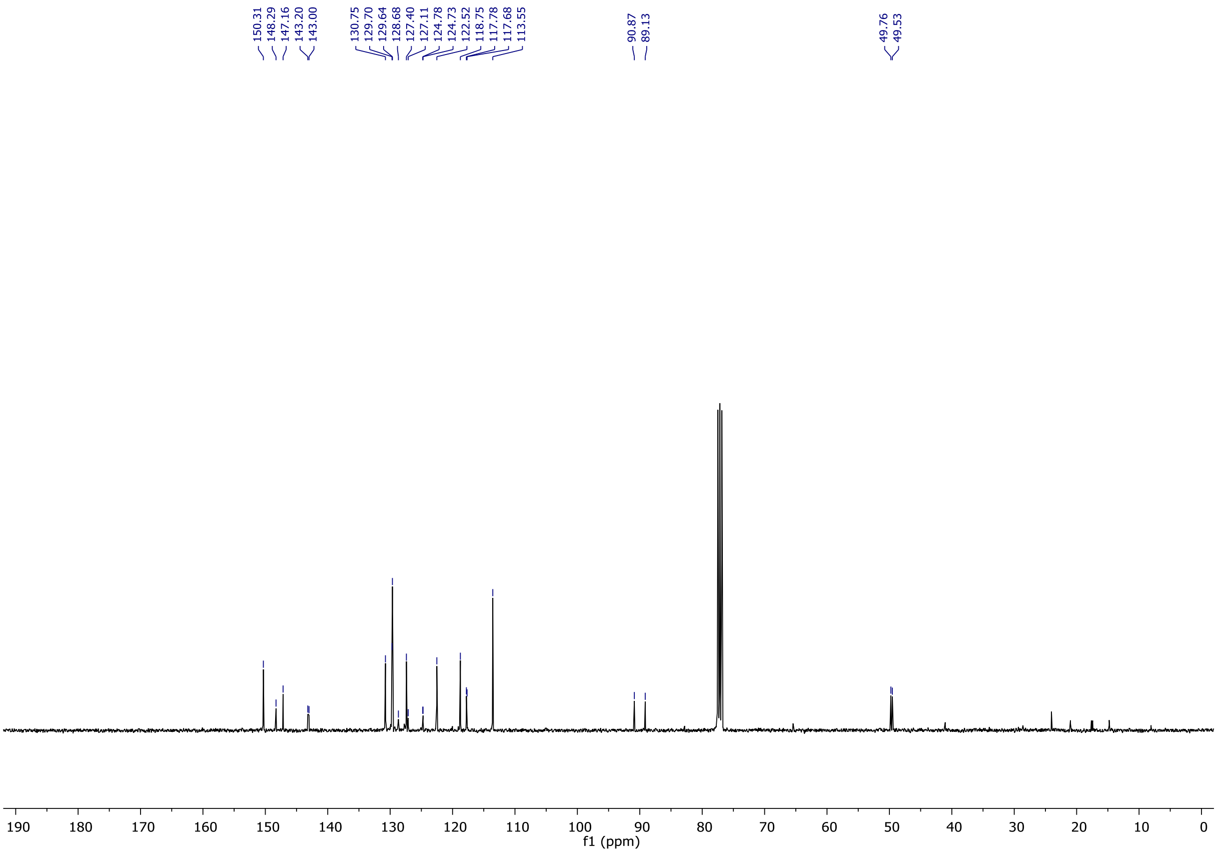

^{19}F NMR of 7v

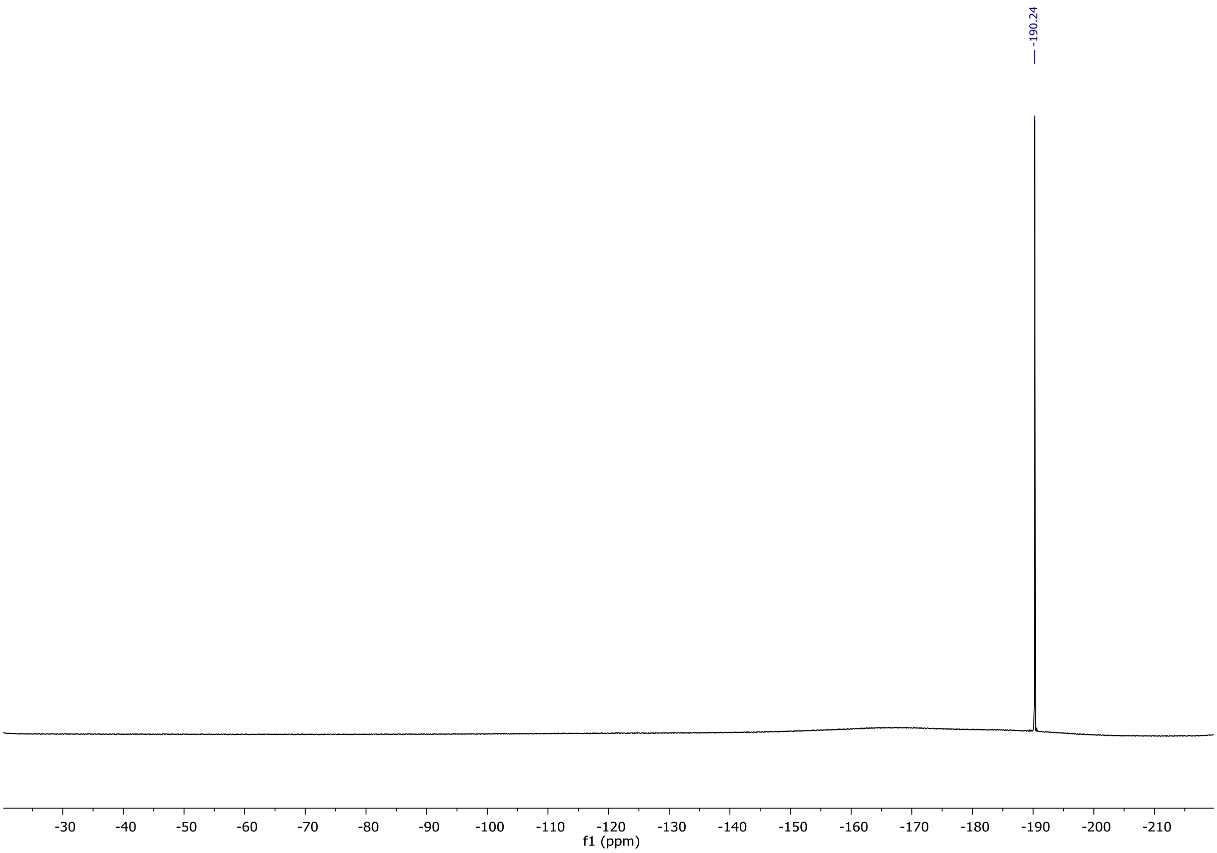

^{1}H NMR of 7w

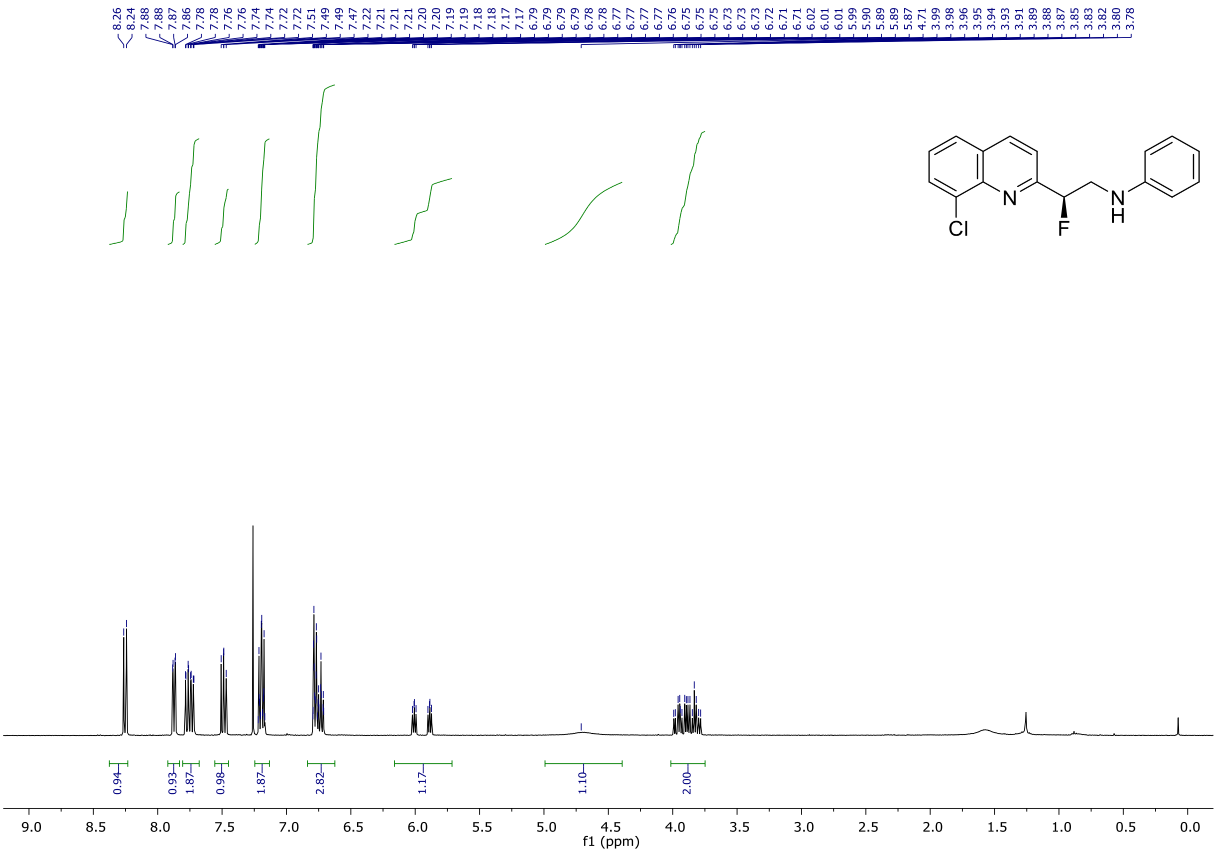

^{13}C NMR of 7w

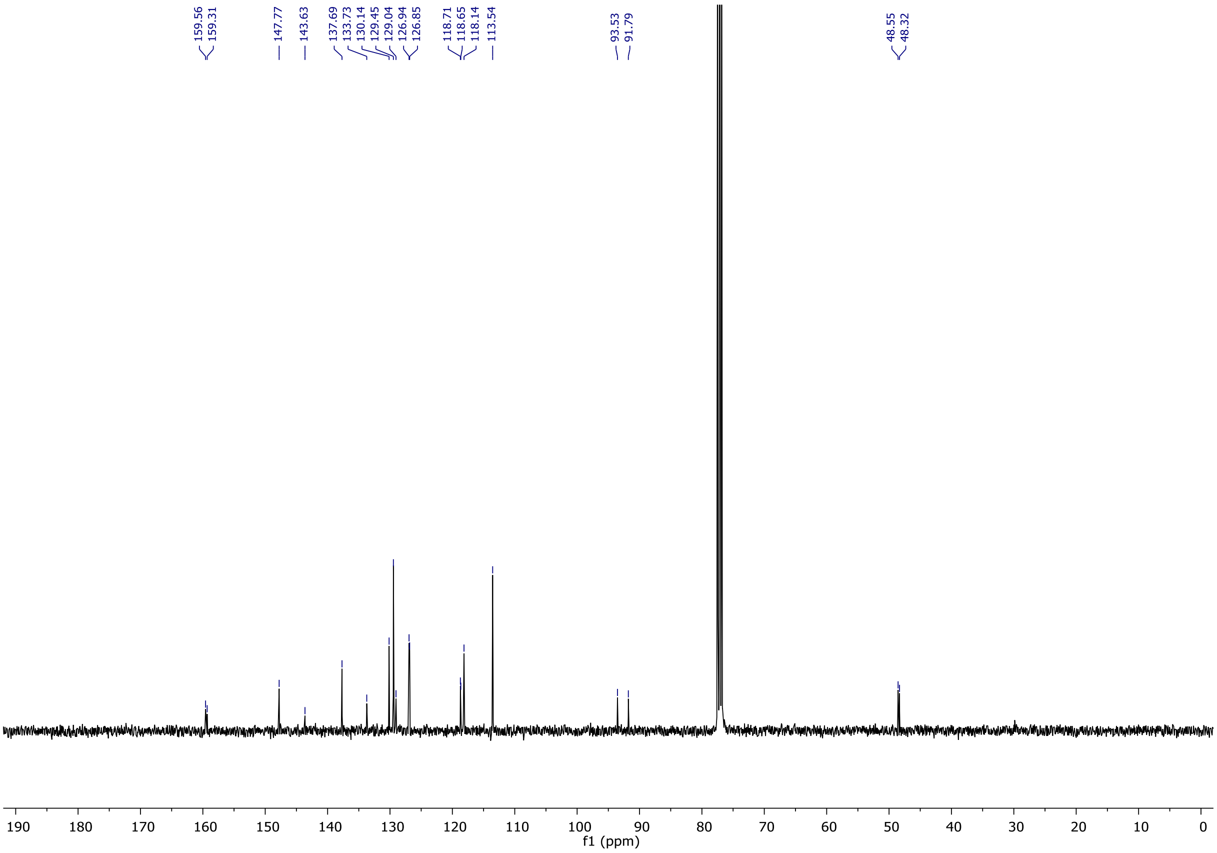

^{19}F NMR of 7w

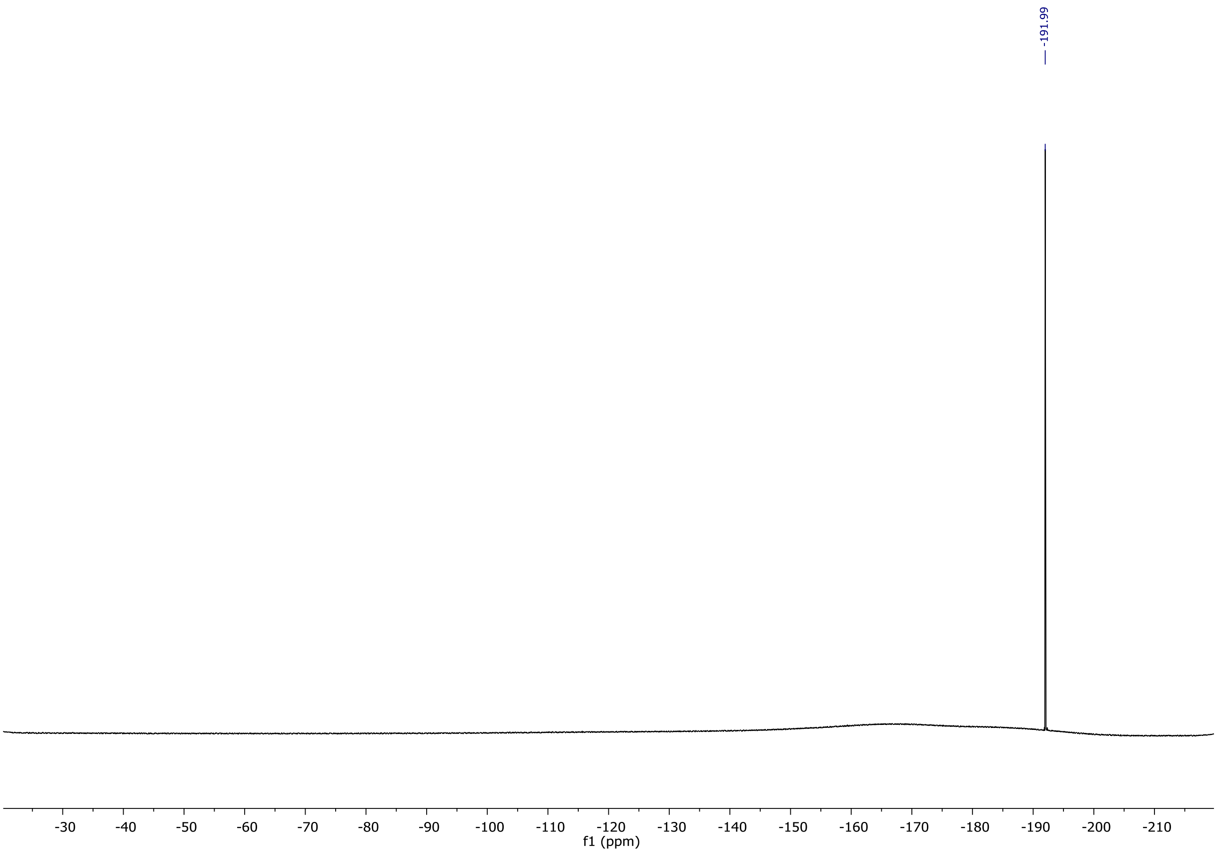

^{1}H NMR of 10

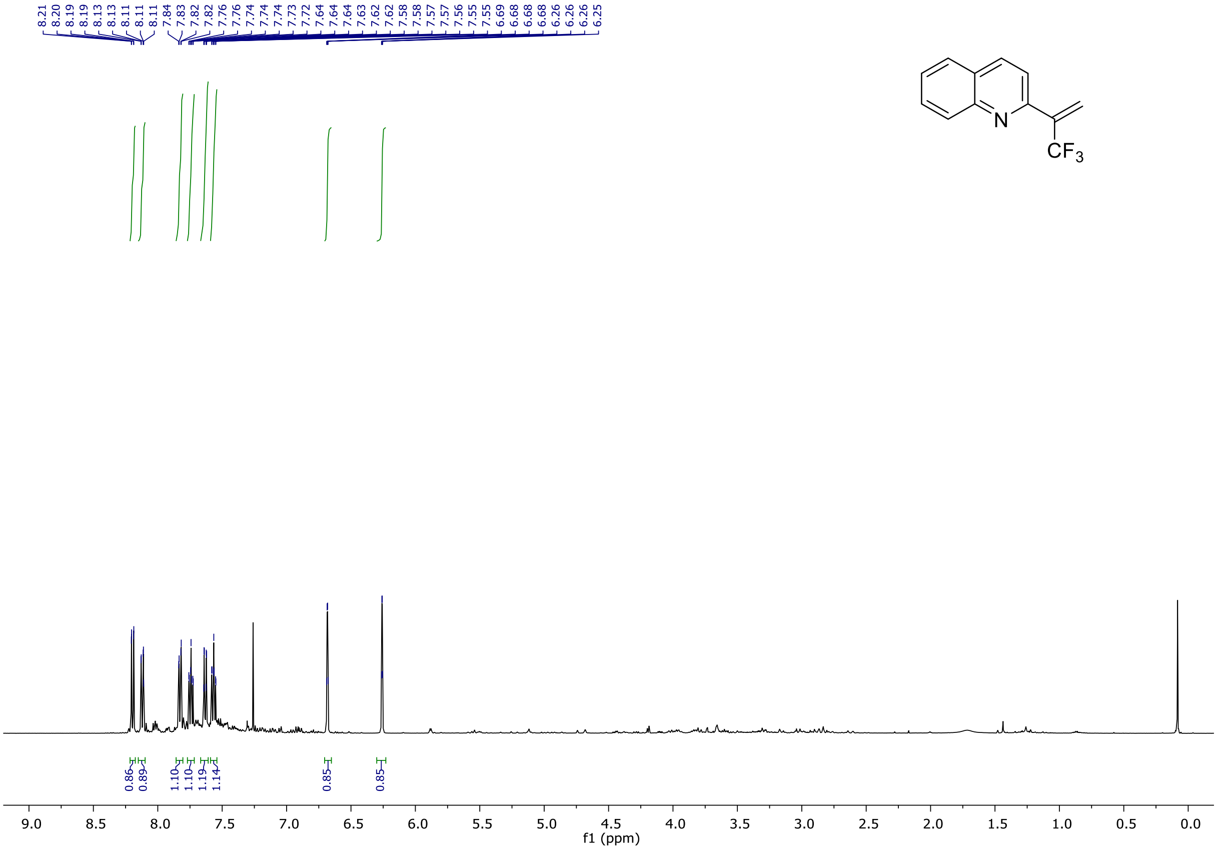

^{13}C NMR of 10

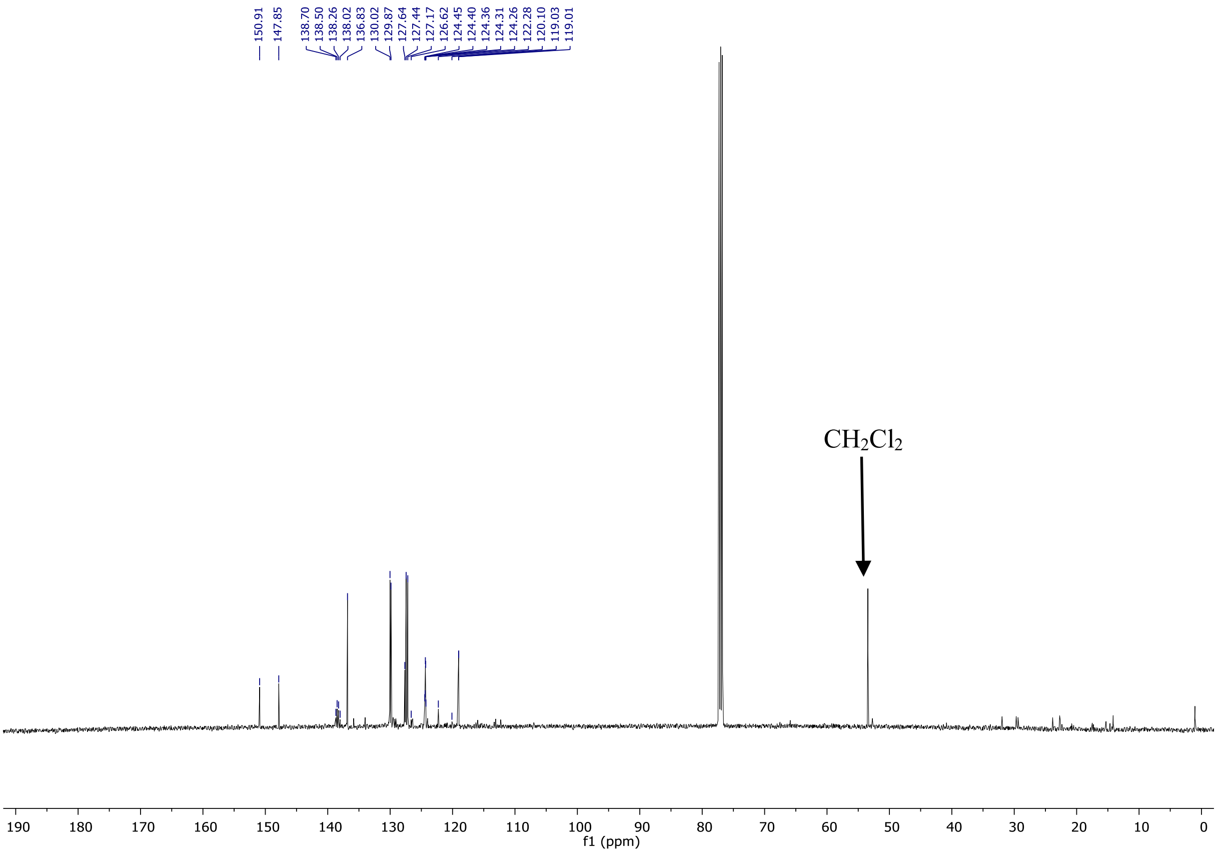

^{19}F NMR of 10

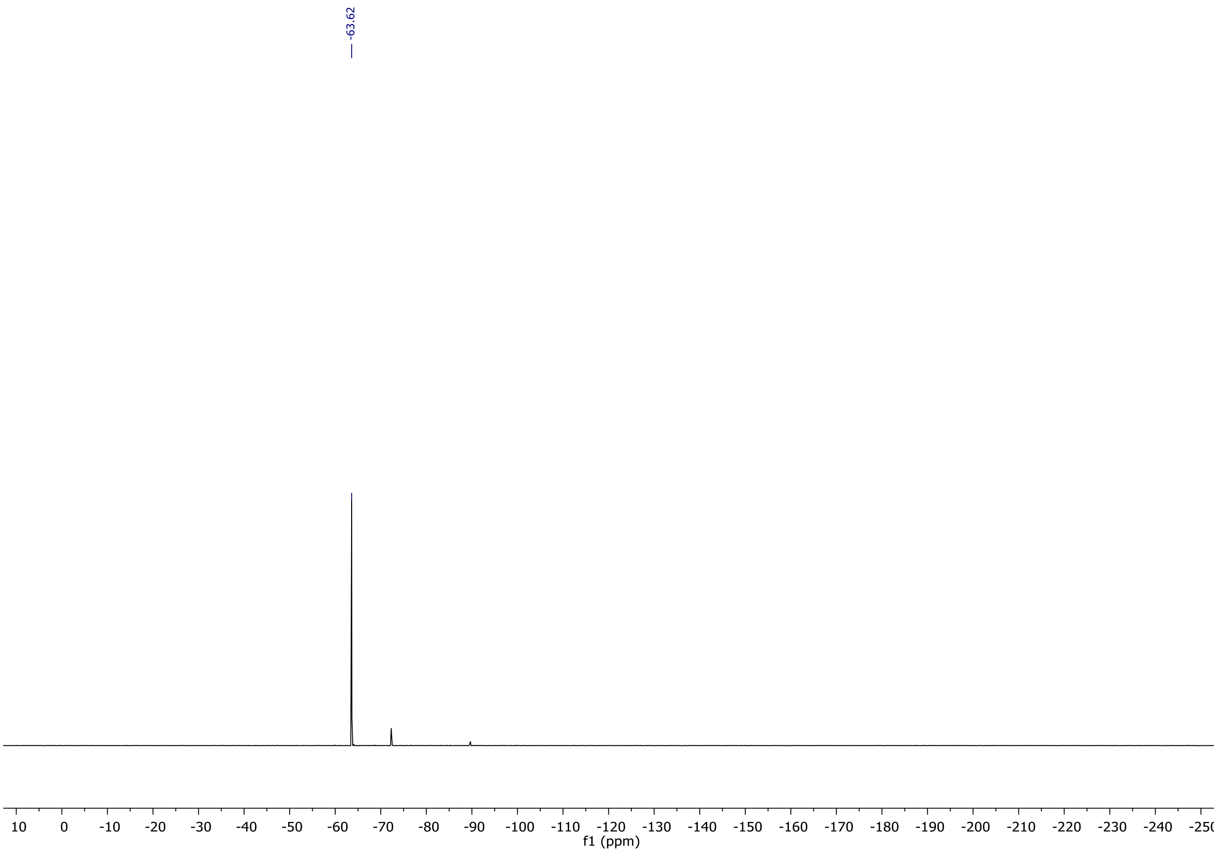

^{1}H NMR of 11

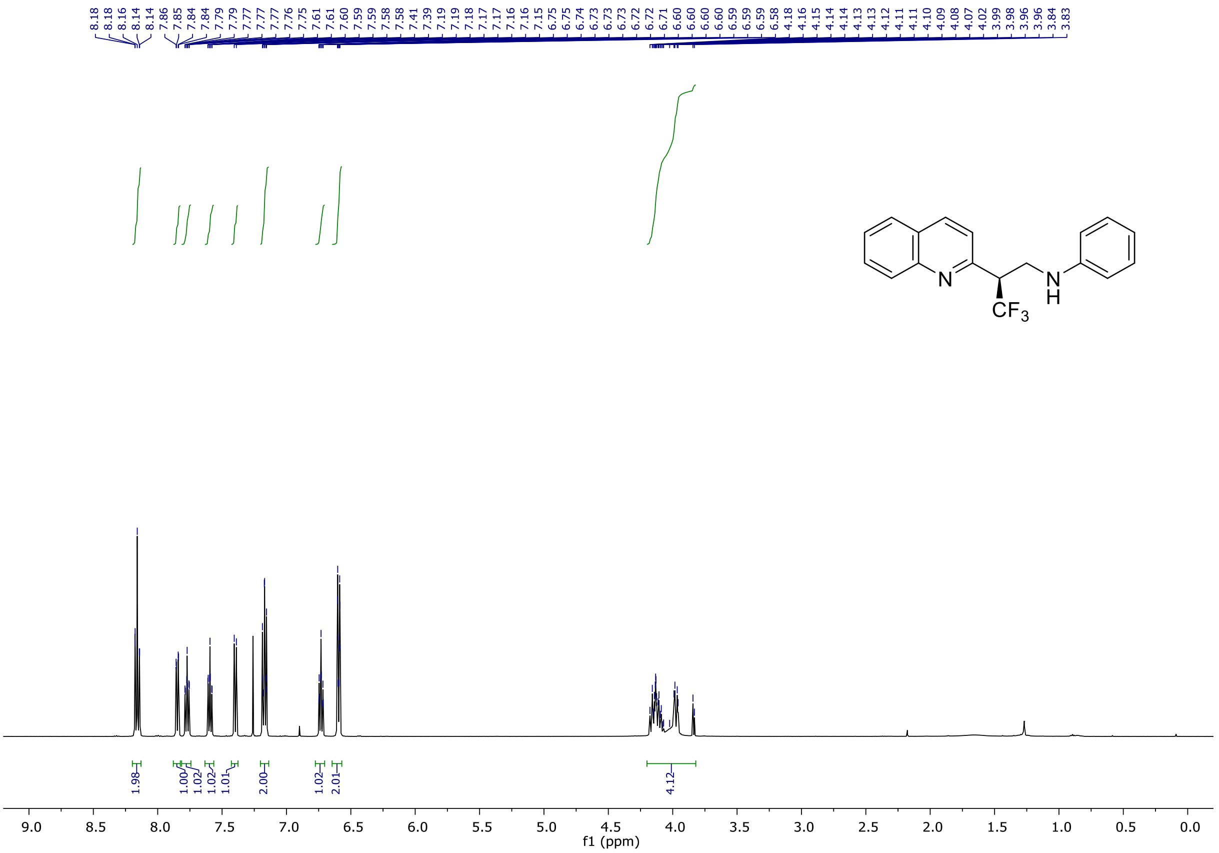

^{13}C NMR of 11

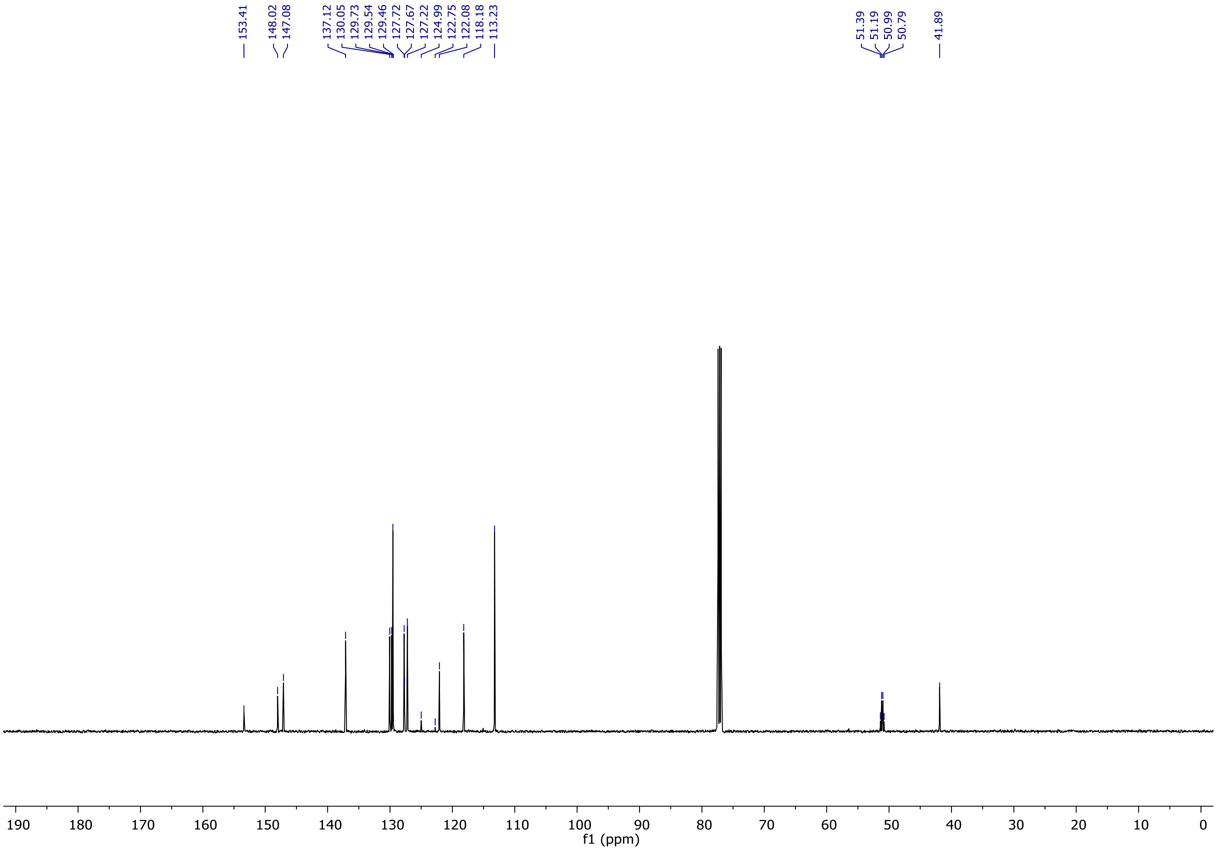

^{19}F NMR of 11

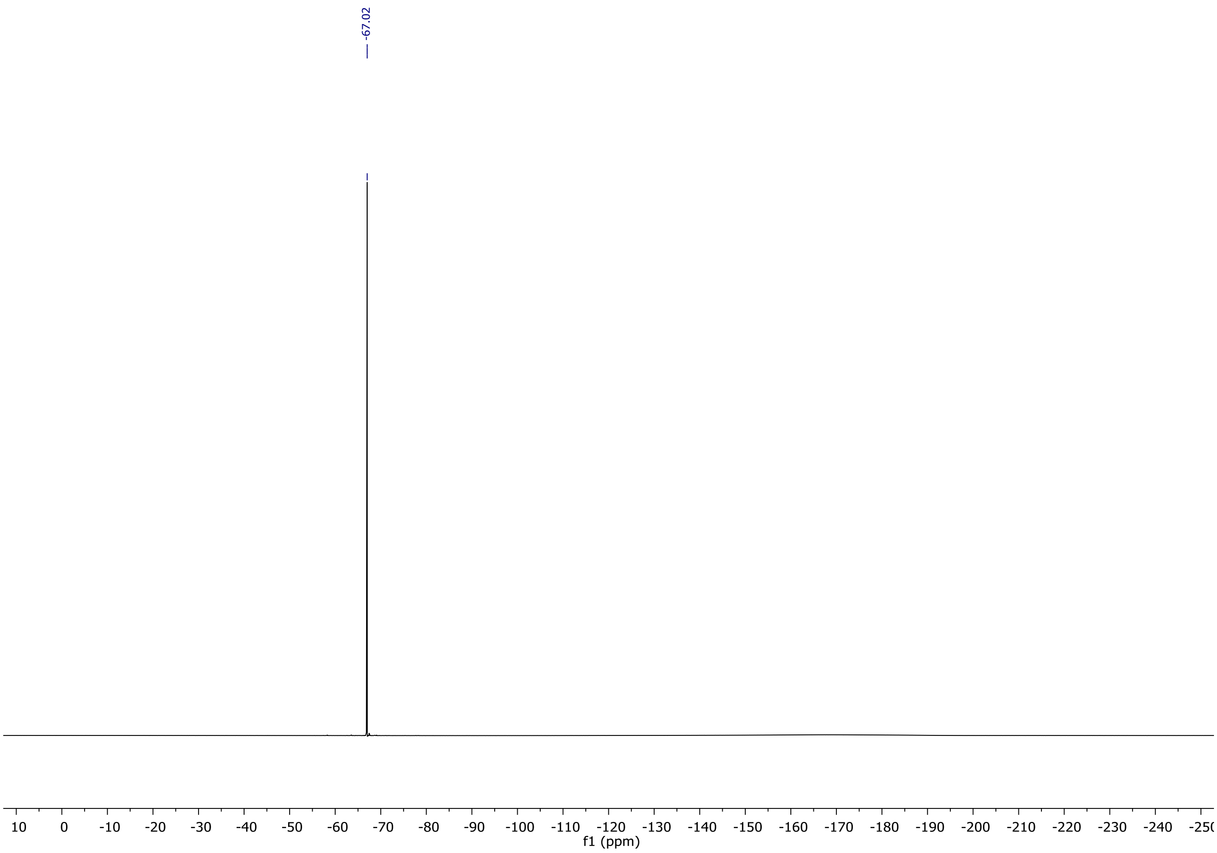

^{1}H NMR of 16

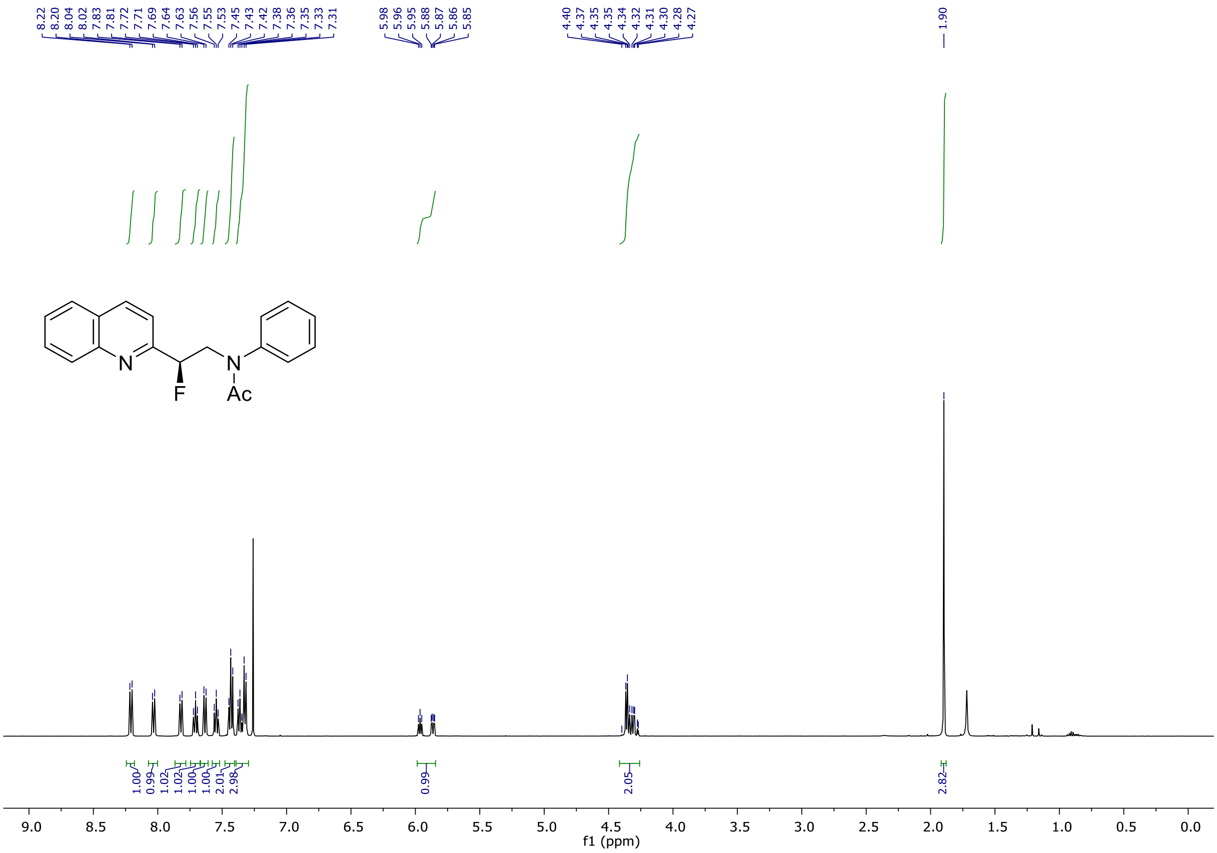

^{13}C NMR of 16

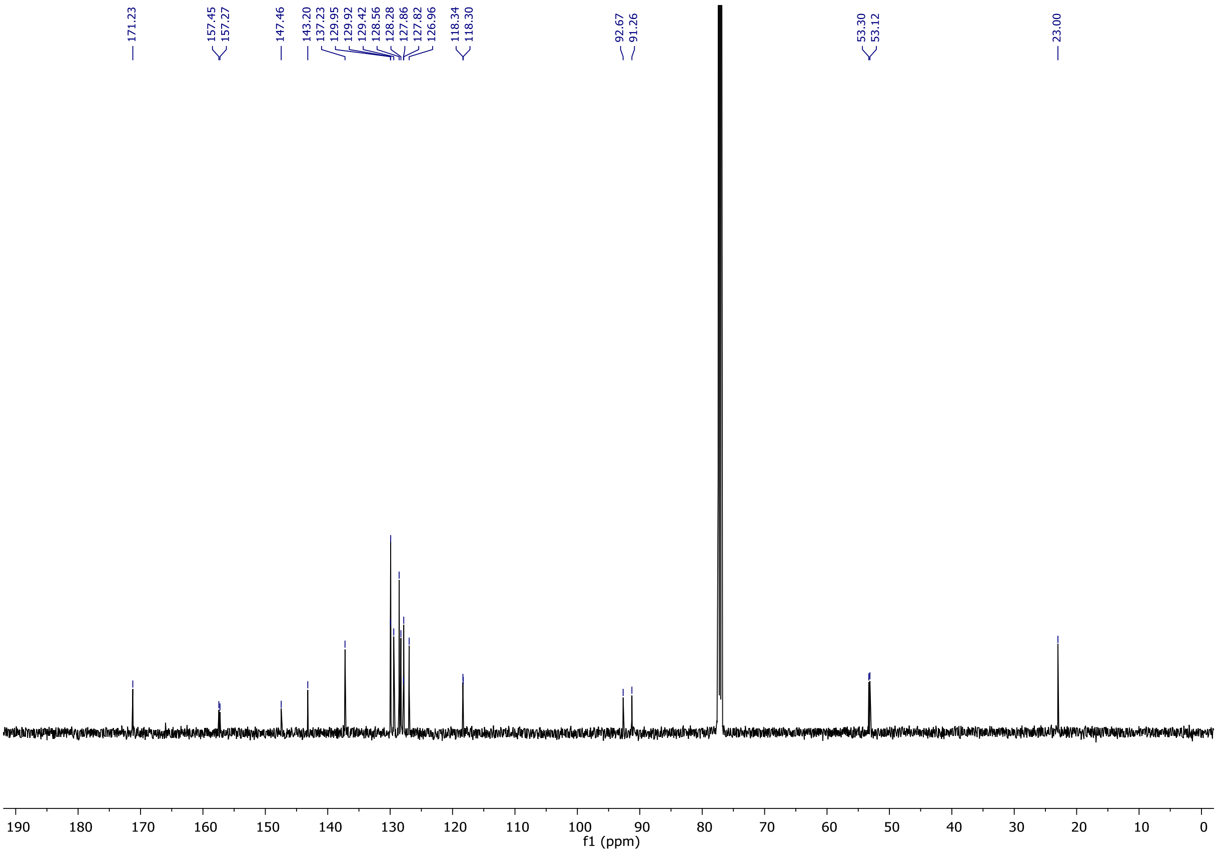

^{91}F NMR of 16

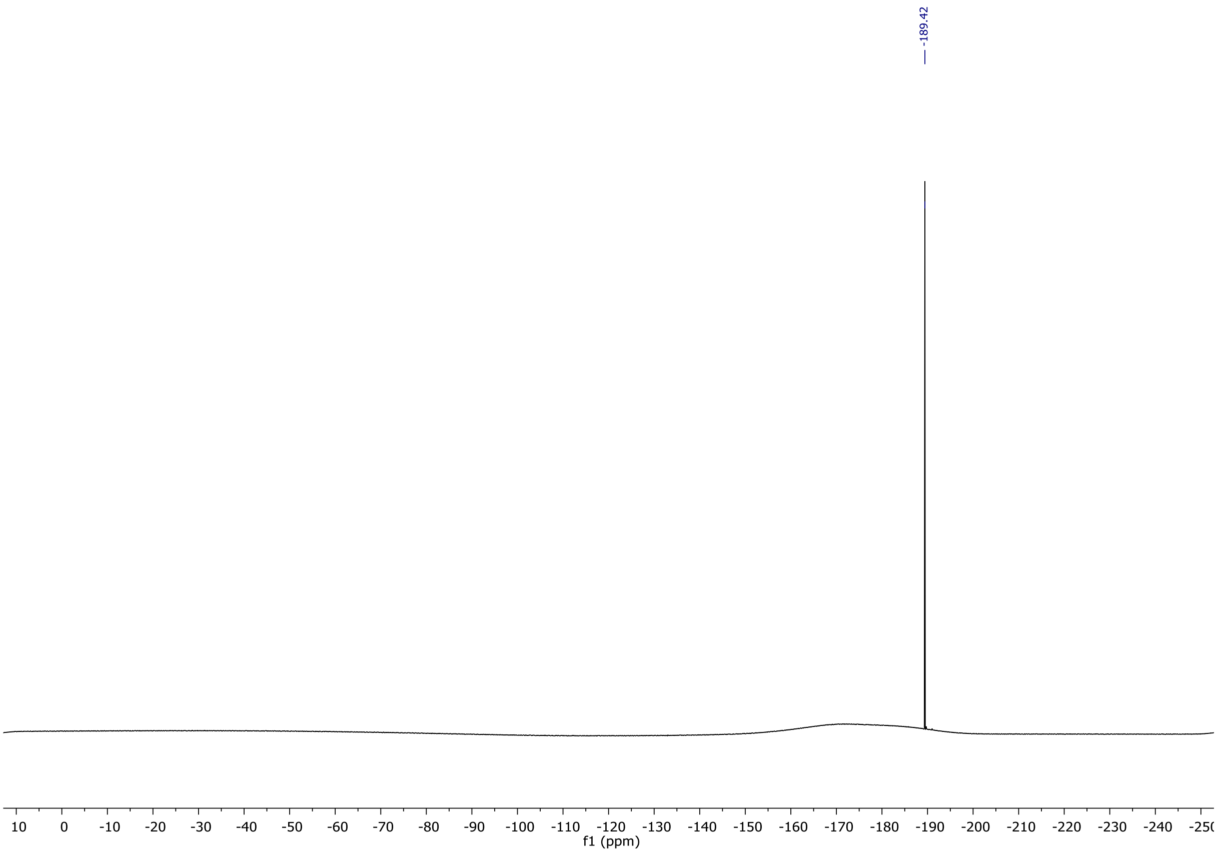

10. X-Ray Data

The datasets for all three compounds were collected using a Rigaku FR-X Ultrahigh Brilliance Microfocus RA generator/confocal optics with XtaLAB P200 diffractometer. The data for **7a** was collected at 125K with Cu radiation (λ = 1.54187 Å), whereas the data for **11** and **16** were collected at 93 K with Mo radiation (λ = 0.71075 Å). Intensity data were collected using ω steps accumulating area detector images spanning at least a hemisphere of reciprocal space. All data were corrected for Lorentz polarization effects. A multiscan absorption correction was applied by using CrysAlisPro. The structure of **7a** was solved using direct methods (SIR2011), whereas **11** and **16** were solved using dual space methods (SHELXT). The structures were refined by full-matrix least-squares against F^2 (SHELXL-2013). Non-hydrogen atoms were refined anisotropically, and hydrogen atoms were refined using a riding model, with the exception of H11 in **7a**, which was located via the electron density map. All calculations for **7a** were performed using the CrystalStructure interface and Olex2 for structures **11** and **16**. Selected crystallographic data are presented in Table 1. CCDC 1900849-1900851 contains the supplementary crystallographic data for this paper. The data can be obtained free of charge from the Cambridge Crystallographic Data Centre via www.ccdc.cam.ac.uk/structures.

	**7a**	**16**	**11**
Crystal data			
Chemical formula	$C_{17}H_{15}FN_2$	$C_{19}H_{17}FN_2O$	$C_{18}H_{15}F_3N_2$
M_r	266.32	308.35	316.32
Crystal system, space group	Orthorhombic, $P2_12_12_1$	Monoclinic, $C2/c$	Monoclinic, $P2_1$
Temperature (K)	125	93	93
a, b, c (Å)	5.44863 (10), 10.49560 (18), 23.5843 (4)	18.6107 (4), 7.75338 (14), 23.2547 (5)	10.1249 (3), 5.4916 (1), 13.3402 (5)
α, β, γ (°)	90, 90, 90	90, 111.212 (2), 90	90, 93.978 (3), 90
V (Å^3)	1348.71 (4)	3128.21 (12)	739.95 (4)
Z	4	8	2
Radiation type	Cu $K\alpha$	Mo $K\alpha$	Mo $K\alpha$
μ (mm^{-1})	0.71	0.09	0.11
Crystal size (mm)	0.12 × 0.10 × 0.10	0.27 × 0.18 × 0.06	0.36 × 0.09 × 0.03
Data collection			
T_{min}, T_{max}	0.889, 0.932	0.881, 0.995	0.871, 1.000
No. of measured, independent and observed reflections	15722, 2741, 2729	22904, 3460, 3247	8314, 2968, 2587
R_{int}	0.019	0.018	0.022
(sin θ/λ)$_{max}$ (Å^{-1})	0.628	0.664	0.660

Refinement			
$R[F^2 > 2\sigma(F^2)]$, $wR(F^2)$, S	0.027, 0.071, 1.09	0.040, 0.107, 1.02	0.039, 0.103, 1.06
No. of reflections	2741	3460	2968
No. of parameters	186	209	256
No. of restraints	1	0	8
$\Delta\rangle_{max}$, $\Delta\rangle_{min}$ (e Å^{-3})	0.16, −0.18	0.50, −0.31	0.24, −0.23
Absolute structure parameter	0.06 (4)	–	−0.2 (3)

Computer programs: *CrysAlis PRO* 1.171.39.8d (Rigaku OD, 2015), *CrysAlis PRO* 1.171.40.29a (Rigaku OD, 2018), *SIR2011* (Burla, *et al.*, 2012), *SHELXT* Version 2014/4 (Sheldrick, 2014), *SHELXT* (Sheldrick, 2015), *SHELXL* Version 2014/7 (Sheldrick, 2008), *SHELXL* (Sheldrick, 2015), Olex2 (Dolomanov *et al.*, 2009), *CrystalStructure* 4.2 (Rigaku, 2015), *CrystalStructure* 4.3 (Rigaku, 2018).
